# Proteomic Shifts Reflecting Oxidative Stress and Reduced Capacity for Protein Synthesis, and Alterations to Mitochondrial Membranes in *Neurospora crassa* Lacking VDAC

**DOI:** 10.3390/microorganisms10020198

**Published:** 2022-01-18

**Authors:** Sabbir R. Shuvo, Anna Motnenko, Oleg V. Krokhin, Victor Spicer, Deborah A. Court

**Affiliations:** 1 Department of Biochemistry and Microbiology, North South University, Dhaka 1229, Bangladesh; Sabbir.Shuvo@northsouth.edu; 2 Department of Microbiology, University of Manitoba, Winnipeg, MB R3T 2N2, Canada; Anna.Motnenko@umanitoba.ca; 3 Department of Internal Medicine, University of Manitoba, Winnipeg, MB R3T 2N2, Canada; Oleg.Krokhine@umanitoba.ca; 4 Manitoba Centre for Proteomics and Systems Biology, Winnipeg, MB R3E 3P4, Canada; Victor.Spicer@umanitoba.ca

**Keywords:** VDAC, *Neurospora crassa*, mitochondria, cytosol, proteomics, unfolded protein response, oxidative stress, ergosterol, hyphae, shikimate, amino acid biosynthesis

## Abstract

Voltage-dependent anion-selective channels (VDAC) maintain the bidirectional flow of small metabolites across the mitochondrial outer membrane and participate in the regulation of multiple cellular processes. To understand the roles of VDAC in cellular homeostasis, preliminary proteomic analyses of S100 cytosolic and mitochondria-enriched fractions from a VDAC-less *Neurospora crassa* strain (ΔPor-1) were performed. In the variant cells, less abundant proteins include subunits of translation initiation factor eIF-2, enzymes in the shikimate pathway leading to precursors of aromatic amino acids, and enzymes involved in sulfate assimilation and in the synthesis of methionine, cysteine, alanine, serine, and threonine. In contrast, some of the more abundant proteins are involved in electron flow, such as the α subunit of the electron transfer flavoprotein and lactate dehydrogenase, which is involved in one pathway leading to pyruvate synthesis. Increased levels of catalase and catalase activity support predicted increased levels of oxidative stress in ΔPor-1 cells, and higher levels of protein disulfide isomerase suggest activation of the unfolded protein response in the endoplasmic reticulum. ΔPor-1 cells are cold-sensitive, which led us to investigate the impact of the absence of VDAC on several mitochondrial membrane characteristics. Mitochondrial membranes in ΔPor-1 are more fluid than those of wild-type cells, the ratio of C18:1 to C18:3n3 acyl chains is reduced, and ergosterol levels are lower. In summary, these initial results indicate that VDAC-less *N. crassa* cells are characterized by a lower abundance of proteins involved in amino acid and protein synthesis and by increases in some associated with pyruvate metabolism and stress responses. Membrane lipids and hyphal morphology are also impacted by the absence of VDAC.

## 1. Introduction

The mitochondrial outer membrane (MOM) harbours mitochondrial porins, also known as voltage-dependent anion-selective channels (VDAC). These proteins are composed of 19 β-strands and an N-terminal α-helix [1,2,3,4] and form aqueous channels linking the intermembrane space and the cytosol [5]. In artificial membranes, the open conformation of VDAC is slightly anion-selective and a voltage-induced, partially closed confirmation is slightly cation-selective [6,7]. The N-terminus of VDAC is proposed to participate in the gating of VDAC between these two states [8,9,10]. *Neurospora crassa* is a useful model organism for the investigation of the functions of VDAC, as a single VDAC isoform is present [11]. The strain of *N. crassa* lacking VDAC (ΔPor-1) is viable but cold-sensitive and slow-growing, and it displays electron transport chain (ETC) defects and expresses alternative oxidase [12]. These changes are associated with increased potential for the production of reactive oxygen species (ROS) [13]. Thus, the absence of VDAC is associated with altered mitochondrial function and may be accompanied by stress responses.

Previously, iTRAQ [12] and one-dimensional liquid chromatography followed by tandem mass spectroscopy (1D LC-MS/MS) [14] were used to analyze the proteome of VDAC-less *N. crassa* mitochondria. A small number of proteins (Table 1) were identified as being over- or under-represented in the ΔPor-1 strain in these studies. In general, proteins involved in energy production, mitochondrial amino acid metabolism, and transport were more abundant; in contrast, some NADH–ubiquinone oxidoreductase (complex I) subunits were less abundant [14]. Although the same strains were used in both studies, some of the intermembrane space proteins that were relatively low in the iTRAQ experiments [12] had similar relative levels as those in the wild-type (WT) in the 1D LC-MS/MS study [14]. The flotation gradient step used to purify mitochondria more highly in the iTRAQ study might have caused additional disruption of the MOM in the VDAC-less strain, leading to under-representation of proteins from that compartment [14].

The impacts on other non-mitochondrial compartments and biochemical pathways were not examined in the previous studies of samples enriched for mitochondria. In the current study, the proteomic profiles of S100 fractions enriched in cytosolic proteins were examined and complemented with a preliminary, but deeper, 2D LC-MS/MS proteomic analysis of mitochondria-enriched samples to further enhance our understanding of the protein expression profile of the VDAC knockout *N. crassa* cells. The stress responses implicated by these data were examined in vivo. Finally, analyses of the acyl chains of membrane lipids and ergosterol were carried out to investigate the contributors to the cold sensitivity of the ΔPor-1 strain.

## 2. Materials and Methods

### 2.1. Chemicals

Unless otherwise mentioned, chemicals were obtained from Thermo Fisher Scientific (Mississauga, ON, Canada), Sigma-Aldrich Canada (Oakville, ON, Canada) or BioShop Canada (Burlington, ON, Canada).

### 2.2. N. crassa Strains and Growth Conditions

*N. crassa* strains FGSC 9718 (WT, Δ*mus-5::bar mat a*; [15,16] and ΔPor-1 (Δ*por::hph*^+^ Δ*mus51::bar*^+^ transformant of FGSC 9718) [12] were used in this study. Growth and handling of the *N. crassa* strains were performed following the procedures described in [17] at 30 °C.

### 2.3. Proteomic Analysis of Enriched Mitochondrial and S100 Cytosolic Fractions

Mitochondria were enriched from *N. crassa* cell extracts by following the differential centrifugation protocol described in [18,19]. To avoid damage to the membranes, mitochondria were prepared without a flotation gradient step. Samples were prepared for proteomic analysis as described in [20]. In brief, mitochondria-enriched samples were lysed with sodium dodecyl sulfate (SDS) and subsequently washed with urea buffer to remove SDS. Proteins were digested with trypsin (50:1 (*w*/*w*)) and then acidified using trifluoroacetic acid (final concentration 0.5%) and desalted using a 1 × 100 mm column packed with 5 µm of Luna C18(2) (Phenomenex, Torrance, CA, USA). Purified peptide mixtures were lyophilized and subjected to 2D liquid chromatography (LC) and tandem mass spectroscopy (MS/MS) following the method of [21].

Mass spectrometric (MS) acquisition was performed using an ABSciexTripleTOF 5600 TOF-MS system (Applied Biosystems, Foster City, CA, USA) equipped with a Nano-sprayIII ionization source. Each acquisition cycle included a 250 ms survey, MS acquisition (*m*/*z* 400–1500) and up to twenty 100 ms MS/MS measurements on the most intense parent ions (300 counts/s threshold, +2 to +4 charge state, *m*/*z* 100–1500 mass range for MS/MS). The numbers of MS/MS ions and peptides are listed in Table S1.

For isolating samples enriched in cytosolic proteins (S100), *N. crassa* cells were grown to exponential phase, and hyphae were harvested by filtration. Cells were ground with 1 g of sand in 1.5 mL of SEM buffer (250 mM of sucrose, 1 mM of EDTA, 9 mM of MOPS (3-[*N*-Morpholino]-propanesulfonic acid), pH 7.5) containing 1 mM of PMSF (phenylmethylsulfonyl fluoride) per 1 g of hyphae. Samples were then spun at 1900× *g* for 5 min in an SS34 rotor (Sorvall RC 6 Plus, ThermoFisher Scientific, Burlington, ON, Canada) to remove the cell debris and sand. Supernatants were collected and further clarified by spinning at 100,000× *g* (Beckman Coulter, Optima^TM^ Max-E Ultracentrifuge in a TLA100.3 rotor, (Beckman Coulter, LP, Mississauga, ON, Canada) for 45 min at 4 °C. This S100 fraction was enriched in soluble proteins, protein complexes, and ribosomes [22], and likely, due to rupture of organelles during isolation, contained some proteins from mitochondria, the endoplasmic reticulum and Golgi, and plasma membrane (see Table S2).

For sample preparation for mass spectrometry the following protocol was used [23]. The soluble proteins were reduced (10 mM of dithiothreitol, 30 min, 57 °C), alkylated (50 mM iodoacetamide, 30 min in the dark at room temperature), and dialyzed against 100 mM of NH_4_HCO_3_ for 6 h. Then, samples were digested overnight with modified trypsin (Promega, Madison, WI, USA) with a 1:100 (*w*/*w*) ratio of enzyme to substrate for 12 h at 37 °C. The resulting tryptic digests were acidified with trifluoroacetic acid (TFA) and purified by reversed-phase solid phase extraction. Approximately 50 µg of each digest, as determined by NanoDrop 2000 (ThermoFisher Scientific), was sufficient for each LC-MS experiment [21].

One-dimensional LC-MS/MS analyses were performed for 3 h of acquisition time for each sample. A splitless nano-flow 2D LC Ultra system (Eksigent, Dublin, CA, USA) was used to deliver water–acetonitrile gradient at 500 nL/min flow rate through a 100 µm × 200 mm analytical column packed with 3 µm of Luna C18(2) (Phenomenex, Torrance, CA, USA) at room temperature. Samples were injected (~1 µg of peptides in 10 µL of buffer A (water)) via a 300 µm × 5 mm PepMap100 trap-column (ThermoFisher Scientific). The gradient program included the following steps: linear increase from 0.5 to 35% of buffer B (acetonitrile) in 168 min followed by 5 min column wash with 90% buffer B and 7 min system equilibration using starting conditions of 0.5% buffer B. Both eluents A (water) and B (acetonitrile) contained 0.1% formic acid as the ion-pairing modifier.

Data-dependent acquisition with a TripleTOF5600 mass spectrometer (ABSciex, Framingham, MA USA) was performed using following settings: 250 ms survey MS spectra (*m*/*z* 375–1500), which was followed by up to 20 MS/MS measurements on the most intense parent ions (300 counts/sec threshold, +2 to +4 charge state, *m*/*z* 100–1500 mass range for MS/MS, 100 ms each, high sensitivity mode). Previously targeted parent ions were excluded from repetitive MS/MS acquisition for 12 s (50 mDa mass tolerance). See Table S1 for a summary of the data acquired.

For both data sets, the resulting raw WIFF files were processed using a standard conversion script, bundled with Analyst QS 1.6 (ABSciex) into Mascot Generic File format (MGF). MGF files contain information on charge, *m*/*z*, retention time of fragmented peptide as well as *m*/*z* values and intensities of fragment ions. Mass measurements for the parent and daughter ions were used for peptide identification, while intensities of the daughter ions were used for quantitation. Spectra (in MGF format) and an overall log2 protein expression matrix are available at the University of California, San Diego, in the MassIVE archive (massive.ucsd.edu) under the accession number MSV000088567.

An in-house GPU-based peptide identification engine [24] was used for protein identification, with the following search parameters: 20 ppm and 0.1 Da mass tolerance on the parent and fragment mass, respectively, and fixed modification of cysteine residues +57.021 Da (cysteine protection with iodoacetamide). Only tryptic peptides with expectation values of log(e) < −1.5 and with up to one permitted missed cleavage were used for identification. Sequence data were obtained from the 7th annotated version of the *N. crassa* genome, obtained from the *Neurospora crassa* Sequencing Project, Broad Institute of Harvard and MIT (http://www.broadinstitute.org/, accessed on 11 February 2016).

Protein total ion counts (TIC) were used as a relative measure of protein expression within an experimental run; these values represent the sum of the MS/MS fragment ion intensities for every identified member peptide. This value is expressed in a log_2_ scale. A single dataset was available for the mitochondria-enriched samples, which was analyzed following the methods described in [14]. These results are therefore preliminary. In short, for each protein identified in both the wild-type and mutant mitochondria, log_2_ difference values were determined and normalized to generate a mean near zero and standard deviation near one. z-Scores were calculated, and a minimum of 95% confidence interval was used to identify proteins differentially expressed in the two strains (−1.96 > z > 1.96, Table S3 [25]).

For cytosol-enriched S100 proteins, two samples were evaluated per strain (see Table S1 for scatter plots), and the relative abundance of each protein was calculated as described in [26,27]. In brief, the differences between the relative abundances (TIC) for replicates from the same strain (R0, R1) and between strains (Z0, Z1) were calculated. These populations of difference values were normalized to generate an average of 0, and the normalized populations were used to determine Wstat as a measure of the signal-to-noise ratio (S/N). A S/N value of 2.8 was chosen as the cut-off for significance and represents *p* < 0.05 [27]. The average TIC of the proteins within this group had a difference of at least two-fold (−1 > log_2_ > 1) between wild-type and mutant samples and was used for the current analysis (Table S4).

To estimate the contributions of non-cytosolic proteins to the S100 fraction, the list of 1037 proteins detected in both strains was compared to the list of putative mitochondrial proteins [14] and lists of *N. crassa* proteins obtained from UniProt, using the search parameters *Neurospora crassa* and 23-OR23-1a (the standard lab strain) as well as one of the following terms: “endoplasmic reticulum”, “Golgi”, “endoplasmic reticulum” or “lysosome”. We added a Supplementary Table (Table S2) that describes these data and presents the lists of sequences in all categories. Overall, this analysis showed 232 predicted mitochondrial proteins, 58 ribosomal proteins, 23 ER proteins, and 12 Golgi proteins, all detected in the S100 fraction of both strains.

### 2.4. Stress Analysis

To determine the sensitivity of the cells to tunicamycin, growth rates of the cells were measured in race tubes containing Vogel’s medium (VM) [17] in the presence of 2 µg/mL of tunicamycin [28], which induces a mild unfolded protein response in the endoplasmic reticulum of *N. crassa* [29].

Intracellular reactive oxygen species were measured following the protocol described in [30], with slight modifications. Cells were grown to exponential phase in 25 mL of VM at 30 °C and then incubated in 15 µM of 2′,7′-dichlorofluorescein (DCF) for 1 h. Cells were washed with 100 mL of water and then ground in liquid nitrogen with a mortar and pestle. A total of 15–25 mg of cell material was resuspended in 1 mL of phosphate buffer (10 mM of Na_2_HPO_4_, 1.8 mM of KH_2_PO_4_, 137 mM of NaCl, 2.7 mM of KCl, pH 7.4). The solution was diluted by five-fold in the same buffer, and fluorescence was measured at an excitation wavelength of 490 and an emission wavelength of 524 in a SpectraMax Plus 384 Microplate Reader (Molecular Devices Sunnyvale, CA, USA) and normalized to the mass of protein.

Catalase activity was determined by following the process, as described in [31], with slight modification. Hyphae of *N. crassa* were broken with a mortar and pestle as was performed for mitochondrial preparation (above). All centrifugation steps were conducted at 4 °C. Cellular debris and sand were removed by centrifugation at 1900× *g* at 4 °C for 5 min. The supernatant was collected, and mitochondria were pelleted by centrifugation at 17,200× *g* for 12 min. The pellet was resuspended in SEMP, and centrifugation was repeated for 20 min. Catalase activity was measured in a Gilson oxygraph equipped with Clark electrode. One unit of catalase is defined as the amount that decomposes 1 µmole of H_2_O_2_ in 1 min in a 60 mM of H_2_O_2_ solution at pH 7.0 [32].

### 2.5. Fatty Acid Analysis

Whole-mitochondrial lipid analysis was performed following the protocol described in [33], with slight modifications. Fatty acids were extracted from mitochondria (3 mg of protein) in 2 mL of 15% concentrated methanolic sulphuric acid and 2 mL of chloroform. An internal standard of C17:1 was used (Nu-Chek Prep Inc., Elysian, MN, USA) in the chloroform for quantification. Samples were heated at 90 °C for 3 h, and 1 mL of deionized water was used to separate the organic phase. The percentage of each acyl chain was calculated relative to the total peak area and correlated to the internal standard.

### 2.6. Sterol Analysis

Sterol analysis was based on the protocol described in [34]. Mitochondria (3 mg of protein) were heated in 3 mL of 10% methanolic KOH at 80 °C for 90 min. After cooling, 5α-cholestanol (250 ng) was added as an internal standard followed by 1 mL of water. Then, 3 mL of hexane was added and mixed with the solution, and 1 mL of the organic layer was collected and used for analysis by gas chromatography [35]. The area under the curve representing ergosterol was normalized to that from the internal standard, and the normalized values for sterol from the wild-type and ΔPor-1 strain were compared.

### 2.7. Fluidity Analysis

Mitochondrial fluidity was measured following with slight modifications of the protocol described in [36]. Mitochondria (100 µg of protein) were incubated in 2 mL of SEM buffer with 40 µL of 1 mM Laurdan (6-Dodecanoyl-2-dimethylamino-naphthalene, dissolved in dimethyl sulfoxide) in a Varian Eclipse spectrofluorometer (Varian Canada Inc., Mississauga, ON, Canada) at 15–58 °C. The excitation wavelength was 350 nm and emission (F) wavelengths were 440 and 490 nm; the ratio of F440/F490 was used as a relative indication of fluidity [36].

## 3. Results and Discussion

To further enhance the understanding of the global responses associated with the absence of VDAC in *N. crassa*, preliminary proteomic analyses of samples enriched in mitochondria, and fractions enriched in cytosolic proteins by centrifugation of cell lysates at 100,000× *g*, were carried out. Two strains were used: ΔPor-1, lacking VDAC [12] and the strain it is derived from, FGSC 9718 (WT). In the S100 sample, 1031 proteins were detected in both strains and of these, 232 were mitochondrial, 58 were ribosomal, 23 were associated with the endoplasmic reticulum, 12 with the Golgi, and 7 with the plasma membrane. No lysosomal proteins were detected (Table S2). This entire group of proteins was analysed, as the goal was to identify differences between the strains, rather than to explicitly examine individual cellular compartments or subfractions. A total of 170 of the 1031 proteins were differentially expressed in ΔPor-1 (Table S4). In the sample enriched for mitochondria, peptides from 2499 proteins were detected; of these 867 are known to be or predicted to be in mitochondria (Table S2; see [14]). A total of 60 of the mitochondrial proteins were differentially expressed (Table S3). This increased the depth of the data obtained in previous experiments (Table 1). The data from the current mitochondria-enriched and S100 cytosol-enriched samples were combined for analysis of pathways impacted by the lack of VDAC and are discussed together.

### 3.1. Less Abundant Proteins in ΔPor-1

**Figure 1:**
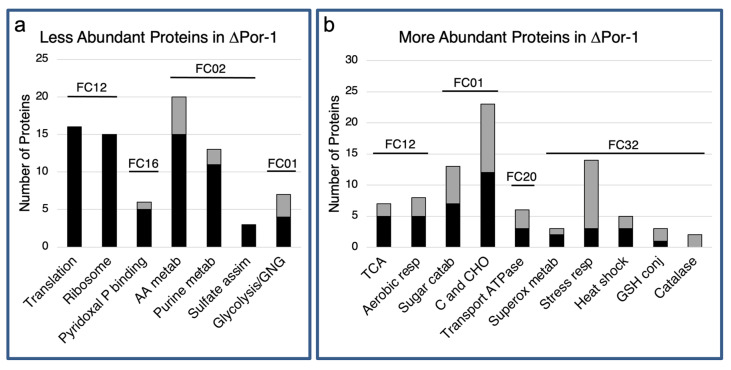
Classification of less and more abundant proteins in ΔPor-1. The proteins used in this functional category analysis were at lower (**a**) or higher (**b**) levels in the mutant than in WT cells (Tables S5 and S6). FungiFun2 (https://elbe.hki-jena.de/fungifun/fungifun.php, accessed on 25 November 2017) was used to predict the categories of the proteins highly represented among these proteins, by using the FunCat classification database [42]. This analysis highlights biological processes for which there is a relative enrichment of related proteins [42]; therefore, not all proteins in different amounts are represented in the output. Numbered lines above the bars indicate the main FunCat (FC) categories identified: 12, protein synthesis; 16, proteins with binding function; 02, energy; and 01, metabolism; cellular transport, transport facilitation and transport routes (20) and cell rescue, defence, and virulence (32). The black section of each bar indicates the number of S100 cytosol-enriched proteins, and the grey section indicates the mitochondrial proteins as defined in [14]. Abbreviations: (**a**) Pyridoxial P: pyridoxal phosphate binding proteins; AA metab: amino acid metabolism; Pu metab: purine nucleotide/nucleobase metabolism; sulfate assim: sulfate assimilation; glyco/GNG: glycolysis and gluconeogenesis. (**b**) TCA: tricarboxylic acid cycle; Aerobic resp: aerobic respiration; C and CHO: Carbon-compound and carbohydrate metabolism; Superox metab: superoxide metabolism; Stress resp: stress response; GHS conj: glutathione conjugation.

A total of 133 proteins were less abundant in ΔPor-1 cells than in WT cells (Table 1). Four main categories in the functional categorization (FunCat, FC) system [37] were represented: protein synthesis (FC 12), proteins with binding function or cofactor requirement (FC 16), energy (FC 02), and metabolism (FC 01) (Figure 1 and Table S5).

The low levels of proteins involved in translation (FC 12.04) and ribosomes (FC 12.01.01; see Table S5A) likely relate to the relatively slow growth rate of ΔPor-1 cells [12] and reduced demand for protein synthesis. Levels of individual mitochondrial and cytosolic ribosomal subunits were not uniformly reduced, but interestingly, the α, β and γ subunits of the eukaryotic initiation factor eIF-2 were all present in significantly lower amounts in ΔPor-1, suggesting reduced initiation on cytoplasmic ribosomes. This observation is intriguing, as the down-regulation of eIF-2 activity usually occurs via phosphorylation of eIF2-α, thereby preventing GDP-GTP exchange (reviewed in [38]).

**Figure 2:**
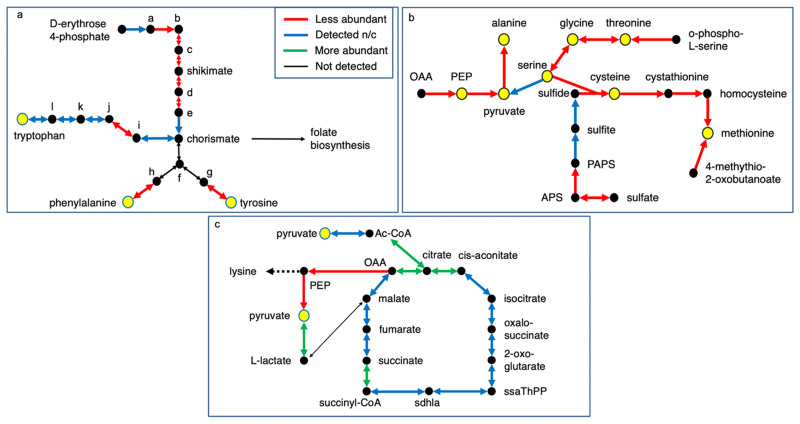
Metabolic pathway that includes proteins present at non-wild-type levels in ΔPor-1. Pathway details were obtained from KEGG pathways (https://www.genome.jp/kegg/pathway.html, accessed 21 November 2021, [44]) and only the segments of pathways that include proteins in relatively higher or lower amounts are shown for clarity. (**a**) Shikimate pathway and aromatic amino acid biosynthesis (KEGG pathways shikimate ncr00400 and sulfur amino acids ncr00920, respectively). Intermediates are as follows: panel A: a: 7-phospho-2-dehydro-3-deoxy-D-arabino-heptonate; b: 3-dehydroxyquinate; c: 3-dehydroshikamate; d: shikimate 3-phosphate; e: 5-O-(1-carboxyvinyl)-3-phosphoshikamate; f: prephenate; g: 4-hydroxy-phenylpyruvate; h: phenylpyruvate; i: anthranilate; j: N-(5-phospho-β-D-ribosyl)-anthranilate; k: 1-(2-carboxyphenylamino)-1′-deoxy-D-ribulose-5-phosphate. (**b**) Amino acid biosynthesis (ncr00260, ncr00270) and sulfur metabolism (ncr00920) APS: adenosine 5′ phosphosulfate; PAPS: 3′ phosphoadenosine 5′ phosphosulfate: OAA: oxaloacetate; PEP, phosphoenolpyruvate (**c**) TCA cycle and pyruvate metabolism (ncr00020 and ncr00620) AcCoA: acetyl coenzyme A; ssaThPP: succinate semialdehyde thiamine diphosphate; sdhla: s-succinyldihydrolipoamide-E.

Several pathways with significant coverage were explored further, with the understanding that protein levels are only one mechanism for controlling metabolism. In parallel to changes potentially impacting translation, proteins catalysing steps in amino acid synthesis are reduced in Δpor-1 (Table S5B; Figure 1), and this group includes enzymes involved in sulfate assimilation (01.02.03.04) and several enzymes that use pyridoxal phosphate as a cofactor (16.21.17; Table S5E). Notably, the shikimate pathway leading to chorismate, a precursor of tryptophan, tyrosine, and phenylalanine, was affected (Figure 2a), as were several enzymes linked to sulfate assimilation, sulfur amino acid synthesis (methionine and cysteine), and serine, glycine, and threonine synthesis (Figure 2b). This resembles general amino acid control (referred to as cross-pathway control (CRC) in *N. crassa*) [39,40]. However, CRC is achieved via phosphorylation of eIF2α, thereby enhancing translation of the upstream open reading frame (uORF)-containing mRNA for the transcription factor, CPC-1 [41], whereas a deficit in all eIF2 subunits may suggest a global decrease in translational initiation.

In terms of energy metabolism, the differential levels of some enzymes in ΔPor-1 can be expected to impact metabolism of pyruvate (Figure 2b,c). Increased levels of lactate dehydrogenase (Table S6) and decreased levels of pyruvate kinase and phosphoenolpyruvate carboxykinase (Table S5) could contribute to maintaining the pool of pyruvate. The enzymes in the TCA cycle were present at similar levels in the two strains, with the exception of succinyl CoA ligase (Figure 2c).

Several enzymes involved in different aspects of nucleic acid metabolism were differentially expressed, including adenylate kinase (Table S4), which is responsible for the interconversion of AMP, ADP, and ATP, which is a part of maintaining the energy balance in a cell (see [43]). Increased levels of adenylate kinase are compatible with the reduced state 3 respiration observed for ΔPor-1 mitochondria [13] (see below).

### 3.2. More Abundant Proteins in ΔPor-1

In the absence of VDAC, the more abundant proteins (Table S6) represent the following: FC 01, metabolism; FC 02, energy; FC 20, cellular transport, transport facilitation and transport routes, and FC 32, cell rescue, defence, and virulence (Figure 1b). In ΔPor-1, there were low levels of ETC complex I (NADH dehydrogenase) and low levels of the heme groups associated with complexes III (CoQH_2_-cytochrome c reductase) and IV (cytochrome c oxidase). In contrast, there were increased levels of cytochrome c, higher alternative NADH dehydrogenase activity, and alternative oxidase was expressed [12,13,14]. Together, these factors and others allow isolated ΔPor-1 mitochondria to maintain a normal membrane potential (Δψ) and oxygen consumption rate (OCR) in the presence of substrate (succinate) when ADP is not available, i.e., non-phosphorylating conditions (state 2 respiration) [3]. However, in the ΔPor-1 mitochondria, the addition of ADP to induce consumption of the potential by ATP synthase (phosphorylating conditions, state 3) is associated with smaller increases in OCR and smaller decreases in Δψ than occur in WT mitochondria [13]. This indicates insufficiencies in the ETC. As expected, other proteins that are more abundant in ΔPor-1 are linked to the electron flow and the ETC–protoheme IX farnesyltransferase, involved in heme a (cytochrome oxidase) synthesis [45], a Mam33 homologue involved in mitochondrial ribosome synthesis [46], the α subunit of the electron transfer flavoprotein, which interacts with multiple dehydrogenases [47], two subunits of the ATP synthase, and the cytochrome c apoprotein (Table S6).

Over-represented proteins in FC 20 (cellular transport, transport facilitation and transport routes; Table S6C) did not include an obvious candidate to replace VDAC as a general transport pore in the MOM. They did include components of the F-type mitochondrial ATPases and may relate to defects in oxidative phosphorylation [13]. Several subunits of vacuolar ATPases were also increased in ΔPor-1. These proteins generate an electrochemical gradient to transport molecules into vacuoles and create an acidic environment for the hydrolytic enzymes therein [48]. Degradation in vacuoles and recycling of the cellular materials [49] may be important for the survival of ΔPor-1 cells. The remaining types of ATP transport proteins are members of the ABC and multidrug transporter families, but their substrates are not known.

More abundant proteins in the FunCat category FC 32: cell rescue, defence, and virulence; include catalases, involved in superoxide metabolism, and proteins in the glutathione conjugate reaction (Table S6D). Increased intracellular ROS has been observed in Saccharomyces cerevisiae lacking VDAC [50], and VDAC may be involved in the release of superoxide from mitochondria [51,52]. Enhanced cytosolic activity was detected in ΔPor-1 compared to WT cells (Table 2), suggesting the cells are undergoing an oxidative stress response. This hypothesis is supported by a relative increase in reactive oxygen species in WT and ΔPor-1 strains, as detected with 2′,7′ dichlorofluorescein (Table 2). *N. crassa* expresses multiple superoxide dismutases (SOD): a Cu/Zn SOD (Sod-1; NCU02133) in the cytoplasm, a Mn-containing mitochondrial enzyme, Sod-2 (NCU01213), a dual-localized enzyme (Sod-3, NCU09560) in mitochondria and the cytosol, and an Fe-containing SOD (NCU07386). Sod-3 was present in increased amounts in the S100 fraction, while the levels of the other three SODs were similar in both strains (Tables S3 and S4).

**Figure 3:**
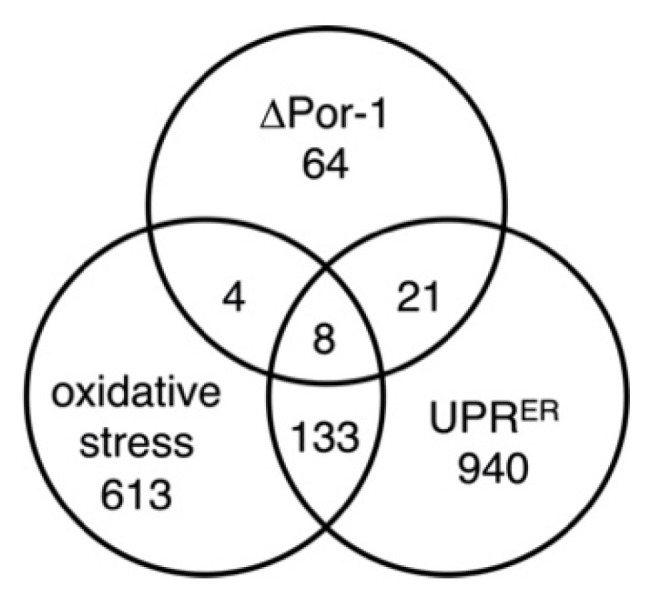
Proteins overexpressed under multiple conditions. The numbers in the overlapping circles indicate the number of proteins or RNA overexpressed in each group of conditions. Eight proteins were more abundant in all three conditions (glutathione S-transferase-1, clock-controlled gene-9, catalase-2, O-methyltransferase family 3, glycine, casein kinase I isoform delta, and hypothetical protein NCU04930). Proteins shared by two or more groups are listed in Table 3. Oxidative stress data were attained from [53] and UPRER data from [29].

To further investigate the possible oxidative stress response, the group of more abundant proteins in ΔPor-1 was compared to those predicted to be upregulated based on the transcriptome analysis of *N. crassa* treated with menadione, an inducer of oxidative stress [53]. About 15% of the more abundant proteins in ΔPor-1 were predicted to be overexpressed in menadione-treated cells; this group includes catalases 2 and 3 and a glutathione S-transferase (Table 3). In terms of more abundant proteins, 12 of those in ΔPor-1 were also more abundant in menadione-treated, oxidatively stressed cells (Figure 3). However, only some of the predicted metabolic changes in the ΔPor-1 cells may reflect an oxidative stress response.

### 3.3. Unfolded Protein Responses

In ΔPor-1, several proteins involved in the mitochondrial misfolded protein response (UPR^mt^) are more abundant than in WT cells [14]. The current study supports these results, as it revealed a relative increase in mitochondrial heat-shock protein 60 (hsp60; Table S6D), which is part of the UPR^mt^ (reviewed in [54]). Levels of Hsp70-5 also increased, but the precise function and cellular location of this isoform have not been confirmed in *N. crassa.*

Protein disulfide isomerase (Pdi-1), a marker for the unfolded protein response of the endoplasmic reticulum (UPR^ER^, [29]), was upregulated in ΔPor-1 (Table S4). In *N. crassa*, UPR^ER^ is triggered when the unfolded protein sensor in the ER membrane, Ire1 (NCU02202), detects unfolded proteins in the lumen. This activates the RNAse domain of the protein, initiating splicing of HAC1 (NCU01856) mRNA to generate the open reading frame for the Hac1 transcription factor (see [28]). Ire1 and Hac1 were not detected in the S100, cytosol-enriched samples. The RNA ligase, TRL1/RLG1 (NCU04410), which completes the splicing process, was found in similar amounts in the S100 fraction of ΔPor-1 and WT cells (Table S4). In further support of a UPR^ER^ response in ΔPor-1, it was noted that 29 of the 97 proteins present in high relative amounts in ΔPor-1 were also predicted from RNA levels to be upregulated in *N. crassa* under one or more UPR^ER^-inducing conditions (presence of dithiothreitol (DTT) or Tunicamycin (TM); [29] (Table 3; Figure 3). Furthermore, two proteins involved in endoplasmic reticulum-associated degradation (ERAD) pathways (Hsp-40 and Pcb-1) were detected only in the ΔPor-1 strain (Table S4), suggesting that they are overexpressed. Therefore, we predicted that ΔPor-1 would be more sensitive than WT to additional drug-induced misfolding of proteins in the ER, due to the presence of misfolded proteins associated with the lack of VDAC. Cells were treated with tunicamycin, which blocks the glycosylation of proteins in the ER, thereby inducing their misfolding [28]. The concentration used was sufficient to induce mild levels of stress in WT cells [28]. Slow growth was observed for WT in the presence of tunicamycin (Table 2), indicating that the cells could survive with the level of misfolded proteins induced by the drug. In contrast, no growth was observed in the ΔPor-1 strain in the presence of tunicamycin, supporting the hypothesis that misfolded proteins are associated with the absence of VDAC and that further increases in their levels are fatal.

Taken together, the overall analysis is suggestive of active UPR^ER^ in ΔPor-1 *N. crassa*, although conclusions drawn from comparisons of these preliminary proteomic analyses and transcriptomic studies need to be considered with caution.

### 3.4. Hyphal Morphology

**Figure 4:**
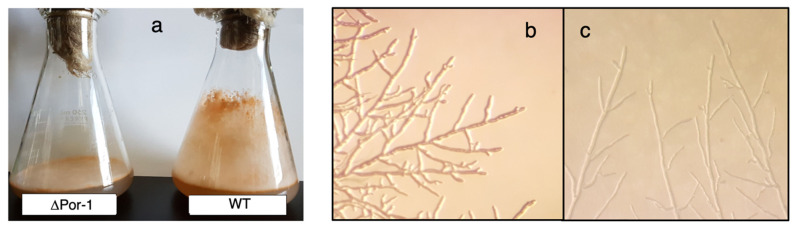
Morphology of ΔPor-1. ΔPor-1 and WT *N. crassa* were grown on solidified VM medium; flasks were incubated in the dark for 3 days and 7 days for WT and ΔPor-1, respectively, and subsequently transferred to the light for 3 days to promote conidiation. In the WT, aerial hyphae were observed as growth on the surface of the flask above the medium, and orange clusters of conidia are seen at the growth front. (**a**) In ΔPor-1, aerial hyphae were not observed, and there was a small amount of conidia at the surface of the medium. Hyphal morphology of ΔPor-1 (**b**) and WT (**c**). Cells were grown on a VM plate for 12 h and 24 h at 30 °C for WT and ΔPor-1, respectively. Plates were observed directly with a Leica Wild M8 stereomicroscope (Wild Heerbrugg, Ottawa, ON, Canada) and the resulting images are presented at 60× magnification.

Several proteins showing differential expression are linked to hyphal morphology. Two regulatory proteins involved in conidiation, Rco-1 (NCU06205), and Nrc-2 (NCU01797) were detected only in ΔPor-1, suggesting that they are upregulated in this mutant (Table S4). Intriguingly, the null mutants of rco-1 (regulator of conidiation-1) [55,56] and ΔPor-1 showed reduced aerial hyphae and conidiation compared to WT (Figure 4), and female sterility [12]. Non-repressible conidiation gene #2 (nrc-2) encodes a serine/threonine kinase required to repress the initiation of conidiation under nutrient-sufficient conditions [57] and therefore, its overexpression is expected to repress conidiation. ΔPor-1 exhibited a very low level of conidiation (Figure 4a), suggesting that the phenotype was impacted most by the increased levels of Nrc-2. ΔPor-1 hyphae (Figure 4b) showed a higher degree of branching than WT hyphae (Figure 4c), but the reason for this phenotype is unknown.

### 3.5. Mitochondrial Membranes

**Figure 5:**
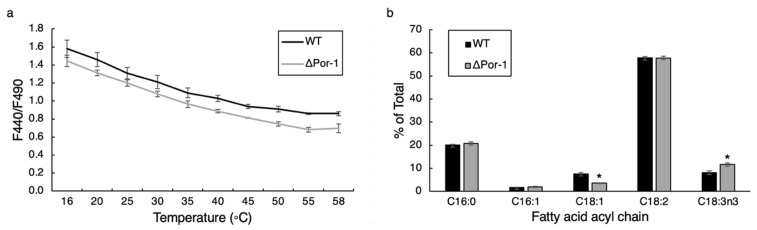
Non-protein components of ΔPor-1 mitochondrial membranes. (**a**) Fluidity of mitochondrial membranes. F440/F490 ratios of Laurdan interacting with WT and ΔPor-1 mitochondria at increasing temperatures. Error bars indicate standard deviation of the samples (*n* = 4). (**b**) Acyl chain analysis of WT and ΔPor-1 mitochondrial phospholipids. The most abundant acyl chains are shown in the graph, and each is represented as the percentage of the total for that strain. * indicates significance at a *p* < 0.05 based on a Student’s *t-*test. (*n* = 3–4). Error bars indicate standard deviation of the samples.

The ΔPor-1 *N. crassa* strain was cold-sensitive and displayed abnormal mitochondrial morphology [12]. These phenotypes likely reflect membrane characteristics in addition to changes in membrane proteomes. As a first step to investigate membranes of ΔPor-1 mitochondria, fluorescence of the probe Laurdan was utilized to assess overall membrane fluidity (Figure 5a). One relative measure of membrane fluidity is the ratio of Laurdan fluorescence at 440 nm to that at 490 nm (F440/F490); a higher ratio is indicative of lower fluidity because the emission of the probe shifts towards the red end of the spectrum (approximately 490 nm) when the membrane is in the fluid phase [36,58,59].

Analysis of whole mitochondrial membranes (Figure 5a) suggests that at 30 °C, the membranes in ΔPor-1 were slightly more fluid than those of the WT, and this trend continued as the membrane sample was heated. Acyl chains of mitochondrial phospholipids and sterol composition were measured, as both can influence the fluidity of the membrane [60,61]. The predominant acyl chain species were very similar in the two strains (Figure 5b), but in the WT strain, the ratio of C18:1 to C18:3n3 acyl chains was higher relative to that in ΔPor-1. The increased fraction of polyunsaturated fatty acids might contribute to increased membrane fluidity in ΔPor-1. Sterol levels also influence membrane fluidity [62]. In ΔPor-1, the amount of ergosterol was lower compared to WT (Table 2), which is expected to increase fluidity [60,63]. Together, these observations suggest a combination of factors affecting physical differences in the lipid environment between ΔPor-1 and WT mitochondria. Membrane fluidity can be influenced by other factors, such as the nature of the polar head groups on the lipids [64]. Detailed analysis of purified inner and outer membranes is required to gain a more complete understanding of the altered membrane properties detected in this study.

## 4. Conclusions

In this study, preliminary proteomic analysis of mitochondrial and S100, cytosol-enriched protein samples provided insights into the mechanisms related to survival of *N. crassa* in the absence of its single isoform of VDAC. In general, proteins involved in oxidative stress responses were more abundant, suggesting increased levels of reactive oxygen species (ROS), as predicted from recent work demonstrating that ΔPor-1 mitochondria have an increased capacity for ROS production [13] and comparisons of whole-cell ROS levels (Table 2). Global comparisons of the preliminary ΔPor-1 proteome with predictions based on transcriptomes studies revealed proteins that were more abundant in ΔPor-1 cells and in WT cells undergoing oxidative stress [53]. Similarly, about 25% of the more abundant proteins detected in ΔPor-1 are also more abundant in cells with activated UPR^ER^, suggesting that part of the response to the absence of VDAC is related to defects in protein folding.

Proteins and enzymes in pathways related to amino acid and protein synthesis were found in relatively low amounts, which is compatible with the slow growth of ΔPor-1. In addition, the acyl chain composition of phospholipids and the abundance of ergosterol were different in ΔPor-1 and WT cells, and this may play a role in altering the physical properties of mitochondrial membranes. Thus, these initial findings indicate that the absence of VDAC induces stress responses and impacts many aspects of cellular structure and function. The mechanism(s) by which substrates and products cross the MOM in ΔPor-1 cells, thereby allowing their survival, remains an intriguing problem for future study. 

## Figures and Tables

**Table 1:** Summary of ΔPor-1 proteomic studies.

Source of Sample (Method)	Proteins Detected	Proteins More Abundant in ΔPor-1 ^2^	Proteins Less Abundant ΔPor-1 ^2^	Reference
S100 cytosol-enriched (1D LC-MS/MS ^1^)	1031	74	96	This work
Mitochondria-enriched (2D LC-MS/MS)	867 ^3^	23	37	This work
Mitochondria-enriched (1D LC-MS/MS)	542	10	13	[14]
Mitochondria-enriched (iTRAQ)	489	12	7	[12]

^1^ D, dimensional; LC, liquid chromatography; MS, mass spectroscopy; ^2^ *p* < 0.05; ^3^ mitochondrial proteins were identified using the method described in [14].

**Table 2:** Characteristics of ΔPor-1 strains.

Measurement	WT	ΔPor-1
^1^ Catalase (cytoplasm, U/mg)	45.8 +/− 3.3	95.5 +/− 21.9
^2^ Intracellular ROS relative to WT (%)	100	150 +/− 50
^3^ Growth rate in the absence of tunicamycin or with 2.5 μg/mL tunicamycin (cm/day)	11.7 +/− 0.1 2.7 +/− 0.8	4.1 +/− 0.7 no growth
^4^ Ergosterol content (% relative to WT)	100 +/− 16.8	59.6 +/− 9.4

^1^ Catalase activity was measured as described in Materials and Methods, and values were normalized to the mass of cytoplasmic protein in the assay. One unit (U) of catalase decomposes 1 µmole of H_2_O_2_ in 1 min in a 60 mM of H_2_O_2_ solution at pH 7.0. Average and standard deviations (*n* = 3 or 4) are presented. WT and ΔPor-1 values were different *p* < 0.05 based on a Student’s *t* test. ^2^ The ratio of fluorescence of 2′,7′ dichlorofluorescein at 524 nm was used to compare intracellular ROS in WT and ΔPor-1 strains. Fluorescence intensity was normalized to the mass of cellular protein in the sample, and the value for WT was set to 100%. The results represent the ratios of normalized fluorescence in ΔPor-1 to WT for 4 pairs of biological replicates, with 2–3 technical replicates per sample. *p* < 0.1 ^3^ Linear growth was measured in “race tubes” containing solid Vogel’s medium containing 2 µg/mL of tunicamycin. Following inoculation of one end of the tube with conidia, linear growth was measured and used to calculate rate in cm/day. WT data from [12]. ^4^ Ergosterol content was measured as described in Materials and Methods, and the WT value was set to 100%.

**Table 3:** More abundant proteins shared among ΔPor-1, oxidatively stressed cells and cells undergoing unfolded protein response in the ER.

A. More Abundant Proteins in ΔPor-1 and Cells Undergoing UPR^ER 1^		B. More Abundant Proteins in ΔPor-1 and Menadione-Treated Cells ^2^	
NCU05780	glutathione S-transferase-1	NCU05780	glutathione S-transferase-1
NCU09519	2,5-diketo-D-gluconic acid reductase A	NCU09559	clock-controlled gene-9
NCU10572	short-chain oxidoreductase	NCU05770	catalase-2
NCU01272	mitochondrial presequence protease	NCU04930	hypothetical protein
NCU02549	processing enhancing protein	NCU00355	catalase-3
NCU09559	clock-controlled gene-9	NCU09674	O-methyltransferase family 3
NCU09560	superoxide dismutase	NCU02812	uridylate kinase
NCU05770	catalase-2	NCU08402	zinc-binding alcohol dehydrogenase
NCU03739	ERP38 protein	NCU02727	glycine cleavage system T protein
NCU04930	hypothetical protein	NCU08004	electron transfer flavoprotein alpha-subunit
NCU01589	heat-shock protein 60	NCU06974	histidinol-phosphatase
NCU09674	O-methyltransferase family 3	NCU00685	casein kinase I isoform delta
NCU03611	chitin synthase-1		
NCU03795	cell division control protein 12		
NCU03949	nitropropane dioxygenase-1	**C. More abundant proteins in ΔPor-1, menadione-treated cells and cells undergoing UPR^ER^**	
NCU05881	DUF500 and UBA/TS-N domain-containing protein	NCU05780	glutathione S-transferase-1
NCU10810	mRNA-splicing protein	NCU09559	clock-controlled gene-9
NCU02727	glycine cleavage system T protein	NCU05770	catalase-2
NCU06738	protein transporter sec-31	NCU04930	hypothetical protein
NCU01166	microcycle blastoconidiation	NCU09674	O-methyltransferase family 3
NCU03596	CRAL/TRIO domain-containing protein	NCU02727	glycine cleavage system T protein
NCU06974	histidinol-phosphatase	NCU06974	histidinol-phosphatase
NCU00864	TIM-barrel enzyme family protein	NCU00685	casein kinase I isoform delta
NCU09223	protein disulfide-isomerase		
NCU10360	hypothetical protein		
NCU05495	clock-controlled gene-16		
NCU01004	phosphatidylserine decarboxylase proenzyme		
NCU00685	casein kinase I isoform delta		
NCU00350	epoxide hydrolase		

^1^ UPR^ER^ data from [29]; ^2^ data from menadione-treated cells from [53].

## Data Availability

Data are available in supplementary files.

## Supplementary Materials

The following are available online at https://www.mdpi.com/article/10.3390/microorganisms10020198/s1, Table S1: MS/MS spectra data and scatter plots of replicates of S100 cytosolic data. Table S2: composition of S100 and mitochondria-enriched fractions. Table S3: mitochondrial proteome data. Table S4: S100 cytosolic proteome data. Table S5: categories of proteins enriched the pool of downregulated proteins in ΔPor-1. Table S6: categories of proteins enriched the pool of upregulated proteins in ΔPor-1.
