# Supplementary material for: Proteomic Shifts Reflecting Oxidative Stress and Reduced Capacity for Protein Synthesis, and Alterations to Mitochondrial Membranes in Neurospora crassa Lacking VDAC
Source: Microorganisms. 2022 Jan 18;10(2):198. doi: 10.3390/microorganisms10020198 (PMC8877502; doi:10.3390/microorganisms10020198)
Supplement: Supplementary file 1 [file microorganisms-10-00198-s001.zip › microorganisms-1509771-supplementary.pdf]

Table S1: MS/MS spectra data and scatter plots of replicates of S100 cytosolic data......................page 2
Table S2: Composition of S100 and mitochondria-enriched fractions...page 3
Table S3: Mitochondrial proteome data...page 26
Table S4: S100 cytosolic proteome data...page 38
Table S5: Categories of proteins enriched the pool of downregulated proteins in ΔPor 1.............page 63
Table S6: Categories of proteins enriched the pool of upregulated proteins in   Por 1..................page 65

Table S1

	Run Spectra	Peptides Quantitated
**Mito 2D**		
Wild-type NCWT2D	326084	123107
Delta Por NCKD2D	226857	180125
**S100 Cytoplasm**		
Wild-type #1 NC1S	58594	36947
Wild-type #2 NC2S	58763	36946
Delta Por-1 #1 NC5S	59635	37184
Delta Por-1 #2 NC6S	60657	38145

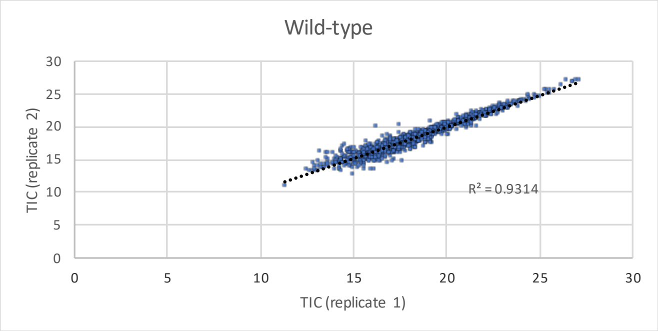

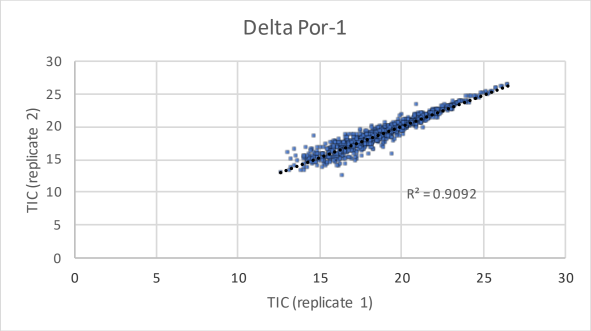

Table S2

S100 proteome

Total	1037
Mitochondrial	232
Ribosomal	58
ER	23
Golgi	12
Lysosomal	0
Plasma membr	7
Cytoplasmic	705

S100 Cytosolic proteins

locus	product
NCU00021	hypothetical protein
NCU00028	S
NCU00040	eukaryotic translation initiation factor 3 1
NCU00043	protein phosphatase-3
NCU00087	pentose phosphate metabolism-1
NCU00102	hypothetical protein
NCU00106	proline-4
NCU00112	aminopeptidase P
NCU00134	exportin-T
NCU00147	ARD/ARD family protein
NCU00150	histidine-7
NCU00173	esterase D
NCU00177	phosphoribosylformylglycinamidine cyclo
NCU00187	menadione-induced gene-11
NCU00194	ornithine transaminase
NCU00200	tryptophan-1
NCU00202	coronin 1
NCU00225	hypothetical protein
NCU00236	flavoprotein oxygenase
NCU00260	oxidoreductase
NCU00261	CTP synthase
NCU00304	hypothetical protein
NCU00326	calcium homeostasis protein Regucalcin
NCU00350	epoxide hydrolase
NCU00364	hypothetical protein
NCU00396	pre-mRNA-splicing factor rse-1
NCU00397	hypothetical protein
NCU00414	adenosine kinase
NCU00422	curved DNA-binding protein
NCU00443	ran-specific GTPase-activating protein 1
NCU00457	translation initiation factor 4B
NCU00463	kynureninase
NCU00472	CDC37
NCU00480	ubiquitin C-terminal hydrolase
NCU00481	a-pheromone processing metallopeptida
NCU00488	protein phosphatase PP2A regulatory sul
NCU00505	small nuclear ribonucleoprotein Sm D1
NCU00518	MGS207 protein
NCU00520	oxidoreductase
NCU00522	cystathionine beta-lyase
NCU00554	homoserine-1
NCU00590	hypothetical protein
NCU00602	siroheme synthase
NCU00629	embden-meyerhof pathway-3
NCU00633	hypothetical protein
NCU00649	nicotinic acid-6
NCU00665	Ser/Thr protein phosphatase

Mitochondrial proteins in S100

locus	product
NCU00021	hypothetical protein
NCU00122	aspartyl aminopeptidase
NCU00295	hypothetical protein
NCU00355	catalase-3
NCU00405	glycyl-tRNA synthetase 1
NCU00461	glutamate dehydrogenase-1
NCU00477	carboxypeptidase Y
NCU00519	ribulose-phosphate 3-epimerase
NCU00537	hypothetical protein
NCU00549	glutathione transferase omega-1
NCU00567	arginine-6
NCU00573	hypothetical protein
NCU00575	glucokinase
NCU00582	cryptochrome
NCU00635	nascent polypeptide-associated complex subunit alpha
NCU00673	serine protease p2
NCU00680	2-methylcitrate dehydratase
NCU00726	cyclosporin-resistant-1
NCU00775	tricarboxylic acid-6
NCU00823	regulatory particle, non-ATPase-like-11
NCU00824	histone deacetylase-3
NCU00936	succinate semialdehyde dehydrogenase
NCU00951	inorganic pyrophosphatase
NCU01021	eukaryotic translation initiation factor 3 subunit EifCf
NCU01175	farnesyl-pyrophosphate synthetase
NCU01219	glutaredoxin
NCU01227	tricarboxylic acid-8
NCU01258	cyanase
NCU01272	mitochondrial presequence protease
NCU01328	transketolase
NCU01422	multiprotein-bridging factor 1
NCU01428	hydroxyisourate hydrolase
NCU01444	rheb small monomeric GTPase RhbA
NCU01528	glyceraldehyde-3-phosphate dehydrogenase-1
NCU01546	coproporphyrinogen III oxidase
NCU01547	regulatory particle, non-ATPase-like-8
NCU01550	adenylate kinase cytosolic
NCU01589	heat shock protein 60
NCU01611	carnitine acetyl transferase
NCU01666	isoleucine-valine-4
NCU01667	arginine-12
NCU01692	citrate synthase
NCU01754	alcohol dehydrogenase-1
NCU01808	cytochrome c-1
NCU01821	alanine-glyoxylate aminotransferase
NCU01955	autophagocytosis protein Aut1
NCU01965	cytochrome-20
NCU01985	cysteine-11
NCU02044	GTP-binding protein
NCU02133	superoxide dismutase-1
NCU02136	transaldolase
NCU02193	cellular filament polypeptide
NCU02224	regulatory particle, non-ATPase-like-3
NCU02273	PEP4 homolog
NCU02328	O-methyltransferase
NCU02366	tricarboxylic acid-3
NCU02391	protein transporter sec-24
NCU02407	dihydrolipoyl dehydrogenase
NCU02438	dihydrolipoamide succinyltransferase
NCU02450	threonine dehydratase
NCU02475	glycine dehydrogenase
NCU02480	short-chain dehydrogenase/reductase
NCU02514	ATPase-1
NCU02533	DNA-directed RNA polymerase I and III polypeptide

Cytosolic proteins continued	
NCU00685	casein kinase I isoform delta
NCU00707	hypothetical protein
NCU00714	heat shock protein STI1
NCU00720	tricarboxylic acid-17
NCU00742	glycerol-3-phosphate dehydrogenase
NCU00743	glycogen debranching enzyme
NCU00768	mRNA binding post-transcriptional regula
NCU00792	branched-chain-amino-acid aminotransf
NCU00793	trehalose phosphate synthase
NCU00838	3-dehydroshikimate dehydratase
NCU00843	adenine-9
NCU00847	hypothetical protein
NCU00864	TIM-barrel enzyme family protein
NCU00867	hypothetical protein
NCU00880	phospholipase A-2-activating protein
NCU00884	NAD dependent epimerase/dehydratase
NCU00892	methionine-6
NCU00903	hypothetical protein
NCU00915	aspartyl-tRNA synthetase
NCU00935	hypothetical protein
NCU00963	methylenetetrahydrofolate dehydrogena
NCU00964	hypothetical protein
NCU00999	hypothetical protein
NCU01083	spermidine-2
NCU01107	short-chain dehydrogenase
NCU01121	hypothetical protein
NCU01157	glutamate-cysteine ligase catalytic subu
NCU01160	ATP-dependent RNA helicase dbp-5
NCU01163	GAF domain nucleotide-binding protein
NCU01166	microcycle blastoconidiation
NCU01177	NAD dependent epimerase/dehydratase
NCU01195	amination-deficient
NCU01197	gulliver-1
NCU01204	tropomyosin
NCU01224	regulatory particle, ATPase-like-2
NCU01233	aldose reductase
NCU01234	eukaryotic initiation factor 4A-12
NCU01240	hypothetical protein
NCU01249	importin alpha subunit
NCU01283	hypothetical protein
NCU01290	centromere/microtubule-binding protein
NCU01300	imidazoleglycerol-phosphate dehydratas
NCU01369	ATP-dependent RNA helicase ded-1
NCU01381	hypothetical protein
NCU01412	proline-3
NCU01419	quinone oxidoreductase
NCU01420	hypothetical protein
NCU01424	DUF636 domain-containing protein
NCU01429	serine-7
NCU01433	phosphoprotein phosphatase-1
NCU01438	nucleosome assembly factor-1
NCU01439	D-3-phosphoglycerate dehydrogenase 1
NCU01443	seryl-tRNA synthetase
NCU01446	uracil phosphoribosyltransferase
NCU01468	translation initiation factor eIF-2B subun
NCU01486	IKI3 family protein
NCU01489	histidine phosphotransferase-1
NCU01523	GTP-binding protein ypt3
NCU01571	hypothetical protein
NCU01587	cofilin
NCU01596	regulatory particle, non-ATPase-like-6
NCU01612	pre-mRNA splicing factor ATP-dependen
NCU01632	aromatic-1 gene cluster
NCU01638	DNA-directed RNA polymerase I subunit
NCU01652	O-acetylhomoserine
NCU01669	putative arginine methyltransferase-3
NCU01701	ribitol kinase

S100 Mitochondrial proteins continued	
NCU02549	processing enhancing protein
NCU02566	alanyl-tRNA synthetase
NCU02580	fumarate reductase Osm1
NCU02634	hypothetical protein
NCU02639	arginine-1
NCU02657	ethionine resistant-1
NCU02677	arginine-3
NCU02701	dipeptidyl peptidase
NCU02726	ethanolamine kinase
NCU02727	glycine cleavage system T protein
NCU02812	uridylate kinase
NCU02887	voltage-gated potassium channel beta-2 subunit
NCU02948	non-anchored cell wall protein-4
NCU02960	peroxin 5
NCU03004	acetate-3
NCU03006	ergosterol-4
NCU03076	delta-1-pyrroline-5-carboxylate dehydrogenase
NCU03125	NIMA-interacting protein TinC
NCU03131	FAD dependent oxidoreductase superfamily
NCU03339	glutathione reductase
NCU03347	nicotinic acid-4
NCU03608	isoleucine + valine-2
NCU03737	elongation factor Tu
NCU03739	ERP38 protein
NCU03804	serine/threonine-protein phosphatase 2B catalytic subunit
NCU03813	formate dehydrogenase
NCU03857	tricarboxylic acid-5
NCU03877	C-1-tetrahydrofolate synthase
NCU03882	hypothetical protein
NCU03904	hypothetical protein
NCU03922	hydroxymethylglutaryl-CoA synthase
NCU03949	nitropropane dioxygenase-1
NCU03982	glucose regulated protein 78
NCU03992	fimbrin
NCU04100	vacuolar sorting protein 1
NCU04202	nucleoside diphosphate kinase-1
NCU04230	acetate utilization-3
NCU04280	aconitate hydratase
NCU04292	branched-chain-amino-acid aminotransferase
NCU04368	glutathione S-transferase Gst3
NCU04370	ubiquitin-activating enzyme E1 1
NCU04410	tRNA ligase
NCU04452	menadione-induced gene-3
NCU04569	5-oxoprolinase
NCU04579	dihydroxy-acid dehydratase
NCU04594	4-carboxymuconolactone decarboxylase
NCU04600	protein phosphatase 2C isoform gamma
NCU04635	hypothetical protein
NCU04754	isoleucine-valine-6
NCU04780	VEG136 protein
NCU04815	lactoylglutathione lyase
NCU04899	tricarboxylic acid-15
NCU04907	hypothetical protein
NCU04910	hypothetical protein
NCU05264	pyrimidine 5'-nucleotidase
NCU05270	mitochondrial translation initiation factor IF-2
NCU05288	rab GDP-dissociation inhibitor
NCU05363	26S protease regulatory subunit 8
NCU05400	hypothetical protein
NCU05410	arginine-5
NCU05418	deoxyhypusine synthase-1
NCU05425	tricarboxylic acid-7
NCU05430	ATPase-2
NCU05488	RNA-binding protein Vip1
NCU05495	clock-controlled gene-16
NCU05516	biotin apo-protein ligase
NCU05526	lysine-5

Cytosolic proteins continued	
NCU01704	NADP:D-xylose dehydrogenase
NCU01744	enhancer-2 of am
NCU01756	actin-related protein-3
NCU01759	menadione-induced gene-5
NCU01768	hypothetical protein
NCU01784	pseudouridylate synthase 3
NCU01786	ribose-phosphate pyrophosphokinase II
NCU01793	RNA binding domain-containing protein
NCU01816	allantoicase-l
NCU01820	exportin-1
NCU01824	UDP-galactopyranose mutase
NCU01838	nitrilase
NCU01843	T-complex protein 1 subunit gamma
NCU01855	hypothetical protein
NCU01918	ARP2/3 complex 20 kDa subunit
NCU01919	ubiquitin C-terminal hydrolase
NCU01939	nonsense-mediated mRNA decay protei
NCU01945	IMP-specific 5'-nucleotidase 1
NCU01949	unknown-16
NCU01956	encodes anonymous transcript-2
NCU02003	translation elongation factor-1
NCU02010	leucine-4
NCU02011	importin subunit beta-1
NCU02018	short chain dehydrogenase
NCU02027	acyl-protein thioesterase 1
NCU02055	uridine nucleosidase Urh1
NCU02075	heat shock protein 70-2
NCU02076	eukaryotic translation initiation factor 4E
NCU02082	cft-1
NCU02090	adenine phosphoribosyltransferase
NCU02103	DNA-directed RNA polymerase II largest
NCU02109	UDP-N-acetylglucosamine pyrophosphor
NCU02113	ubiquitin-conjugating enzyme E2 13
NCU02124	dienelactone hydrolase
NCU02207	T-complex protein 1 subunit beta
NCU02208	unknown-10
NCU02226	methylthioribose-1-phosphate isomerase
NCU02252	embden-meyerhof pathway-6
NCU02260	regulatory particle, ATPase-like-3
NCU02274	formate
NCU02284	nucleolar ATPase Kre33
NCU02289	ubiquitin-conjugating enzyme E2
NCU02319	aminopeptidase
NCU02322	mannose-6-phosphate isomerase
NCU02325	guanine-3
NCU02333	arginase-1
NCU02335	short chain dehydrogenase
NCU02357	importin subunit beta-3
NCU02374	regulatory particle, non-ATPase-like-9
NCU02380	threonyl-tRNA synthetase
NCU02385	hypothetical protein
NCU02387	nuclear import and export protein Msn5
NCU02393	mitogen-activated protein kinase-2
NCU02395	uroporphyrinogen-III synthase
NCU02397	isoleucine-valine-5
NCU02399	hypothetical protein
NCU02404	RNP domain-containing protein
NCU02414	translation initiation factor eif-2b epsilon
NCU02428	nucleolar essential protein 1
NCU02430	cystathionine gamma-synthase
NCU02433	ATG4 protein
NCU02435	histone H2B
NCU02459	DUF52 domain-containing protein
NCU02464	neuronal-specific septin-3
NCU02479	glutamine synthetase
NCU02493	proteasome component C1
NCU02505	succinate

S100 Mitochondrial proteins continued	
NCU05542	xanthine phosphoribosyltransferase 1
NCU05642	cysteinyl-tRNA synthetase
NCU05683	dihydroxy-acid dehydratase
NCU05777	ubiquitin carboxyl-terminal hydrolase 14
NCU05832	hypothetical protein
NCU05850	rubredoxin-NA
NCU05881	DUF500 and UBA/TS-N domain-containing protein
NCU05983	3,4-dihydroxy-2-butanone 4-phosphate synthase
NCU06031	rehydrin
NCU06086	regulatory protein suaprga1
NCU06110	thiazole biosynthetic enzyme
NCU06149	ATP-dependent RNA helicase dhh-1
NCU06211	tricarboxylic acid-16
NCU06247	hypothetical protein
NCU06307	multisynthetase complex auxiliary component p43
NCU06336	N2,N2-dimethylguanosine tRNA methyltransferase
NCU06346	hypothetical protein
NCU06410	GTP-binding protein YPT52
NCU06441	D-lactate dehydrogenase 2
NCU06448	enoyl-CoA hydratase
NCU06452	cysteine-17
NCU06482	acetate-2
NCU06549	pyridoxine-2
NCU06550	pyridoxine 1
NCU06556	thioredoxin II
NCU06603	ThiJ/PfpI family protein
NCU06666	inositol
NCU06678	exonuclease Kem1
NCU06687	glycogen synthase-1
NCU06698	glycogenin
NCU06732	leukotriene A-4 hydrolase
NCU06780	tRNA
NCU06875	hypothetical protein
NCU06914	histidyl-tRNA synthetase
NCU06943	SIK1
NCU06970	ribose-phosphate pyrophosphokinase II
NCU07126	hypothetical protein
NCU07153	glutamate carboxypeptidase
NCU07267	blue light-induced-3
NCU07365	proteasome catalytic beta-7
NCU07414	hypothetical protein similar to protein mitochondrial targeting prote
NCU07439	hypothetical protein
NCU07550	triosephosphate isomerase
NCU07590	Ser/Thr protein phosphatase
NCU07608	pentose phosphate metabolism-3
NCU07659	acetate-4
NCU07697	tricarboxylic acid-4
NCU07853	uricase
NCU07941	aspartate aminotransferase
NCU07947	glycolipid transfer protein HET-C2
NCU08002	carnitine acetyl transferase
NCU08004	electron transfer flavoprotein alpha-subunit
NCU08022	pol
NCU08053	hypothetical protein
NCU08070	meiotically up-regulated 182 protein
NCU08183	hypothetical protein
NCU08195	arginyl-tRNA synthetase
NCU08269	pyridoxine-4
NCU08352	cysteine-9
NCU08384	xylose reductase
NCU08390	hypothetical protein
NCU08411	aspartate aminotransferase
NCU08471	tricarboxylic acid-9
NCU08477	ypt-like-1
NCU08568	ADP-ribose pyrophosphatase
NCU08677	hypothetical protein
NCU08693	heat shock protein 70-5

Cytosolic proteins continued

NCU02513	YjeF domain-containing protein
NCU02542	embden-meyerhof pathway-1
NCU02547	hypothetical protein
NCU02560	hypothetical protein
NCU02563	transcription elongation factor S-II
NCU02571	acetyl-CoA acetyltransferase
NCU02572	U5 small nuclear ribonucleoprotein comp
NCU02588	nuclear movement protein nudC
NCU02605	DNA-directed RNA polymerase I/II/III su
NCU02619	tRNA-dihydrouridine synthase 3
NCU02629	adenine-5
NCU02650	regulatory particle, non-ATPase-like-5
NCU02668	cell wall synthesis protein-Penicillium chr
NCU02684	chromatin remodelling factor 4-3
NCU02688	ubiquitin-conjugating enzyme E2Z
NCU02720	hypothetical protein
NCU02765	RNA binding protein
NCU02771	uroporphyrinogen decarboxylase
NCU02781	hypothetical protein
NCU02784	hypothetical protein
NCU02785	aromatic-8
NCU02786	hypothetical protein
NCU02788	ric8-like
NCU02797	UTP-glucose-1-phosphate uridylyltransfe
NCU02798	hypothetical protein
NCU02806	Neurospora fourteen-three-three homolo
NCU02809	hypothetical protein
NCU02810	eukaryotic translation initiation factor 2 g
NCU02813	eukaryotic translation initiation factor 3 s
NCU02815	osmotic-1
NCU02829	Sec53-like
NCU02839	T-complex protein 1
NCU02840	regulatory particle, ATPase-like-1
NCU02888	hypothetical protein
NCU02899	hypothetical protein
NCU02979	AMP deaminase
NCU02982	regulatory particle, non-ATPase-like-10
NCU02998	nicotinate-nucleotide pyrophosphorylase
NCU03009	zuotin
NCU03010	lysine-3
NCU03023	phenol 2-monooxygenase
NCU03050	ARP2/3 complex 34 kDa subunit
NCU03061	translation initiation factor RLI1
NCU03068	pyridoxine-3
NCU03084	inosine-uridine preferring nucleoside hyd
NCU03087	diploid state maintenance protein chpA
NCU03100	pentose phosphate metabolism-2
NCU03108	glutamate carboxypeptidase
NCU03117	inosine-5'-monophosphate dehydrogena
NCU03118	hypothetical protein
NCU03124	casein kinase II subunit alpha
NCU03127	urease Ure
NCU03139	histidine-3
NCU03151	peroxisomal membrane protein
NCU03166	adenine-3A
NCU03194	phosphoribosylaminoimidazole carboxyla
NCU03200	serine/threonine protein kinase-10
NCU03204	iron-sulfur cluster assembly associated p
NCU03234	ubr1-like
NCU03241	FK506-resistant-4
NCU03282	3-hydroxyanthranilate 3,4-dioxygenase
NCU03283	2-keto-4-pentenoate hydratase
NCU03290	dipeptidyl peptidase
NCU03300	Neurospora fourteen-three-three homolo
NCU03304	proteosome catalytic beta-3
NCU03306	hypothetical protein
NCU03350	xanthine dehydrogenase-1

S100 Mitochondrial proteins continued

NCU08791	catalase-1
NCU08909	beta-1,3-glucanosyltransferase
NCU08936	clock-controlled gene-15
NCU08954	hypothetical protein
NCU08973	SNARE protein Ykt6
NCU08991	sulfur control-3
NCU08998	4-aminobutyrate aminotransferase
NCU09004	eukaryotic translation initiation factor 6
NCU09116	aromatic aminotransferase Aro8
NCU09123	calcium/calmodulin-dependent kinase-1
NCU09132	tubulin alpha-1
NCU09141	menadione-induced gene-9
NCU09175	GPI-anchored cell wall beta-1,3-endoglucanase EglC
NCU09182	stress responsive A/B barrel domain-containing protein
NCU09223	protein disulfide-isomerase
NCU09228	aminopeptidase 2
NCU09230	cysteine-16
NCU09285	menadione-induced gene-6
NCU09309	proteasome component PRE2
NCU09331	HMF1
NCU09345	no message in thiamine-1
NCU09347	fructose-2,6-bisphosphatase
NCU09366	proteasome catalytic beta-6
NCU09425	NdvB protein
NCU09450	regulatory particle, non-ATPase-like-2
NCU09463	leucine-6
NCU09536	hypothetical protein
NCU09560	superoxide dismutase
NCU09602	heat shock protein 70-1
NCU09770	acetate utilization-8
NCU10008	tricarboxylic acid-14
NCU10020	methionine synthase
NCU10042	embden-meyerhof pathway-7
NCU10468	arginine-4
**total**	**232**

S100 Ribosomal proteins

NCU01452	40S ribosomal protein S1
NCU03102	40S ribosomal protein S11
NCU06432	40S ribosomal protein S12
NCU03038	40S ribosomal protein S13
NCU08620	40S ribosomal protein S16
NCU00475	40S ribosomal protein S18
NCU07826	40S ribosomal protein S19
NCU06047	40S ribosomal protein S2
NCU06892	40S ribosomal protein S20
NCU06431	40S ribosomal protein S22
NCU07182	40S ribosomal protein S24
NCU00618	40S ribosomal protein S27
NCU05599	40S ribosomal protein S28
NCU02181	40S ribosomal protein S4
NCU09475	40s ribosomal protein s5
NCU08502	40S ribosomal protein S6
NCU08500	40S ribosomal protein S8
NCU00979	60S acidic ribosomal protein P2
NCU08964	60S ribosomal protein L10
NCU00294	60S ribosomal protein L10a
NCU02509	60S ribosomal protein L11
NCU01317	60S ribosomal protein L12
NCU01776	60S ribosomal protein L15
NCU01221	60S ribosomal protein L16
NCU03703	60S ribosomal protein L17
NCU03988	60S ribosomal protein L18
NCU05804	60S ribosomal protein L19
NCU00413	60S ribosomal protein L2

Cytosolic proteins continued	
NCU03358	ketoreductase
NCU03368	hypothetical protein
NCU03370	hypothetical protein
NCU03396	nucleolar protein nop-58
NCU03399	hypothetical protein
NCU03425	threonine synthase
NCU03436	tangerine
NCU03438	Arp2/3 complex subunit
NCU03488	pyrimidine-4
NCU03500	aminotransferase
NCU03539	unknown-24
NCU03548	hypothetical protein
NCU03575	isoleucyl-tRNA synthetase
NCU03596	CRAL/TRIO domain-containing protein
NCU03605	amidohydrolase
NCU03606	replication protein A-1
NCU03632	farnesyltransferase/geranylgeranyltransf
NCU03633	mevalonate kinase
NCU03634	hypothetical protein
NCU03651	NADP-dependent malic enzyme
NCU03690	importin beta-2 subunit
NCU03702	rRNA 2'-O-methyltransferase fibrillarin
NCU03704	ubiquitin carboxyl-terminal hydrolase 2
NCU03717	translation initiation factor 4E
NCU03731	HAD superfamily hydrolase
NCU03748	saccharopine dehydrogenase
NCU03755	flavin dependent monooxygenase
NCU03768	lysophospholipase
NCU03779	dihydroxyacetone kinase-1
NCU03781	CobW domain-containing protein
NCU03786	serine/threonine protein phosphatase 2A
NCU03795	cell division control protein 12
NCU03797	ubiquitin C-terminal hydrolase
NCU03806	cycloheximide resistant-2
NCU03826	elongation factor 1-gamma
NCU03836	O-sialoglycoprotein endopeptidase
NCU03853	peptidyl-prolyl cis-trans isomerase
NCU03859	MYG1 protein
NCU03870	hypothetical protein
NCU03876	eukaryotic translation initiation factor 3 s
NCU03897	RNA binding effector protein Scp160
NCU03905	hypothetical protein
NCU03911	F-actin-capping protein subunit alpha
NCU03935	homoserine dehydrogenase
NCU03936	tRNA methyltransferase
NCU03944	WD repeat containing protein 2
NCU03963	nicotinic acid-7
NCU03970	riboflavin kinase
NCU03972	regulatory particle, non-ATPase-like-7
NCU03973	alanine
NCU03980	T-complex protein 1 subunit epsilon
NCU04016	phosphoglycerate mutase
NCU04020	lysyl-tRNA synthetase
NCU04040	tRNA
NCU04054	Benomyl resistant
NCU04069	3'-phosphoadenosine 5'-phosphatase isoform B
NCU04077	cysteine-2
NCU04086	hydrolase
NCU04087	Aha1 domain-containing protein
NCU04097	ABC transporter
NCU04104	chromosome segregation protein Cse1
NCU04109	glutathione S-transferase-2
NCU04118	aspartokinase
NCU04120	calmodulin A
NCU04129	gamma-tocopherol methyltransferase
NCU04130	acylase ACY 1
NCU04140	FK506 resistant-2

S100 Ribosomal proteins continued	
NCU08389	60S ribosomal protein L20
NCU06661	60S ribosomal protein L22
NCU02905	60S ribosomal protein L23
NCU03150	60S ribosomal protein L24
NCU06226	60S ribosomal protein L25
NCU01827	60S ribosomal protein L27
NCU06843	60S ribosomal protein L3
NCU08963	60S ribosomal protein L30
NCU00464	60S ribosomal protein L32
NCU09109	60S ribosomal protein L33
NCU03635	60S ribosomal protein L38
NCU03757	60S ribosomal protein L4-A
NCU07562	60S ribosomal protein L43
NCU02707	60S ribosomal protein L6
NCU07829	60S ribosomal protein L7
NCU04779	60S ribosomal protein L8
NCU02744	60S ribosomal protein L9
NCU00489	cytoplasmic ribosomal protein-10
NCU00258	cytoplasmic ribosomal protein-15
NCU07830	cytoplasmic ribosomal protein-2
NCU07014	cytoplasmic ribosomal protein-3
NCU04331	cytoplasmic ribosomal protein-4
NCU04552	cytoplasmic ribosomal protein-5
NCU08627	cytoplasmic ribosomal protein-7
NCU00634	ribosomal protein L14
NCU03565	ribosomal protein L26
NCU00971	ribosomal protein S12
NCU01552	ribosomal protein S28
NCU08595	ribosome biogenesis protein
NCU03393	ribosome-associated protein-1
**S100 Golgi**	
NCU01004	phosphatidylserine decarboxylase proenzyme
NCU01100	hypothetical protein
NCU01440	myosin-2
NCU02263	Sec14 cytosolic factor
NCU02510	clathrin heavy chain
NCU04115	clathrin light chain
NCU04511	bud site selection protein 7
NCU05525	DUF833 domain-containing protein
NCU07734	zinc finger protein gcs1
NCU08340	ADP-ribosylation factor 1
NCU08811	arf GTPase-activating protein
**S100 PM**	
NCU02111	myosin-5
NCU03611	chitin synthase-1
NCU04142	heat shock protein 80
NCU08823	band
NCU09469	EF hand domain-containing protein
NCU09842	mitogen activated protein kinase-1
NCU10073	actin binding protein

Cytosolic proteins continued	
NCU04153	pseudouridine synthase
NCU04164	hypothetical protein
NCU04173	actin
NCU04185	protein kinase-1
NCU04187	cap binding protein
NCU04213	progesterone binding protein
NCU04216	adenine-7
NCU04221	trehalase-2
NCU04228	phytanoyl-CoA dioxygenase
NCU04237	phosphopantothenoylcysteine decarboxylase
NCU04238	histone H1-binding protein
NCU04277	threonine-4
NCU04289	hypothetical protein
NCU04303	asparagine synthetase 2
NCU04306	methionine aminopeptidase 2B
NCU04315	dre-2
NCU04317	DUF431 domain-containing protein
NCU04323	dihydroorotase
NCU04337	survival factor 1
NCU04342	hypothetical protein
NCU04344	translation initiation factor eIF-2B alpha subunit
NCU04385	leucine-2
NCU04411	tryptophan-4
NCU04414	regulatory particle, ATPase-like-5
NCU04442	GAL10
NCU04443	quinone oxidoreductase
NCU04448	T-complex protein 1 subunit alpha
NCU04449	prolyl-tRNA synthetase
NCU04460	galactose-1-phosphate uridylyltransferase
NCU04470	HD domain-containing protein
NCU04479	LAP2
NCU04483	sedoheptulose-1,7-bisphosphatase
NCU04510	glycerol dehydrogenase-3
NCU04583	acetyltransferase
NCU04592	3-oxoacyl
NCU04611	transcription elongation factor spt-6
NCU04640	eukaryotic translation initiation factor 2 beta subunit
NCU04642	adenosine deaminase
NCU04647	actin binding protein
NCU04648	glutamine-dependent NA
NCU04676	glutathione S-transferase
NCU04720	nitrate nonutilizer-6
NCU04757	hypothetical protein
NCU04759	nuclear transport factor 2
NCU04791	menadione-induced gene-10
NCU04799	polyadenylate-binding protein
NCU04826	hypothetical protein
NCU04843	hypothetical protein
NCU04856	glutamine-2
NCU04904	hypothetical protein
NCU04909	hypothetical protein
NCU04923	glycerol dehydrogenase-1
NCU04924	hypothetical protein similar to phosphatidyl synthase
NCU04930	hypothetical protein
NCU05041	trehalose-phosphatase
NCU05095	phenylalanyl-tRNA synthetase subunit alpha
NCU05120	DNA-dependent RNA polymerase II RPB140
NCU05129	tryptophan-2
NCU05137	non-anchored cell wall protein-1
NCU05143	rds1
NCU05164	short chain dehydrogenase/reductase SDR
NCU05200	Pro-apoptotic serine protease nma-111
NCU05238	cysteine-4
NCU05252	deoxyhypusine hydroxylase
NCU05269	heat shock protein 88
NCU05274	eukaryotic initiation factor 5A
NCU05290	orotate phosphoribosyltransferase

Cytosolic proteins continued

NCU05291	polyamine acetyltransferase
NCU05292	DNA damage-inducible protein 1
NCU05295	proteasome catalytic alpha-5
NCU05301	methyltransferase
NCU05305	DNA-directed RNA polymerase II polypeptide
NCU05342	hypothetical protein
NCU05387	hydrolase
NCU05420	aromatic-3
NCU05426	WD repeat protein
NCU05429	1,4-alpha-glucan branching enzyme
NCU05485	casein kinase II regulatory beta subunit-1
NCU05498	hypothetical protein
NCU05512	copper resistance protein Crd2
NCU05548	aromatic-6
NCU05554	unknown-25
NCU05594	L-galactose dehydrogenase
NCU05620	proteasome activator subunit 4
NCU05650	karyopherin Kap123
NCU05667	anchored cell wall protein-3
NCU05716	hypothetical protein
NCU05770	catalase-2
NCU05780	glutathione S-transferase-1
NCU05800	zinc knuckle domain-containing protein
NCU05810	cross pathway control-2
NCU05889	eukaryotic translation initiation factor 3 subunit 6
NCU05937	GDP-mannose pyrophosphorylase
NCU05942	proteosome catalytic alpha-3
NCU05974	cell wall glucanosyltransferase Mwg1
NCU05999	CaaX farnesyltransferase beta subunit Ram1
NCU06003	mannose-1-phosphate guanyltransferase
NCU06035	elongation factor 1-beta
NCU06042	FAD dependent oxidoreductase
NCU06052	DnaJ domain-containing protein
NCU06075	acetate-8
NCU06099	hypothetical protein
NCU06101	hypothetical protein
NCU06105	ADP-ribosylation factor-binding protein GGA1
NCU06108	UPF0135 protein
NCU06112	glutamate decarboxylase
NCU06187	adenine-4
NCU06191	glutathione synthetase large subunit
NCU06210	hypothetical protein
NCU06228	hypothetical protein
NCU06232	leucine-1
NCU06251	KH domain RNA-binding protein
NCU06261	uracil phosphoribosyltransferase
NCU06279	eukaryotic translation initiation factor 3
NCU06290	hypothetical protein
NCU06300	guanylate kinase
NCU06301	hypothetical protein
NCU06310	exopolyphosphatase
NCU06311	GMP synthase
NCU06340	TPR repeat protein
NCU06342	phospholipase D
NCU06348	myo-inositol-1-monophosphatase
NCU06355	karyopherin
NCU06360	histidinol-phosphate aminotransferase
NCU06372	ubiquitin hydrolase L3
NCU06397	profilin
NCU06416	thymine dioxygenase
NCU06417	uracil-5-carboxylate decarboxylase
NCU06440	proteosome catalytic alpha-4
NCU06457	asparaginyl-tRNA synthetase
NCU06459	differentiation regulator
NCU06464	hypothetical protein
NCU06471	proline-1
NCU06472	histone acetyl transferase-1

Cytosolic proteins continued

NCU06512	methionine-8
NCU06554	phosphoserine phosphatase
NCU06561	RHO protein GDP dissociation inhibitor-1
NCU06578	KapG
NCU06617	myosin regulatory light chain cdc4
NCU06622	U2 small nuclear ribonucleoprotein A'
NCU06630	protein phosphatase-1
NCU06652	S
NCU06664	translin
NCU06684	RNA binding protein
NCU06693	hypothetical protein
NCU06711	hypothetical protein
NCU06712	proteasome catalytic alpha-6
NCU06722	tryptophan-5
NCU06724	glutamine-1
NCU06726	nitrilase
NCU06727	spermidine-3
NCU06743	hypothetical protein
NCU06764	proteasome catalytic alpha-2
NCU06783	ATP citrate lyase
NCU06785	ATP-citrate synthase subunit 1
NCU06821	CRO1 protein
NCU06836	acetate utilization-5
NCU06844	hypothetical protein
NCU06863	histone H1
NCU06876	adenosine 5'-monophosphoramidase
NCU06915	hypothetical protein
NCU06923	methionine aminopeptidase 1
NCU06940	hypothetical protein
NCU06941	prefoldin subunit 6
NCU06945	hypothetical protein
NCU06974	histidinol-phosphatase
NCU06998	scavenger mRNA decapping enzyme
NCU07001	methionine-5
NCU07008	carotenoid oxygenase-1
NCU07012	hypothetical protein
NCU07024	osmotic sensitive-2
NCU07027	glycogen phosphorylase
NCU07117	ornithine-N5-oxygenase
NCU07127	dienelactone hydrolase
NCU07156	histidine-6
NCU07165	mannose-6-phosphate isomerase
NCU07167	isoflavone reductase
NCU07171	actin-related protein 2
NCU07192	hypothetical protein
NCU07240	aflatoxin B1 aldehyde reductase member 2
NCU07266	diphthine synthase
NCU07273	hypothetical protein
NCU07281	glucose-6-phosphate isomerase
NCU07282	hypothetical protein
NCU07287	hypothetical protein
NCU07307	chain elongation-2
NCU07308	chain elongation-1
NCU07320	phosphatidylinositol transporter
NCU07352	hypothetical protein
NCU07366	glucosamine-fructose-6-phosphate aminotransferase
NCU07367	regulatory particle, ATPase-like-4
NCU07380	eukaryotic translation initiation factor 3
NCU07408	phosphoprotein P0
NCU07409	hypothetical protein
NCU07415	proline iminopeptidase
NCU07420	eIF4A
NCU07422	hypothetical protein
NCU07437	eukaryotic translation initiation factor 1A
NCU07451	methionyl-tRNA synthetase
NCU07458	N-acetylglucosamine-phosphate mutase
NCU07459	protein arginine N-methyltransferase-1

Cytosolic proteins continued

NCU07467	hypothetical protein
NCU07471	F-actin-capping protein subunit beta
NCU07539	hypothetical protein
NCU07542	rad23-like
NCU07567	T-complex protein 1 subunit theta
NCU07574	hypothetical protein
NCU07589	acetyltransferase
NCU07688	rho GTPase activator Rga
NCU07690	methylenetetrahydrofolate reductase 1
NCU07700	colonial temperature-sensitive-3
NCU07719	isopentenyl-diphosphate delta-isomerase
NCU07721	regulatory particle, non-ATPase-like-1
NCU07723	norsolorinic acid reductase
NCU07725	chorismate mutase
NCU07735	Grp1p
NCU07737	salicylate hydroxylase
NCU07738	hypothetical protein
NCU07755	tyrosyl-tRNA synthetase
NCU07774	GTP cyclohydrolase-1
NCU07776	anchored cell wall protein-5
NCU07807	fructose bisphosphate aldolase
NCU07808	hypothetical protein
NCU07814	DUF89 domain-containing protein
NCU07831	eukaryotic translation initiation factor 3
NCU07832	pre-mRNA processing splicing factor 8
NCU07839	ATP-dependent RNA helicase dbp-2
NCU07866	DEAD helicase superfamily protein
NCU07868	hypothetical protein
NCU07874	nuclear and cytoplasmic polyadenylated RNA-binding protein pub1
NCU07887	ribonucleoside-diphosphate reductase small subunit
NCU07914	phosphoglycerate kinase
NCU07922	elongation factor 3
NCU07926	glutaminyl-tRNA synthetase
NCU07929	eukaryotic translation initiation factor 3 subunit 3
NCU07930	cysteine-18
NCU07936	NMDA receptor-regulated protein 1
NCU07962	hypothetical protein
NCU07987	cystathionine beta-lyase
NCU08003	RSC complex subunit
NCU08008	adenylyl cyclase-associated protein
NCU08029	thiamine pyrophosphokinase
NCU08040	NEDD8-activating enzyme E1 regulatory subunit
NCU08044	oxidoreductase
NCU08046	eukaryotic translation initiation factor 3
NCU08094	dihydrofolate reductase
NCU08130	hypothetical protein
NCU08133	carbonic anhydrase 2
NCU08162	arginine-10
NCU08171	anchored cell wall protein-12
NCU08216	cystathionine beta-synthase
NCU08275	aromatic-L-amino-acid decarboxylase
NCU08277	eukaryotic translation initiation factor 2 alpha subunit
NCU08287	pyrimidine-3
NCU08295	RNA-binding La domain-containing protein
NCU08330	hypothetical protein
NCU08332	hexagonal-1
NCU08374	hypothetical protein
NCU08402	zinc-binding alcohol dehydrogenase
NCU08409	tryptophan-3
NCU08423	bromodomain protein-3
NCU08434	5-methyltetrahydropteroyltriglutamate-homocysteine methyltransferase
NCU08436	alpha/beta hydrolase
NCU08485	WD repeat protein
NCU08499	GTPase-activating protein GYP5
NCU08501	E3 ubiquitin-protein ligase TOM1-like protein
NCU08507	zinc finger protein zpr1
NCU08535	acetyl-CoA carboxylase

Cytosolic proteins continued

NCU08550	hypothetical protein
NCU08554	site-specific parvulin-1
NCU08578	oxysterol binding protein
NCU08605	proteosome catalytic beta-2
NCU08666	HAD superfamily hydrolase
NCU08669	betaine aldehyde dehydrogenase 2
NCU08671	phosphomevalonate kinase
NCU08685	phosphoribosylformylglycinamidine synthase
NCU08687	galactokinase
NCU08695	hypothetical protein
NCU08767	serine/threonine protein kinase-52
NCU08824	molybdopterin binding domain-containing protein
NCU08828	fatty acid oxidation-2
NCU08840	chromatin remodeling complex subunit
NCU08859	hypothetical protein
NCU08875	Cullin binding protein CanA
NCU08882	NAD binding Rossmann fold oxidoreductase
NCU08886	amidohydrolase
NCU08888	phenylalanyl-tRNA synthetase subunit beta
NCU08894	glutamyl-tRNA synthetase
NCU08920	ATP-binding cassette sub-family F member 2
NCU08923	zinc knuckle domain-containing protein
NCU08931	merozoite capping protein-1
NCU08935	peroxisomal-coenzyme A synthetase
NCU08944	N-acetyltransferase complex ARD1 subunit
NCU08952	translation regulator GCD7
NCU08955	UBX domain-containing protein
NCU08959	hypothetical protein
NCU08960	hypothetical protein
NCU08997	repressible alkaline phosphatase
NCU09014	cleavage and polyadenylation specificity factor subunit 5
NCU09040	menadione-induced gene-4
NCU09041	L-xylulose reductase
NCU09076	ATP-dependent RNA helicase
NCU09089	hypothetical protein
NCU09090	phosphopantothenate-cysteine ligase
NCU09097	hypothetical protein
NCU09111	glucose-6-phosphate 1-dehydrogenase
NCU09174	hypothetical protein
NCU09194	nuclease S1
NCU09237	KH domain RNA binding protein
NCU09239	proliferating cell nuclear antigen
NCU09264	APAF1-interacting protein
NCU09269	ran-like
NCU09320	histidine-2
NCU09371	6,7-dimethyl-8-ribityllumazine synthase
NCU09377	B-regulatory subunit-1
NCU09442	DUF757 domain-containing protein
NCU09468	tubulin alpha-2
NCU09519	2,5-diketo-D-gluconic acid reductase A
NCU09533	NAD binding Rossmann fold oxidoreductase
NCU09545	methylenetetrahydrofolate reductase 2
NCU09547	U4/U6.U5 tri-snRNP-associated protein 2
NCU09559	clock-controlled gene-9
NCU09572	ARP2/3 complex 21 kDa subunit
NCU09600	dienelactone hydrolase
NCU09646	3-ketoacyl-CoA thiolase
NCU09674	O-methyltransferase family 3
NCU09699	histone acetyltransferase Spt10
NCU09700	T-complex protein 1 subunit beta
NCU09707	hypothetical protein
NCU09709	T-complex protein 1 subunit zeta
NCU09715	alpha,alpha-trehalose-phosphate synthase
NCU09730	kinesin-1
NCU09744	nuclear pore complex subunit
NCU09746	gephyrin
NCU09783	D-isomer specific 2-hydroxyacid dehydrogenase

Cytosolic proteins continued

NCU09789	adenine-8
NCU09794	NAD-binding Rossmann fold oxidoreductase
NCU09798	aryl-alcohol dehydrogenase
NCU09803	thioredoxin
NCU09817	aromatic-7
NCU09821	oxidoreductase
NCU09841	phosphotyrosine protein phosphatase
NCU09861	Ser/Thr protein phosphatase
NCU09873	acetate utilization-6
NCU09896	adenylyl-sulfate kinase
NCU09911	formyltetrahydrofolate deformylase
NCU09995	hypothetical protein
NCU10007	acetate utilization-9
NCU10029	peptide methionine sulfoxide reductase msrA
NCU10051	flavohemoglobin
NCU10058	phosphoglucomutase 2
NCU10061	proteasome catalytic alpha-1
NCU10067	regulatory particle, non-ATPase-like-12
NCU10292	porphobilinogen deaminase
NCU10360	hypothetical protein
NCU10477	ubiquitin-conjugating enzyme E2
NCU10572	short chain oxidoreductase
NCU10810	mRNA splicing protein
NCU10852	exochitinase
NCU11027	zinc metalloprotease
NCU11173	thiamine-phosphate pyrophosphorylase
NCU11186	NUDIX family hydrolase
NCU11195	D-isomer specific 2-hydroxyacid dehydrogenase
NCU11259	hypothetical protein
NCU11288	xaa-Pro dipeptidase
NCU11292	hypothetical protein
NCU11353	D-xylulose kinase
NCU11356	phenazine biosynthesis PhzC/PhzF protein
NCU11357	cell cycle control protein
NCU11365	aminotransferase
NCU11369	BAR domain-containing protein
NCU11370	6-phosphogluconate dehydrogenase 2
NCU11371	COBW domain-containing protein 1
NCU11381	diphosphomevalonate decarboxylase
NCU11395	S
NCU11427	CUE domain-containing protein
NCU12152	hypothetical protein
NCU15835	hypothetical protein
NCU16368	hypothetical protein
NCU16466	nucleic acid-binding protein
NCU16651	dihydrofolate reductase
NCU16822	hypothetical protein
NCU17177	glycylpeptide N-tetradecanoyltransferase

end cytosolic proteins

Mitochondria-enriched proteome
Mitochondrial proteins

locus	product
NCU00160	NADH:ubiquinone oxidoreductase 6.6kD subunit
NCU00712	3-hydroxy-3-methylglutaryl-coenzyme A reductase
NCU00336	U3 small nucleolar RNA-associated protein 10
NCU02704	branched-chain alpha-keto acid dehydrogenase E
NCU01145	hypothetical protein
NCU01501	mitochondrial peptidyl-tRNA hydrolase Pth2
NCU08961	hypothetical protein, variant
NCU04781	NADH:ubiquinone oxidoreductase 9.8 kDa subunit
NCU06740	hypothetical protein
NCU00528	FHA domain-containing protein
NCU09460	NADH:ubiquinone oxidoreductase 20.1kD subunit
NCU00670	NADH:ubiquinone oxidoreductase 9.5kD subunit
NCU01324	hypothetical protein
NCU07489	serine/threonine-protein phosphatase PP-Z
NCU01296	hypothetical protein
NCU06110	thiazole biosynthetic enzyme
NCU09539	40S ribosomal protein S13
NCU09291	FAD dependent sulfhydryl oxidase Erv2
NCU00107	mitochondrial inner membrane protease ATP-23
NCU08397	hypothetical protein
NCU01024	hypothetical protein
NCU01854	hypothetical protein
NCU04653	LCCL domain-containing protein
NCU09864	2-oxoisovalerate dehydrogenase alpha subunit
NCU07062	protein kinase
NCU04907	hypothetical protein
NCU09536	hypothetical protein
NCU08940	ubiquinol-cytochrome c reductase comple
NCU00347	peroxin-26 Pex26-Penicillium chrysogenum
NCU09345	thiamine biosynthesis protein NMT-1, variant 2
NCU02472	NADH:ubiquinone oxidoreductase 20.8kD subunit
NCU05338	hypothetical protein
NCU07988	ATPase family AAA domain-containing protein 1
NCU05009	NADH-quinone oxidoreductase chain I, variant
NCU02936	proline oxidase
NCU02669	signal sequence binding protein
NCU03561	mitochondrial carrier protein
NCU06149	ATP-dependent RNA helicase dhh-1
NCU06542	mitochondrial 30S ribosomal protein S12
NCU00459	hypothetical protein
NCU08699	bli-4 protein
NCU07075	calcium/proton exchanger, variant
NCU02635	mannan polymerase complexes MNN9 subunit
NCU01754	alcohol dehydrogenase I
NCU06332	alpha/beta hydrolase
NCU01633	hexose transporter HXT13
NCU01467	NADH:ubiquinone oxidoreductase 10.4kD subunit
NCU06943	SIK1
NCU02564	cysteine synthase 2
NCU01360	NADH:ubiquinone oxidoreductase 11.5kD subunit
NCU06410	GTP-binding protein YPT52
NCU02202	serine/threonine kinase IREI
NCU05564	peroxisomal membrane protein PEX31, variant 2
NCU09299	NADH:ubiquinone oxidoreductase 14kD subunit
NCU05513	hypothetical protein
NCU08957	hypothetical protein
NCU09808	dynamin-1
NCU01142	NADH:ubiquinone oxidoreductase 13.4kD subunit
NCU03092	nuclear localization sequence binding protein
NCU10311	mitochondrial ribosomal protein subunit S4
NCU06317	stress response RCI peptide
NCU08939	mannan polymerase II complex ANP1 subunit
NCU00216	NADH-cytochrome b5 reductase 1

Mitochondria-enriched proteome.
Non-mitochondrial proteins

locus	product
NCU16742	hypothetical protein
NCU16385	glutaredoxin domain-containing protein
NCU12046	gamma-butyrobetaine dioxygenase
NCU11258	LYR family protein
NCU10225	hypothetical protein
NCU09886	hypothetical protein
NCU09877	hypothetical protein
NCU09596	phytanoyl-CoA dioxygenase
NCU09517	hypothetical protein
NCU09308	glycoprotease
NCU09222	hypothetical protein
NCU09129	hypothetical protein
NCU09057	hypothetical protein
NCU08954	hypothetical protein
NCU08712	hypothetical protein
NCU08661	pantoate-beta-alanine ligase
NCU08568	ADP-ribose pyrophosphatase
NCU08166	hypothetical protein
NCU07925	LRP16
NCU07871	hypothetical protein
NCU07858	DUF498 domain-containing protein
NCU07608	ribose 5-phosphate isomerase A, variant
NCU07552	hypothetical protein
NCU07423	hypothetical protein
NCU07298	CAIB/BAIF family enzyme
NCU07267	bli-3
NCU07153	glutamate carboxypeptidase
NCU06780	tRNA
NCU06732	leukotriene A-4 hydrolase
NCU06391	hypothetical protein
NCU06336	N2,N2-dimethylguanosine tRNA methyltransfera
NCU06309	hypothetical protein
NCU06201	cell wall biogenesis protein Ecm1
NCU06069	hypothetical protein
NCU05865	hypothetical protein
NCU05838	hypothetical protein
NCU05695	hypothetical protein
NCU05652	hypothetical protein
NCU05642	cysteinyl-tRNA synthetase
NCU05541	hypothetical protein
NCU05427	ATP-dependent Clp protease, variant
NCU05279	hypothetical protein
NCU05148	hypothetical protein
NCU04815	lactoylglutathione lyase
NCU04594	4-carboxymuconolactone decarboxylase
NCU04588	hypothetical protein
NCU04577	hypothetical protein
NCU04368	glutathione S-transferase Gst3
NCU04354	DEAD box family helicase
NCU04212	hypothetical protein
NCU04113	hypothetical protein
NCU04064	hypothetical protein
NCU03817	FMI1 protein
NCU03296	F1 ATPase assembly protein 11
NCU03155	hypothetical protein
NCU03131	FAD dependent oxidoreductase superfamil
NCU03020	IdgA domain-containing protein
NCU02906	NAD-dependent malic enzyme 1
NCU02701	dipeptidyl peptidase
NCU02389	hypothetical protein
NCU02128	D-arabinitol dehydrogenase
NCU02108	BolA domain-containing protein
NCU02095	hypothetical protein

Mitochondria-enriched proteome - mito proteins cont'd	
NCU04645	DUF124 domain-containing protein
NCU06189	5-aminolevulinate synthase
NCU03953	mitochondrial NADH-ubiquinone oxidoreductase 2
NCU03602	hypothetical protein
NCU06430	capsule-associated protein CAP1
NCU01070	hypothetical protein
NCU09816	cytochrome-26
NCU04276	hypothetical protein
NCU00352	phospholipid-transporting ATPase
NCU00249	hypothetical protein
NCU00930	mitochondrial import inner membrane translocas
NCU02074	endoplasmic oxidoreductin-1
NCU00519	ribulose-phosphate 3-epimerase
NCU09002	NADH:ubiquinone oxidoreductase 10.6kD subunit
NCU03827	40S ribosomal protein S9
NCU08947	ubiquinol-cytochrome-c reductase chain VIII
NCU03913	2-oxoisovalerate dehydrogenase beta subunit
NCU01422	multiprotein-bridging factor 1
NCU06741	cytochrome c oxidase subunit VIb
NCU05777	ubiquitin carboxyl-terminal hydrolase 14
NCU06678	exonuclease Kem1
NCU05689	cytochrome c oxidase polypeptide IV
NCU00418	NADH:ubiquinone oxidoreductase 14.8kD subunit
NCU01371	UPF0660 protein
NCU08269	pyridoxamine 5'-phosphate oxidase
NCU08930	NADH:ubiquinone oxidoreductase 21.3kD subunit A
NCU02391	protein transporter sec-24
NCU06695	cytochrome c oxidase polypeptide VI, variant
NCU03798	mitochondrial import inner membrane translocas
NCU08561	succinate/fumarate mitochondrial transporter
NCU08948	NIF domain-containing protein
NCU01101	mitochondrial import protein 1
NCU02373	NADH-ubiquinone oxidoreductase 40 kDa subunit
NCU06606	ubiquinol-cytochrome c reductase iron-sulfu
NCU05220	ATP synthase subunit F
NCU00101	pbn-1
NCU09143	hypothetical protein
NCU07495	sphingolipid long chain base-responsive protei
NCU04814	DUF21 and CBS domain-containing protein
NCU04605	hypothetical protein
NCU01401	mitochondrial outer membrane protein, variant
NCU08358	hypothetical protein
NCU05457	cytochrome c oxidase subunit IV
NCU08066	amino acid transporter
NCU05890	hypothetical protein
NCU00227	mitochondrial cation transporter
NCU05266	KH domain-containing protein
NCU03093	NADH:ubiquinone oxidoreductase 12.3kD subunit
NCU03214	ER lumen protein retaining receptor
NCU03559	ubiquinol-cytochrome c reductase complex cor
NCU09175	GPI-anchored cell wall beta-1,3-endoglucanas
NCU04174	hypothetical protein
NCU03493	glycine rich protein
NCU08354	hypothetical protein
NCU03661	GTPase-activating protein GYP7
NCU02618	peroxisomal membrane protein
NCU06924	kynurenine 3-monooxygenase
NCU05410	acetylornithine aminotransferase
NCU08541	hypothetical protein
NCU08621	hypothetical protein
NCU08411	aspartate aminotransferase
NCU00502	ATP synthase subunit 4
NCU02972	hypothetical protein
NCU06450	mitochondrial ribosomal protein
NCU05706	glutathione S-transferase
NCU02677	carbamoyl-phosphate synthase large subunit
NCU02280	NADH:ubiquinone oxidoreductase 21.3kD subunit B

Mitochondria-enriched proteome - non-mito proteins cont'd	
NCU01861	short chain dehydrogenase/reductase famil
NCU01767	phophatase 2C family protein
NCU01651	hypothetical protein
NCU01306	hypothetical protein
NCU01218	hypothetical protein
NCU01141	CDP-diacylglycerol-glycerol-3-phosphat
NCU01092	3-oxoacyl-
NCU01015	hypothetical protein
NCU00893	folC protein
NCU00608	AMP-binding enzyme
NCU00584	hypothetical protein
NCU00562	hypothetical protein
NCU00498	molybdenum cofactor biosynthesis protein 1 B
NCU00405	glycyl-tRNA synthetase 1
NCU00378	aldehyde dehydrogenase
NCU00293	NADH pyrophosphatase
NCU00264	hypothetical protein
NCU00232	hypothetical protein
NCU00137	ssDNA binding protein
NCU00122	aspartyl aminopeptidase
NCU00051	hypothetical protein
Hypothetical	41

Mitochondria-enriched proteome - mito proteins cont'd	
NCU01689	mitochondrial DNA replication protein YHM2
NCU01666	acetolactate synthase small subunit
NCU05390	mitochondrial phosphate carrier protein
NCU02475	glycine dehydrogenase
NCU07732	carbamoyl-phosphate synthase small subunit
NCU04783	EH domain binding protein epsin 2
NCU04265	invertase
NCU01761	hypothetical protein
NCU03608	ketol-acid reductoisomerase
NCU06524	protease inhibitor
NCU04245	outer mitochondrial membrane translocase
NCU04421	annexin XIV, variant
NCU00431	mitochondrial import receptor subunit Tom22
NCU03837	Snf1 kinase complex beta-subunit Gal83
NCU00684	endonuclease/Exonuclease/phosphatase
NCU03966	mitochondrial Rho GTPase 1
NCU01241	mitochondrial carrier protein
NCU09123	Ca/CaM-dependent kinase-1, variant 2
NCU00789	DUF221 domain-containing protein
NCU00461	NAD-specific glutamate dehydrogenase
NCU00536	homoserine O-acetyltransferase
NCU00969	NADH:ubiquinone oxidoreductase 17.8kD subunit
NCU05221	NADH:ubiquinone oxidoreductase 21kD subunit
NCU04754	branched-chain-amino-acid aminotransferase
NCU03735	hypothetical protein
NCU01821	alanine-glyoxylate aminotransferase
NCU03129	threonyl-tRNA synthetase
NCU08720	hypothetical protein
NCU04481	mitochondrial ATPase
NCU00411	hypothetical protein
NCU06875	hypothetical protein
NCU03711	GTP-binding protein ypt7
NCU02951	40S ribosomal protein S8
NCU01550	adenylate kinase cytosolic
NCU01485	hypothetical protein
NCU06768	60S ribosomal protein L16
NCU03076	delta-1-pyrroline-5-carboxylate dehydrogenase
NCU06662	mitochondrial carrier protein
NCU01878	vesicle-mediated transporter
NCU07112	hypothetical protein
NCU05454	glycerol-3-phosphate dehydrogenase
NCU04412	hypothetical protein
NCU00567	Arg-6 protein, variant
NCU00436	GTPase FZO1
NCU17017	mitochondrial inner membrane protease subunit 1
NCU07756	succinate dehydrogenase cytochrome b560 subunit
NCU07884	mitochondrial oxaloacetate transporter
NCU05027	hypothetical protein
NCU02623	mitochondrial hypoxia responsiv
NCU05299	NADH:ubiquinone oxidoreductase
NCU04579	dihydroxy-acid dehydratase
NCU03556	peroxisomal membrane protein Pmp47
NCU02133	superoxide dismutase
NCU01650	hypothetical protein
NCU00114	30S ribosomal protein S10
NCU01680	plasma membrane ATPase
NCU04899	malate dehydrogenase
NCU01765	NADH:ubiquinone oxidoreductase 78
NCU00050	pyruvate dehydrogenase X component
NCU04946	hypothetical protein
NCU02064	hypothetical protein
NCU01528	glyceraldehyde 3-phosphate-dehydrogenase
NCU10468	arginine biosynthesis argJ
NCU09477	ADP/ATP carrier protein, variant
NCU02544	ABC transporter
NCU09223	protein disulfide-isomerase
NCU04569	5-oxoprolinase

Mitochondria-enriched proteome - mito proteins cont'd

NCU04138	hypothetical protein
NCU03230	mitochondrial ribosomal protein subunit S18
NCU10048	3-methyl-2-oxobutanoat
NCU07879	mitochondrial metal transporter 2
NCU07384	mitochondrial thiamine pyrophosphate carrier 1
NCU02295	phosphatidylinositol-4-phosphate 5-kinase its3
NCU01747	glycerophosphocholine phosphodiesterase Gde1
NCU04636	cysteine desulfurase
NCU00573	hypothetical protein
NCU00430	Na
NCU09119	ATP synthase subunit gamma
NCU03982	glucose-regulated protein
NCU01905	sorbitol dehydrogenase
NCU09463	leucyl-tRNA synthetase
NCU02407	dihydrolipoyl dehydrogenase
NCU12023	mito ribosomal protein S2
NCU08272	cytochrome b2
NCU03802	trimethyllysine dioxygenase
NCU02657	s-adenosylmethionine synthetase, variant
NCU00564	mitochondrial 40S ribosomal protein MRP2
NCU00166	isochorismatase domain-containing protein 2A
NCU05633	stomatin family protein
NCU05526	homocitrate synthase, variant 2
NCU04249	hypothetical protein
NCU08980	alternative NADH-dehydrogenase
NCU04230	isocitrate lyase
NCU03813	formate dehydrogenase
NCU02269	mitochondrial carnitine/acylcarnitine carrie
NCU01692	mitochondrial citrate synthase
NCU01606	ATP synthase subunit 5
NCU00894	hypothetical protein
NCU04044	NADH2 dehydrogenase flavoprotein 1
NCU00605	ThiF domain-containing protein
NCU11345	hypothetical protein
NCU02450	threonine dehydratase
NCU01484	rho-type GTPase
NCU03343	37S ribosomal protein S25
NCU08071	ribosomal protein S16
NCU05881	DUF500 and UBA/TS-N domain-containing protein
NCU02482	2-methylcitrate synthase, variant 2
NCU02419	mitochondrial 37S ribosomal protein S17
NCU01859	NADH:ubiquinone oxidoreductase 20.9kD subunit
NCU08949	hypothetical protein
NCU02013	hypothetical protein
NCU01179	outer membrane translocase 40 kDa subunit
NCU05717	mito ribosomal protein S6
NCU03877	C-1-tetrahydrofolate synthase
NCU03112	NADH-cytochrome b5 reductase 2
NCU05226	ABC transporter
NCU04807	universal stress protein famil
NCU02514	mitochondrial ATP synthase alpha subunit
NCU03558	hypothetical protein
NCU08120	mitochondrial ribosomal protein DAP3
NCU01444	rheb small monomeric GTPase RhbA
NCU01021	Eukaryotic translation initiation factor 3 subunit F
NCU09537	rho-GTPase-activating protein 8
NCU08924	acyl-CoA dehydrogenase
NCU09903	elongation factor 3
NCU07560	50S ribosomal subunit L30
NCU05008	acp-1
NCU05430	ATP synthase beta subunit
NCU03753	clock controlled protein CCG-1
NCU10008	fumarate hydratase
NCU07414	DnaJ family protein
NCU06666	inositol-3-phosphate synthase
NCU09228	aminopeptidase 2
NCU04566	protein kinase SNF1

Mitochondria-enriched proteome - mito proteins cont'd

NCU04013	aldehyde dehydrogenase
NCU02451	mitochondrial hypoxia responsiv
NCU02545	hypothetical protein
NCU02504	DUF1640 domain-containing protein
NCU01454	mitochondrial hydrolase
NCU06881	succinyl-CoA:3-ketoacid-coenzyme A transferas
NCU02812	uridylate kinase
NCU06643	oleate-induced peroxisomal protein
NCU08146	hypothetical protein
NCU07280	serine/threonine-protein kinase gad8
NCU05425	2-oxoglutarate dehydrogenase E1 component
NCU04280	aconitate hydratase
NCU02727	glycine cleavage system T protein
NCU05593	mitochondrial outer membrane beta-barrel protei
NCU04837	mitochondrial 2-oxodicarboxylate carrier 1
NCU06482	pyruvate dehydrogenase E1 component alph
NCU05075	mitochondrial tricarboxylate transporter
NCU02585	aspartyl-tRNA synthetase
NCU02337	mitochondrial carrier protein
NCU03116	Ras GTPase activating protein
NCU08861	hypothetical protein
NCU07982	acetolactate synthase
NCU10020	methionine synthase
NCU09885	acyl-CoA dehydrogenase
NCU07253	1,3-beta-glucanosyltransferase gel1
NCU04180	mitochondrial folate carrier protein Flx1
NCU03339	glutathione reductase
NCU09999	hypothetical protein
NCU07659	pyruvate dehydrogenase complex
NCU05029	iron-sulfur clusters transporter atm-1
NCU11311	small zinc finger protein Tim8
NCU08898	homoaconitase
NCU01473	mitochondrial large ribosomal subunit YmL35
NCU16791	mitochondrial inner membrane protease subunit 2
NCU05400	hypothetical protein
NCU03882	hypothetical protein
NCU02549	ubiquinol-cytochrome-c reductase subunit beta
NCU09741	NADPH-cytochrome P450 reductase
NCU07057	hypothetical protein
NCU04379	neuronal calcium sensor 1
NCU09810	succinyl-CoA synthetase subunit alpha
NCU09551	MFS multidrug transporter
NCU09108	hypothetical protein
NCU04380	acyl-CoA synthetase
NCU03653	hypothetical protein
NCU00737	presequence translocated-associated moto
NCU00385	ATP synthase subunit delta
NCU07362	L-lactate ferricytochrome c oxidoreductase
NCU06441	D-lactate dehydrogenase 2
NCU03926	37S ribosomal protein Rsm24
NCU03216	adenylate kinase
NCU02690	mitochondrial integral membrane protein
NCU06469	54S ribosomal protein L12
NCU07941	aspartate aminotransferase
NCU06761	sphingosine-1-phosphate lyase
NCU04803	2-nitropropane dioxygenase famil
NCU02639	argininosuccinate synthase, variant
NCU01219	glutaredoxin
NCU00316	peroxisomal adenine nucleotide transporter 1
NCU07549	mitochondrial ribosomal protein L43
NCU04181	TOM7, variant 2
NCU01227	succinyl-CoA ligase alpha-chain
NCU09770	acetyl-coa hydrolase
NCU08693	hsp70-like protein
NCU07697	isocitrate dehydrogenase subunit 2
NCU04074	NADH:ubiquinone oxidoreductase 30.4 kDa subunit
NCU02757	60S ribosomal protein L2

Mitochondria-enriched proteome - mito proteins cont'd

NCU02534	NADH:ubiquinone oxidoreductase 49kD subunit
NCU01213	superoxide dismutase
NCU06038	ribosomal protein L36
NCU04415	hypothetical protein
NCU02438	dihydrolipoamide succinyltransferase
NCU01589	heat shock protein 60
NCU09534	peroxiredoxin HYR1
NCU01965	valyl-tRNA synthetase
NCU00896	synaptojanin-1
NCU00828	peroxisomal membrane protein
NCU06749	60S ribosomal protein L3
NCU08053	hypothetical protein
NCU06958	mito ribosomal protein S21
NCU04753	NADH:ubiquinone oxidoreductase 11.6kD subunit
NCU04806	hypothetical protein
NCU04502	hypothetical protein
NCU03188	sugar 1,4-lactone oxidase
NCU06451	hypothetical protein
NCU04410	tRNA ligase
NCU00905	N-acylethanolamine amidohydrolase
NCU08471	succinyl-CoA ligase beta-chain
NCU08329	mitochondrial ribosomal protein subunit L23
NCU07310	hypothetical protein
NCU01546	coproporphyrinogen III oxidase
NCU01010	hypothetical protein
NCU00136	Mito Translational Optimization
NCU08356	acetamidase
NCU08336	succinate dehydrogenase flavoprotein subunit
NCU00477	carboxypeptidase Y
NCU04768	electron transfer flavoprotein-ubiquinon
NCU00680	2-methylcitrate dehydratase
NCU00676	F1-ATP synthase assembly protein
NCU01545	autophagy protein 8
NCU03888	DUF500 and SH3 domain-containing protein
NCU10028	bax Inhibitor family protein
NCU06760	pyruvate dehydrogenase kinase
NCU08552	50S ribosomal protein L14
NCU06452	cysteine synthase
NCU02959	hypothetical protein
NCU01975	complex I intermediate-associated protein CIA30
NCU01255	mitochondrial dynamin GTPase
NCU07493	hypothetical protein
NCU07852	ribosomal protein L13
NCU05313	mitochondria fission 1 protein
NCU07733	electron transfer flavoprotein beta-subunit
NCU04336	60S ribosomal protein L19
NCU03695	phosphatidylserine decarboxylase proenzyme 1
NCU03297	cytochrome c peroxidase
NCU03004	pyruvate dehydrogenase E1 component
NCU05248	hypothetical protein
NCU01514	mitochondrial dicarboxylate carrier
NCU09347	fructose-2,6-bisphosphatase
NCU06211	malate dehydrogenase, variant
NCU03857	tricarboxylic acid-5 protein, variant 2
NCU03211	hypothetical protein
NCU01667	ornithine carbamoyltransferase
NCU09598	mitochondrial escape protein 2
NCU08299	60S ribosomal protein L3
NCU03947	ubiquitin ligase
NCU01169	NADH:ubiquinone oxidoreductase 24
NCU03177	sco1
NCU01808	cytochrome c
NCU00644	ATP synthase subunit G
NCU03739	protein disulfide-isomerase tigA
NCU03158	alpha/beta hydrolase
NCU09688	AP-1 complex subunit mu
NCU09017	hypothetical protein

Mitochondria-enriched proteome - mito proteins cont'd

NCU02804	mitochondrial 60S ribosomal protein L25
NCU07724	mitochondrial division protein 1
NCU06880	AhpC/TSA family protein
NCU05552	hypothetical protein
NCU04945	mitochondrial intermembrane space protein Mia40
NCU03229	mitochondrial inner membrane translocase subuni
NCU01800	37S ribosomal protein S5
NCU08877	glycine cleavage system H protein
NCU05515	mitochondrial import inner membrane translocas
NCU00673	serine protease p2
NCU03777	mitochondrial 3-hydroxyisobutyryl-CoA hydrolase
NCU07286	membrane-associated progesterone recepto
NCU03199	ATP synthase subunit H
NCU01102	mitochondrial export translocase Oxa1
NCU00959	succinate dehydrogenase iron-sulfur protein
NCU00591	methylcrotonoyl-CoA carboxylase subunit alpha
NCU09732	acetyl-CoA acetyltransferase
NCU02580	fumarate reductase Osm1
NCU06244	mitochondrial import inner membrane translocas
NCU00775	isocitrate dehydrogenase subunit 1
NCU06518	NADH-cytochrome b5 reductase 2
NCU03922	hydroxymethylglutaryl-CoA synthase
NCU01962	encodes anonymous transcript-5 protein, variant
NCU00895	RAB GTPase Ypt5
NCU05270	Eukaryotic translation initiation factor 5B
NCU04225	60S ribosomal protein L20
NCU00582	cryptochrome DASH
NCU06307	multisynthetase complex auxiliary component p43
NCU00360	NAD dependent epimerase/dehydratase
NCU09366	proteasome component C5
NCU07955	mitochondrion biogenesis protein
NCU08707	iron sulfur assembly protein
NCU07824	MDM10
NCU00056	3-oxoacyl-[acyl-carrier-protein]-synthase
NCU05623	mitochondrial inner membrane translocase subuni
NCU04817	electron transfer protein 1
NCU04100	vacuolar sorting protein 1
NCU00951	inorganic pyrophosphatase
NCU16833	hypothetical protein
NCU03893	short-chain dehydrogenase/reductase SDR
NCU03737	elongation factor Tu
NCU02403	ubiquinone biosynthesis protein COQ9
NCU01564	calcium dependent mitochondrial carrier protein
NCU08477	small GTP-binding protein
NCU08145	coenzyme A transferase
NCU06559	3-hydroxyisobutyrate dehydrogenase
NCU03359	intermembrane space AAA protease IAP-1
NCU02153	hypothetical protein
NCU02070	peroxisomal biogenesis factor 2
NCU08296	mito ribosomal protein S4
NCU01894	hypothetical protein
NCU03992	fimbrin
NCU03173	HAD superfamily hydrolase
NCU06832	kinesin
NCU06247	hypothetical protein
NCU08291	ferrochelatase
NCU04149	mitochondrial GTPase
NCU08946	hypothetical protein
NCU00958	5-azacytidine resistance protein azr1
NCU00153	pyridine nucleotide-disulfide oxidoreductase
NCU09309	proteasome component PRE2
NCU00660	glutamyl-tRNA amidotransferase
NCU04334	chaperonin
NCU03276	bem46 family protein, variant
NCU00725	epsin-3
NCU00371	mitochondrial import inner membrane translocas
NCU00484	NADH:ubiquinone oxidoreductase 18.4kD subunit

Mitochondria-enriched proteome - mito proteins cont'd

NCU01023	50S ribosomal protein L17
NCU08936	clock-controlled gene-15, variant
NCU05202	hypothetical protein
NCU10021	MFS monosaccharide transporter
NCU03217	MRS7 family protein
NCU00075	mitochondrial import inner membrane translocas
NCU08312	Sec1 family superfamily protein
NCU03979	biotin synthase
NCU02954	homoisocitrate dehydrogenase
NCU02548	hypothetical protein
NCU09403	NmrA family protein
NCU09327	protein phosphatase
NCU03006	sterol 24-C-methyltransferase
NCU08352	thioredoxin-disulfide reductase
NCU03156	NUO
NCU02366	aconitase
NCU02155	hypothetical protein
NCU08991	sulfur control-3 protein, variant
NCU07550	triosephosphate isomerase
NCU04015	vacuolar protein sorting-associated protein 27
NCU03516	mitochondrial ribosomal protein subunit L32
NCU02773	hypothetical protein
NCU03637	CaaX prenyl protease Ste24
NCU09331	HMF1
NCU02734	citrate lyase beta subunit
NCU10732	mitochondrial dicarboxylate transporter
NCU07947	glycolipid transfer protein HET-C2
NCU03525	3-oxoacyl-[acyl-carrier-protein]-reductase
NCU02807	hypothetical protein
NCU01474	60S ribosomal protein L4, variant
NCU00537	hypothetical protein
NCU09553	3-hydroxybutyryl CoA dehydrogenase
NCU07853	uricase
NCU00211	mitochondrial ribosomal protein subunit L31
NCU07020	vacuolar protein sorting-associated protei
NCU16821	hypothetical protein
NCU04554	endochitinase 1
NCU04452	12-oxophytodienoate reductase 1
NCU04388	phosphatidylglycerol/phosphatidylinosito
NCU03008	hypothetical protein
NCU07263	carnitine/acyl carnitine carrier
NCU03125	NIMA-interacting protein TinC
NCU03796	pyruvate dehydrogenase kinase
NCU02291	glutaryl-CoA dehydrogenase
NCU08004	electron transfer flavoprotein alpha-subunit
NCU04429	mitochondrial import protein mmp37
NCU08893	60S ribosomal protein L1
NCU07473	glycosyltransferase family 28 domain-containin
NCU05488	RNA-binding protein Vip1
NCU04068	60S ribosomal protein L6
NCU09450	26S proteasome regulatory subunit rpn2
NCU05714	50S ribosomal subunit protein L15
NCU00655	mitochondrial enoyl reductase
NCU06632	outer mitochondrial membrane translocase 20
NCU01258	cyanase, variant
NCU00549	glutathione transferase omega-1
NCU05982	hexaprenyldihydroxybenzoate methyltransferase
NCU04910	hypothetical protein
NCU08382	peroxisomal half ABC transporter
NCU06448	enoyl-CoA hydratase
NCU02943	mitochondrial import inner membrane translocas
NCU05419	hydroxymethylglutaryl-CoA lyase
NCU03667	hypothetical protein
NCU07478	mitochondrial carrier protein
NCU08002	carnitine acetyl transferase
NCU07386	Fe superoxide dismutase
NCU02287	acyl-CoA dehydrogenase

Mitochondria-enriched proteome - mito proteins cont'd

NCU08941	calcium-binding mitochondrial carrier protei
NCU08048	NAD dehydrogenase
NCU07904	short chain dehydrogenase/reductase
NCU03925	toxin biosynthesis protein
NCU07468	NAD dependent epimerase/dehydratase
NCU06914	histidyl-tRNA synthetase
NCU06086	regulatory protein suaprga1
NCU02305	decaprenyl-diphosphate synthase subunit 1
NCU01104	ATP-dependent RNA helicase MSS116
NCU05261	ATP-dependent protease La
NCU01007	mito ribosomal protein S18
NCU07578	peroxisomal adenine nucleotide transporter 1
NCU01985	sulfate adenylyltransferase
NCU01469	hypothetical protein
NCU02960	peroxisomal targeting signal receptor
NCU07682	acetylglutamate synthase
NCU05363	26S protease regulatory subunit 8
NCU02955	elongation factor G 1
NCU06403	hypothetical protein
NCU05971	xaa-Pro dipeptidase
NCU03394	ribosomal protein S15
NCU08692	cya-5
NCU07465	mitochondrial phosphate carrier protein 2
NCU03347	kynurenine-oxoglutarate transaminase 1
NCU01006	complex I intermediate-associated protein CIA84
NCU00726	peptidyl-prolyl cis-trans isomerase
NCU00694	hypothetical protein
NCU09693	hypothetical protein
NCU06424	aminomethyl transferase
NCU04509	HIT domain-containing protein
NCU04370	ubiquitin-activating enzyme E1 1
NCU09602	heat shock protein 70
NCU09594	seryl-tRNA synthetase
NCU09599	hypothetical protein
NCU05686	cell wall glucanase
NCU01272	mitochondrial presequence protease
NCU05813	mitochondrial large ribosomal subunit
NCU00565	lipoic acid synthetase
NCU03357	ubiquitin conjugation factor E4, variant
NCU03094	hypothetical protein
NCU02136	transaldolase
NCU02063	mitochondrial intermediate peptidase
NCU08278	mitochondrial carrier protein
NCU05288	rab GDP-dissociation inhibitor
NCU04287	hypothetical protein
NCU09560	superoxide dismutase
NCU03030	tyrosyl-tRNA synthetase
NCU00599	mito ribosomal protein S22
NCU02123	mitochondrial GTPase
NCU00831	extracellular serine carboxypeptidase, variant
NCU10987	MFS transporter Fmp42
NCU07670	CYT-19 DEAD-box protein
NCU06331	hypothetical protein
NCU00977	aconitate hydratase
NCU06270	mitochondrial-processing peptidase subuni
NCU05302	GTP-binding protein Obg
NCU02846	hypothetical protein
NCU07793	hypothetical protein
NCU05517	mitochondrial genome maintenance protein MGM101
NCU06103	Mito Translation initiation factor
NCU01479	matrix AAA protease MAP-1
NCU01343	TPR repeat protein
NCU00395	mRNA processing protein
NCU00030	mitochondrial nuclease
NCU05828	hypothetical protein
NCU01512	phenylalanyl-tRNA synthetase
NCU08416	DUF1674 domain-containing protein

Mitochondria-enriched proteome - mito proteins cont'd

NCU01175	farnesyl-pyrophosphate synthetase
NCU08791	catalase-1
NCU04008	mitochondrial ATPase
NCU03908	fmp-52
NCU00486	hypothetical protein
NCU05805	serine hydroxymethyltransferase
NCU04578	ATP-dependent Clp protease proteolytic subuni
NCU09754	mitochondrial chaperone Frataxin
NCU06536	hypothetical protein
NCU04809	MFS phospholipid transporter
NCU02224	26S proteasome non-ATPase regulatory subunit 3
NCU01553	para-hydroxybenzoate-polyprenyltransferase Coq2
NCU00952	mitochondrial ribosomal protein L44
NCU09025	glutamyl-tRNA
NCU08909	beta-1,3-glucanosyltransferase
NCU05772	TOM6
NCU08992	hypothetical protein
NCU01568	hypothetical protein
NCU06778	OXA-2
NCU02002	hypothetical protein
NCU00936	succinate semialdehyde dehydrogenase
NCU09132	alpha tubulin
NCU05601	cytochrome c heme lyase
NCU09091	mitochondrial inner membrane magnesiu
NCU09058	enoyl-CoA hydratase
NCU08005	NADPH-adrenodoxin reductase Arh1
NCU06543	acyl-CoA dehydrogenase
NCU00923	topogenesis of outer membrane beta barre
NCU00278	hypothetical protein
NCU02481	mitochondrial 2-methylisocitrate lyase
NCU04292	branched-chain-amino-acid aminotransferase
NCU08126	D-lactate dehydrogenase
NCU06308	DNA-directed RNA polymerase
NCU03372	nonspecific lipid-transfer protein
NCU03231	mitochondrial chaperone BCS1
NCU06371	mitochondrial ATP-dependent RNA helicase Suv3
NCU09266	methylmalonate-semialdehyde dehydrogenase
NCU01657	cytochrome-c oxidase assembly protein
NCU02396	mitochondrial FAD-linked sulfhydryl oxidas
NCU02634	hypothetical protein
NCU03764	microtubule associated protein
NCU05558	3-ketoacyl-CoA thiolase
NCU03989	ADP,ATP carrier protein
NCU02499	DNL zinc finger domain-containing protein
NCU05927	GTP-binding protein GUF1
NCU06031	mitochondrial peroxiredoxin PRX1
NCU02193	pyruvate decarboxylase
NCU01916	mitochondrial carrier protein RIM2
NCU00823	26S proteasome regulatory subunit RPN11
NCU06556	thioredoxin II
NCU06698	glycogenin, variant
NCU06786	50S ribosomal protein L30
NCU05225	mitochondrial NADH dehydrogenase
NCU08410	50S ribosomal protein L24
NCU06587	hypothetical protein
NCU02705	F1F0 ATP synthase assembly protein Atp10
NCU02267	mitochondrial protein Fmp25
NCU08998	4-aminobutyrate aminotransferase
NCU03759	methionine-tRNA synthetase
NCU01810	hypothetical protein
NCU03310	prohibitin-2
NCU00355	catalase-3
NCU06309	hypothetical protein
NCU00904	D-lactate dehydrogenase
NCU05287	50S ribosomal protein L4
NCU06017	thiosulfate sulfurtransferase
NCU04202	nucleoside diphosphate kinase

Mitochondria-enriched proteome - mito proteins cont'd

NCU02802	mitochondrial ornithine carrier protein
NCU06647	enoyl-CoA hydratase/isomerase
NCU06550	pyridoxine 1
NCU06351	phytase
NCU07021	peptide chain release factor 3
NCU03338	hypothetical protein
NCU00918	hypothetical protein
NCU03823	ABC1 protein
NCU00575	glucokinase
NCU02973	mitochondrial carrier protein
NCU03816	hypothetical protein
NCU07802	hypothetical protein
NCU06721	peptide chain release factor 1
NCU03031	succinate dehydrogenase cytochrome b smal
NCU02946	mitochondrial carrier domain-containing protein
NCU00103	hypothetical protein
NCU05535	hypothetical protein
NCU02127	methylcrotonoyl-CoA carboxylase subunit beta
NCU01428	hydroxyisourate hydrolase
NCU05459	mitochondrial AAA ATPase
NCU04171	50S ribosomal protein L5
NCU03957	COQ7
NCU02273	vacuolar protease A
NCU09263	anchored cell wall protein 4
NCU09826	RNase III domain-containing protein
NCU07295	mitochondrial import inner membrane translocas
NCU01328	transketolase
NCU05850	rubredoxin-NAD
NCU02887	voltage-gated potassium channel beta-2 subunit
NCU02630	heat shock protein 78
NCU00466	glutamyl-tRNA synthetase
NCU06549	pyridoxine 2
NCU00183	DUF185 domain-containing protein
NCU02480	short-chain dehydrogenase/reductase
NCU06246	ATP-dependent RNA helicase mrh-4
NCU02530	cytochrome c oxidase copper chaperone Cox17
NCU04600	protein phosphatase 2C isoform gamma
NCU09432	SNARE-dependent exocytosis protein
NCU05196	mitochondrial DnaJ chaperone
NCU02157	COQ4
NCU17277	hypothetical protein
NCU04313	methionyl-tRNA formyltransferase
NCU04098	monothiol glutaredoxin-5
NCU16968	cytochrome c mitochondrial import factor
NCU06546	hypothetical protein
NCU09862	ubiquinone biosynthesis methyltransferase coq5
NCU04764	hypothetical protein
NCU03929	acyl-CoA synthetase
NCU03814	leucyl-tRNA synthetase
NCU08093	hypothetical protein
NCU07794	2Fe-2S iron-sulfur cluster bindin
NCU04137	vacuolar protein sorting-associated protei
NCU16657	tryptophanyl-tRNA synthetase
NCU09285	zinc-containing alcohol dehydrogenase
NCU04736	plasma membrane calcium-transporting ATPase 3
NCU03949	FMN-dependent 2-nitropropane dioxygenase
NCU02948	non-anchored cell wall protein 4
NCU02126	isovaleryl-CoA dehydrogenase
NCU01041	mitochondrial metalloendopeptidase OMA1
NCU08138	cytochrome C1 heme lyase
NCU00635	nascent polypeptide-associated complex subuni
NCU01304	mitochondrial mRNA processing protein PET127
NCU03749	hydroxyacylglutathione hydrolase
NCU02423	mitoferrin-1
NCU07742	poly
NCU01516	mitochondrial co-chaperone GrpE
NCU08418	tripeptidyl-peptidase

Mitochondria-enriched proteome - mito proteins cont'd

NCU09255	acetate non-utilizing protein 9
NCU09447	NAD
NCU05778	iron sulfur cluster assembly protein 1
NCU00734	Ulp1 protease
NCU03340	cytochrome-c oxidase chain VIIc
NCU09243	hypothetical protein
NCU06404	GTP-binding protein
NCU10226	mmf1
NCU01297	hypothetical protein
NCU06603	ThiJ/PfpI family protein
NCU04433	sulfate permease II
NCU07121	modin
NCU00641	cytochrome c oxidase-assembly factor cox-16
NCU08810	mitochondrial import inner membrane translocas
NCU08476	hypothetical protein
NCU00399	cell wall protein PhiA
NCU01955	autophagocytosis protein Aut1
NCU06495	mitochondrial inner membrane organizatio
NCU06346	hypothetical protein
NCU02750	PH domain-containing protein
NCU06926	hypothetical protein
NCU07332	hypothetical protein
NCU01140	NAD
NCU06436	hypothetical protein
NCU08728	mitochondrial ribosomal protein S19
NCU08326	mitochondrial carrier protein LEU5
NCU02179	D-lactate dehydrogenase
NCU02162	SURF-family protein
NCU07546	multidrug resistance protein MDR
NCU05495	CVNH domain-containing protein
NCU07407	MutS2 protein
NCU06141	protoheme IX farnesyltransferase
NCU09182	stress responsive A/B barrel domain-containin
NCU00865	oxalate decarboxylase oxdC
NCU00276	DNA polymerase gamma
NCU02097	short chain dehydrogenase
NCU07481	morphogenesis protein
NCU01914	sorting nexin-3
NCU00465	chaperone dnaJ
NCU06687	glycogen synthase, variant 3
NCU09975	multidrug resistance protein 3
NCU05005	HHE domain-containing protein

	92
non-mito	

Table S3. Analysis of the Neurospora crassa mitochondrial proteome.					
More abundant proteins are highlighted in green, and those in pink are less abundant.					
z-values of < -1.86 or > 1.86 are considered significant (0.05)					
		Total Ion Counts		Difference (log2)	
**Locus**	**Description of protein**	**WT**	**ΔPor-1**	**ΔPor-1- WT**	**z-value**
**Proteins in higher relative abundance in ΔPor-1**					
NCU06043	GPR/FUN34 family protein	18.50	22.22	3.72	4.20
NCU05005	HHE domain-containing protein	21.09	24.28	3.19	3.70
NCU09975	multidrug resistance protein 3	17.65	20.29	2.64	3.18
NCU06687	glycogen synthase, variant 3	20.58	23.21	2.63	3.17
NCU08954	hypothetical protein	21.01	23.20	2.19	2.75
NCU00465	chaperone dnaJ	17.70	19.88	2.18	2.75
NCU01914	sorting nexin-3	17.12	19.28	2.16	2.73
NCU01092	3-oxoacyl-	20.74	22.80	2.06	2.63
NCU04594	4-carboxymuconolactone decarboxylase	17.89	19.71	1.82	2.41
NCU07481	morphogenesis protein	15.58	17.30	1.72	2.31
NCU07851	superoxide dismutase 1 copper chaperone	18.61	20.30	1.69	2.28
NCU02097	short chain dehydrogenase	21.51	23.15	1.64	2.23
NCU00276	DNA polymerase gamma	18.43	20.01	1.58	2.18
NCU00865	oxalate decarboxylase oxdC	21.56	23.13	1.57	2.17
NCU09182	stress responsive A/B barrel domain-containin	17.86	19.35	1.49	2.09
NCU04368	glutathione S-transferase Gst3	21.12	22.61	1.49	2.09
NCU05695	hypothetical protein	18.40	19.87	1.47	2.07
NCU06141	protoheme IX farnesyltransferase	16.95	18.39	1.44	2.05
NCU07407	MutS2 protein	18.08	19.51	1.43	2.04
NCU05495	CVNH domain-containing protein	19.57	21.00	1.43	2.04
NCU07546	multidrug resistance protein MDR	20.20	21.57	1.37	1.98
NCU02162	SURF-family protein	22.22	23.53	1.31	1.92
NCU00893	folC protein	16.69	18.00	1.31	1.92
**Proteins in lower relative abundance in ΔPor-1**					
NCU03561	mitochondrial carrier protein	27.25	24.55	-2.70	-1.87
NCU02669	signal sequence binding protein	20.40	17.69	-2.71	-1.88
NCU02936	proline oxidase	23.76	21.04	-2.72	-1.89
NCU05009	NADH-quinone oxidoreductase chain I, variant	25.07	22.30	-2.77	-1.93
NCU07988	ATPase family AAA domain-containing protein 1	22.62	19.78	-2.84	-2.00
NCU05338	hypothetical protein	26.18	23.31	-2.87	-2.03
NCU02472	NADH:ubiquinone oxidoreductase 20.8kD subunit	26.39	23.51	-2.88	-2.04
NCU09345	thiamine biosynthesis protein NMT-1, variant 2	26.30	23.39	-2.91	-2.07
NCU09536	hypothetical protein	23.59	20.58	-3.01	-2.16
NCU08940	ubiquinol-cytochrome c reductase comple	24.26	21.25	-3.01	-2.16
NCU00347	peroxin-26 Pex26-Penicillium chrysogenum	21.12	18.11	-3.01	-2.16
NCU04907	hypothetical protein	22.28	19.26	-3.02	-2.17
NCU07062	protein kinase	17.15	14.10	-3.05	-2.20
NCU09864	2-oxoisovalerate dehydrogenase alpha subunit	20.09	17.02	-3.07	-2.22
NCU04653	LCCL domain-containing protein	18.37	15.27	-3.10	-2.25
NCU01854	hypothetical protein	21.23	18.10	-3.13	-2.28
NCU01024	hypothetical protein	24.44	21.29	-3.15	-2.29
NCU08397	hypothetical protein	19.41	16.24	-3.17	-2.31
NCU00107	mitochondrial inner membrane protease ATP-23	23.45	20.26	-3.19	-2.33
NCU09291	FAD dependent sulfhydryl oxidase Erv2	20.82	17.61	-3.21	-2.35
NCU09539	40S ribosomal protein S13	23.20	19.97	-3.23	-2.37
NCU06110	thiazole biosynthetic enzyme	25.24	21.98	-3.26	-2.40
NCU01296	hypothetical protein	20.31	17.00	-3.31	-2.45
NCU07489	serine/threonine-protein phosphatase PP-Z	18.99	15.60	-3.39	-2.52
NCU01324	hypothetical protein	19.22	15.79	-3.43	-2.56
NCU00670	NADH:ubiquinone oxidoreductase 9.5kD subunit	24.07	20.62	-3.45	-2.58
NCU09460	NADH:ubiquinone oxidoreductase 20.1kD subunit	23.75	20.29	-3.46	-2.59
NCU00528	FHA domain-containing protein	20.04	16.47	-3.57	-2.69
NCU06740	hypothetical protein	21.02	17.27	-3.75	-2.86
NCU04781	NADH:ubiquinone oxidoreductase 9.8 kDa subunit	23.22	19.13	-4.09	-3.18
NCU08961	hypothetical protein, variant	19.34	15.17	-4.17	-3.26
NCU01501	mitochondrial peptidyl-tRNA hydrolase Pth2	22.49	18.29	-4.20	-3.29
NCU01145	hypothetical protein	18.91	14.44	-4.47	-3.54
NCU02704	branched-chain alpha-keto acid dehydrogenase E	21.85	17.32	-4.53	-3.60
NCU00336	U3 small nucleolar RNA-associated protein 10	22.24	17.55	-4.69	-3.75
NCU00712	3-hydroxy-3-methylglutaryl-coenzyme A reductase	21.93	16.99	-4.94	-3.99
NCU00160	NADH:ubiquinone oxidoreductase 6.6kD subunit	22.26	16.10	-6.16	-5.14
NCU02179	D-lactate dehydrogenase	20.17	21.41	1.24	1.86
NCU08326	mitochondrial carrier protein LEU5	20.87	22.10	1.23	1.85

NCU08568	ADP-ribose pyrophosphatase	19.75	20.95	1.20	1.82
NCU08728	mitochondrial ribosomal protein S19	20.99	22.16	1.17	1.79
NCU06436	hypothetical protein	18.86	19.99	1.13	1.75
NCU07332	hypothetical protein	16.54	17.64	1.10	1.72
NCU01140	NAD	23.29	24.39	1.10	1.72
NCU06926	hypothetical protein	16.78	17.88	1.10	1.72
NCU02750	PH domain-containing protein	17.39	18.48	1.09	1.71
NCU06346	hypothetical protein	18.07	19.15	1.08	1.71
NCU02906	NAD-dependent malic enzyme 1	22.59	23.66	1.07	1.70
NCU06495	mitochondrial inner membrane organizatio	21.85	22.91	1.06	1.69
NCU01955	autophagocytosis protein Aut1	17.09	18.14	1.05	1.68
NCU00399	cell wall protein PhiA	18.01	19.05	1.04	1.67
NCU08476	hypothetical protein	21.69	22.72	1.03	1.66
NCU08810	mitochondrial import inner membrane translocas	20.59	21.61	1.02	1.65
NCU00641	cytochrome c oxidase-assembly factor cox-16	18.78	19.78	1.00	1.63
NCU07121	modin	18.01	18.96	0.95	1.58
NCU04433	sulfate permease II	18.22	19.14	0.92	1.55
NCU06603	ThiJ/PfpI family protein	17.07	17.96	0.89	1.53
NCU10226	mmf1	22.31	23.18	0.87	1.51
NCU01297	hypothetical protein	18.05	18.92	0.87	1.51
NCU09243	hypothetical protein	16.60	17.46	0.86	1.50
NCU06404	GTP-binding protein	18.57	19.43	0.86	1.50
NCU09308	glycoprotease	20.60	21.45	0.85	1.49
NCU03340	cytochrome-c oxidase chain VIIc	20.77	21.61	0.84	1.48
NCU01306	hypothetical protein	21.83	22.66	0.83	1.47
NCU05778	iron sulfur cluster assembly protein 1	23.50	24.31	0.81	1.45
NCU00734	Ulp1 protease	17.92	18.73	0.81	1.45
NCU09447	NAD	16.56	17.36	0.80	1.44
NCU09255	acetate non-utilizing protein 9	17.74	18.53	0.79	1.43
NCU08418	tripeptidyl-peptidase	17.11	17.90	0.79	1.43
NCU01516	mitochondrial co-chaperone GrpE	24.04	24.80	0.76	1.40
NCU07742	poly	18.99	19.73	0.74	1.38
NCU06780	tRNA	17.34	18.08	0.74	1.38
NCU03749	hydroxyacylglutathione hydrolase	23.28	24.02	0.74	1.38
NCU02423	mitoferrin-1	22.32	23.06	0.74	1.38
NCU01304	mitochondrial mRNA processing protein PET127	22.97	23.69	0.72	1.36
NCU02389	hypothetical protein	17.04	17.71	0.67	1.32
NCU00635	nascent polypeptide-associated complex subuni	19.83	20.50	0.67	1.32
NCU08138	cytochrome C1 heme lyase	22.19	22.85	0.66	1.31
NCU04113	hypothetical protein	17.08	17.73	0.65	1.30
NCU01041	mitochondrial metalloendopeptidase OMA1	20.10	20.73	0.63	1.28
NCU02126	isovaleryl-CoA dehydrogenase	20.51	21.12	0.61	1.26
NCU00137	ssDNA binding protein	24.01	24.61	0.60	1.25
NCU02948	non-anchored cell wall protein 4	23.57	24.16	0.59	1.24
NCU03949	FMN-dependent 2-nitropropane dioxygenase	20.16	20.73	0.57	1.22
NCU04736	plasma membrane calcium-transporting ATPase 3	21.33	21.89	0.56	1.21
NCU09285	zinc-containing alcohol dehydrogenase	24.04	24.59	0.55	1.20
NCU16657	tryptophanyl-tRNA synthetase	22.08	22.62	0.54	1.19
NCU01861	short chain dehydrogenase/reductase famil	19.15	19.69	0.54	1.19
NCU07794	2Fe-2S iron-sulfur cluster bindin	20.82	21.36	0.54	1.19
NCU04137	vacuolar protein sorting-associated protei	18.52	19.06	0.54	1.19
NCU16742	hypothetical protein	23.52	24.05	0.53	1.19
NCU08093	hypothetical protein	20.52	21.05	0.53	1.19
NCU03814	leucyl-tRNA synthetase	24.54	25.06	0.52	1.18
NCU03929	acyl-CoA synthetase	22.45	22.96	0.51	1.17
NCU04764	hypothetical protein	22.31	22.81	0.50	1.16
NCU16385	glutaredoxin domain-containing protein	20.80	21.28	0.48	1.14
NCU09862	ubiquinone biosynthesis methyltransferase coq5	24.18	24.66	0.48	1.14
NCU07298	CAIB/BAIF family enzyme	22.78	23.25	0.47	1.13
NCU06546	hypothetical protein	22.62	23.09	0.47	1.13
NCU16968	cytochrome c mitochondrial import factor	22.27	22.73	0.46	1.12
NCU04064	hypothetical protein	17.56	18.02	0.46	1.12
NCU04313	methionyl-tRNA formyltransferase	20.17	20.63	0.46	1.12
NCU04098	monothiol glutaredoxin-5	23.85	24.31	0.46	1.12
NCU17277	hypothetical protein	19.20	19.65	0.45	1.11
NCU05196	mitochondrial DnaJ chaperone	24.58	25.02	0.44	1.10
NCU02157	COQ4	22.93	23.37	0.44	1.10
NCU09432	SNARE-dependent exocytosis protein	18.14	18.58	0.44	1.10
NCU04600	protein phosphatase 2C isoform gamma	19.41	19.83	0.42	1.08
NCU06246	ATP-dependent RNA helicase mrh-4	24.39	24.80	0.41	1.07
NCU02530	cytochrome c oxidase copper chaperone Cox17	20.29	20.70	0.41	1.07
NCU00232	hypothetical protein	18.40	18.81	0.41	1.07
NCU05279	hypothetical protein	20.90	21.30	0.40	1.06
NCU02480	short-chain dehydrogenase/reductase	18.29	18.68	0.39	1.05

NCU05865	hypothetical protein	19.62	20.00	0.38	1.04
NCU00183	DUF185 domain-containing protein	23.27	23.65	0.38	1.04
NCU06549	pyridoxine 2	17.48	17.85	0.37	1.03
NCU04815	lactoylglutathione lyase	20.49	20.86	0.37	1.03
NCU00293	NADH pyrophosphatase	19.69	20.06	0.37	1.03
NCU12046	gamma-butyrobetaine dioxygenase	24.72	25.08	0.36	1.02
NCU00466	glutamyl-tRNA synthetase	24.72	25.08	0.36	1.02
NCU02887	voltage-gated potassium channel beta-2 subunit	22.75	23.10	0.35	1.02
NCU02630	heat shock protein 78	24.38	24.73	0.35	1.02
NCU05850	rubredoxin-NAD	23.80	24.15	0.35	1.02
NCU07295	mitochondrial import inner membrane translocas	23.35	23.69	0.34	1.01
NCU01328	transketolase	22.93	23.27	0.34	1.01
NCU09826	RNase III domain-containing protein	23.39	23.72	0.33	1.00
NCU09263	anchored cell wall protein 4	20.46	20.78	0.32	0.99
NCU03131	FAD dependent oxidoreductase superfamil	17.74	18.06	0.32	0.99
NCU02273	vacuolar protease A	24.65	24.96	0.31	0.98
NCU05459	mitochondrial AAA ATPase	23.14	23.45	0.31	0.98
NCU04171	50S ribosomal protein L5	24.52	24.83	0.31	0.98
NCU03957	COQ7	22.48	22.79	0.31	0.98
NCU02127	methylcrotonoyl-CoA carboxylase subunit beta	21.07	21.37	0.30	0.97
NCU01428	hydroxyisourate hydrolase	16.43	16.73	0.30	0.97
NCU05535	hypothetical protein	18.44	18.74	0.30	0.97
NCU01015	hypothetical protein	19.50	19.79	0.29	0.96
NCU00103	hypothetical protein	24.03	24.32	0.29	0.96
NCU00122	aspartyl aminopeptidase	23.04	23.32	0.28	0.95
NCU03031	succinate dehydrogenase cytochrome b smal	24.11	24.38	0.27	0.94
NCU02946	mitochondrial carrier domain-containing protein	17.61	17.88	0.27	0.94
NCU07802	hypothetical protein	16.85	17.11	0.26	0.93
NCU06721	peptide chain release factor 1	21.35	21.61	0.26	0.93
NCU03816	hypothetical protein	20.73	20.97	0.24	0.91
NCU02973	mitochondrial carrier protein	20.35	20.57	0.22	0.89
NCU00575	glucokinase	20.41	20.62	0.21	0.88
NCU03823	ABC1 protein	23.51	23.72	0.21	0.88
NCU03338	hypothetical protein	23.30	23.50	0.20	0.87
NCU00918	hypothetical protein	16.64	16.84	0.20	0.87
NCU07925	LRP16	19.77	19.96	0.19	0.86
NCU07021	peptide chain release factor 3	24.00	24.19	0.19	0.86
NCU06351	phytase	18.78	18.97	0.19	0.86
NCU01651	hypothetical protein	19.53	19.72	0.19	0.86
NCU06550	pyridoxine 1	22.09	22.26	0.17	0.84
NCU06647	enoyl-CoA hydratase/isomerase	22.28	22.45	0.17	0.84
NCU02128	D-arabinitol dehydrogenase	22.19	22.36	0.17	0.84
NCU02802	mitochondrial ornithine carrier protein	21.69	21.85	0.16	0.84
NCU06017	thiosulfate sulfurtransferase	23.79	23.94	0.15	0.83
NCU04202	nucleoside diphosphate kinase	25.52	25.67	0.15	0.83
NCU05287	50S ribosomal protein L4	23.69	23.84	0.15	0.83
NCU05148	hypothetical protein	18.57	18.72	0.15	0.83
NCU00904	D-lactate dehydrogenase	25.90	26.04	0.14	0.82
NCU06309	hypothetical protein	21.39	21.52	0.13	0.81
NCU05652	hypothetical protein	19.19	19.32	0.13	0.81
NCU03310	prohibitin-2	26.40	26.52	0.12	0.80
NCU00355	catalase-3	20.77	20.89	0.12	0.80
NCU09222	hypothetical protein	21.92	22.04	0.12	0.80
NCU03759	methionine-tRNA synthetase	24.19	24.31	0.12	0.80
NCU01810	hypothetical protein	20.44	20.56	0.12	0.80
NCU08998	4-aminobutyrate aminotransferase	25.84	25.94	0.10	0.78
NCU02267	mitochondrial protein Fmp25	26.03	26.13	0.10	0.78
NCU08410	50S ribosomal protein L24	24.47	24.56	0.09	0.77
NCU06587	hypothetical protein	22.90	22.99	0.09	0.77
NCU02705	F1F0 ATP synthase assembly protein Atp10	24.01	24.10	0.09	0.77
NCU08712	hypothetical protein	18.69	18.77	0.08	0.76
NCU06786	50S ribosomal protein L30	22.91	22.99	0.08	0.76
NCU05225	mitochondrial NADH dehydrogenase	24.32	24.40	0.08	0.76
NCU09596	phytanoyl-CoA dioxygenase	20.36	20.43	0.07	0.75
NCU09057	hypothetical protein	23.97	24.04	0.07	0.75
NCU02095	hypothetical protein	21.97	22.04	0.07	0.75
NCU06698	glycogenin, variant	19.29	19.35	0.06	0.74
NCU06556	thioredoxin II	21.75	21.81	0.06	0.74
NCU07871	hypothetical protein	22.93	22.98	0.05	0.73
NCU06031	mitochondrial peroxiredoxin PRX1	22.09	22.14	0.05	0.73
NCU02193	pyruvate decarboxylase	23.47	23.52	0.05	0.73
NCU01916	mitochondrial carrier protein RIM2	22.16	22.21	0.05	0.73
NCU00823	26S proteasome regulatory subunit RPN11	17.63	17.68	0.05	0.73
NCU08070	meiotically up-regulated 182 protein	19.69	19.74	0.05	0.73

NCU05927	GTP-binding protein GUF1	20.67	20.72	0.05	0.73
NCU03989	ADP,ATP carrier protein	22.16	22.20	0.04	0.72
NCU02499	DNL zinc finger domain-containing protein	21.91	21.95	0.04	0.72
NCU06201	cell wall biogenesis protein Ecm1	20.98	21.01	0.03	0.71
NCU05558	3-ketoacyl-CoA thiolase	24.26	24.28	0.02	0.70
NCU03764	microtubule associated protein	21.72	21.73	0.01	0.69
NCU02634	hypothetical protein	17.42	17.42	0.00	0.68
NCU03817	FMI1 protein	20.29	20.28	-0.01	0.67
NCU02396	mitochondrial FAD-linked sulfhydryl oxidas	20.11	20.10	-0.01	0.67
NCU09266	methylmalonate-semialdehyde dehydrogenase	24.41	24.40	-0.01	0.67
NCU01657	cytochrome-c oxidase assembly protein	22.39	22.38	-0.01	0.67
NCU06371	mitochondrial ATP-dependent RNA helicase Suv3	22.93	22.91	-0.02	0.67
NCU06308	DNA-directed RNA polymerase	22.61	22.58	-0.03	0.66
NCU03372	nonspecific lipid-transfer protein	21.62	21.59	-0.03	0.66
NCU03231	mitochondrial chaperone BCS1	23.18	23.15	-0.03	0.66
NCU08126	D-lactate dehydrogenase	18.66	18.62	-0.04	0.65
NCU04292	branched-chain-amino-acid aminotransferase	16.78	16.74	-0.04	0.65
NCU02481	mitochondrial 2-methylisocitrate lyase	25.22	25.17	-0.05	0.64
NCU06543	acyl-CoA dehydrogenase	23.73	23.68	-0.05	0.64
NCU00923	topogenesis of outer membrane beta barre	21.55	21.50	-0.05	0.64
NCU00278	hypothetical protein	23.52	23.47	-0.05	0.64
NCU09091	mitochondrial inner membrane magnesiu	21.40	21.33	-0.07	0.62
NCU09058	enoyl-CoA hydratase	25.48	25.41	-0.07	0.62
NCU08005	NADPH-adrenodoxin reductase Arh1	24.53	24.46	-0.07	0.62
NCU09132	alpha tubulin	20.31	20.23	-0.08	0.61
NCU05601	cytochrome c heme lyase	24.70	24.62	-0.08	0.61
NCU00936	succinate semialdehyde dehydrogenase	25.37	25.29	-0.08	0.61
NCU06778	OXA-2	22.05	21.96	-0.09	0.60
NCU02002	hypothetical protein	23.46	23.37	-0.09	0.60
NCU08992	hypothetical protein	20.82	20.72	-0.10	0.59
NCU01568	hypothetical protein	19.48	19.38	-0.10	0.59
NCU08909	beta-1,3-glucanosyltransferase	23.67	23.56	-0.11	0.58
NCU05772	TOM6	21.35	21.24	-0.11	0.58
NCU09025	glutamyl-tRNA	22.42	22.30	-0.12	0.57
NCU01553	para-hydroxybenzoate-polyprenyltransferase Coq2	19.70	19.56	-0.14	0.55
NCU00952	mitochondrial ribosomal protein L44	23.44	23.30	-0.14	0.55
NCU09754	mitochondrial chaperone Frataxin	24.20	24.05	-0.15	0.54
NCU07423	hypothetical protein	22.97	22.82	-0.15	0.54
NCU06536	hypothetical protein	25.08	24.93	-0.15	0.54
NCU04809	MFS phospholipid transporter	16.63	16.48	-0.15	0.54
NCU02224	26S proteasome non-ATPase regulatory subunit 3	19.32	19.17	-0.15	0.54
NCU05805	serine hydroxymethyltransferase	25.76	25.61	-0.15	0.54
NCU04578	ATP-dependent Clp protease proteolytic subuni	22.37	22.22	-0.15	0.54
NCU08791	catalase-1	20.86	20.70	-0.16	0.53
NCU04008	mitochondrial ATPase	20.04	19.88	-0.16	0.53
NCU03908	fmp-52	23.10	22.94	-0.16	0.53
NCU02108	BolA domain-containing protein	18.94	18.78	-0.16	0.53
NCU00584	hypothetical protein	23.05	22.89	-0.16	0.53
NCU00486	hypothetical protein	19.14	18.98	-0.16	0.53
NCU08416	DUF1674 domain-containing protein	16.43	16.26	-0.17	0.52
NCU01175	farnesyl-pyrophosphate synthetase	20.15	19.98	-0.17	0.52
NCU05828	hypothetical protein	22.75	22.58	-0.17	0.52
NCU04577	hypothetical protein	20.55	20.38	-0.17	0.52
NCU01767	phophatase 2C family protein	25.21	25.04	-0.17	0.52
NCU01512	phenylalanyl-tRNA synthetase	23.48	23.31	-0.17	0.52
NCU06103	Mito Translation initiation factor	23.40	23.22	-0.18	0.51
NCU01479	matrix AAA protease MAP-1	26.66	26.48	-0.18	0.51
NCU01343	TPR repeat protein	22.93	22.75	-0.18	0.51
NCU00395	mRNA processing protein	24.29	24.11	-0.18	0.51
NCU00030	mitochondrial nuclease	24.27	24.09	-0.18	0.51
NCU07793	hypothetical protein	23.81	23.62	-0.19	0.50
NCU05517	mitochondrial genome maintenance protein MGM101	24.08	23.89	-0.19	0.50
NCU04212	hypothetical protein	24.33	24.14	-0.19	0.50
NCU06270	mitochondrial-processing peptidase subuni	25.52	25.33	-0.19	0.50
NCU05302	GTP-binding protein Obg	22.18	21.99	-0.19	0.50
NCU02846	hypothetical protein	19.84	19.65	-0.19	0.50
NCU10987	MFS transporter Fmp42	18.95	18.75	-0.20	0.50
NCU07670	CYT-19 DEAD-box protein	26.37	26.17	-0.20	0.50
NCU06331	hypothetical protein	16.83	16.63	-0.20	0.50
NCU00977	aconitate hydratase	23.82	23.62	-0.20	0.50
NCU00608	AMP-binding enzyme	19.92	19.72	-0.20	0.50
NCU02123	mitochondrial GTPase	21.31	21.10	-0.21	0.49
NCU00831	extracellular serine carboxypeptidase, variant	23.24	23.03	-0.21	0.49
NCU09560	superoxide dismutase	25.02	24.81	-0.21	0.49

NCU03030	tyrosyl-tRNA synthetase	24.28	24.07	-0.21	0.49
NCU00599	mito ribosomal protein S22	24.07	23.86	-0.21	0.49
NCU08278	mitochondrial carrier protein	20.32	20.10	-0.22	0.48
NCU05288	rab GDP-dissociation inhibitor	21.58	21.36	-0.22	0.48
NCU04287	hypothetical protein	23.07	22.85	-0.22	0.48
NCU06069	hypothetical protein	21.17	20.94	-0.23	0.47
NCU03357	ubiquitin conjugation factor E4, variant	16.00	15.77	-0.23	0.47
NCU03094	hypothetical protein	23.25	23.02	-0.23	0.47
NCU02136	transaldolase	23.36	23.13	-0.23	0.47
NCU02063	mitochondrial intermediate peptidase	23.53	23.30	-0.23	0.47
NCU05813	mitochondrial large ribosomal subunit	24.16	23.92	-0.24	0.46
NCU00565	lipoic acid synthetase	22.81	22.57	-0.24	0.46
NCU01272	mitochondrial presequence protease	27.57	27.33	-0.24	0.46
NCU00498	molybdenum cofactor biosynthesis protein 1 B	21.46	21.22	-0.24	0.46
NCU09599	hypothetical protein	21.97	21.72	-0.25	0.45
NCU05686	cell wall glucanase	18.31	18.06	-0.25	0.45
NCU09602	heat shock protein 70	24.15	23.89	-0.26	0.44
NCU09594	seryl-tRNA synthetase	24.08	23.82	-0.26	0.44
NCU04370	ubiquitin-activating enzyme E1 1	20.07	19.81	-0.26	0.44
NCU09693	hypothetical protein	17.84	17.57	-0.27	0.43
NCU06424	aminomethyl transferase	23.44	23.17	-0.27	0.43
NCU04509	HIT domain-containing protein	20.55	20.28	-0.27	0.43
NCU00694	hypothetical protein	16.10	15.83	-0.27	0.43
NCU07858	DUF498 domain-containing protein	22.20	21.92	-0.28	0.42
NCU08692		20.59	20.31	-0.28	0.42
NCU07465	mitochondrial phosphate carrier protein 2	26.00	25.72	-0.28	0.42
NCU03347	kynurenine-oxoglutarate transaminase 1	22.78	22.50	-0.28	0.42
NCU01006	complex I intermediate-associated protein CIA84	24.73	24.45	-0.28	0.42
NCU00726	peptidyl-prolyl cis-trans isomerase	27.00	26.72	-0.28	0.42
NCU06403	hypothetical protein	24.63	24.34	-0.29	0.41
NCU06391	hypothetical protein	21.59	21.30	-0.29	0.41
NCU05971	xaa-Pro dipeptidase	23.27	22.98	-0.29	0.41
NCU03394	ribosomal protein S15	24.08	23.79	-0.29	0.41
NCU02955	elongation factor G 1	25.62	25.33	-0.29	0.41
NCU07682	acetylglutamate synthase	23.73	23.43	-0.30	0.40
NCU05363	26S protease regulatory subunit 8	18.02	17.72	-0.30	0.40
NCU02960	peroxisomal targeting signal receptor	19.00	18.69	-0.31	0.39
NCU07578	peroxisomal adenine nucleotide transporter 1	18.31	17.99	-0.32	0.38
NCU01985	sulfate adenylyltransferase	21.96	21.64	-0.32	0.38
NCU01469	hypothetical protein	23.09	22.77	-0.32	0.38
NCU01007	mito ribosomal protein S18	23.67	23.34	-0.33	0.37
NCU05261	ATP-dependent protease La	26.49	26.14	-0.35	0.35
NCU02305	decaprenyl-diphosphate synthase subunit 1	23.62	23.27	-0.35	0.35
NCU02044	GTP-binding protein	23.35	23.00	-0.35	0.35
NCU01104	ATP-dependent RNA helicase MSS116	20.34	19.99	-0.35	0.35
NCU06914	histidyl-tRNA synthetase	22.59	22.23	-0.36	0.34
NCU06086	regulatory protein suaprga1	24.34	23.98	-0.36	0.34
NCU07468	NAD dependent epimerase/dehydratase	18.17	17.81	-0.36	0.34
NCU03925	toxin biosynthesis protein	23.54	23.17	-0.37	0.33
NCU08941	calcium-binding mitochondrial carrier protei	21.82	21.45	-0.37	0.33
NCU08048	NAD dehydrogenase	23.95	23.58	-0.37	0.33
NCU07904	short chain dehydrogenase/reductase	23.70	23.33	-0.37	0.33
NCU08002	carnitine acetyl transferase	27.14	26.76	-0.38	0.32
NCU07386	Fe superoxide dismutase	24.43	24.05	-0.38	0.32
NCU03020	IdgA domain-containing protein	23.98	23.60	-0.38	0.32
NCU02287	acyl-CoA dehydrogenase	24.90	24.52	-0.38	0.32
NCU07478	mitochondrial carrier protein	20.21	19.83	-0.38	0.32
NCU05419	hydroxymethylglutaryl-CoA lyase	24.22	23.83	-0.39	0.32
NCU03667	hypothetical protein	16.92	16.53	-0.39	0.32
NCU02566	alanyl-tRNA synthetase	22.88	22.49	-0.39	0.32
NCU08382	peroxisomal half ABC transporter	22.68	22.28	-0.40	0.31
NCU06448	enoyl-CoA hydratase	25.84	25.44	-0.40	0.31
NCU02943	mitochondrial import inner membrane translocas	25.77	25.37	-0.40	0.31
NCU05982	hexaprenyldihydroxybenzoate methyltransferase	22.76	22.36	-0.40	0.31
NCU04910	hypothetical protein	18.44	18.04	-0.40	0.31
NCU06632	outer mitochondrial membrane translocase 20	25.09	24.68	-0.41	0.30
NCU01258	cyanase, variant	19.84	19.43	-0.41	0.30
NCU00549	glutathione transferase omega-1	17.64	17.23	-0.41	0.30
NCU09450	26S proteasome regulatory subunit rpn2	19.31	18.89	-0.42	0.29
NCU05714	50S ribosomal subunit protein L15	24.36	23.94	-0.42	0.29
NCU00655	mitochondrial enoyl reductase	26.02	25.60	-0.42	0.29
NCU08893	60S ribosomal protein L1	24.51	24.09	-0.42	0.29
NCU07473	glycosyltransferase family 28 domain-containin	17.10	16.68	-0.42	0.29
NCU05488	RNA-binding protein Vip1	21.94	21.52	-0.42	0.29

NCU04068	60S ribosomal protein L6	23.80	23.38	-0.42	0.29
NCU08004	electron transfer flavoprotein alpha-subunit	26.14	25.71	-0.43	0.28
NCU04429	mitochondrial import protein mmp37	24.28	23.85	-0.43	0.28
NCU03796	pyruvate dehydrogenase kinase	23.04	22.60	-0.44	0.27
NCU02291	glutaryl-CoA dehydrogenase	24.58	24.14	-0.44	0.27
NCU07263	carnitine/acyl carnitine carrier	23.71	23.27	-0.44	0.27
NCU03125	NIMA-interacting protein TinC	20.07	19.63	-0.44	0.27
NCU16821	hypothetical protein	22.37	21.92	-0.45	0.26
NCU04554	endochitinase 1	18.58	18.13	-0.45	0.26
NCU04452	12-oxophytodienoate reductase 1	18.48	18.03	-0.45	0.26
NCU04388	phosphatidylglycerol/phosphatidylinosito	21.71	21.26	-0.45	0.26
NCU03008	hypothetical protein	20.82	20.37	-0.45	0.26
NCU07020	vacuolar protein sorting-associated protei	19.44	18.99	-0.45	0.26
NCU09553	3-hydroxybutyryl CoA dehydrogenase	24.24	23.78	-0.46	0.25
NCU07853	uricase	19.56	19.10	-0.46	0.25
NCU00211	mitochondrial ribosomal protein subunit L31	23.24	22.78	-0.46	0.25
NCU07153	glutamate carboxypeptidase	22.57	22.11	-0.46	0.25
NCU03525	3-oxoacyl-[acyl-carrier-protein]-reductase	23.03	22.57	-0.46	0.25
NCU02807	hypothetical protein	25.12	24.66	-0.46	0.25
NCU01474	60S ribosomal protein L4, variant	24.18	23.72	-0.46	0.25
NCU00537	hypothetical protein	17.11	16.65	-0.46	0.25
NCU10732	mitochondrial dicarboxylate transporter	23.88	23.41	-0.47	0.24
NCU07947	glycolipid transfer protein HET-C2	20.88	20.41	-0.47	0.24
NCU04354	DEAD box family helicase	20.82	20.35	-0.47	0.24
NCU02734	citrate lyase beta subunit	24.19	23.72	-0.47	0.24
NCU09331	HMF1	18.70	18.22	-0.48	0.23
NCU01218	hypothetical protein	24.10	23.62	-0.48	0.23
NCU00562	hypothetical protein	23.38	22.89	-0.49	0.22
NCU03637	CaaX prenyl protease Ste24	24.26	23.77	-0.49	0.22
NCU08991	sulfur control-3 protein, variant	19.08	18.58	-0.50	0.21
NCU07550	triosephosphate isomerase	23.34	22.84	-0.50	0.21
NCU04015	vacuolar protein sorting-associated protein 27	20.27	19.77	-0.50	0.21
NCU03516	mitochondrial ribosomal protein subunit L32	21.45	20.95	-0.50	0.21
NCU02773	hypothetical protein	16.74	16.24	-0.50	0.21
NCU08352	thioredoxin-disulfide reductase	21.47	20.96	-0.51	0.20
NCU03156	NUO	24.11	23.60	-0.51	0.20
NCU02366	aconitase	29.43	28.92	-0.51	0.20
NCU02155	hypothetical protein	19.68	19.17	-0.51	0.20
NCU09403	NmrA family protein	22.59	22.08	-0.51	0.20
NCU09327	protein phosphatase	23.67	23.16	-0.51	0.20
NCU03006	sterol 24-C-methyltransferase	22.94	22.43	-0.51	0.20
NCU08312	Sec1 family superfamily protein	17.18	16.66	-0.52	0.19
NCU03979	biotin synthase	25.48	24.96	-0.52	0.19
NCU02954	homoisocitrate dehydrogenase	27.06	26.54	-0.52	0.19
NCU02548	hypothetical protein	19.95	19.43	-0.52	0.19
NCU00075	mitochondrial import inner membrane translocas	20.26	19.74	-0.52	0.19
NCU10021	MFS monosaccharide transporter	22.08	21.55	-0.53	0.18
NCU03217	MRS7 family protein	24.40	23.87	-0.53	0.18
NCU08936	clock-controlled gene-15, variant	25.20	24.66	-0.54	0.17
NCU05202	hypothetical protein	22.98	22.44	-0.54	0.17
NCU01023	50S ribosomal protein L17	24.26	23.72	-0.54	0.17
NCU08195	arginyl-tRNA synthetase	23.33	22.78	-0.55	0.16
NCU00484	NADH:ubiquinone oxidoreductase 18.4kD subunit	24.49	23.94	-0.55	0.16
NCU06732	leukotriene A-4 hydrolase	22.00	21.45	-0.55	0.16
NCU04334	chaperonin	25.89	25.34	-0.55	0.16
NCU03276	bem46 family protein, variant	20.50	19.95	-0.55	0.16
NCU00725	epsin-3	17.57	17.02	-0.55	0.16
NCU00371	mitochondrial import inner membrane translocas	23.34	22.79	-0.55	0.16
NCU09309	proteasome component PRE2	18.77	18.21	-0.56	0.15
NCU00660	glutamyl-tRNA amidotransferase	22.38	21.82	-0.56	0.15
NCU08946	hypothetical protein	26.46	25.90	-0.56	0.15
NCU02701	dipeptidyl peptidase	23.14	22.58	-0.56	0.15
NCU00958	5-azacytidine resistance protein azr1	23.01	22.45	-0.56	0.15
NCU00153	pyridine nucleotide-disulfide oxidoreductase	24.10	23.54	-0.56	0.15
NCU08291	ferrochelatase	26.40	25.83	-0.57	0.15
NCU04149	mitochondrial GTPase	19.93	19.36	-0.57	0.15
NCU06832	kinesin	20.02	19.44	-0.58	0.14
NCU06247	hypothetical protein	25.86	25.28	-0.58	0.14
NCU09517	hypothetical protein	23.55	22.97	-0.58	0.14
NCU07267	bli-3	20.78	20.20	-0.58	0.14
NCU03173	HAD superfamily hydrolase	24.17	23.59	-0.58	0.14
NCU03992	fimbrin	20.50	19.91	-0.59	0.13
NCU00405	glycyl-tRNA synthetase 1	23.22	22.63	-0.59	0.13
NCU08296	mito ribosomal protein S4	25.06	24.46	-0.60	0.12

NCU01894	hypothetical protein	24.61	24.01	-0.60	0.12
NCU05642	cysteinyl-tRNA synthetase	22.51	21.91	-0.60	0.12
NCU08477	small GTP-binding protein	24.49	23.88	-0.61	0.11
NCU08145	coenzyme A transferase	21.97	21.36	-0.61	0.11
NCU06559	3-hydroxyisobutyrate dehydrogenase	18.81	18.20	-0.61	0.11
NCU03359	intermembrane space AAA protease IAP-1	24.89	24.28	-0.61	0.11
NCU02153	hypothetical protein	18.13	17.52	-0.61	0.11
NCU02070	peroxisomal biogenesis factor 2	21.81	21.20	-0.61	0.11
NCU00378	aldehyde dehydrogenase	22.72	22.11	-0.61	0.11
NCU04588	hypothetical protein	22.65	22.03	-0.62	0.10
NCU16833	hypothetical protein	23.77	23.15	-0.62	0.10
NCU03893	short-chain dehydrogenase/reductase SDR	26.57	25.95	-0.62	0.10
NCU03737	elongation factor Tu	26.95	26.33	-0.62	0.10
NCU02403	ubiquinone biosynthesis protein COQ9	22.39	21.77	-0.62	0.10
NCU01564	calcium dependent mitochondrial carrier protein	22.80	22.18	-0.62	0.10
NCU05623	mitochondrial inner membrane translocase subuni	22.52	21.89	-0.63	0.09
NCU04817	electron transfer protein 1	24.77	24.14	-0.63	0.09
NCU04100	vacuolar sorting protein 1	23.74	23.11	-0.63	0.09
NCU00951	inorganic pyrophosphatase	25.08	24.45	-0.63	0.09
NCU07824	MDM10	20.19	19.56	-0.63	0.09
NCU00056	3-oxoacyl-[acyl-carrier-protein]-synthase	20.01	19.38	-0.63	0.09
NCU05427	ATP-dependent Clp protease, variant	22.08	21.44	-0.64	0.08
NCU08707	iron sulfur assembly protein	22.16	21.52	-0.64	0.08
NCU07608	ribose 5-phosphate isomerase A, variant	21.80	21.16	-0.64	0.08
NCU09366	proteasome component C5	18.16	17.51	-0.65	0.07
NCU07955	mitochondrion biogenesis protein	19.90	19.25	-0.65	0.07
NCU06307	multisynthetase complex auxiliary component p43	23.44	22.79	-0.65	0.07
NCU00360	NAD dependent epimerase/dehydratase	24.35	23.70	-0.65	0.07
NCU10225	hypothetical protein	21.78	21.12	-0.66	0.06
NCU05270		20.20	19.54	-0.66	0.06
NCU04225	60S ribosomal protein L20	24.54	23.88	-0.66	0.06
NCU00582	cryptochrome DASH	17.95	17.29	-0.66	0.06
NCU00264	hypothetical protein	22.75	22.09	-0.66	0.06
NCU06518	NADH-cytochrome b5 reductase 2	23.64	22.97	-0.67	0.05
NCU03922	hydroxymethylglutaryl-CoA synthase	19.28	18.61	-0.67	0.05
NCU01962	encodes anonymous transcript-5 protein, variant	24.28	23.61	-0.67	0.05
NCU00895	RAB GTPase Ypt5	24.37	23.70	-0.67	0.05
NCU00775	isocitrate dehydrogenase subunit 1	27.57	26.89	-0.68	0.04
NCU06244	mitochondrial import inner membrane translocas	20.38	19.69	-0.69	0.03
NCU09732	acetyl-CoA acetyltransferase	25.50	24.81	-0.69	0.03
NCU08166	hypothetical protein	18.85	18.16	-0.69	0.03
NCU02580	fumarate reductase Osm1	27.75	27.06	-0.69	0.03
NCU07286	membrane-associated progesterone recepto	22.74	22.04	-0.70	0.02
NCU03199	ATP synthase subunit H	26.37	25.67	-0.70	0.02
NCU01102	mitochondrial export translocase Oxa1	24.56	23.86	-0.70	0.02
NCU00959	succinate dehydrogenase iron-sulfur protein	27.27	26.57	-0.70	0.02
NCU00591	methylcrotonoyl-CoA carboxylase subunit alpha	21.97	21.27	-0.70	0.02
NCU03777	mitochondrial 3-hydroxyisobutyryl-CoA hydrolase	26.51	25.81	-0.70	0.02
NCU08877	glycine cleavage system H protein	25.98	25.27	-0.71	0.01
NCU05515	mitochondrial import inner membrane translocas	23.94	23.23	-0.71	0.01
NCU03155	hypothetical protein	24.60	23.89	-0.71	0.01
NCU01141	CDP-diacylglycerol-glycerol-3-phosphat	21.46	20.75	-0.71	0.01
NCU00673	serine protease p2	25.36	24.65	-0.71	0.01
NCU03229	mitochondrial inner membrane translocase subuni	25.85	25.13	-0.72	0.00
NCU01800	37S ribosomal protein S5	25.53	24.81	-0.72	0.00
NCU07552	hypothetical protein	22.33	21.60	-0.73	-0.01
NCU07724	mitochondrial division protein 1	23.66	22.93	-0.73	-0.01
NCU06880	AhpC/TSA family protein	25.60	24.87	-0.73	-0.01
NCU05552	hypothetical protein	24.96	24.23	-0.73	-0.01
NCU04945	mitochondrial intermembrane space protein Mia40	25.89	25.16	-0.73	-0.01
NCU09688	AP-1 complex subunit mu	19.06	18.32	-0.74	-0.02
NCU09017	hypothetical protein	19.18	18.44	-0.74	-0.02
NCU02804	mitochondrial 60S ribosomal protein L25	24.86	24.12	-0.74	-0.02
NCU03739	protein disulfide-isomerase tigA	23.94	23.20	-0.74	-0.02
NCU03158	alpha/beta hydrolase	22.57	21.83	-0.74	-0.02
NCU09129	hypothetical protein	18.06	17.31	-0.75	-0.02
NCU05541	hypothetical protein	21.74	20.99	-0.75	-0.02
NCU00644	ATP synthase subunit G	26.18	25.43	-0.75	-0.02
NCU03177	sco1	24.11	23.35	-0.76	-0.03
NCU01808	cytochrome c	26.40	25.64	-0.76	-0.03
NCU09598	mitochondrial escape protein 2	25.73	24.97	-0.76	-0.03
NCU08661	pantoate-beta-alanine ligase	21.75	20.99	-0.76	-0.03
NCU08299	60S ribosomal protein L3	24.84	24.08	-0.76	-0.03
NCU03947	ubiquitin ligase	21.60	20.84	-0.76	-0.03

NCU01169	NADH:ubiquinone oxidoreductase 24	26.26	25.50	-0.76	-0.03
NCU09347	fructose-2,6-bisphosphatase	18.78	18.01	-0.77	-0.04
NCU06211	malate dehydrogenase, variant	26.88	26.11	-0.77	-0.04
NCU03857	tricarboxylic acid-5 protein, variant 2	26.97	26.20	-0.77	-0.04
NCU03211	hypothetical protein	24.06	23.29	-0.77	-0.04
NCU01667	ornithine carbamoyltransferase	25.96	25.19	-0.77	-0.04
NCU05248	hypothetical protein	24.63	23.85	-0.78	-0.05
NCU01514	mitochondrial dicarboxylate carrier	20.97	20.19	-0.78	-0.05
NCU07733	electron transfer flavoprotein beta-subunit	26.67	25.89	-0.78	-0.05
NCU04336	60S ribosomal protein L19	25.52	24.74	-0.78	-0.05
NCU03695	phosphatidylserine decarboxylase proenzyme 1	24.21	23.43	-0.78	-0.05
NCU03297	cytochrome c peroxidase	27.85	27.07	-0.78	-0.05
NCU03004	pyruvate dehydrogenase E1 component	27.00	26.22	-0.78	-0.05
NCU05313	mitochondria fission 1 protein	23.59	22.80	-0.79	-0.06
NCU07852	ribosomal protein L13	23.92	23.13	-0.79	-0.06
NCU07493	hypothetical protein	25.74	24.94	-0.80	-0.07
NCU08552	50S ribosomal protein L14	22.80	22.00	-0.80	-0.07
NCU06452	cysteine synthase	26.37	25.57	-0.80	-0.07
NCU03296	F1 ATPase assembly protein 11	22.96	22.16	-0.80	-0.07
NCU02959	hypothetical protein	25.92	25.12	-0.80	-0.07
NCU01975	complex I intermediate-associated protein CIA30	23.42	22.62	-0.80	-0.07
NCU01255	mitochondrial dynamin GTPase	25.11	24.31	-0.80	-0.07
NCU10042	enolase, variant	25.49	24.68	-0.81	-0.08
NCU10028	bax Inhibitor family protein	22.13	21.32	-0.81	-0.08
NCU06760	pyruvate dehydrogenase kinase	25.83	25.02	-0.81	-0.08
NCU03888	DUF500 and SH3 domain-containing protein	19.87	19.06	-0.81	-0.08
NCU01545	autophagy protein 8	20.24	19.42	-0.82	-0.09
NCU09877	hypothetical protein	20.96	20.14	-0.82	-0.09
NCU04768	electron transfer flavoprotein-ubiquinon	26.42	25.60	-0.82	-0.09
NCU00680	2-methylcitrate dehydratase	26.67	25.85	-0.82	-0.09
NCU00676	F1-ATP synthase assembly protein	24.23	23.41	-0.82	-0.09
NCU00051	hypothetical protein	24.36	23.54	-0.82	-0.09
NCU00477	carboxypeptidase Y	23.79	22.96	-0.83	-0.10
NCU08356	acetamidase	23.91	23.08	-0.83	-0.10
NCU08336	succinate dehydrogenase flavoprotein subunit	28.51	27.68	-0.83	-0.10
NCU08471	succinyl-CoA ligase beta-chain	28.07	27.23	-0.84	-0.11
NCU08329	mitochondrial ribosomal protein subunit L23	24.00	23.16	-0.84	-0.11
NCU07310	hypothetical protein	20.24	19.40	-0.84	-0.11
NCU01546	coproporphyrinogen III oxidase	23.95	23.11	-0.84	-0.11
NCU01010	hypothetical protein	15.92	15.08	-0.84	-0.11
NCU00136	Mito Translational Optimization	21.63	20.79	-0.84	-0.11
NCU06451	hypothetical protein	25.29	24.44	-0.85	-0.12
NCU04410	tRNA ligase	18.61	17.76	-0.85	-0.12
NCU00905	N-acylethanolamine amidohydrolase	22.65	21.80	-0.85	-0.12
NCU04806	hypothetical protein	24.14	23.29	-0.85	-0.12
NCU04502	hypothetical protein	23.21	22.36	-0.85	-0.12
NCU03188	sugar 1,4-lactone oxidase	24.76	23.91	-0.85	-0.12
NCU08053	hypothetical protein	18.43	17.57	-0.86	-0.13
NCU06958	mito ribosomal protein S21	21.56	20.70	-0.86	-0.13
NCU04753	NADH:ubiquinone oxidoreductase 11.6kD subunit	23.36	22.50	-0.86	-0.13
NCU06749	60S ribosomal protein L3	26.06	25.19	-0.87	-0.14
NCU09534	peroxiredoxin HYR1	19.21	18.34	-0.87	-0.14
NCU01965	valyl-tRNA synthetase	23.39	22.52	-0.87	-0.14
NCU00896	synaptojanin-1	21.91	21.04	-0.87	-0.14
NCU00828	peroxisomal membrane protein	20.67	19.80	-0.87	-0.14
NCU06038	ribosomal protein L36	19.65	18.77	-0.88	-0.15
NCU04415	hypothetical protein	22.59	21.71	-0.88	-0.15
NCU02438	dihydrolipoamide succinyltransferase	27.07	26.19	-0.88	-0.15
NCU01589	heat shock protein 60	28.36	27.48	-0.88	-0.15
NCU08693	hsp70-like protein	29.27	28.38	-0.89	-0.16
NCU07697	isocitrate dehydrogenase subunit 2	27.60	26.71	-0.89	-0.16
NCU04074	NADH:ubiquinone oxidoreductase 30.4 kDa subunit	26.38	25.49	-0.89	-0.16
NCU02757	60S ribosomal protein L2	24.45	23.56	-0.89	-0.16
NCU02534	NADH:ubiquinone oxidoreductase 49kD subunit	26.64	25.75	-0.89	-0.16
NCU01213	superoxide dismutase	18.85	17.96	-0.89	-0.16
NCU09770	acetyl-coa hydrolase	26.61	25.71	-0.90	-0.17
NCU07549	mitochondrial ribosomal protein L43	23.64	22.74	-0.90	-0.17
NCU04181	TOM7, variant 2	22.12	21.22	-0.90	-0.17
NCU01227	succinyl-CoA ligase alpha-chain	27.14	26.24	-0.90	-0.17
NCU07941	aspartate aminotransferase	23.03	22.12	-0.91	-0.18
NCU06761	sphingosine-1-phosphate lyase	25.98	25.07	-0.91	-0.18
NCU04803	2-nitropropane dioxygenase famil	24.62	23.71	-0.91	-0.18
NCU02639	argininosuccinate synthase, variant	21.89	20.98	-0.91	-0.18
NCU01219	glutaredoxin	21.20	20.29	-0.91	-0.18

NCU00316	peroxisomal adenine nucleotide transporter 1	21.91	21.00	-0.91	-0.18
NCU06469	54S ribosomal protein L12	23.59	22.67	-0.92	-0.19
NCU03216	adenylate kinase	21.98	21.06	-0.92	-0.19
NCU02690	mitochondrial integral membrane protein	22.82	21.90	-0.92	-0.19
NCU07362	L-lactate ferricytochrome c oxidoreductase	23.75	22.82	-0.93	-0.20
NCU06441	D-lactate dehydrogenase 2	26.99	26.06	-0.93	-0.20
NCU03926	37S ribosomal protein Rsm24	25.89	24.96	-0.93	-0.20
NCU00385	ATP synthase subunit delta	24.81	23.87	-0.94	-0.20
NCU09810	succinyl-CoA synthetase subunit alpha	24.52	23.58	-0.94	-0.20
NCU09551	MFS multidrug transporter	19.46	18.52	-0.94	-0.20
NCU09108	hypothetical protein	19.27	18.33	-0.94	-0.20
NCU04380	acyl-CoA synthetase	25.71	24.77	-0.94	-0.20
NCU03653	hypothetical protein	24.89	23.95	-0.94	-0.20
NCU00737	presequence translocated-associated moto	23.00	22.06	-0.94	-0.20
NCU07057	hypothetical protein	22.93	21.98	-0.95	-0.21
NCU04379	neuronal calcium sensor 1	19.29	18.34	-0.95	-0.21
NCU09741	NADPH-cytochrome P450 reductase	23.17	22.22	-0.95	-0.21
NCU02549	ubiquinol-cytochrome-c reductase subunit beta	28.47	27.51	-0.96	-0.22
NCU16791	mitochondrial inner membrane protease subunit 2	19.36	18.40	-0.96	-0.22
NCU05400	hypothetical protein	18.45	17.49	-0.96	-0.22
NCU03882	hypothetical protein	20.66	19.70	-0.96	-0.22
NCU11311	small zinc finger protein Tim8	22.27	21.30	-0.97	-0.23
NCU08898	homoaconitase	26.74	25.77	-0.97	-0.23
NCU01473	mitochondrial large ribosomal subunit YmL35	25.64	24.67	-0.97	-0.23
NCU07659	pyruvate dehydrogenase complex	28.20	27.22	-0.98	-0.24
NCU05029	iron-sulfur clusters transporter atm-1	23.52	22.54	-0.98	-0.24
NCU09999	hypothetical protein	24.86	23.87	-0.99	-0.25
NCU03339	glutathione reductase	24.71	23.72	-0.99	-0.25
NCU10020	methionine synthase	17.50	16.50	-1.00	-0.26
NCU09885	acyl-CoA dehydrogenase	21.00	20.00	-1.00	-0.26
NCU07253	1,3-beta-glucanosyltransferase gel1	21.47	20.47	-1.00	-0.26
NCU04180	mitochondrial folate carrier protein Flx1	20.21	19.21	-1.00	-0.26
NCU07982	acetolactate synthase	26.81	25.80	-1.01	-0.27
NCU09886	hypothetical protein	24.62	23.61	-1.01	-0.27
NCU08861	hypothetical protein	25.28	24.26	-1.02	-0.28
NCU03116	Ras GTPase activating protein	16.26	15.24	-1.02	-0.28
NCU02585	aspartyl-tRNA synthetase	25.01	23.99	-1.02	-0.28
NCU02337	mitochondrial carrier protein	18.76	17.74	-1.02	-0.28
NCU05075	mitochondrial tricarboxylate transporter	23.92	22.89	-1.03	-0.29
NCU06482	pyruvate dehydrogenase E1 component alph	27.93	26.89	-1.04	-0.30
NCU05593	mitochondrial outer membrane beta-barrel protei	25.12	24.07	-1.05	-0.31
NCU04837	mitochondrial 2-oxodicarboxylate carrier 1	22.66	21.61	-1.05	-0.31
NCU08146	hypothetical protein	25.11	24.05	-1.06	-0.32
NCU07280	serine/threonine-protein kinase gad8	21.31	20.25	-1.06	-0.32
NCU05425	2-oxoglutarate dehydrogenase E1 component	28.27	27.21	-1.06	-0.32
NCU04280	aconitate hydratase	26.16	25.10	-1.06	-0.32
NCU02727	glycine cleavage system T protein	26.50	25.44	-1.06	-0.32
NCU06643	oleate-induced peroxisomal protein	23.58	22.51	-1.07	-0.33
NCU06881	succinyl-CoA:3-ketoacid-coenzyme A transferas	25.04	23.97	-1.07	-0.33
NCU02812	uridylate kinase	25.36	24.29	-1.07	-0.33
NCU02545	hypothetical protein	24.93	23.85	-1.08	-0.34
NCU02504	DUF1640 domain-containing protein	22.56	21.48	-1.08	-0.34
NCU01454	mitochondrial hydrolase	23.81	22.73	-1.08	-0.34
NCU09228	aminopeptidase 2	25.64	24.55	-1.09	-0.35
NCU04566	protein kinase SNF1	17.79	16.70	-1.09	-0.35
NCU04013	aldehyde dehydrogenase	25.65	24.56	-1.09	-0.35
NCU02451	mitochondrial hypoxia responsiv	24.22	23.13	-1.09	-0.35
NCU06666	inositol-3-phosphate synthase	22.56	21.46	-1.10	-0.36
NCU10008	fumarate hydratase	26.23	25.13	-1.10	-0.36
NCU07414	DnaJ family protein	21.46	20.36	-1.10	-0.36
NCU05430	ATP synthase beta subunit	30.08	28.97	-1.11	-0.37
NCU03753	clock controlled protein CCG-1	19.48	18.37	-1.11	-0.37
NCU05008	acp-1	25.63	24.51	-1.12	-0.37
NCU09903	elongation factor 3	17.26	16.14	-1.12	-0.37
NCU07560	50S ribosomal subunit L30	24.51	23.39	-1.12	-0.37
NCU08924	acyl-CoA dehydrogenase	24.78	23.65	-1.13	-0.38
NCU09537	rho-GTPase-activating protein 8	19.85	18.71	-1.14	-0.39
NCU05838	hypothetical protein	20.57	19.43	-1.14	-0.39
NCU08120	mitochondrial ribosomal protein DAP3	25.24	24.09	-1.15	-0.40
NCU01444	rheb small monomeric GTPase RhbA	19.20	18.05	-1.15	-0.40
NCU01021		18.95	17.80	-1.15	-0.40
NCU03558	hypothetical protein	24.03	22.88	-1.15	-0.40
NCU05226	ABC transporter	25.24	24.08	-1.16	-0.41
NCU04807	universal stress protein famil	17.93	16.77	-1.16	-0.41

NCU02514	mitochondrial ATP synthase alpha subunit	30.28	29.12	-1.16	-0.41
NCU05717	mito ribosomal protein S6	24.05	22.88	-1.17	-0.42
NCU03877	C-1-tetrahydrofolate synthase	23.91	22.74	-1.17	-0.42
NCU03112	NADH-cytochrome b5 reductase 2	27.55	26.38	-1.17	-0.42
NCU02013	hypothetical protein	24.35	23.17	-1.18	-0.43
NCU01179	outer membrane translocase 40 kDa subunit	25.78	24.60	-1.18	-0.43
NCU08949	hypothetical protein	26.08	24.89	-1.19	-0.44
NCU05881	DUF500 and UBA/TS-N domain-containing protein	22.01	20.82	-1.19	-0.44
NCU02482	2-methylcitrate synthase, variant 2	26.14	24.95	-1.19	-0.44
NCU02419	mitochondrial 37S ribosomal protein S17	24.42	23.23	-1.19	-0.44
NCU01859	NADH:ubiquinone oxidoreductase 20.9kD subunit	25.39	24.20	-1.19	-0.44
NCU08071	ribosomal protein S16	23.86	22.66	-1.20	-0.45
NCU03343	37S ribosomal protein S25	24.88	23.67	-1.21	-0.46
NCU11345	hypothetical protein	25.68	24.46	-1.22	-0.47
NCU02450	threonine dehydratase	17.97	16.75	-1.22	-0.47
NCU01484	rho-type GTPase	25.47	24.25	-1.22	-0.47
NCU04044	NADH2 dehydrogenase flavoprotein 1	27.15	25.92	-1.23	-0.48
NCU00605	ThiF domain-containing protein	24.65	23.42	-1.23	-0.48
NCU08980	alternative NADH-dehydrogenase	25.84	24.61	-1.23	-0.48
NCU04230	isocitrate lyase	26.03	24.80	-1.23	-0.48
NCU03813	formate dehydrogenase	21.68	20.45	-1.23	-0.48
NCU02269	mitochondrial carnitine/acylcarnitine carrie	23.88	22.65	-1.23	-0.48
NCU01692	mitochondrial citrate synthase	28.78	27.55	-1.23	-0.48
NCU01606	ATP synthase subunit 5	27.85	26.62	-1.23	-0.48
NCU00894	hypothetical protein	27.50	26.27	-1.23	-0.48
NCU05633	stomatin family protein	26.07	24.83	-1.24	-0.49
NCU05526	homocitrate synthase, variant 2	21.71	20.47	-1.24	-0.49
NCU04249	hypothetical protein	19.17	17.93	-1.24	-0.49
NCU12023	mito ribosomal protein S2	24.91	23.66	-1.25	-0.50
NCU08272	cytochrome b2	26.37	25.12	-1.25	-0.50
NCU03802	trimethyllysine dioxygenase	24.29	23.04	-1.25	-0.50
NCU02657	s-adenosylmethionine synthetase, variant	22.33	21.08	-1.25	-0.50
NCU00564	mitochondrial 40S ribosomal protein MRP2	23.41	22.16	-1.25	-0.50
NCU00166	isochorismatase domain-containing protein 2A	23.02	21.77	-1.25	-0.50
NCU09463	leucyl-tRNA synthetase	22.08	20.82	-1.26	-0.51
NCU02407	dihydrolipoyl dehydrogenase	28.58	27.32	-1.26	-0.51
NCU09119	ATP synthase subunit gamma	27.34	26.08	-1.26	-0.51
NCU03982	glucose-regulated protein	26.01	24.75	-1.26	-0.51
NCU01905	sorbitol dehydrogenase	16.46	15.20	-1.26	-0.51
NCU00430	Na	18.52	17.25	-1.27	-0.52
NCU00573	hypothetical protein	22.10	20.83	-1.27	-0.52
NCU04636	cysteine desulfurase	26.20	24.92	-1.28	-0.53
NCU01747	glycerophosphocholine phosphodiesterase Gde1	17.06	15.77	-1.29	-0.54
NCU10048	3-methyl-2-oxobutanoat	21.17	19.87	-1.30	-0.55
NCU07879	mitochondrial metal transporter 2	20.13	18.83	-1.30	-0.55
NCU07384	mitochondrial thiamine pyrophosphate carrier 1	20.12	18.82	-1.30	-0.55
NCU02295	phosphatidylinositol-4-phosphate 5-kinase its3	20.37	19.07	-1.30	-0.55
NCU09223	protein disulfide-isomerase	25.70	24.39	-1.31	-0.55
NCU04569	5-oxoprolinase	20.58	19.27	-1.31	-0.55
NCU04138	hypothetical protein	24.06	22.75	-1.31	-0.55
NCU03230	mitochondrial ribosomal protein subunit S18	24.08	22.77	-1.31	-0.55
NCU09477	ADP/ATP carrier protein, variant	29.64	28.33	-1.31	-0.55
NCU02544	ABC transporter	23.39	22.08	-1.31	-0.55
NCU10468	arginine biosynthesis argJ	26.83	25.51	-1.32	-0.56
NCU04946	hypothetical protein	22.56	21.24	-1.32	-0.56
NCU02064	hypothetical protein	26.32	25.00	-1.32	-0.56
NCU01528	glyceraldehyde 3-phosphate-dehydrogenase	25.12	23.80	-1.32	-0.56
NCU04899	malate dehydrogenase	29.54	28.20	-1.34	-0.58
NCU01765	NADH:ubiquinone oxidoreductase 78	27.73	26.39	-1.34	-0.58
NCU00050	pyruvate dehydrogenase X component	26.62	25.28	-1.34	-0.58
NCU01680	plasma membrane ATPase	27.88	26.53	-1.35	-0.59
NCU05299	NADH:ubiquinone oxidoreductase	25.68	24.33	-1.35	-0.59
NCU04579	dihydroxy-acid dehydratase	27.64	26.29	-1.35	-0.59
NCU03556	peroxisomal membrane protein Pmp47	24.03	22.68	-1.35	-0.59
NCU02133	superoxide dismutase	23.80	22.45	-1.35	-0.59
NCU01650	hypothetical protein	24.30	22.95	-1.35	-0.59
NCU00114	30S ribosomal protein S10	25.17	23.82	-1.35	-0.59
NCU07884	mitochondrial oxaloacetate transporter	22.50	21.14	-1.36	-0.60
NCU05027	hypothetical protein	25.70	24.34	-1.36	-0.60
NCU02623	mitochondrial hypoxia responsiv	25.73	24.37	-1.36	-0.60
NCU07756	succinate dehydrogenase cytochrome b560 subunit	26.42	25.06	-1.36	-0.60
NCU17017	mitochondrial inner membrane protease subunit 1	19.37	18.00	-1.37	-0.61
NCU05454	glycerol-3-phosphate dehydrogenase	27.49	26.11	-1.38	-0.62
NCU04412	hypothetical protein	24.63	23.25	-1.38	-0.62

NCU00567	Arg-6 protein, variant	27.75	26.37	-1.38	-0.62
NCU00436	GTPase FZO1	23.99	22.61	-1.38	-0.62
NCU07112	hypothetical protein	26.92	25.54	-1.38	-0.62
NCU06662	mitochondrial carrier protein	20.40	19.00	-1.40	-0.64
NCU01878	vesicle-mediated transporter	17.91	16.51	-1.40	-0.64
NCU03076	delta-1-pyrroline-5-carboxylate dehydrogenase	26.32	24.91	-1.41	-0.65
NCU06768	60S ribosomal protein L16	23.50	22.07	-1.43	-0.67
NCU01550	adenylate kinase cytosolic	27.49	26.05	-1.44	-0.68
NCU01485	hypothetical protein	26.15	24.71	-1.44	-0.68
NCU06875	hypothetical protein	18.03	16.59	-1.44	-0.68
NCU03711	GTP-binding protein ypt7	24.53	23.09	-1.44	-0.68
NCU02951	40S ribosomal protein S8	24.27	22.83	-1.44	-0.68
NCU08720	hypothetical protein	19.06	17.61	-1.45	-0.69
NCU04481	mitochondrial ATPase	24.13	22.68	-1.45	-0.69
NCU00411	hypothetical protein	22.16	20.71	-1.45	-0.69
NCU03129	threonyl-tRNA synthetase	22.88	21.42	-1.46	-0.70
NCU03735	hypothetical protein	20.69	19.21	-1.48	-0.72
NCU01821	alanine-glyoxylate aminotransferase	24.97	23.49	-1.48	-0.72
NCU04754	branched-chain-amino-acid aminotransferase	26.76	25.27	-1.49	-0.72
NCU06336	N2,N2-dimethylguanosine tRNA methyltransferase	17.72	16.22	-1.50	-0.73
NCU05221	NADH:ubiquinone oxidoreductase 21kD subunit	26.68	25.15	-1.53	-0.76
NCU00969	NADH:ubiquinone oxidoreductase 17.8kD subunit	24.32	22.78	-1.54	-0.77
NCU00536	homoserine O-acetyltransferase	24.83	23.28	-1.55	-0.78
NCU00461	NAD-specific glutamate dehydrogenase	16.84	15.29	-1.55	-0.78
NCU09123	Ca/CaM-dependent kinase-1, variant 2	20.61	19.05	-1.56	-0.79
NCU00789	DUF221 domain-containing protein	22.70	21.14	-1.56	-0.79
NCU11258	LYR family protein	26.03	24.47	-1.56	-0.79
NCU03966	mitochondrial Rho GTPase 1	23.05	21.48	-1.57	-0.80
NCU01241	mitochondrial carrier protein	26.53	24.96	-1.57	-0.80
NCU03837	Snf1 kinase complex beta-subunit Gal83	17.79	16.21	-1.58	-0.81
NCU00684	endonuclease/Exonuclease/phosphatase	21.90	20.32	-1.58	-0.81
NCU00431	mitochondrial import receptor subunit Tom22	25.47	23.88	-1.59	-0.82
NCU04421	annexin XIV, variant	16.04	14.44	-1.60	-0.83
NCU11348	NADH-ubiquinone oxidoreductase B18 subunit	24.18	22.57	-1.61	-0.84
NCU06524	protease inhibitor	19.15	17.54	-1.61	-0.84
NCU04245	outer mitochondrial membrane translocase	27.52	25.91	-1.61	-0.84
NCU03608	ketol-acid reductoisomerase	29.06	27.44	-1.62	-0.85
NCU07732	carbamoyl-phosphate synthase small subunit	25.98	24.35	-1.63	-0.86
NCU04783	EH domain binding protein epsin 2	19.61	17.98	-1.63	-0.86
NCU04265	invertase	20.77	19.14	-1.63	-0.86
NCU01761	hypothetical protein	23.34	21.71	-1.63	-0.86
NCU02475	glycine dehydrogenase	26.88	25.23	-1.65	-0.88
NCU05390	mitochondrial phosphate carrier protein	28.67	27.02	-1.65	-0.88
NCU02280	NADH:ubiquinone oxidoreductase 21.3kD subunit B	25.71	24.05	-1.66	-0.89
NCU01689	mitochondrial DNA replication protein YHM2	24.20	22.54	-1.66	-0.89
NCU01666	acetolactate synthase small subunit	26.84	25.18	-1.66	-0.89
NCU02677	carbamoyl-phosphate synthase large subunit	28.56	26.89	-1.67	-0.89
NCU08973	SNARE protein Ykt6	21.81	20.13	-1.68	-0.90
NCU06450	mitochondrial ribosomal protein	24.71	23.03	-1.68	-0.90
NCU05706	glutathione S-transferase	20.94	19.26	-1.68	-0.90
NCU02972	hypothetical protein	21.04	19.35	-1.69	-0.91
NCU08621	hypothetical protein	18.51	16.82	-1.69	-0.91
NCU08411	aspartate aminotransferase	27.89	26.20	-1.69	-0.91
NCU00502	ATP synthase subunit 4	27.55	25.86	-1.69	-0.91
NCU08541	hypothetical protein	21.68	19.98	-1.70	-0.92
NCU06924	kynurenine 3-monooxygenase	25.16	23.45	-1.71	-0.93
NCU05410	acetylornithine aminotransferase	26.68	24.97	-1.71	-0.93
NCU08354	hypothetical protein	23.74	22.02	-1.72	-0.94
NCU03661	GTPase-activating protein GYP7	18.93	17.21	-1.72	-0.94
NCU02618	peroxisomal membrane protein	20.72	19.00	-1.72	-0.94
NCU03493	glycine rich protein	20.30	18.58	-1.72	-0.94
NCU09175	GPI-anchored cell wall beta-1,3-endoglucanas	23.05	21.32	-1.73	-0.95
NCU04174	hypothetical protein	21.04	19.31	-1.73	-0.95
NCU03559	ubiquinol-cytochrome c reductase complex cor	27.91	26.17	-1.74	-0.96
NCU03214	ER lumen protein retaining receptor	18.78	17.04	-1.74	-0.96
NCU03093	NADH:ubiquinone oxidoreductase 12.3kD subunit	25.48	23.72	-1.76	-0.98
NCU05266	KH domain-containing protein	17.55	15.78	-1.77	-0.99
NCU00227	mitochondrial cation transporter	23.27	21.49	-1.78	-1.00
NCU05890	hypothetical protein	22.07	20.28	-1.79	-1.01
NCU08066	amino acid transporter	18.47	16.67	-1.80	-1.02
NCU05457	cytochrome c oxidase subunit IV	27.07	25.26	-1.81	-1.03
NCU08358	hypothetical protein	22.39	20.58	-1.81	-1.03
NCU04605	hypothetical protein	24.56	22.74	-1.82	-1.04
NCU01401	mitochondrial outer membrane protein, variant	23.06	21.24	-1.82	-1.04

NCU07495	sphingolipid long chain base-responsive protei	21.08	19.24	-1.84	-1.06
NCU04814	DUF21 and CBS domain-containing protein	22.39	20.55	-1.84	-1.06
NCU09143	hypothetical protein	22.65	20.80	-1.85	-1.07
NCU00101	pbn-1	21.18	19.33	-1.85	-1.07
NCU05220	ATP synthase subunit F	26.23	24.37	-1.86	-1.07
NCU06606	ubiquinol-cytochrome c reductase iron-sulfu	27.06	25.19	-1.87	-1.08
NCU02373	NADH-ubiquinone oxidoreductase 40 kDa subunit	26.91	25.02	-1.89	-1.10
NCU08948	NIF domain-containing protein	18.09	16.19	-1.90	-1.11
NCU01101	mitochondrial import protein 1	22.18	20.28	-1.90	-1.11
NCU08561	succinate/fumarate mitochondrial transporter	23.16	21.25	-1.91	-1.12
NCU03798	mitochondrial import inner membrane translocas	20.06	18.14	-1.92	-1.13
NCU06695	cytochrome c oxidase polypeptide VI, variant	27.13	25.20	-1.93	-1.14
NCU02391	protein transporter sec-24	20.18	18.24	-1.94	-1.15
NCU08930	NADH:ubiquinone oxidoreductase 21.3kD subunit A	26.22	24.27	-1.95	-1.16
NCU08269	pyridoxamine 5'-phosphate oxidase	20.17	18.22	-1.95	-1.16
NCU01371	UPF0660 protein	20.50	18.53	-1.97	-1.18
NCU00418	NADH:ubiquinone oxidoreductase 14.8kD subunit	25.70	23.71	-1.99	-1.20
NCU06678	exonuclease Kem1	20.79	18.78	-2.01	-1.22
NCU05689	cytochrome c oxidase polypeptide IV	27.20	25.19	-2.01	-1.22
NCU05777	ubiquitin carboxyl-terminal hydrolase 14	16.88	14.87	-2.01	-1.22
NCU06741	cytochrome c oxidase subunit VIb	25.97	23.91	-2.06	-1.26
NCU01422	multiprotein-bridging factor 1	20.37	18.31	-2.06	-1.26
NCU08947	ubiquinol-cytochrome-c reductase chain VIII	26.66	24.59	-2.07	-1.27
NCU03913	2-oxoisovalerate dehydrogenase beta subunit	19.52	17.45	-2.07	-1.27
NCU09002	NADH:ubiquinone oxidoreductase 10.6kD subunit	24.27	22.18	-2.09	-1.29
NCU03827	40S ribosomal protein S9	25.33	23.24	-2.09	-1.29
NCU00519	ribulose-phosphate 3-epimerase	17.93	15.83	-2.10	-1.30
NCU02074	endoplasmic oxidoreductin-1	22.07	19.97	-2.10	-1.30
NCU00930	mitochondrial import inner membrane translocas	22.49	20.38	-2.11	-1.31
NCU00249	hypothetical protein	17.13	15.01	-2.12	-1.32
NCU00352	phospholipid-transporting ATPase	19.19	17.07	-2.12	-1.32
NCU04276	hypothetical protein	24.65	22.52	-2.13	-1.33
NCU09816	cytochrome-26	28.20	26.06	-2.14	-1.34
NCU06430	capsule-associated protein CAP1	20.88	18.69	-2.19	-1.39
NCU01070	hypothetical protein	19.99	17.80	-2.19	-1.39
NCU03602	hypothetical protein	19.10	16.90	-2.20	-1.40
NCU03953	mitochondrial NADH-ubiquinone oxidoreductase 2	25.38	23.17	-2.21	-1.41
NCU06189	5-aminolevulinate synthase	25.85	23.64	-2.21	-1.41
NCU04645	DUF124 domain-containing protein	20.62	18.40	-2.22	-1.41
NCU08939	mannan polymerase II complex ANP1 subunit	20.74	18.49	-2.25	-1.44
NCU00216	NADH-cytochrome b5 reductase 1	22.62	20.37	-2.25	-1.44
NCU06317	stress response RCI peptide	21.56	19.30	-2.26	-1.45
NCU10311	mitochondrial ribosomal protein subunit S4	23.35	21.09	-2.26	-1.45
NCU03092	nuclear localization sequence binding protein	17.92	15.65	-2.27	-1.46
NCU09808	dynamin-1	21.22	18.92	-2.30	-1.49
NCU01142	NADH:ubiquinone oxidoreductase 13.4kD subunit	25.33	23.03	-2.30	-1.49
NCU08957	hypothetical protein	20.87	18.54	-2.33	-1.52
NCU05513	hypothetical protein	20.67	18.31	-2.36	-1.55
NCU09299	NADH:ubiquinone oxidoreductase 14kD subunit	24.83	22.45	-2.38	-1.57
NCU05564	peroxisomal membrane protein PEX31, variant 2	19.02	16.63	-2.39	-1.58
NCU02202	serine/threonine kinase IREI	19.10	16.69	-2.41	-1.59
NCU06410	GTP-binding protein YPT52	21.24	18.74	-2.50	-1.68
NCU06943	SIK1	20.02	17.50	-2.52	-1.70
NCU02564	cysteine synthase 2	23.40	20.88	-2.52	-1.70
NCU01360	NADH:ubiquinone oxidoreductase 11.5kD subunit	23.09	20.57	-2.52	-1.70
NCU01467	NADH:ubiquinone oxidoreductase 10.4kD subunit	24.18	21.65	-2.53	-1.71
NCU01633	hexose transporter HXT13	25.89	23.35	-2.54	-1.72
NCU06332	alpha/beta hydrolase	21.78	19.24	-2.54	-1.72
NCU01754	alcohol dehydrogenase I	23.65	21.10	-2.55	-1.73
NCU07075	calcium/proton exchanger, variant	21.97	19.39	-2.58	-1.76
NCU02635	mannan polymerase complexes MNN9 subunit	20.84	18.26	-2.58	-1.76
NCU08699	bli-4 protein	21.98	19.39	-2.59	-1.76
NCU00459	hypothetical protein	18.40	15.79	-2.61	-1.78
NCU06542	mitochondrial 30S ribosomal protein S12	23.21	20.57	-2.64	-1.81
NCU06149	ATP-dependent RNA helicase dhh-1	19.60	16.94	-2.66	-1.83

Table S4 Data from S100 Cytosolic Proteome (Log of total ion counts)

increased levels - green; decreased levels - red

		WT-1	WT-2	delta Po	delta Por	*AVG*	*0.02*	*0.13*	*0.28*	*0.39*	*SD*	*0.70*	*0.75*	*1.27*	*1.23*		average
**protein**	**description**	**nc1s**	**nc2s**	**nc5s**	**nc6s**		**R0**	**R1**	**Z0**	**Z1**		**NR0**	**NR1**	**NZ0**	**NZ1**	**SN**	**Z0 Z1**
NCU00021	hypothetical protein	17.22	16.06	19.57	20.19		-1.16	0.62	2.35	4.13		-1.18	0.49	2.07	3.74	3.36	3.24
NCU05780	glutathione S-transferase-1	14.39	14.48	20.09	19.33		0.09	-0.76	5.70	4.85		0.07	-0.89	5.42	4.46	7.89	5.28
NCU09519	2,5-diketo-D-gluconic acid reductase A	13.08	13.24	17.79	17.12		0.16	-0.67	4.71	3.88		0.14	-0.80	4.43	3.49	6.97	4.30
NCU10572	short chain oxidoreductase	17.40	19.04	21.88	21.95		1.64	0.07	4.48	2.91		1.62	-0.06	4.20	2.52	3.02	3.70
NCU01272	mitochondrial presequence protease	16.01	16.84	20.30	20.07		0.83	-0.23	4.29	3.23		0.81	-0.36	4.01	2.84	5.53	3.76
NCU02549	processing enhancing protein	16.37	17.08	20.60	20.87		0.71	0.27	4.23	3.79		0.69	0.14	3.95	3.40	7.36	4.01
NCU09533	NAD binding Rossmann fold oxidoreductase	14.55	15.25	18.65	18.73		0.70	0.08	4.10	3.48		0.68	-0.05	3.82	3.09	7.17	3.79
NCU02514	ATPase-1	18.44	18.17	22.28	21.36		-0.27	-0.92	3.84	3.19		-0.29	-1.05	3.56	2.80	4.18	3.52
NCU09559	clock-controlled gene-9	14.15	14.61	17.99	18.81		0.46	0.82	3.84	4.20		0.44	0.69	3.56	3.81	6.34	4.02
NCU03737	elongation factor Tu	15.79	15.65	19.50	19.73		-0.14	0.23	3.71	4.08		-0.16	0.10	3.43	3.69	26.98	3.90
NCU09560	superoxide dismutase	16.06	16.18	19.75	18.72		0.12	-1.03	3.69	2.54		0.10	-1.16	3.41	2.15	3.47	3.12
NCU05770	catalase-2	18.70	18.88	22.28	22.45		0.18	0.17	3.58	3.57		0.16	0.04	3.30	3.18	27.06	3.58
NCU03739	ERP38 protein	14.80	15.96	18.35	18.07		1.16	-0.28	3.55	2.11		1.14	-0.41	3.27	1.72	3.05	2.83
NCU04930	hypothetical protein	15.43	16.03	18.97	19.34		0.60	0.37	3.54	3.31		0.58	0.24	3.26	2.92	6.93	3.43
NCU06441	D-lactate dehydrogenase 2	15.59	16.49	18.92	18.76		0.90	-0.16	3.33	2.27		0.88	-0.29	3.05	1.88	3.86	2.80
NCU08130	hypothetical protein	17.61	17.87	20.93	20.74		0.26	-0.19	3.32	2.87		0.24	-0.32	3.04	2.48	9.82	3.10
NCU02809	hypothetical protein	11.30	11.09	14.54	16.64		-0.21	2.10	3.24	5.55		-0.23	1.97	2.96	5.16	3.00	4.40
NCU01589	heat shock protein 60	18.70	18.83	21.81	21.64		0.13	-0.17	3.11	2.81		0.11	-0.30	2.83	2.42	11.72	2.96
NCU06086	regulatory protein suaprga1	14.88	14.13	17.99	17.85		-0.75	-0.14	3.11	3.72		-0.77	-0.27	2.83	3.33	5.39	3.42
NCU00355	catalase-3	19.04	19.52	22.00	22.42		0.48	0.42	2.96	2.90		0.46	0.29	2.68	2.51	6.70	2.93
NCU00549	glutathione transferase omega-1	15.69	14.97	18.64	18.07		-0.72	-0.57	2.95	3.10		-0.74	-0.70	2.67	2.71	3.76	3.03
NCU09674	O-methyltransferase family 3	17.82	18.46	20.76	20.98		0.64	0.22	2.94	2.52		0.62	0.09	2.66	2.13	5.41	2.73
NCU04483	sedoheptulose-1,7-bisphosphatase	15.21	16.00	18.12	18.42		0.79	0.30	2.91	2.42		0.77	0.17	2.63	2.03	4.20	2.67
NCU03611	chitin synthase-1	13.34	13.88	16.16	17.03		0.54	0.87	2.82	3.15		0.52	0.74	2.54	2.76	4.13	2.99
NCU02812	uridylate kinase	16.39	16.03	19.17	19.06		-0.36	-0.11	2.78	3.03		-0.38	-0.24	2.50	2.64	8.19	2.91
NCU02771	uroporphyrinogen decarboxylase	19.84	20.67	22.58	22.50		0.83	-0.08	2.74	1.83		0.81	-0.21	2.46	1.44	3.40	2.29
NCU08402	zinc-binding alcohol dehydrogenase	17.90	18.00	20.62	20.84		0.10	0.22	2.72	2.84		0.08	0.09	2.44	2.45	27.63	2.78
NCU02899	hypothetical protein	14.38	14.51	17.07	16.29		0.13	-0.78	2.69	1.78		0.11	-0.91	2.41	1.39	3.05	2.24
NCU03795	cell division control protein 12	16.48	16.27	19.14	18.92		-0.21	-0.22	2.66	2.65		-0.23	-0.35	2.38	2.26	7.94	2.66
NCU08044	oxidoreductase	17.37	18.10	20.02	19.56		0.73	-0.46	2.65	1.46		0.71	-0.59	2.37	1.07	2.82	2.06
NCU08693	heat shock protein 70-5	20.41	20.30	23.06	23.36		-0.11	0.30	2.65	3.06		-0.13	0.17	2.37	2.67	16.70	2.86
NCU00720	tricarboxylic acid-17	13.99	14.92	16.63	16.80		0.93	0.17	2.64	1.88		0.91	0.04	2.36	1.49	3.06	2.26
NCU03949	nitropropane dioxygenase-1	18.77	19.16	21.39	21.45		0.39	0.06	2.62	2.29		0.37	-0.07	2.34	1.90	7.95	2.46
NCU05430	ATPase-2	19.88	19.60	22.41	21.96		-0.28	-0.45	2.53	2.36		-0.30	-0.58	2.25	1.97	4.62	2.45
NCU04129	gamma-tocopherol methyltransferase	15.82	15.68	18.28	18.22		-0.14	-0.06	2.46	2.54		-0.16	-0.19	2.18	2.15	12.59	2.50
NCU05881	DUF500 and UBA/TS-N domain-containing protein	15.37	14.83	17.81	17.33		-0.54	-0.48	2.44	2.50		-0.56	-0.61	2.16	2.11	3.67	2.47
NCU10810	mRNA splicing protein	14.28	14.89	16.72	16.49		0.61	-0.23	2.44	1.60		0.59	-0.36	2.16	1.21	3.58	2.02
NCU02727	glycine cleavage system T protein	15.86	16.14	18.22	17.73		0.28	-0.49	2.36	1.59		0.26	-0.62	2.08	1.20	3.59	1.98
NCU06738	protein transporter sec-31	15.62	15.77	17.94	17.50		0.15	-0.44	2.32	1.73		0.13	-0.57	2.04	1.34	4.20	2.03
NCU07008	carotenoid oxygenase-1	14.92	13.80	17.22	18.02		-1.12	0.80	2.30	4.22		-1.14	0.67	2.02	3.83	3.28	3.26
NCU03786	serine/threonine protein phosphatase 2A	13.30	13.87	15.58	15.68		0.57	0.10	2.28	1.81		0.55	-0.03	2.00	1.42	4.43	2.05
NCU02464	neuronal-specific septin-3	16.60	16.91	18.86	18.47		0.31	-0.39	2.26	1.56		0.29	-0.52	1.98	1.17	3.88	1.91
NCU09090	phosphopantothenate-cysteine ligase	15.56	16.03	17.77	17.81		0.47	0.04	2.21	1.78		0.45	-0.09	1.93	1.39	5.16	2.00
NCU01166	microcycle blastoconidiation	16.11	16.13	18.27	17.91		0.02	-0.36	2.16	1.78		0.00	-0.49	1.88	1.39	4.81	1.97
NCU01258	cyanase	18.31	17.77	20.47	20.52		-0.54	0.05	2.16	2.75		-0.56	-0.08	1.88	2.36	5.38	2.46
NCU06915	hypothetical protein	15.38	14.84	17.52	18.19		-0.54	0.67	2.14	3.35		-0.56	0.54	1.86	2.96	4.50	2.75
NCU03068	pyridoxine-3	16.63	16.62	18.76	18.33		-0.01	-0.43	2.13	1.71		-0.03	-0.56	1.85	1.32	4.08	1.92
NCU00880	phospholipase A-2-activating protein	16.26	14.99	18.37	18.75		-1.27	0.38	2.11	3.76		-1.29	0.25	1.83	3.37	2.93	2.94
NCU09798	aryl-alcohol dehydrogenase	15.38	16.13	17.48	17.59		0.75	0.11	2.10	1.46		0.73	-0.02	1.82	1.07	2.88	1.78
NCU03596	CRAL/TRIO domain-containing protein	14.21	14.41	16.23	15.75		0.20	-0.48	2.02	1.34		0.18	-0.61	1.74	0.95	3.13	1.68
NCU02113	ubiquitin-conjugating enzyme E2 13	15.84	16.37	17.84	17.79		0.53	-0.05	2.00	1.42		0.51	-0.18	1.72	1.03	3.70	1.71
NCU07659	acetate-4	17.61	17.11	19.60	20.02		-0.50	0.42	1.99	2.91		-0.52	0.29	1.71	2.52	5.14	2.45
NCU08004	electron transfer flavoprotein alpha-subunit	15.03	14.51	16.99	16.49		-0.52	-0.50	1.96	1.98		-0.54	-0.63	1.68	1.59	)=SQRT((	1.97
NCU04216	adenine-7	16.66	16.40	18.59	18.61		-0.26	0.02	1.93	2.21		-0.28	-0.11	1.65	1.82	8.32	2.07
NCU06974	histidinol-phosphatase	15.36	15.70	17.27	17.49		0.34	0.22	1.91	1.79		0.32	0.09	1.63	1.40	6.39	1.85
NCU00488	protein phosphatase PP2A regulatory subunit A	18.53	18.23	20.42	20.49		-0.30	0.07	1.89	2.26		-0.32	-0.06	1.61	1.87	7.70	2.08
NCU01424	DUF636 domain-containing protein	18.61	19.03	20.48	21.08		0.42	0.60	1.87	2.05		0.40	0.47	1.59	1.66	3.70	1.96
NCU00461	glutamate dehydrogenase-1	14.85	15.07	16.67	16.27		0.22	-0.40	1.82	1.20		0.20	-0.53	1.54	0.81	3.09	1.51
NCU02510	clathrin heavy chain	19.80	20.06	21.55	21.29		0.26	-0.26	1.75	1.23		0.24	-0.39	1.47	0.84	3.71	1.49
NCU05143	rds1	15.67	15.23	17.38	17.26		-0.44	-0.12	1.71	2.03		-0.46	-0.25	1.43	1.64	4.21	1.87
NCU00864	TIM-barrel enzyme family protein	17.13	17.54	18.83	18.86		0.41	0.03	1.70	1.32		0.39	-0.10	1.42	0.93	4.20	1.51
NCU01919	ubiquitin C-terminal hydrolase	17.29	17.15	18.99	18.72		-0.14	-0.27	1.70	1.57		-0.16	-0.40	1.42	1.18	4.34	1.64
NCU07737	salicylate hydroxylase	18.16	18.51	19.84	20.37		0.35	0.53	1.68	1.86		0.33	0.40	1.40	1.47	3.89	1.77
NCU09223	protein disulfide-isomerase	17.17	16.58	18.84	18.70		-0.59	-0.14	1.67	2.12		-0.61	-0.27	1.39	1.73	3.36	1.90
NCU06372	ubiquitin hydrolase L3	17.21	16.56	18.73	18.75		-0.65	0.02	1.52	2.19		-0.67	-0.11	1.24	1.80	3.25	1.86
NCU10360	hypothetical protein	16.21	16.26	17.73	17.89		0.05	0.16	1.52	1.63		0.03	0.03	1.24	1.24	37.12	1.58
NCU08515	vacuolar membrane ATPase-2	19.58	19.46	21.09	21.22		-0.12	0.13	1.51	1.76		-0.14	0.00	1.23	1.37	13.57	1.64
NCU01207	vacuolar membrane ATPase-1	20.74	20.81	22.20	22.05		0.07	-0.15	1.46	1.24		0.05	-0.28	1.18	0.85	5.17	1.35
NCU05495	clock-controlled gene-16	16.78	16.63	18.23	18.95		-0.15	0.72	1.45	2.32		-0.17	0.59	1.17	1.93	3.67	1.89
NCU02366	tricarboxylic acid-3	21.08	21.22	22.52	22.24		0.14	-0.28	1.44	1.02		0.12	-0.41	1.16	0.63	3.11	1.23
NCU01004	phosphatidylserine decarboxylase proenzyme	17.17	17.35	18.61	18.65		0.18	0.04	1.44	1.30		0.16	-0.09	1.16	0.91	7.97	1.37
NCU07282	hypothetical protein	15.33	15.23	16.73	16.89		-0.10	0.16	1.40	1.66		-0.12	0.03	1.12	1.27	14.08	1.53
NCU02055	uridine nucleosidase Urh1	16.30	16.59	17.69	17.57		0.29	-0.12	1.39	0.98		0.27	-0.25	1.11	0.59	3.42	1.19
NCU11369	BAR domain-containing protein	18.24	18.35	19.62	19.68		0.11	0.06	1.38	1.33		0.09	-0.07	1.10	0.94	12.58	1.36
NCU08384	xylose reductase	14.96	15.37	16.34	16.79		0.41	0.45	1.38	1.42		0.39	0.32	1.10	1.03	2.97	1.40
NCU00173	esterase D	18.28	18.72	19.64	19.80		0.44	0.16	1.36	1.08		0.42	0.03	1.08	0.69	3.03	1.22
NCU01227	tricarboxylic acid-8	17.48	16.84	18.84	18.90		-0.64	0.06	1.36	2.06		-0.66	-0.07	1.08	1.67	3.02	1.71
NCU01820	exportin-1	18.47	18.66	19.83	19.82		0.19	-0.01	1.36	1.16		0.17	-0.14	1.08	0.77	6.01	1.26
NCU00685	casein kinase I isoform delta	16.42	16.25	17.77	17.84		-0.17	0.07	1.35	1.59		-0.19	-0.06	1.07	1.20	8.29	1.47
NCU05291	polyamine acetyltransferase	15.37	15.16	16.71	16.65		-0.21	-0.06	1.34	1.49		-0.23	-0.19	1.06	1.10	5.22	1.42
NCU01808	cytochrome c-1	17.48	17.55	18.80	18.61		0.07	-0.19	1.32	1.06		0.05	-0.32	1.04	0.67	3.86	1.19
NCU00350	epoxide hydrolase	19.24	19.26	20.47	21.01		0.02	0.54	1.23	1.75		0.00	0.41	0.95	1.36	4.03	1.49
NCU06482	acetate-2	17.79	18.17	19.02	19.25		0.38	0.23	1.23	1.08		0.36	0.10	0.95	0.69	3.12	1.16
NCU05594	L-galactose dehydrogenase	18.30	18.58	19.52	19.81		0.28	0.29	1.22	1.23		0.26	0.16	0.94	0.84	4.08	1.23
NCU11186	NUDIX family hydrolase	14.72	14.23	15.87	16.02		-0.49	0.15	1.15	1.79		-0.51	0.02	0.87	1.40	3.26	1.47
NCU04069	3'-phosphoadenosine 5'-phosphatase isoform B	19.38	19.20	20.49	20.85		-0.18	0.36	1.11	1.65		-0.20	0.23	0.83	1.26	4.97	1.38
NCU05264	pyrimidine 5'-nucleotidase	17.34	17.51	18.45	18.72		0.17	0.27	1.11	1.21		0.15	0.14	0.83	0.82	5.58	1.16
NCU03970	riboflavin kinase	16.58	16.55	17.68	18.14		-0.03	0.46	1.10	1.59		-0.05	0.33	0.82	1.20	4.34	1.35
NCU05041	trehalose-phosphatase	19.32	18.96	20.42	20.30		-0.36	-0.12	1.10	1.34		-0.38	-0.25	0.82	0.95	2.80	1.22
NCU07273	hypothetical protein	15.10	14.61	16.16	16.73		-0.49	0.57	1.06	2.12		-0.51	0.44	0.78	1.73	2.83	1.59
NCU08171	anchored cell wall protein-12	17.66	17.13	18.72	19.15		-0.53	0.43	1.06	2.02		-0.55	0.30	0.78	1.63	2.90	1.54
NCU01692	citrate synthase	21.36	21.49	22.40	22.55		0.13	0.15	1.04	1.06		0.11	0.02	0.76	0.67	8.75	1.05
NCU01440	myosin-2	16.10	16.14	17.13	16.96		0.04	-0.17	1.03	0.82		0.02	-0.30	0.75	0.43	2.92	0.92
NCU04757	hypothetical protein	16.70	16.87	17.71	17.88		0.17	0.17	1.01	1.01		0.15	0.04	0.73	0.62	6.02	1.01
NCU04040	tRNA	15.62	15.28	16.62	16.71		-0.34	0.09	1.00	1.43		-0.36	-0.04	0.72	1.04	3.55	1.22

NCU04404	coatomer beta subunit	17.93	17.89	18.92	19.05		-0.04	0.13	0.99	1.16		-0.06	0.00	0.71	0.77	18.76	1.08
NCU10292	porphobilinogen deaminase	18.67	18.38	19.66	20.19		-0.29	0.53	0.99	1.81		-0.31	0.40	0.71	1.42	3.15	1.40
NCU07240	aflatoxin B1 aldehyde reductase member 2	19.36	19.15	20.33	20.82		-0.21	0.49	0.97	1.67		-0.23	0.36	0.69	1.28	3.41	1.32
NCU05270	mitochondrial translation initiation factor IF-2	16.89	17.02	17.85	18.14		0.13	0.29	0.96	1.12		0.11	0.16	0.68	0.73	5.04	1.04
NCU00680	2-methylcitrate dehydratase	18.99	19.24	19.89	20.00		0.25	0.11	0.90	0.76		0.23	-0.02	0.62	0.37	3.10	0.83
NCU04087	Aha1 domain-containing protein	16.45	16.26	17.30	17.63		-0.19	0.33	0.85	1.37		-0.21	0.20	0.57	0.98	3.94	1.11
NCU04452	menadione-induced gene-3	19.19	18.81	20.01	20.18		-0.38	0.17	0.82	1.37		-0.40	0.04	0.54	0.98	2.82	1.10
NCU01197	gulliver-1	19.35	19.42	20.14	20.11		0.07	-0.03	0.79	0.69		0.05	-0.16	0.51	0.30	3.59	0.74
NCU08436	alpha/beta hydrolase	17.92	17.72	18.71	18.76		-0.20	0.05	0.79	1.04		-0.22	-0.08	0.51	0.65	3.62	0.92
NCU04443	quinone oxidoreductase	18.91	19.08	19.68	19.72		0.17	0.04	0.77	0.64		0.15	-0.09	0.49	0.25	3.14	0.71
NCU05937	GDP-mannose pyrophosphorylase	18.97	18.67	19.73	20.04		-0.30	0.31	0.76	1.37		-0.32	0.18	0.48	0.98	3.00	1.07
NCU04826	hypothetical protein	18.89	18.77	19.65	19.73		-0.12	0.08	0.76	0.96		-0.14	-0.05	0.48	0.57	5.21	0.86
NCU04142	heat shock protein 80	23.46	23.33	24.18	24.59		-0.13	0.41	0.72	1.26		-0.15	0.28	0.44	0.87	3.08	0.99
NCU03857	tricarboxylic acid-5	23.00	22.81	23.69	23.82		-0.19	0.13	0.69	1.01		-0.21	0.00	0.41	0.62	3.63	0.85
NCU04368	glutathione S-transferase Gst3	18.72	18.80	19.36	19.42		0.08	0.06	0.64	0.62		0.06	-0.07	0.36	0.23	4.66	0.63
NCU02193	cellular filament polypeptide	25.53	25.57	26.16	26.20		0.04	0.04	0.63	0.63		0.02	-0.09	0.35	0.24	4.76	0.63
NCU07171	actin-related protein 2	18.86	18.67	19.49	19.62		-0.19	0.13	0.63	0.95		-0.21	0.00	0.35	0.56	3.23	0.79
NCU02982	regulatory particle, non-ATPase-like-10	17.25	17.16	17.86	18.12		-0.09	0.26	0.61	0.96		-0.11	0.13	0.33	0.57	3.90	0.79
NCU06783	ATP citrate lyase	22.70	22.67	23.28	23.31		-0.03	0.03	0.58	0.64		-0.05	-0.10	0.30	0.25	3.68	0.61
NCU08687	galactokinase	15.97	15.89	16.53	16.85		-0.08	0.32	0.56	0.96		-0.10	0.19	0.28	0.57	2.97	0.76
NCU08535	acetyl-CoA carboxylase	20.67	20.53	21.23	21.50		-0.14	0.27	0.56	0.97		-0.16	0.14	0.28	0.58	3.06	0.76
NCU00743	glycogen debranching enzyme	21.16	21.17	21.52	21.64		0.01	0.12	0.36	0.47		-0.01	-0.01	0.08	0.08	12.81	0.41
NCU06300	guanylate kinase	18.63	18.66	18.99	19.15		0.03	0.16	0.36	0.49		0.01	0.03	0.08	0.10	3.71	0.42
NCU06727	spermidine-3	22.70	22.66	22.71	22.82		-0.04	0.11	0.01	0.16		-0.06	-0.02	-0.27	-0.23	5.98	0.09
NCU07914	phosphoglycerate kinase	25.79	25.72	25.76	25.79		-0.07	0.03	-0.03	0.07		-0.09	-0.10	-0.31	-0.32	3.40	0.02
NCU05290	orotate phosphoribosyltransferase	21.09	21.10	21.03	21.09		0.01	0.06	-0.06	-0.01		-0.01	-0.07	-0.34	-0.40	7.73	-0.04
NCU01821	alanine-glyoxylate aminotransferase	20.87	20.90	20.78	20.68		0.03	-0.10	-0.09	-0.22		0.01	-0.23	-0.37	-0.61	3.12	-0.15
NCU00522	cystathionine beta-lyase	20.22	20.34	20.11	20.24		0.12	0.13	-0.11	-0.10		0.10	0.00	-0.39	-0.49	5.97	-0.11
NCU02588	nuclear movement protein nudC	17.48	17.70	17.37	17.44		0.22	0.07	-0.11	-0.26		0.20	-0.06	-0.39	-0.65	3.56	-0.18
NCU04442	GAL10	20.69	20.60	20.56	20.78		-0.09	0.22	-0.13	0.18		-0.11	0.09	-0.41	-0.21	3.23	0.02
NCU07807	fructose bisphosphate aldolase	25.33	25.60	25.18	25.05		0.27	-0.13	-0.15	-0.55		0.25	-0.26	-0.43	-0.94	2.85	-0.35
NCU05095	phenylalanyl-tRNA synthetase subunit alpha	22.03	22.28	21.87	22.05		0.25	0.18	-0.16	-0.23		0.23	0.05	-0.44	-0.62	3.15	-0.20
NCU08162	arginine-10	22.71	22.79	22.54	22.83		0.08	0.29	-0.17	0.04		0.06	0.16	-0.45	-0.35	3.23	-0.07
NCU08964	60S ribosomal protein L10	21.34	21.59	21.16	21.09		0.25	-0.07	-0.18	-0.50		0.23	-0.20	-0.46	-0.89	3.26	-0.34
NCU07451	methionyl-tRNA synthetase	21.75	21.88	21.55	21.74		0.13	0.19	-0.20	-0.14		0.11	0.06	-0.48	-0.53	5.45	-0.17
NCU03826	elongation factor 1-gamma	24.18	24.15	23.93	23.74		-0.03	-0.19	-0.25	-0.41		-0.05	-0.32	-0.53	-0.80	2.98	-0.33
NCU00258	cytoplasmic ribosomal protein-15	22.29	22.34	22.01	22.34		0.05	0.33	-0.28	0.00		0.03	0.20	-0.56	-0.39	3.29	-0.14
NCU04303	asparagine synthetase 2	21.97	22.09	21.69	21.76		0.12	0.07	-0.28	-0.33		0.10	-0.06	-0.56	-0.72	7.65	-0.30
NCU03882	hypothetical protein	18.39	18.73	18.10	18.03		0.34	-0.07	-0.29	-0.70		0.32	-0.20	-0.57	-1.09	3.23	-0.49
NCU03117	inosine-5'-monophosphate dehydrogenase IMD2	21.70	22.10	21.39	21.51		0.40	0.12	-0.31	-0.59		0.38	-0.01	-0.59	-0.98	2.97	-0.45
NCU01528	glyceraldehyde-3-phosphate dehydrogenase-1	26.81	26.82	26.49	26.44		0.01	-0.05	-0.32	-0.38		-0.01	-0.18	-0.60	-0.77	5.48	-0.35
NCU07458	N-acetylglucosamine-phosphate mutase	21.65	21.92	21.33	21.28		0.27	-0.05	-0.32	-0.64		0.25	-0.18	-0.60	-1.03	3.84	-0.48
NCU06251	KH domain RNA-binding protein	19.87	20.10	19.54	19.23		0.23	-0.31	-0.33	-0.87		0.21	-0.44	-0.61	-1.26	2.87	-0.60
NCU10042	embden-meyerhof pathway-7	26.81	26.75	26.44	26.33		-0.06	-0.11	-0.37	-0.42		-0.08	-0.24	-0.65	-0.81	4.15	-0.40
NCU02357	importin subunit beta-3	19.79	19.92	19.41	19.79		0.13	0.38	-0.38	-0.13		0.11	0.25	-0.66	-0.52	3.01	-0.26
NCU03488	pyrimidine-4	22.08	21.90	21.70	21.89		-0.18	0.19	-0.38	-0.01		-0.20	0.06	-0.66	-0.40	3.72	-0.19
NCU02726	ethanolamine kinase	18.43	18.55	18.03	17.84		0.12	-0.19	-0.40	-0.71		0.10	-0.32	-0.68	-1.10	3.86	-0.56
NCU06550	pyridoxine 1	23.84	23.89	23.44	23.63		0.05	0.19	-0.40	-0.26		0.03	0.06	-0.68	-0.65	13.07	-0.33
NCU00489	cytoplasmic ribosomal protein-10	23.31	23.21	22.90	23.16		-0.10	0.26	-0.41	-0.05		-0.12	0.13	-0.69	-0.44	4.61	-0.23
NCU08963	60S ribosomal protein L30	20.48	20.47	20.07	19.84		-0.01	-0.23	-0.41	-0.63		-0.03	-0.36	-0.69	-1.02	3.43	-0.52
NCU02513	YjeF domain-containing protein	19.18	18.95	18.76	18.73		-0.23	-0.03	-0.42	-0.22		-0.25	-0.16	-0.70	-0.61	3.16	-0.32
NCU05498	hypothetical protein	20.52	20.80	20.08	20.16		0.28	0.08	-0.44	-0.64		0.26	-0.05	-0.72	-1.03	4.67	-0.54
NCU08620	40S ribosomal protein S16	22.18	22.49	21.73	21.88		0.31	0.15	-0.45	-0.61		0.29	0.02	-0.73	-1.00	4.18	-0.53
NCU02404	RNP domain-containing protein	19.13	19.31	18.67	18.53		0.18	-0.14	-0.46	-0.78		0.16	-0.27	-0.74	-1.17	4.40	-0.62
NCU06440	proteosome catalytic alpha-4	19.26	19.28	18.80	18.61		0.02	-0.19	-0.46	-0.67		0.00	-0.32	-0.74	-1.06	4.06	-0.57
NCU06346	hypothetical protein	20.84	20.77	20.34	20.40		-0.07	0.06	-0.50	-0.37		-0.09	-0.07	-0.78	-0.76	9.94	-0.44
NCU10020	methionine synthase	19.56	19.67	19.05	19.19		0.11	0.14	-0.51	-0.48		0.09	0.01	-0.79	-0.87	12.33	-0.49
NCU07830	cytoplasmic ribosomal protein-2	22.31	22.59	21.78	22.14		0.28	0.36	-0.53	-0.45		0.26	0.23	-0.81	-0.84	3.30	-0.49
NCU00177	phosphoribosylformylglycinamidine cyclo-ligase	22.78	22.55	22.25	22.48		-0.23	0.23	-0.53	-0.07		-0.25	0.10	-0.81	-0.46	3.47	-0.30
NCU00397	hypothetical protein	18.94	19.13	18.41	18.83		0.19	0.42	-0.53	-0.30		0.17	0.29	-0.81	-0.69	3.11	-0.42
NCU02325	guanine-3	22.66	22.69	22.13	22.32		0.03	0.19	-0.53	-0.37		0.01	0.06	-0.81	-0.76	17.13	-0.45
NCU05129	tryptophan-2	19.27	19.66	18.74	19.01		0.39	0.27	-0.53	-0.65		0.37	0.14	-0.81	-1.04	3.28	-0.59
NCU01669	putative arginine methyltransferase-3	17.29	17.59	16.74	16.75		0.30	0.01	-0.55	-0.84		0.28	-0.12	-0.83	-1.23	4.82	-0.70
NCU03806	cycloheximide resistant-2	22.34	22.27	21.79	22.12		-0.07	0.33	-0.55	-0.15		-0.09	0.20	-0.83	-0.54	4.47	-0.35
NCU03757	60S ribosomal protein L4-A	23.90	23.81	23.34	23.58		-0.09	0.24	-0.56	-0.23		-0.11	0.11	-0.84	-0.62	6.71	-0.40
NCU07308	chain elongation-1	23.08	23.04	22.52	22.64		-0.04	0.12	-0.56	-0.40		-0.06	-0.01	-0.84	-0.79	20.34	-0.48
NCU06301	hypothetical protein	19.48	19.49	18.91	19.32		0.01	0.41	-0.57	-0.17		-0.01	0.28	-0.85	-0.56	3.58	-0.37
NCU02103	DNA-directed RNA polymerase II largest subunit	18.36	18.27	17.77	17.90		-0.09	0.13	-0.59	-0.37		-0.11	0.00	-0.87	-0.76	10.85	-0.48
NCU02829	Sec53-like	21.63	21.51	21.00	21.21		-0.12	0.21	-0.63	-0.30		-0.14	0.08	-0.91	-0.69	7.14	-0.47
NCU10007	acetate utilization-9	18.91	19.20	18.28	18.59		0.29	0.31	-0.63	-0.61		0.27	0.18	-0.91	-1.00	4.09	-0.62
NCU04385	leucine-2	22.48	22.67	21.84	21.85		0.19	0.01	-0.64	-0.82		0.17	-0.12	-0.92	-1.21	7.23	-0.73
NCU04411	tryptophan-4	17.87	18.07	17.23	17.32		0.20	0.09	-0.64	-0.75		0.18	-0.04	-0.92	-1.14	7.78	-0.70
NCU07826	40S ribosomal protein S19	21.80	22.03	21.16	21.20		0.23	0.04	-0.64	-0.83		0.21	-0.09	-0.92	-1.22	6.59	-0.74
NCU09475	40s ribosomal protein s5	22.82	22.80	22.18	22.48		-0.02	0.30	-0.64	-0.32		-0.04	0.17	-0.92	-0.71	6.55	-0.48
NCU08685	phosphoribosylformylglycinamidine synthase	21.81	22.00	21.13	20.82		0.19	-0.31	-0.68	-1.18		0.17	-0.44	-0.96	-1.57	3.90	-0.93
NCU16368	hypothetical protein	17.35	17.01	16.67	16.58		-0.34	-0.09	-0.68	-0.43		-0.36	-0.22	-0.96	-0.82	3.02	-0.56
NCU07307	chain elongation-2	23.26	23.34	22.58	22.91		0.08	0.33	-0.68	-0.43		0.06	0.20	-0.96	-0.82	5.91	-0.56
NCU01195	amination-deficient	26.11	26.25	25.42	25.33		0.14	-0.09	-0.69	-0.92		0.12	-0.22	-0.97	-1.31	6.50	-0.81
NCU00475	40S ribosomal protein S18	22.34	22.44	21.64	21.84		0.10	0.20	-0.70	-0.60		0.08	0.07	-0.98	-0.99	12.47	-0.65
NCU09116	aromatic aminotransferase Aro8	17.54	17.31	16.84	16.81		-0.23	-0.03	-0.70	-0.50		-0.25	-0.16	-0.98	-0.89	4.52	-0.60
NCU03608	isoleucine + valine-2	21.81	21.77	21.09	20.68		-0.04	-0.41	-0.72	-1.09		-0.06	-0.54	-1.00	-1.48	3.30	-0.90
NCU01824	UDP-galactopyranose mutase	22.16	22.35	21.41	20.88		0.19	-0.53	-0.75	-1.47		0.17	-0.66	-1.03	-1.86	3.12	-1.11
NCU08277	eukaryotic translation initiation factor 2 alpha subunit	19.82	19.60	19.07	19.07		-0.22	0.00	-0.75	-0.53		-0.24	-0.13	-1.03	-0.92	5.13	-0.64
NCU07014	cytoplasmic ribosomal protein-3	21.97	21.79	21.21	21.53		-0.18	0.32	-0.76	-0.26		-0.20	0.19	-1.04	-0.65	4.44	-0.51
NCU01439	D-3-phosphoglycerate dehydrogenase 1	23.95	23.97	23.18	23.33		0.02	0.15	-0.77	-0.64		0.00	0.02	-1.05	-1.03	62.79	-0.71
NCU06075	acetate-8	25.01	24.66	24.24	24.48		-0.35	0.24	-0.77	-0.18		-0.37	0.11	-1.05	-0.57	3.11	-0.48
NCU03922	hydroxymethylglutaryl-CoA synthase	21.60	22.09	20.82	20.46		0.49	-0.36	-0.78	-1.63		0.47	-0.49	-1.06	-2.02	3.35	-1.21
NCU09194	nuclease S1	17.57	17.35	16.78	16.61		-0.22	-0.17	-0.79	-0.74		-0.24	-0.30	-1.07	-1.13	4.09	-0.77
NCU07735	Grp1p	22.78	22.59	21.99	21.83		-0.19	-0.16	-0.79	-0.76		-0.21	-0.29	-1.07	-1.15	4.43	-0.78
NCU03010	lysine-3	22.44	22.59	21.64	21.43		0.15	-0.21	-0.80	-1.16		0.13	-0.34	-1.08	-1.55	5.20	-0.98
NCU03118	hypothetical protein	23.75	23.67	22.93	23.08		-0.08	0.15	-0.82	-0.59		-0.10	0.02	-1.10	-0.98	14.87	-0.71
NCU06843	60S ribosomal protein L3	22.80	22.90	21.96	21.89		0.10	-0.07	-0.84	-1.01		0.08	-0.20	-1.12	-1.40	8.35	-0.92
NCU00999	hypothetical protein	15.55	16.08	14.70	14.66		0.53	-0.04	-0.85	-1.42		0.51	-0.17	-1.13	-1.81	3.94	-1.14
NCU05238	cysteine-4	22.93	22.98	22.08	22.47		0.05	0.39	-0.85	-0.51		0.03	0.26	-1.13	-0.90	5.43	-0.68
NCU02274	formate	24.43	24.19	23.57	23.87		-0.24	0.30	-0.86	-0.32		-0.26	0.17	-1.14	-0.71	4.33	-0.59
NCU04449	prolyl-tRNA synthetase	20.85	20.85	19.99	20.38		0.00	0.39	-0.86	-0.47		-0.02	0.26	-1.14	-0.86	5.40	-0.67
NCU00971	ribosomal protein S12	21.97	21.96	21.10	21.59		-0.01	0.49	-0.87	-0.37		-0.03	0.36	-1.15	-0.76	3.77	-0.62
NCU09794	NAD-binding Rossmann fold oxidoreductase	18.27	18.93	17.40	17.51		0.66	0.11	-0.87	-1.42		0.64	-0.02	-1.15	-1.81	3.32	-1.15
NCU02979	AMP deaminase	18.20	18.27	17.32	17.65		0.07	0.33	-0.88	-0.62		0.05	0.20	-1.16	-1.01	7.30	-0.75

NCU01754	alcohol dehydrogenase-1	24.02	24.10	23.13	22.69		0.08	-0.44	-0.89	-1.41		0.06	-0.57	-1.17	-1.80	3.75	-1.15
NCU03425	threonine synthase	22.42	22.38	21.52	21.55		-0.04	0.03	-0.90	-0.83		-0.06	-0.10	-1.18	-1.22	15.11	-0.87
NCU08502	40S ribosomal protein S6	22.67	22.70	21.77	22.30		0.03	0.53	-0.90	-0.40		0.01	0.40	-1.18	-0.79	3.51	-0.65
NCU09896	adenylyl-sulfate kinase	21.41	21.15	20.48	20.50		-0.26	0.02	-0.93	-0.65		-0.28	-0.11	-1.21	-1.04	5.37	-0.79
NCU01985	cysteine-11	24.26	23.93	23.32	23.63		-0.33	0.31	-0.94	-0.30		-0.35	0.18	-1.22	-0.69	3.57	-0.62
NCU08998	4-aminobutyrate aminotransferase	19.46	19.10	18.52	18.82		-0.36	0.30	-0.94	-0.28		-0.38	0.17	-1.22	-0.67	3.35	-0.61
NCU11356	phenazine biosynthesis PhzC/PhzF protein	19.91	20.20	18.96	19.40		0.29	0.44	-0.95	-0.80		0.27	0.31	-1.23	-1.19	4.10	-0.88
NCU07930	cysteine-18	25.49	25.16	24.52	24.32		-0.33	-0.20	-0.97	-0.84		-0.35	-0.33	-1.25	-1.23	3.67	-0.90
NCU06110	thiazole biosynthetic enzyme	26.40	27.05	25.40	25.19		0.65	-0.21	-1.00	-1.86		0.63	-0.34	-1.28	-2.25	3.60	-1.43
NCU09789	adenine-8	21.33	21.36	20.33	20.97		0.03	0.64	-1.00	-0.39		0.01	0.51	-1.28	-0.78	2.91	-0.70
NCU07719	isopentenyl-diphosphate delta-isomerase	20.03	20.29	18.99	19.49		0.26	0.50	-1.04	-0.80		0.24	0.37	-1.32	-1.19	3.98	-0.92
NCU01632	aromatic-1 gene cluster	22.81	22.91	21.75	22.11		0.10	0.36	-1.06	-0.80		0.08	0.23	-1.34	-1.19	7.22	-0.93
NCU07467	hypothetical protein	18.67	18.67	17.61	17.81		0.00	0.20	-1.06	-0.86		-0.02	0.07	-1.34	-1.25	24.45	-0.96
NCU03897	RNA binding effector protein Scp160	18.10	18.46	17.03	17.44		0.36	0.41	-1.07	-1.02		0.34	0.28	-1.35	-1.41	4.37	-1.05
NCU08859	hypothetical protein	16.67	17.22	15.60	15.62		0.55	0.02	-1.07	-1.60		0.53	-0.11	-1.35	-1.99	4.41	-1.34
NCU02571	acetyl-CoA acetyltransferase	22.84	23.54	21.76	22.20		0.70	0.44	-1.08	-1.34		0.68	0.31	-1.36	-1.73	2.92	-1.21
NCU06512	methionine-8	27.15	27.13	26.06	26.07		-0.02	0.01	-1.09	-1.06		-0.04	-0.12	-1.37	-1.45	16.26	-1.08
NCU02533	DNA-directed RNA polymerase I and III polypeptide	16.39	16.67	15.29	14.99		0.28	-0.30	-1.10	-1.68		0.26	-0.43	-1.38	-2.07	4.95	-1.39
NCU09345	no message in thiamine-1	26.91	27.09	25.79	25.83		0.18	0.04	-1.12	-1.26		0.16	-0.09	-1.40	-1.65	11.63	-1.19
NCU01300	imidazoleglycerol-phosphate dehydratase	18.82	19.00	17.69	18.18		0.18	0.49	-1.13	-0.82		0.16	0.36	-1.41	-1.21	4.65	-0.98
NCU05620	proteasome activator subunit 4	16.74	16.21	15.61	15.87		-0.53	0.26	-1.13	-0.34		-0.55	0.13	-1.41	-0.73	2.82	-0.74
NCU00396	pre-mRNA-splicing factor rse-1	17.33	17.09	16.18	15.52		-0.24	-0.66	-1.15	-1.57		-0.26	-0.79	-1.43	-1.96	2.93	-1.36
NCU04292	branched-chain-amino-acid aminotransferase	20.40	20.64	19.25	19.64		0.24	0.39	-1.15	-1.00		0.22	0.26	-1.43	-1.39	5.76	-1.08
NCU00635	nascent polypeptide-associated complex subunit alpha	20.90	20.75	19.72	19.98		-0.15	0.26	-1.18	-0.77		-0.17	0.13	-1.46	-1.16	8.74	-0.98
NCU06417	uracil-5-carboxylate decarboxylase	19.10	18.94	17.92	18.34		-0.16	0.42	-1.18	-0.60		-0.18	0.29	-1.46	-0.99	5.15	-0.89
NCU03973	alanine	19.08	19.47	17.89	18.07		0.39	0.18	-1.19	-1.40		0.37	0.05	-1.47	-1.79	6.12	-1.30
NCU11381	diphosphomevalonate decarboxylase	20.11	20.10	18.90	19.29		-0.01	0.39	-1.21	-0.81		-0.03	0.26	-1.49	-1.20	7.22	-1.01
NCU04799	polyadenylate-binding protein	22.08	21.92	20.85	21.26		-0.16	0.41	-1.23	-0.66		-0.18	0.28	-1.51	-1.05	5.50	-0.94
NCU06459	differentiation regulator	16.11	15.62	14.88	15.10		-0.49	0.22	-1.23	-0.52		-0.51	0.09	-1.51	-0.91	3.42	-0.87
NCU08295	RNA-binding La domain-containing protein	18.29	18.35	17.03	17.34		0.06	0.31	-1.26	-1.01		0.04	0.18	-1.54	-1.40	11.03	-1.14
NCU04640	eukaryotic translation initiation factor 2 beta subunit	19.42	19.78	18.15	18.53		0.36	0.38	-1.27	-1.25		0.34	0.25	-1.55	-1.64	5.27	-1.26
NCU01550	adenylate kinase cytosolic	18.04	18.07	16.71	17.34		0.03	0.63	-1.33	-0.73		0.01	0.50	-1.61	-1.12	3.89	-1.03
NCU08216	cystathionine beta-synthase	21.57	21.25	20.24	20.28		-0.32	0.04	-1.33	-0.97		-0.34	-0.09	-1.61	-1.36	6.06	-1.15
NCU04611	transcription elongation factor spt-6	16.97	17.69	15.63	14.79		0.72	-0.84	-1.34	-2.90		0.70	-0.97	-1.62	-3.29	3.06	-2.12
NCU05485	casein kinase II regulatory beta subunit-1	17.74	17.37	16.35	16.94		-0.37	0.59	-1.39	-0.43		-0.39	0.46	-1.67	-0.82	3.08	-0.91
NCU00692	chaperone dnaK	19.78	19.42	18.39	19.05		-0.36	0.66	-1.39	-0.37		-0.38	0.53	-1.67	-0.76	2.81	-0.88
NCU01571	hypothetical protein	16.01	15.64	14.57	15.09		-0.37	0.52	-1.44	-0.55		-0.39	0.39	-1.72	-0.94	3.55	-1.00
NCU08269	pyridoxine-4	18.72	19.03	17.27	17.98		0.31	0.71	-1.45	-1.05		0.29	0.58	-1.73	-1.44	3.44	-1.25
NCU07267	blue light-induced-3	18.90	18.24	17.37	16.95		-0.66	-0.42	-1.53	-1.29		-0.68	-0.55	-1.81	-1.68	2.83	-1.41
NCU02810	eukaryotic translation initiation factor 2 gamma subunit	20.55	20.83	18.98	19.81		0.28	0.83	-1.57	-1.02		0.26	0.70	-1.85	-1.41	3.09	-1.30
NCU02435	histone H2B	19.96	20.35	18.38	18.30		0.39	-0.08	-1.58	-2.05		0.37	-0.21	-1.86	-2.44	7.17	-1.82
NCU06603	ThiJ/PfpI family protein	21.05	20.64	19.46	19.51		-0.41	0.05	-1.59	-1.13		-0.43	-0.08	-1.87	-1.52	5.56	-1.36
NCU05305	DNA-directed RNA polymerase II polypeptide	16.17	16.28	14.49	15.31		0.11	0.82	-1.68	-0.97		0.09	0.69	-1.96	-1.36	3.40	-1.33
NCU08183	hypothetical protein	18.33	18.93	16.64	16.94		0.60	0.30	-1.69	-1.99		0.58	0.17	-1.97	-2.38	5.06	-1.84
NCU03194	phosphoribosylaminoimidazole carboxylase	21.34	21.07	19.63	20.41		-0.27	0.78	-1.71	-0.66		-0.29	0.65	-1.99	-1.05	3.15	-1.19
NCU07936	NMDA receptor-regulated protein 1	18.93	18.97	17.13	17.29		0.04	0.16	-1.80	-1.68		0.02	0.03	-2.08	-2.07	71.79	-1.74
NCU07774	GTP cyclohydrolase-1	17.33	17.27	15.52	15.21		-0.06	-0.31	-1.81	-2.06		-0.08	-0.44	-2.09	-2.45	7.25	-1.94
NCU02688	ubiquitin-conjugating enzyme E2Z	15.06	14.88	13.22	13.66		-0.18	0.44	-1.84	-1.22		-0.20	0.31	-2.12	-1.61	7.19	-1.53
NCU01428	hydroxyisourate hydrolase	18.66	18.58	16.79	17.00		-0.08	0.21	-1.87	-1.58		-0.10	0.08	-2.15	-1.97	22.94	-1.73
NCU01652	O-acetylhomoserine	21.10	20.80	19.21	19.26		-0.30	0.05	-1.89	-1.54		-0.32	-0.08	-2.17	-1.93	8.91	-1.72
NCU09132	tubulin alpha-1	20.89	20.20	19.00	19.22		-0.69	0.22	-1.89	-0.98		-0.71	0.09	-2.17	-1.37	3.60	-1.44
NCU05512	copper resistance protein Crd2	19.52	20.05	17.60	18.13		0.53	0.53	-1.92	-1.92		0.51	0.40	-2.20	-2.31	4.88	-1.92
NCU05974	cell wall glucanosyltransferase Mwg1	20.14	20.74	18.06	17.56		0.60	-0.50	-2.08	-3.18		0.58	-0.63	-2.36	-3.57	4.99	-2.63
NCU04187	cap binding protein	17.32	16.97	15.23	15.22		-0.35	-0.01	-2.09	-1.75		-0.37	-0.14	-2.37	-2.14	8.16	-1.92
NCU09873	acetate utilization-6	21.33	21.67	19.19	19.96		0.34	0.77	-2.14	-1.71		0.32	0.64	-2.42	-2.10	4.44	-1.93
NCU01438	nucleosome assembly factor-1	22.52	22.38	20.38	21.38		-0.14	1.00	-2.14	-1.00		-0.16	0.87	-2.42	-1.39	3.14	-1.57
NCU07192	hypothetical protein	17.87	17.77	15.72	15.40		-0.10	-0.32	-2.15	-2.37		-0.12	-0.45	-2.43	-2.76	7.95	-2.26
NCU09821	oxidoreductase	17.87	17.56	15.71	16.46		-0.31	0.75	-2.16	-1.10		-0.33	0.62	-2.44	-1.49	4.06	-1.63
NCU07437	eukaryotic translation initiation factor 1A	18.48	18.21	16.27	16.62		-0.27	0.35	-2.21	-1.59		-0.29	0.22	-2.49	-1.98	8.76	-1.90
NCU08767	serine/threonine protein kinase-52	15.62	14.61	13.16	13.38		-1.01	0.22	-2.46	-1.23		-1.03	0.09	-2.74	-1.62	3.08	-1.85
NCU01638	DNA-directed RNA polymerase I subunit RPA1	17.48	16.66	14.88	15.50		-0.82	0.62	-2.60	-1.16		-0.84	0.49	-2.88	-1.55	3.36	-1.88
NCU04720	nitrate nonutilizer-6	19.74	19.77	16.21	13.92		0.03	-2.29	-3.53	-5.85		0.01	-2.42	-3.81	-6.24	3.02	-4.69
NCU11027	zinc metalloprotease	19.83	19.38	16.29	17.59		-0.45	1.30	-3.54	-1.79		-0.47	1.17	-3.82	-2.18	3.48	-2.67
NCU07117	ornithine-N5-oxygenase	19.22	19.85	15.49	15.16		0.63	-0.33	-3.73	-4.69		0.61	-0.46	-4.01	-5.08	8.45	-4.21
NCU02684	chromatin remodelling factor 4-3	20.05	20.10	15.96	17.90		0.05	1.94	-4.09	-2.20		0.03	1.81	-4.37	-2.59	2.80	-3.15
NCU02668	cell wall synthesis protein-Penicillium chrysogenum	18.10	18.09	12.58	13.11		-0.01	0.53	-5.52	-4.98		-0.03	0.40	-5.80	-5.37	19.56	-5.25
NCU00792	branched-chain-amino-acid aminotransferase	21.55	21.21	20.76	21.11		-0.34	0.35	-0.79	-0.10		-0.36	0.22	-1.07	-0.49	2.79	
NCU06211	tricarboxylic acid-16	22.44	22.64	23.24	23.27		0.20	0.03	0.80	0.63		0.18	-0.10	0.52	0.24	2.78	
NCU06052	DnaJ domain-containing protein	14.28	13.45	16.50	16.00		-0.83	-0.50	2.22	2.55		-0.85	-0.63	1.94	2.16	2.76	
NCU03904	hypothetical protein	14.17	15.36	17.30	18.05		1.19	0.75	3.13	2.69		1.17	0.62	2.85	2.30	2.76	
NCU16466	nucleic acid-binding protein	15.92	16.28	17.63	18.54		0.36	0.91	1.71	2.26		0.34	0.78	1.43	1.87	2.76	
NCU05292	DNA damage-inducible protein 1	16.35	15.78	17.37	17.70		-0.57	0.33	1.02	1.92		-0.59	0.20	0.74	1.53	2.75	
NCU02387	nuclear import and export protein Msn5	14.42	15.20	16.71	17.50		0.78	0.79	2.29	2.30		0.76	0.66	2.01	1.91	2.75	
NCU06785	ATP-citrate synthase subunit 1	23.22	23.06	23.72	23.93		-0.16	0.21	0.50	0.87		-0.18	0.08	0.22	0.48	2.74	
NCU00714	heat shock protein STI1	19.29	19.73	20.56	20.83		0.44	0.27	1.27	1.10		0.42	0.14	0.99	0.71	2.73	
NCU04923	glycerol dehydrogenase-1	20.97	21.43	20.66	20.75		0.46	0.09	-0.31	-0.68		0.44	-0.04	-0.59	-1.07	2.73	
NCU08888	phenylalanyl-tRNA synthetase subunit beta	22.70	22.49	22.31	22.58		-0.21	0.27	-0.39	0.09		-0.23	0.14	-0.67	-0.30	2.73	
NCU03944	WD repeat containing protein 2	19.10	19.01	20.39	20.08		-0.09	-0.31	1.29	1.07		-0.11	-0.44	1.01	0.68	2.72	
NCU09097	hypothetical protein	15.53	16.40	14.47	14.81		0.87	0.34	-1.06	-1.59		0.85	0.21	-1.34	-1.98	2.71	
NCU01443	seryl-tRNA synthetase	21.11	21.91	20.11	20.48		0.80	0.37	-1.00	-1.43		0.78	0.24	-1.28	-1.82	2.71	
NCU03748	saccharopine dehydrogenase	22.91	22.82	22.55	22.27		-0.09	-0.28	-0.36	-0.55		-0.11	-0.41	-0.64	-0.94	2.69	
NCU04100	vacuolar sorting protein 1	15.99	16.63	17.70	17.95		0.64	0.25	1.71	1.32		0.62	0.12	1.43	0.93	2.69	
NCU03339	glutathione reductase	20.66	20.38	21.47	21.46		-0.28	-0.01	0.81	1.08		-0.30	-0.14	0.53	0.69	2.68	
NCU02027	acyl-protein thioesterase 1	15.73	16.56	20.13	18.78		0.83	-1.35	4.40	2.22		0.81	-1.48	4.12	1.83	2.68	
NCU07266	diphthine synthase	16.19	16.08	15.18	15.83		-0.11	0.65	-1.01	-0.25		-0.13	0.52	-1.29	-0.64	2.67	
NCU02563	transcription elongation factor S-II	15.87	16.86	14.81	14.09		0.99	-0.72	-1.06	-2.77		0.97	-0.85	-1.34	-3.16	2.66	
NCU08695	hypothetical protein	12.49	13.43	15.22	14.92		0.94	-0.30	2.73	1.49		0.92	-0.43	2.45	1.10	2.64	
NCU09237	KH domain RNA binding protein	17.65	17.63	18.21	18.57		-0.02	0.36	0.56	0.94		-0.04	0.23	0.28	0.55	2.64	
NCU08554	site-specific parvulin-1	15.53	16.25	14.98	14.80		0.72	-0.18	-0.55	-1.45		0.70	-0.31	-0.83	-1.84	2.62	
NCU01100	hypothetical protein	17.47	17.25	15.97	14.86		-0.22	-1.11	-1.50	-2.39		-0.24	-1.24	-1.78	-2.78	2.62	
NCU01756	actin-related protein-3	19.73	19.53	20.33	20.40		-0.20	0.07	0.60	0.87		-0.22	-0.06	0.32	0.48	2.60	
NCU00884	NAD dependent epimerase/dehydratase	20.48	20.33	21.21	21.73		-0.15	0.52	0.73	1.40		-0.17	0.39	0.45	1.01	2.60	
NCU06724	glutamine-1	22.02	22.32	21.38	20.71		0.30	-0.67	-0.64	-1.61		0.28	-0.80	-0.92	-2.00	2.60	
NCU04899	tricarboxylic acid-15	21.52	22.00	22.83	23.03		0.48	0.20	1.31	1.03		0.46	0.07	1.03	0.64	2.59	
NCU04077	cysteine-2	21.82	22.15	21.23	21.70		0.33	0.47	-0.59	-0.45		0.31	0.34	-0.87	-0.84	2.59	
NCU04583	acetyltransferase	19.24	18.83	18.15	18.66		-0.41	0.51	-1.09	-0.17		-0.43	0.38	-1.37	-0.56	2.58	

NCU05429	1,4-alpha-glucan branching enzyme	21.57	21.75	21.33	21.00		0.18	-0.33	-0.24	-0.75		0.16	-0.46	-0.52	-1.14	2.57	
NCU10468	arginine-4	17.01	17.06	18.50	18.09		0.05	-0.41	1.49	1.03		0.03	-0.54	1.21	0.64	2.55	
NCU08500	40S ribosomal protein S8	22.69	21.92	21.10	21.42		-0.77	0.32	-1.59	-0.50		-0.79	0.19	-1.87	-0.89	2.55	
NCU02374	regulatory particle, non-ATPase-like-9	18.33	18.36	19.23	19.08		0.03	-0.15	0.90	0.72		0.01	-0.28	0.62	0.33	2.55	
NCU00554	homoserine-1	22.32	22.57	22.20	21.99		0.25	-0.21	-0.12	-0.58		0.23	-0.34	-0.40	-0.97	2.55	
NCU03290	dipeptidyl peptidase	19.58	19.93	19.06	19.49		0.35	0.43	-0.52	-0.44		0.33	0.30	-0.80	-0.83	2.55	
NCU05516	biotin apo-protein ligase	17.08	17.28	16.01	16.75		0.20	0.74	-1.07	-0.53		0.18	0.61	-1.35	-0.92	2.54	
NCU00935	hypothetical protein	21.82	22.03	21.19	20.51		0.21	-0.68	-0.63	-1.52		0.19	-0.81	-0.91	-1.91	2.54	
NCU02629	adenine-5	22.83	23.32	22.53	22.65		0.49	0.12	-0.30	-0.67		0.47	-0.01	-0.58	-1.06	2.54	
NCU02538	coatomer zeta subunit	17.18	16.92	17.84	18.27		-0.26	0.43	0.66	1.35		-0.28	0.30	0.38	0.96	2.53	
NCU00742	glycerol-3-phosphate dehydrogenase	18.93	18.59	18.26	17.97		-0.34	-0.29	-0.67	-0.62		-0.36	-0.42	-0.95	-1.01	2.52	
NCU04054	Benomyl resistant	22.24	22.07	19.00	20.68		-0.17	1.68	-3.24	-1.39		-0.19	1.55	-3.52	-1.78	2.52	
NCU07738	hypothetical protein	16.16	16.14	17.83	17.32		-0.02	-0.51	1.67	1.18		-0.04	-0.64	1.39	0.79	2.51	
NCU01446	uracil phosphoribosyltransferase	19.02	18.99	20.05	19.83		-0.03	-0.22	1.03	0.84		-0.05	-0.35	0.75	0.45	2.51	
NCU03102	40S ribosomal protein S11	22.15	22.49	22.04	22.16		0.34	0.12	-0.11	-0.33		0.32	-0.01	-0.39	-0.72	2.51	
NCU00457	translation initiation factor 4B	17.62	17.49	16.51	17.23		-0.13	0.72	-1.11	-0.26		-0.15	0.59	-1.39	-0.65	2.50	
NCU07926	glutaminyl-tRNA synthetase	20.39	20.63	20.27	20.04		0.24	-0.23	-0.12	-0.59		0.22	-0.36	-0.40	-0.98	2.50	
NCU05999	CaaX farnesyltransferase beta subunit Ram1	14.71	14.99	15.57	15.83		0.28	0.26	0.86	0.84		0.26	0.13	0.58	0.45	2.50	
NCU05363	26S protease regulatory subunit 8	18.38	18.08	19.07	19.15		-0.30	0.08	0.69	1.07		-0.32	-0.05	0.41	0.68	2.50	
NCU11292	hypothetical protein	16.73	15.39	19.31	18.75		-1.34	-0.56	2.58	3.36		-1.36	-0.69	2.30	2.97	2.47	
NCU02744	60S ribosomal protein L9	21.12	21.38	21.01	21.24		0.26	0.23	-0.11	-0.14		0.24	0.10	-0.39	-0.53	2.47	
NCU08374	hypothetical protein	13.42	14.33	16.18	15.69		0.91	-0.49	2.76	1.36		0.89	-0.62	2.48	0.97	2.46	
NCU04317	DUF431 domain-containing protein	16.92	16.93	18.42	17.98		0.01	-0.44	1.50	1.05		-0.01	-0.57	1.22	0.66	2.45	
NCU01283	hypothetical protein	18.83	19.33	18.22	17.50		0.50	-0.72	-0.61	-1.83		0.48	-0.85	-0.89	-2.22	2.45	
NCU06310	exopolyphosphatase	15.75	15.05	14.23	14.70		-0.70	0.47	-1.52	-0.35		-0.72	0.34	-1.80	-0.74	2.45	
NCU04843	hypothetical protein	19.77	19.91	18.61	17.43		0.14	-1.18	-1.16	-2.48		0.12	-1.31	-1.44	-2.87	2.44	
NCU06914	histidyl-tRNA synthetase	20.59	20.30	19.20	20.00		-0.29	0.80	-1.39	-0.30		-0.31	0.67	-1.67	-0.69	2.44	
NCU10029	peptide methionine sulfoxide reductase msrA	17.13	17.49	16.98	17.15		0.36	0.17	-0.15	-0.34		0.34	0.04	-0.43	-0.73	2.43	
NCU00573	hypothetical protein	16.19	16.62	18.26	17.71		0.43	-0.55	2.07	1.09		0.41	-0.68	1.79	0.70	2.43	
NCU04410	tRNA ligase	18.56	18.71	14.46	16.65		0.15	2.19	-4.10	-2.06		0.13	2.06	-4.38	-2.45	2.43	
NCU11195	D-isomer specific 2-hydroxyacid dehydrogenase	15.99	16.40	18.20	17.56		0.41	-0.64	2.21	1.16		0.39	-0.77	1.93	0.77	2.42	
NCU05426	WD repeat protein	15.86	15.17	14.59	14.25		-0.69	-0.34	-1.27	-0.92		-0.71	-0.47	-1.55	-1.31	2.39	
NCU06717	arsenical pump-driving ATPase	17.11	16.80	17.73	18.11		-0.31	0.38	0.62	1.31		-0.33	0.25	0.34	0.92	2.39	
NCU03992	fimbrin	17.75	16.94	19.86	19.24		-0.81	-0.62	2.11	2.30		-0.83	-0.75	1.83	1.91	2.38	
NCU08280	squalene epoxidase	14.23	14.08	13.39	14.00		-0.15	0.61	-0.84	-0.08		-0.17	0.48	-1.12	-0.47	2.37	
NCU04331	cytoplasmic ribosomal protein-4	22.48	23.21	22.07	21.94		0.73	-0.13	-0.41	-1.27		0.71	-0.26	-0.69	-1.66	2.36	
NCU06945	hypothetical protein	16.40	17.05	18.95	18.27		0.65	-0.68	2.55	1.22		0.63	-0.81	2.27	0.83	2.36	
NCU05525	DUF833 domain-containing protein	17.49	16.64	15.93	15.56		-0.85	-0.37	-1.56	-1.08		-0.87	-0.50	-1.84	-1.47	2.35	
NCU01317	60S ribosomal protein L12	21.21	20.81	20.62	20.74		-0.40	0.12	-0.59	-0.07		-0.42	-0.01	-0.87	-0.46	2.35	
NCU01965	cytochrome-20	21.93	22.48	21.69	21.67		0.55	-0.02	-0.24	-0.81		0.53	-0.15	-0.52	-1.20	2.35	
NCU03368	hypothetical protein	18.14	18.00	17.91	17.71		-0.14	-0.20	-0.23	-0.29		-0.16	-0.33	-0.51	-0.68	2.33	
NCU00537	hypothetical protein	14.44	14.47	15.50	15.25		0.03	-0.25	1.06	0.78		0.01	-0.38	0.78	0.39	2.32	
NCU08499	GTPase-activating protein GYP5	14.63	15.64	17.38	16.95		1.01	-0.43	2.75	1.31		0.99	-0.56	2.47	0.92	2.32	
NCU02380	threonyl-tRNA synthetase	20.00	19.69	20.58	20.90		-0.31	0.32	0.58	1.21		-0.33	0.19	0.30	0.82	2.32	
NCU01784	pseudouridylate synthase 3	13.43	13.82	14.77	15.54		0.39	0.77	1.34	1.72		0.37	0.64	1.06	1.33	2.29	
NCU11288	xaa-Pro dipeptidase	18.61	18.77	18.75	18.77		0.16	0.02	0.14	0.00		0.14	-0.11	-0.14	-0.39	2.29	
NCU09817	aromatic-7	19.41	19.07	20.27	21.05		-0.34	0.78	0.86	1.98		-0.36	0.65	0.58	1.59	2.28	
NCU00122	aspartyl aminopeptidase	18.75	18.31	19.69	20.55		-0.44	0.86	0.94	2.24		-0.46	0.73	0.66	1.85	2.28	
NCU04104	chromosome segregation protein Cse1	18.98	19.35	20.28	20.09		0.37	-0.19	1.30	0.74		0.35	-0.32	1.02	0.35	2.28	
NCU11353	D-xylulose kinase	17.28	16.18	14.50	15.51		-1.10	1.01	-2.78	-0.67		-1.12	0.88	-3.06	-1.06	2.27	
NCU04647	actin binding protein	15.33	14.71	14.29	14.49		-0.62	0.20	-1.04	-0.22		-0.64	0.07	-1.32	-0.61	2.26	
NCU04635	hypothetical protein	16.02	16.47	15.39	14.47		0.45	-0.92	-0.63	-2.00		0.43	-1.05	-0.91	-2.39	2.25	
NCU08923	zinc knuckle domain-containing protein	14.57	14.17	18.25	16.65		-0.40	-1.60	3.68	2.48		-0.42	-1.73	3.40	2.09	2.25	
NCU03023	phenol 2-monooxygenase	19.42	19.79	19.33	19.09		0.37	-0.24	-0.09	-0.70		0.35	-0.37	-0.37	-1.09	2.25	
NCU01175	farnesyl-pyrophosphate synthetase	22.36	22.75	22.18	22.40		0.39	0.22	-0.18	-0.35		0.37	0.09	-0.46	-0.74	2.25	
NCU08022	pol	15.02	14.96	15.81	16.49		-0.06	0.68	0.79	1.53		-0.08	0.55	0.51	1.14	2.25	
NCU00843	adenine-9	20.60	21.40	20.01	20.24		0.80	0.23	-0.59	-1.16		0.78	0.10	-0.87	-1.55	2.24	
NCU06149	ATP-dependent RNA helicase dhh-1	14.79	15.92	17.35	17.35		1.13	0.00	2.56	1.43		1.11	-0.13	2.28	1.04	2.24	
NCU03258	oxysterol-binding protein	16.88	15.79	18.72	18.37		-1.09	-0.35	1.84	2.58		-1.11	-0.48	1.56	2.19	2.24	
NCU06448	enoyl-CoA hydratase	14.88	15.11	17.29	16.40		0.23	-0.89	2.41	1.29		0.21	-1.02	2.13	0.90	2.23	
NCU02905	60S ribosomal protein L23	19.48	19.85	20.45	20.63		0.37	0.18	0.97	0.78		0.35	0.05	0.69	0.39	2.23	
NCU03565	ribosomal protein L26	20.97	20.88	19.52	20.49		-0.09	0.97	-1.45	-0.39		-0.11	0.84	-1.73	-0.78	2.23	
NCU00823	regulatory particle, non-ATPase-like-11	17.27	16.64	18.75	18.38		-0.63	-0.37	1.48	1.74		-0.65	-0.50	1.20	1.35	2.22	
NCU06397	profilin	21.54	21.83	21.52	21.68		0.29	0.16	-0.02	-0.15		0.27	0.03	-0.30	-0.54	2.22	
NCU07156	histidine-6	20.41	20.69	20.41	20.56		0.28	0.15	0.00	-0.13		0.26	0.02	-0.28	-0.52	2.21	
NCU08959	hypothetical protein	16.63	15.80	17.60	18.09		-0.83	0.49	0.97	2.29		-0.85	0.36	0.69	1.90	2.20	
NCU08550	hypothetical protein	18.49	18.03	17.62	17.12		-0.46	-0.50	-0.87	-0.91		-0.48	-0.63	-1.15	-1.30	2.20	
NCU16651	dihydrofolate reductase	15.77	15.10	14.72	14.61		-0.67	-0.11	-1.05	-0.49		-0.69	-0.24	-1.33	-0.88	2.19	
NCU04370	ubiquitin-activating enzyme E1 1	20.54	20.54	21.32	21.98		0.00	0.66	0.78	1.44		-0.02	0.53	0.50	1.05	2.19	
NCU07700	colonial temperature-sensitive-3	23.35	22.83	24.57	24.31		-0.52	-0.26	1.22	1.48		-0.54	-0.39	0.94	1.09	2.18	
NCU05683	dihydroxy-acid dehydratase	15.94	16.49	15.27	14.22		0.55	-1.05	-0.67	-2.27		0.53	-1.18	-0.95	-2.66	2.18	
NCU08409	tryptophan-3	19.81	19.56	20.41	20.93		-0.25	0.52	0.60	1.37		-0.27	0.39	0.32	0.98	2.18	
NCU07697	tricarboxylic acid-4	18.63	19.06	20.79	20.10		0.43	-0.69	2.16	1.04		0.41	-0.82	1.88	0.65	2.18	
NCU09425	NdvB protein	15.60	15.76	16.41	16.96		0.16	0.55	0.81	1.20		0.14	0.42	0.53	0.81	2.18	
NCU03982	glucose regulated protein 78	16.93	16.24	15.85	15.74		-0.69	-0.11	-1.08	-0.50		-0.71	-0.24	-1.36	-0.89	2.18	
NCU10051	flavohemoglobin	24.48	24.40	24.52	24.51		-0.08	-0.01	0.04	0.11		-0.10	-0.14	-0.24	-0.28	2.18	
NCU09468	tubulin alpha-2	21.49	21.06	18.21	20.04		-0.43	1.83	-3.28	-1.02		-0.45	1.70	-3.56	-1.41	2.17	
NCU09285	menadione-induced gene-6	23.97	23.93	25.24	24.85		-0.04	-0.39	1.27	0.92		-0.06	-0.52	0.99	0.53	2.17	
NCU04924	hypothetical protein similar to phosphatidyl synthase	19.98	20.17	19.38	18.48		0.19	-0.90	-0.60	-1.69		0.17	-1.03	-0.88	-2.08	2.16	
NCU06666	inositol	24.38	24.63	24.44	24.56		0.25	0.12	0.06	-0.07		0.23	-0.01	-0.22	-0.46	2.16	
NCU06307	multisynthetase complex auxiliary component p43	21.08	21.25	21.16	21.04		0.17	-0.12	0.08	-0.21		0.15	-0.25	-0.20	-0.60	2.16	
NCU03038	40S ribosomal protein S13	22.08	21.86	21.26	21.87		-0.22	0.61	-0.82	0.01		-0.24	0.48	-1.10	-0.38	2.16	
NCU07941	aspartate aminotransferase	19.53	19.56	19.47	19.23		0.03	-0.24	-0.06	-0.33		0.01	-0.37	-0.34	-0.72	2.16	
NCU02136	transaldolase	25.24	25.55	25.17	25.38		0.31	0.21	-0.07	-0.17		0.29	0.08	-0.35	-0.56	2.15	
NCU09041	L-xylulose reductase	20.71	20.46	19.97	19.26		-0.25	-0.71	-0.74	-1.20		-0.27	-0.84	-1.02	-1.59	2.15	
NCU01596	regulatory particle, non-ATPase-like-6	18.01	18.46	19.25	19.18		0.45	-0.07	1.24	0.72		0.43	-0.20	0.96	0.33	2.14	
NCU08886	amidohydrolase	18.11	18.01	17.62	17.01		-0.10	-0.61	-0.49	-1.00		-0.12	-0.74	-0.77	-1.39	2.12	
NCU00102	hypothetical protein	18.39	17.61	19.61	19.49		-0.78	-0.12	1.22	1.88		-0.80	-0.25	0.94	1.49	2.12	
NCU07922	elongation factor 3	22.66	23.16	23.94	23.91		0.50	-0.03	1.28	0.75		0.48	-0.16	1.00	0.36	2.10	
NCU07590	Ser/Thr protein phosphatase	15.67	15.91	15.65	15.37		0.24	-0.28	-0.02	-0.54		0.22	-0.41	-0.30	-0.93	2.09	
NCU09076	ATP-dependent RNA helicase	19.91	19.36	21.00	20.82		-0.55	-0.18	1.09	1.46		-0.57	-0.31	0.81	1.07	2.09	
NCU12152	hypothetical protein	15.64	15.26	16.65	17.79		-0.38	1.14	1.01	2.53		-0.40	1.01	0.73	2.14	2.08	
NCU00575	glucokinase	21.90	22.20	21.96	22.02		0.30	0.06	0.06	-0.18		0.28	-0.07	-0.22	-0.57	2.08	
NCU02785	aromatic-8	21.08	20.29	19.66	20.10		-0.79	0.44	-1.42	-0.19		-0.81	0.31	-1.70	-0.58	2.07	
NCU05548	aromatic-6	19.57	19.53	19.98	20.29		-0.04	0.31	0.41	0.76		-0.06	0.18	0.13	0.37	2.07	
NCU04109	glutathione S-transferase-2	18.02	17.99	19.00	19.97		-0.03	0.97	0.98	1.98		-0.05	0.84	0.70	1.59	2.06	
NCU02414	translation initiation factor eif-2b epsilon subunit	18.85	18.50	17.64	18.43		-0.35	0.79	-1.21	-0.07		-0.37	0.66	-1.49	-0.46	2.05	
NCU09463	leucine-6	23.01	23.24	22.77	23.16		0.23	0.39	-0.24	-0.08		0.21	0.26	-0.52	-0.47	2.05	
NCU08824	molybdopterin binding domain-containing protein	14.35	15.05	16.71	16.03		0.70	-0.68	2.36	0.98		0.68	-0.81	2.08	0.59	2.05	

NCU06678	exonuclease Kem1	16.13	15.43	16.91	17.57		-0.70	0.66	0.78	2.14		-0.72	0.53	0.50	1.75	2.04
NCU01419	quinone oxidoreductase	14.81	13.91	15.71	16.42		-0.90	0.71	0.90	2.51		-0.92	0.58	0.62	2.12	2.04
NCU07024	osmotic sensitive-2	18.30	17.63	19.46	19.27		-0.67	-0.19	1.16	1.64		-0.69	-0.32	0.88	1.25	2.03
NCU02797	UTP-glucose-1-phosphate uridylyltransferase	23.35	23.49	22.65	23.32		0.14	0.67	-0.70	-0.17		0.12	0.54	-0.98	-0.56	2.02
NCU00225	hypothetical protein	21.50	22.04	21.29	21.48		0.54	0.19	-0.21	-0.56		0.52	0.06	-0.49	-0.95	2.02
NCU07420	eIF4A	22.96	22.94	23.13	23.17		-0.02	0.04	0.17	0.23		-0.04	-0.09	-0.11	-0.16	2.01
NCU05274	eukaryotic initiation factor 5A	21.32	20.39	19.81	20.07		-0.93	0.26	-1.51	-0.32		-0.95	0.13	-1.79	-0.71	2.01
NCU08195	arginyl-tRNA synthetase	20.62	21.28	20.28	20.51		0.66	0.23	-0.34	-0.77		0.64	0.10	-0.62	-1.16	2.01
NCU07446	vacuolar membrane ATPase-4	18.09	17.24	19.08	19.21		-0.85	0.13	0.99	1.97		-0.87	0.00	0.71	1.58	2.01
NCU09239	proliferating cell nuclear antigen	18.56	19.28	18.06	18.41		0.72	0.35	-0.50	-0.87		0.70	0.22	-0.78	-1.26	2.00
NCU07853	uricase	21.14	21.39	20.90	20.26		0.25	-0.64	-0.24	-1.13		0.23	-0.77	-0.52	-1.52	2.00
NCU06416	thymine dioxygenase	19.03	19.02	17.28	14.95		-0.01	-2.33	-1.75	-4.07		-0.03	-2.46	-2.03	-4.46	1.99
NCU04414	regulatory particle, ATPase-like-5	19.15	18.70	19.98	19.92		-0.45	-0.06	0.83	1.22		-0.47	-0.19	0.55	0.83	1.99
NCU08931	merozoite capping protein-1	16.68	16.95	17.79	18.66		0.27	0.87	1.11	1.71		0.25	0.74	0.83	1.32	1.99
NCU09841	phosphotyrosine protein phosphatase	17.44	16.30	14.35	15.79		-1.14	1.44	-3.09	-0.51		-1.16	1.31	-3.37	-0.90	1.99
NCU00634	ribosomal protein L14	22.17	21.47	20.71	21.40		-0.70	0.69	-1.46	-0.07		-0.72	0.56	-1.74	-0.46	1.97
NCU07690	methylenetetrahydrofolate reductase 1	20.94	20.63	20.36	19.81		-0.31	-0.55	-0.58	-0.82		-0.33	-0.68	-0.86	-1.21	1.97
NCU03548	hypothetical protein	18.38	18.53	15.77	17.52		0.15	1.75	-2.61	-1.01		0.13	1.62	-2.89	-1.40	1.97
NCU00472	CDC37	17.78	17.34	16.91	17.43		-0.44	0.52	-0.87	0.09		-0.46	0.39	-1.15	-0.30	1.97
NCU01224	regulatory particle, ATPase-like-2	18.83	18.84	19.31	19.70		0.01	0.39	0.48	0.86		-0.01	0.26	0.20	0.47	1.96
NCU00018	cell division control protein Cdc48	23.22	23.11	23.27	23.40		-0.11	0.13	0.05	0.29		-0.13	0.00	-0.23	-0.10	1.95
NCU01759	menadione-induced gene-5	16.27	16.98	18.61	17.89		0.71	-0.72	2.34	0.91		0.69	-0.85	2.06	0.52	1.94
NCU07721	regulatory particle, non-ATPase-like-1	20.18	20.07	20.53	20.72		-0.11	0.19	0.35	0.65		-0.13	0.06	0.07	0.26	1.94
NCU05716	hypothetical protein	12.93	14.07	15.18	15.36		1.14	0.18	2.25	1.29		1.12	0.05	1.97	0.90	1.93
NCU06311	GMP synthase	18.36	18.60	15.94	17.62		0.24	1.68	-2.42	-0.98		0.22	1.55	-2.70	-1.37	1.93
NCU00187	menadione-induced gene-11	17.53	17.50	16.23	14.34		-0.03	-1.89	-1.30	-3.16		-0.05	-2.02	-1.58	-3.55	1.92
NCU02252	embden-meyerhof pathway-6	24.75	24.85	24.72	24.38		0.10	-0.34	-0.03	-0.47		0.08	-0.47	-0.31	-0.86	1.92
NCU04460	galactose-1-phosphate uridylyltransferase	18.11	17.86	16.65	17.69		-0.25	1.04	-1.46	-0.17		-0.27	0.91	-1.74	-0.56	1.92
NCU00979	60S acidic ribosomal protein P2	21.77	21.06	20.75	20.93		-0.71	0.18	-1.02	-0.13		-0.73	0.05	-1.30	-0.52	1.92
NCU03635	60S ribosomal protein L38	20.12	19.88	19.51	20.04		-0.24	0.53	-0.61	0.16		-0.26	0.40	-0.89	-0.23	1.92
NCU00903	hypothetical protein	18.18	17.55	18.82	19.36		-0.63	0.54	0.64	1.81		-0.65	0.41	0.36	1.42	1.92
NCU07947	glycolipid transfer protein HET-C2	21.25	21.24	20.88	20.18		-0.01	-0.70	-0.37	-1.06		-0.03	-0.83	-0.65	-1.45	1.92
NCU02044	GTP-binding protein	21.42	21.72	19.80	21.03		0.30	1.23	-1.62	-0.69		0.28	1.10	-1.90	-1.08	1.91
NCU06191	glutathione synthetase large subunit	18.07	17.93	17.90	18.21		-0.14	0.31	-0.17	0.28		-0.16	0.18	-0.45	-0.11	1.91
NCU09600	dienelactone hydrolase	18.49	18.70	19.53	19.29		0.21	-0.24	1.04	0.59		0.19	-0.37	0.76	0.20	1.90
NCU02677	arginine-3	19.49	19.67	19.38	18.87		0.18	-0.51	-0.11	-0.80		0.16	-0.64	-0.39	-1.19	1.90
NCU06970	ribose-phosphate pyrophosphokinase II	17.16	17.22	17.09	17.42		0.06	0.33	-0.07	0.20		0.04	0.20	-0.35	-0.19	1.89
NCU04228	phytanoyl-CoA dioxygenase	15.47	16.75	17.91	18.56		1.28	0.65	2.44	1.81		1.26	0.52	2.16	1.42	1.89
NCU01949	unknown-16	22.35	22.18	21.20	22.10		-0.17	0.90	-1.15	-0.08		-0.19	0.77	-1.43	-0.47	1.89
NCU06712	proteasome catalytic alpha-6	18.63	18.42	18.52	18.62		-0.21	0.10	-0.11	0.20		-0.23	-0.03	-0.39	-0.19	1.88
NCU06108	UPF0135 protein	16.59	17.27	19.00	18.17		0.68	-0.83	2.41	0.90		0.66	-0.96	2.13	0.51	1.88
NCU06941	prefoldin subunit 6	16.30	15.51	14.79	15.47		-0.79	0.68	-1.51	-0.04		-0.81	0.55	-1.79	-0.43	1.88
NCU05800	zinc knuckle domain-containing protein	18.17	18.66	17.07	17.99		0.49	0.92	-1.10	-0.67		0.47	0.79	-1.38	-1.06	1.88
NCU03877	C-1-tetrahydrofolate synthase	18.99	19.18	20.28	19.87		0.19	-0.41	1.29	0.69		0.17	-0.54	1.01	0.30	1.87
NCU08332	hexagonal-1	18.60	18.57	19.62	19.31		-0.03	-0.31	1.02	0.74		-0.05	-0.44	0.74	0.35	1.87
NCU04511	bud site selection protein 7	17.42	17.01	18.05	18.12		-0.41	0.07	0.63	1.11		-0.43	-0.06	0.35	0.72	1.87
NCU02011	importin subunit beta-1	18.61	18.19	19.17	19.82		-0.42	0.65	0.56	1.63		-0.44	0.52	0.28	1.24	1.87
NCU07589	acetyltransferase	18.63	18.67	17.32	18.37		0.04	1.05	-1.31	-0.30		0.02	0.92	-1.59	-0.69	1.87
NCU00405	glycyl-tRNA synthetase 1	22.59	22.74	21.95	22.62		0.15	0.67	-0.64	-0.12		0.13	0.54	-0.92	-0.51	1.87
NCU04553	ubiquitin/cytoplasmic ribosomal protein-6	20.54	20.94	20.46	20.68		0.40	0.22	-0.08	-0.26		0.38	0.09	-0.36	-0.65	1.87
NCU09141	menadione-induced gene-9	18.71	18.31	19.34	19.40		-0.40	0.06	0.63	1.09		-0.42	-0.07	0.35	0.70	1.87
NCU02815	osmotic-1	16.05	15.69	19.03	17.55		-0.36	-1.48	2.98	1.86		-0.38	-1.61	2.70	1.47	1.87
NCU02208	unknown-10	21.47	21.13	21.14	21.09		-0.34	-0.05	-0.33	-0.04		-0.36	-0.18	-0.61	-0.43	1.86
NCU00963	methylenetetrahydrofolate dehydrogenase	20.39	20.39	18.51	19.89		0.00	1.38	-1.88	-0.50		-0.02	1.25	-2.16	-0.89	1.86
NCU08477	ypt-like-1	16.80	16.82	17.81	17.51		0.02	-0.30	1.01	0.69		0.00	-0.43	0.73	0.30	1.86
NCU01547	regulatory particle, non-ATPase-like-8	19.19	18.92	19.65	20.13		-0.27	0.48	0.46	1.21		-0.29	0.35	0.18	0.82	1.86
NCU03963	nicotinic acid-7	18.30	17.81	17.73	17.72		-0.49	-0.01	-0.57	-0.09		-0.51	-0.14	-0.85	-0.48	1.85
NCU04289	hypothetical protein	18.44	17.89	17.20	17.95		-0.55	0.75	-1.24	0.06		-0.57	0.62	-1.52	-0.33	1.84
NCU02639	arginine-1	23.34	23.81	23.39	23.29		0.47	-0.10	0.05	-0.52		0.45	-0.23	-0.23	-0.91	1.84
NCU08434	5-methyltetrahydropteroyltriglutamate-homocysteine methyltransferase	19.56	19.87	21.07	20.57		0.31	-0.50	1.51	0.70		0.29	-0.63	1.23	0.31	1.84
NCU00673	serine protease p2	20.05	19.60	19.55	19.50		-0.45	-0.05	-0.50	-0.10		-0.47	-0.18	-0.78	-0.49	1.84
NCU03935	homoserine dehydrogenase	21.48	21.83	21.58	21.39		0.35	-0.19	0.10	-0.44		0.33	-0.32	-0.18	-0.83	1.84
NCU00294	60S ribosomal protein L10a	22.13	21.60	20.77	21.63		-0.53	0.86	-1.36	0.03		-0.55	0.73	-1.64	-0.36	1.83
NCU08828	fatty acid oxidation-2	15.50	15.11	16.42	16.21		-0.39	-0.21	0.92	1.10		-0.41	-0.34	0.64	0.71	1.82
NCU02948	non-anchored cell wall protein-4	21.91	21.58	21.37	21.80		-0.33	0.43	-0.54	0.22		-0.35	0.30	-0.82	-0.17	1.81
NCU01468	translation initiation factor eIF-2B subunit delta	17.37	17.50	16.97	17.51		0.13	0.54	-0.40	0.01		0.11	0.41	-0.68	-0.38	1.81
NCU03575	isoleucyl-tRNA synthetase	22.45	22.68	22.12	22.61		0.23	0.49	-0.33	-0.07		0.21	0.36	-0.61	-0.46	1.80
NCU00520	oxidoreductase	20.59	20.45	14.64	18.47		-0.14	3.83	-5.95	-1.98		-0.16	3.70	-6.23	-2.37	1.80
NCU00043	protein phosphatase-3	19.21	19.55	17.77	18.95		0.34	1.18	-1.44	-0.60		0.32	1.05	-1.72	-0.99	1.80
NCU00618	40S ribosomal protein S27	19.75	20.11	19.57	19.92		0.36	0.35	-0.18	-0.19		0.34	0.22	-0.46	-0.58	1.79
NCU03399	hypothetical protein	15.79	16.02	17.11	18.55		0.23	1.44	1.32	2.53		0.21	1.31	1.04	2.14	1.79
NCU03911	F-actin-capping protein subunit alpha	16.95	18.02	15.99	16.67		1.07	0.68	-0.96	-1.35		1.05	0.55	-1.24	-1.74	1.79
NCU00106	proline-4	14.33	15.07	15.87	16.65		0.74	0.78	1.54	1.58		0.72	0.65	1.26	1.19	1.78
NCU08954	hypothetical protein	18.28	18.68	17.15	14.77		0.40	-2.38	-1.13	-3.91		0.38	-2.51	-1.41	-4.30	1.78
NCU07408	phosphoprotein P0	22.03	21.61	20.94	21.72		-0.42	0.78	-1.09	0.11		-0.44	0.65	-1.37	-0.28	1.78
NCU03781	CobW domain-containing protein	15.40	14.81	17.66	16.57		-0.59	-1.09	2.26	1.76		-0.61	-1.22	1.98	1.37	1.77
NCU01611	carnitine acetyl transferase	14.50	15.49	14.04	14.30		0.99	0.26	-0.46	-1.19		0.97	0.13	-0.74	-1.58	1.77
NCU00236	flavoprotein oxygenase	13.88	14.18	14.90	15.78		0.30	0.88	1.02	1.60		0.28	0.75	0.74	1.21	1.77
NCU03634	hypothetical protein	15.90	17.15	18.59	18.06		1.25	-0.53	2.69	0.91		1.23	-0.66	2.41	0.52	1.77
NCU01234	eukaryotic initiation factor 4A-12	15.13	15.27	16.71	16.07		0.14	-0.64	1.58	0.80		0.12	-0.77	1.30	0.41	1.76
NCU11370	6-phosphogluconate dehydrogenase 2	17.47	17.68	18.25	18.89		0.21	0.64	0.78	1.21		0.19	0.51	0.50	0.82	1.76
NCU06940	hypothetical protein	16.10	14.66	17.47	17.39		-1.44	-0.08	1.37	2.73		-1.46	-0.21	1.09	2.34	1.76
NCU01121	hypothetical protein	17.07	16.54	17.57	18.13		-0.53	0.56	0.50	1.59		-0.55	0.43	0.22	1.20	1.76
NCU02226	methylthioribose-1-phosphate isomerase	17.14	16.47	17.77	18.03		-0.67	0.26	0.63	1.56		-0.69	0.13	0.35	1.17	1.75
NCU04779	60S ribosomal protein L8	22.28	21.72	21.62	21.42		-0.56	-0.20	-0.66	-0.30		-0.58	-0.33	-0.94	-0.69	1.75
NCU06693	hypothetical protein	15.01	14.48	15.67	16.75		-0.53	1.08	0.66	2.27		-0.55	0.95	0.38	1.88	1.75
NCU05400	hypothetical protein	15.95	15.61	16.42	17.05		-0.34	0.63	0.47	1.44		-0.36	0.50	0.19	1.05	1.74
NCU02284	nucleolar ATPase Kre33	16.48	15.80	14.08	15.62		-0.68	1.54	-2.40	-0.18		-0.70	1.41	-2.68	-0.57	1.74
NCU05599	40S ribosomal protein S28	18.48	18.63	17.77	18.52		0.15	0.75	-0.71	-0.11		0.13	0.62	-0.99	-0.50	1.73
NCU08070	meiotically up-regulated 182 protein	15.59	14.80	16.17	16.92		-0.79	0.75	0.58	2.12		-0.81	0.62	0.30	1.73	1.73
NCU09040	menadione-induced gene-4	20.31	20.17	19.83	18.93		-0.14	-0.90	-0.48	-1.24		-0.16	-1.03	-0.76	-1.63	1.73
NCU02328	O-methyltransferase	16.73	16.11	18.16	17.61		-0.62	-0.55	1.43	1.50		-0.64	-0.68	1.15	1.11	1.73
NCU04230	acetate utilization-3	18.10	19.16	17.24	17.89		1.06	0.65	-0.86	-1.27		1.04	0.52	-1.14	-1.66	1.72
NCU09861	Ser/Thr protein phosphatase	18.30	17.97	19.04	18.92		-0.33	-0.12	0.74	0.95		-0.35	-0.25	0.46	0.56	1.72
NCU01744	enhancer-2 of am	20.63	20.65	20.53	20.03		0.02	-0.50	-0.10	-0.62		0.00	-0.63	-0.38	-1.01	1.71
NCU06452	cysteine-17	14.89	16.08	17.31	16.92		1.19	-0.39	2.42	0.84		1.17	-0.52	2.14	0.45	1.71
NCU08875	Cullin binding protein CanA	16.52	15.87	17.03	17.63		-0.65	0.60	0.51	1.76		-0.67	0.47	0.23	1.37	1.71
NCU01546	coproporphyrinogen III oxidase	21.22	21.83	21.25	20.67		0.61	-0.58	0.03	-1.16		0.59	-0.71	-0.25	-1.55	1.70
NCU02391	protein transporter sec-24	15.62	14.28	16.38	17.21		-1.34	0.83	0.76	2.93		-1.36	0.70	0.48	2.54	1.69

NCU07866	DEAD helicase superfamily protein	13.09	14.03	16.99	15.14	0.94	-1.85	3.90	1.11	0.92	-1.98	3.62	0.72	1.69	
NCU05120	DNA-dependent RNA polymerase II RPB140	18.41	17.81	17.20	17.97	-0.60	0.77	-1.21	0.16	-0.62	0.64	-1.49	-0.23	1.69	
NCU01429	serine-7	22.85	23.02	23.07	23.04	0.17	-0.03	0.22	0.02	0.15	-0.16	-0.06	-0.37	1.69	
NCU09707	hypothetical protein	18.54	17.73	17.33	17.82	-0.81	0.49	-1.21	0.09	-0.83	0.36	-1.49	-0.30	1.68	
NCU02746	vacuolar membrane ATPase-13	16.99	15.45	18.97	18.26	-1.54	-0.71	1.98	2.81	-1.56	-0.84	1.70	2.42	1.68	
NCU06780	tRNA	14.75	15.28	16.12	17.31	0.53	1.19	1.37	2.03	0.51	1.06	1.09	1.64	1.67	
NCU07887	ribonucleoside-diphosphate reductase small subunit	13.60	13.93	14.65	14.43	0.33	-0.22	1.05	0.50	0.31	-0.35	0.77	0.11	1.67	
NCU08287	pyrimidine-3	22.49	22.68	22.70	22.60	0.19	-0.10	0.21	-0.08	0.17	-0.23	-0.07	-0.47	1.65	
NCU01816	allantoicase-l	19.87	19.79	19.40	18.31	-0.08	-1.09	-0.47	-1.48	-0.10	-1.22	-0.75	-1.87	1.65	
NCU03797	ubiquitin C-terminal hydrolase	16.14	16.52	17.23	18.23	0.38	1.00	1.09	1.71	0.36	0.87	0.81	1.32	1.64	
NCU04315	dre-2	18.91	18.22	18.12	18.02	-0.69	-0.10	-0.79	-0.20	-0.71	-0.23	-1.07	-0.59	1.64	
NCU06342	phospholipase D	18.90	17.59	16.56	17.61	-1.31	1.05	-2.34	0.02	-1.33	0.92	-2.62	-0.37	1.64	
NCU07281	glucose-6-phosphate isomerase	23.56	23.84	23.64	23.83	0.28	0.19	0.08	-0.01	0.26	0.06	-0.20	-0.40	1.63	
NCU05387	hydrolase	18.39	18.81	19.38	19.32	0.42	-0.06	0.99	0.51	0.40	-0.19	0.71	0.12	1.63	
NCU03006	ergosterol-4	14.34	14.80	16.34	15.48	0.46	-0.86	2.00	0.68	0.44	-0.99	1.72	0.29	1.62	
NCU04907	hypothetical protein	18.12	17.27	18.73	19.05	-0.85	0.32	0.61	1.78	-0.87	0.19	0.33	1.39	1.61	
NCU02263	Sec14 cytosolic factor	19.61	20.24	20.83	20.93	0.63	0.10	1.22	0.69	0.61	-0.03	0.94	0.30	1.61	
NCU00134	exportin-T	15.11	14.48	15.58	16.49	-0.63	0.91	0.47	2.01	-0.65	0.78	0.19	1.62	1.61	
NCU09646	3-ketoacyl-CoA thiolase	14.34	16.36	17.92	17.42	2.02	-0.50	3.58	1.06	2.00	-0.63	3.30	0.67	1.61	
NCU07287	hypothetical protein	19.94	20.26	19.51	17.89	0.32	-1.62	-0.43	-2.37	0.30	-1.75	-0.71	-2.76	1.60	
NCU01955	autophagocytosis protein Aut1	15.86	14.94	14.09	15.10	-0.92	1.01	-1.77	0.16	-0.94	0.88	-2.05	-0.23	1.60	
NCU02082	cft-1	13.95	13.61	14.64	15.99	-0.34	1.35	0.69	2.38	-0.36	1.22	0.41	1.99	1.60	
NCU17177	glycylpeptide N-tetradecanoyltransferase	17.92	17.06	18.65	18.77	-0.86	0.12	0.73	1.71	-0.88	-0.01	0.45	1.32	1.60	
NCU04173	actin	23.07	22.27	24.15	23.87	-0.80	-0.28	1.08	1.60	-0.82	-0.41	0.80	1.21	1.60	
NCU09995	hypothetical protein	19.62	19.04	17.99	19.17	-0.58	1.18	-1.63	0.13	-0.60	1.05	-1.91	-0.26	1.59	
NCU02580	fumarate reductase Osm1	16.88	17.49	19.06	18.13	0.61	-0.93	2.18	0.64	0.59	-1.06	1.90	0.25	1.58	
NCU09264	APAF1-interacting protein	18.22	18.49	18.40	18.11	0.27	-0.29	0.18	-0.38	0.25	-0.42	-0.10	-0.77	1.58	
NCU03853	peptidyl-prolyl cis-trans isomerase	17.68	18.91	19.77	19.93	1.23	0.16	2.09	1.02	1.21	0.03	1.81	0.63	1.58	
NCU07439	hypothetical protein	17.27	16.70	16.69	16.63	-0.57	-0.06	-0.58	-0.07	-0.59	-0.19	-0.86	-0.46	1.58	
NCU07562	60S ribosomal protein L43	21.61	20.02	19.40	19.78	-1.59	0.38	-2.21	-0.24	-1.61	0.25	-2.49	-0.63	1.58	
NCU08352	cysteine-9	19.49	19.71	19.67	19.79	0.22	0.12	0.18	0.08	0.20	-0.01	-0.10	-0.31	1.58	
NCU03779	dihydroxyacetone kinase-1	18.26	18.78	19.54	19.27	0.52	-0.27	1.28	0.49	0.50	-0.40	1.00	0.10	1.57	
NCU02781	hypothetical protein	18.24	18.54	19.08	19.81	0.30	0.73	0.84	1.27	0.28	0.60	0.56	0.88	1.57	
NCU09182	stress responsive A/B barrel domain-containing protein	14.96	15.93	17.11	16.51	0.97	-0.60	2.15	0.58	0.95	-0.73	1.87	0.19	1.57	
NCU00793	trehalose phosphate synthase	19.02	18.64	20.01	19.67	-0.38	-0.34	0.99	1.03	-0.40	-0.47	0.71	0.64	1.57	
NCU04120	calmodulin A	18.75	17.05	15.72	17.09	-1.70	1.37	-3.03	0.04	-1.72	1.24	-3.31	-0.35	1.57	
NCU05288	rab GDP-dissociation inhibitor	22.73	22.44	22.56	22.55	-0.29	-0.01	-0.17	0.11	-0.31	-0.14	-0.45	-0.28	1.57	
NCU08471	tricarboxylic acid-9	17.18	15.14	19.87	18.59	-2.04	-1.28	2.69	3.45	-2.06	-1.41	2.41	3.06	1.57	
NCU04213	progesterone binding protein	16.57	17.04	16.70	16.71	0.47	0.01	0.13	-0.33	0.45	-0.12	-0.15	-0.72	1.56	
NCU04238	histone H1-binding protein	18.62	18.65	18.45	18.88	0.03	0.43	-0.17	0.23	0.01	0.30	-0.45	-0.16	1.56	
NCU07422	hypothetical protein	16.49	16.10	16.87	17.55	-0.39	0.68	0.38	1.45	-0.41	0.55	0.10	1.06	1.56	
NCU03704	ubiquitin carboxyl-terminal hydrolase 2	14.74	16.59	17.87	17.60	1.85	-0.27	3.13	1.01	1.83	-0.40	2.85	0.62	1.56	
NCU02701	dipeptidyl peptidase	19.65	20.26	19.61	19.78	0.61	0.17	-0.04	-0.48	0.59	0.04	-0.32	-0.87	1.55	
NCU06471	proline-1	14.36	16.33	17.45	18.07	1.97	0.62	3.09	1.74	1.95	0.49	2.81	1.35	1.55	
NCU06630	protein phosphatase-1	18.99	18.22	19.77	19.74	-0.77	-0.03	0.78	1.52	-0.79	-0.16	0.50	1.13	1.55	
NCU02181	40S ribosomal protein S4	23.07	23.14	22.44	23.19	0.07	0.75	-0.63	0.05	0.05	0.62	-0.91	-0.34	1.55	
NCU06228	hypothetical protein	15.39	16.71	17.60	17.66	1.32	0.06	2.21	0.95	1.30	-0.07	1.93	0.56	1.54	
NCU01786	ribose-phosphate pyrophosphokinase II	17.97	17.65	17.60	17.98	-0.32	0.38	-0.37	0.33	-0.34	0.25	-0.65	-0.06	1.54	
NCU01918	ARP2/3 complex 20 kDa subunit	17.91	17.77	18.23	18.60	-0.14	0.37	0.32	0.83	-0.16	0.24	0.04	0.44	1.54	
NCU03731	HAD superfamily hydrolase	18.23	18.14	18.84	19.84	-0.09	1.00	0.61	1.70	-0.11	0.87	0.33	1.31	1.54	
NCU02888	hypothetical protein	19.89	20.54	19.05	19.92	0.65	0.87	-0.84	-0.62	0.63	0.74	-1.12	-1.01	1.54	
NCU04856	glutamine-2	21.35	22.25	21.35	19.93	0.90	-1.42	0.00	-2.32	0.88	-1.55	-0.28	-2.71	1.53	
NCU03396	nucleolar protein nop-58	17.83	17.33	18.25	19.25	-0.50	1.00	0.42	1.92	-0.52	0.87	0.14	1.53	1.52	
NCU04140	FK506 resistant-2	21.91	21.81	20.79	21.86	-0.10	1.07	-1.12	0.05	-0.12	0.94	-1.40	-0.34	1.51	
NCU08568	ADP-ribose pyrophosphatase	17.63	17.53	18.30	19.51	-0.10	1.21	0.67	1.98	-0.12	1.08	0.39	1.59	1.51	
NCU03755	flavin dependent monooxygenase	18.88	19.21	18.76	19.14	0.33	0.38	-0.12	-0.07	0.31	0.25	-0.40	-0.46	1.50	
NCU06279	eukaryotic translation initiation factor 3	18.34	17.91	18.68	19.06	-0.43	0.38	0.34	1.15	-0.45	0.25	0.06	0.76	1.49	
NCU04185	protein kinase-1	16.15	13.62	17.50	17.65	-2.53	0.15	1.35	4.03	-2.55	0.02	1.07	3.64	1.49	
NCU01233	aldose reductase	19.24	18.61	20.13	19.90	-0.63	-0.23	0.89	1.29	-0.65	-0.36	0.61	0.90	1.48	
NCU09450	regulatory particle, non-ATPase-like-2	19.08	18.42	20.27	19.79	-0.66	-0.48	1.19	1.37	-0.68	-0.61	0.91	0.98	1.48	
NCU03076	delta-1-pyrroline-5-carboxylate dehydrogenase	16.54	16.64	17.28	17.10	0.10	-0.18	0.74	0.46	0.08	-0.31	0.46	0.07	1.48	
NCU07153	glutamate carboxypeptidase	21.63	21.45	21.58	21.38	-0.18	-0.20	-0.05	-0.07	-0.20	-0.33	-0.33	-0.46	1.47	
NCU08389	60S ribosomal protein L20	22.12	21.97	21.00	22.08	-0.15	1.08	-1.12	0.11	-0.17	0.95	-1.40	-0.28	1.47	
NCU06561	RHO protein GDP dissociation inhibitor-1	20.69	20.53	20.66	20.45	-0.16	-0.21	-0.03	-0.08	-0.18	-0.34	-0.31	-0.47	1.47	
NCU02322	mannose-6-phosphate isomerase	15.95	13.55	17.08	17.38	-2.40	0.30	1.13	3.83	-2.42	0.17	0.85	3.44	1.46	
NCU07829	60S ribosomal protein L7	22.34	22.23	22.27	21.81	-0.11	-0.46	-0.07	-0.42	-0.13	-0.59	-0.35	-0.81	1.46	
NCU09174	hypothetical protein	20.26	19.78	21.26	20.89	-0.48	-0.37	1.00	1.11	-0.50	-0.50	0.72	0.72	1.46	
NCU03905	hypothetical protein	14.77	15.04	13.66	14.80	0.27	1.14	-1.11	-0.24	0.25	1.01	-1.39	-0.63	1.46	
NCU07165	mannose-6-phosphate isomerase	19.36	19.64	20.27	20.05	0.28	-0.22	0.91	0.41	0.26	-0.35	0.63	0.02	1.46	
NCU02090	adenine phosphoribosyltransferase	18.12	17.36	19.17	18.82	-0.76	-0.35	1.05	1.46	-0.78	-0.48	0.77	1.07	1.45	
NCU05650	karyopherin Kap123	17.57	16.64	16.46	15.58	-0.93	-0.88	-1.11	-1.06	-0.95	-1.01	-1.39	-1.45	1.45	
NCU06035	elongation factor 1-beta	20.60	20.76	20.72	20.20	0.16	-0.52	0.12	-0.56	0.14	-0.65	-0.16	-0.95	1.45	
NCU05200	Pro-apoptotic serine protease nma-111	17.69	17.70	17.90	17.87	0.01	-0.03	0.21	0.17	-0.01	-0.16	-0.07	-0.22	1.44	
NCU07471	F-actin-capping protein subunit beta	17.63	18.15	17.87	17.31	0.52	-0.56	0.24	-0.84	0.50	-0.69	-0.04	-1.23	1.44	
NCU06726	nitrilase	15.70	16.99	15.87	15.26	1.29	-0.61	0.17	-1.73	1.27	-0.74	-0.11	-2.12	1.44	
NCU05418	deoxyhypusine synthase-1	17.15	17.34	16.53	17.32	0.19	0.79	-0.62	-0.02	0.17	0.66	-0.90	-0.41	1.44	
NCU03300	Neurospora fourteen-three-three homolog-1	22.43	21.87	22.89	23.06	-0.56	0.17	0.46	1.19	-0.58	0.04	0.18	0.80	1.43	
NCU01489	histidine phosphotransferase-1	18.10	18.73	17.55	18.25	0.63	0.70	-0.55	-0.48	0.61	0.57	-0.83	-0.87	1.43	
NCU07319	coatomer beta' subunit	19.06	17.98	19.53	19.98	-1.08	0.45	0.47	2.00	-1.10	0.32	0.19	1.61	1.42	
NCU02018	short chain dehydrogenase	16.55	18.53	19.50	19.71	1.98	0.21	2.95	1.18	1.96	0.08	2.67	0.79	1.42	
NCU02333	arginase-1	16.87	16.88	15.06	16.71	0.01	1.65	-1.81	-0.17	-0.01	1.52	-2.09	-0.56	1.42	
NCU07839	ATP-dependent RNA helicase dbp-2	17.73	18.16	17.63	17.99	0.43	0.36	-0.10	-0.17	0.41	0.23	-0.38	-0.56	1.41	
NCU02680	nuclear protein localization protein 4	16.47	14.79	17.74	17.44	-1.68	-0.30	1.27	2.65	-1.70	-0.43	0.99	2.26	1.41	
NCU06355	karyopherin	17.78	18.21	18.01	17.34	0.43	-0.67	0.23	-0.87	0.41	-0.80	-0.05	-1.26	1.40	
NCU05164	short chain dehydrogenase/reductase SDR	17.12	14.82	17.56	18.78	-2.30	1.22	0.44	3.96	-2.32	1.09	0.16	3.57	1.40	
NCU01838	nitrilase	18.80	18.46	18.59	18.42	-0.34	-0.17	-0.21	-0.04	-0.36	-0.30	-0.49	-0.43	1.40	
NCU06187	adenine-4	22.14	21.77	21.74	22.16	-0.37	0.42	-0.40	0.39	-0.39	0.29	-0.68	0.00	1.39	
NCU02650	regulatory particle, non-ATPase-like-5	18.96	18.85	19.39	20.22	-0.11	0.83	0.43	1.37	-0.13	0.70	0.15	0.98	1.39	
NCU08029	thiamine pyrophosphokinase	15.95	16.18	16.19	15.84	0.23	-0.35	0.24	-0.34	0.21	-0.48	-0.04	-0.73	1.39	
NCU03690	importin beta-2 subunit	17.79	17.93	17.47	18.05	0.14	0.58	-0.32	0.12	0.12	0.45	-0.60	-0.27	1.39	
NCU01318	protein transporter sec-23	15.98	15.70	16.29	16.52	-0.28	0.23	0.31	0.82	-0.30	0.10	0.03	0.43	1.39	
NCU01204	tropomyosin	20.17	19.68	19.77	19.89	-0.49	0.12	-0.40	0.21	-0.51	-0.01	-0.68	-0.18	1.38	
NCU09111	glucose-6-phosphate 1-dehydrogenase	22.91	22.69	22.79	23.08	-0.22	0.29	-0.12	0.39	-0.24	0.16	-0.40	0.00	1.38	
NCU06549	pyridoxine-2	20.23	20.53	20.52	20.33	0.30	-0.19	0.29	-0.20	0.28	-0.32	0.01	-0.59	1.38	
NCU00260	oxidoreductase	19.37	19.95	20.44	20.42	0.58	-0.02	1.07	0.47	0.56	-0.15	0.79	0.08	1.37	
NCU02399	hypothetical protein	16.35	17.75	18.51	18.45	1.40	-0.06	2.16	0.70	1.38	-0.19	1.88	0.31	1.37	
NCU09228	aminopeptidase 2	22.51	22.40	23.02	22.97	-0.11	-0.05	0.51	0.57	-0.13	-0.18	0.23	0.18	1.37	
NCU03108	glutamate carboxypeptidase	15.65	16.19	16.97	16.54	0.54	-0.43	1.32	0.35	0.52	-0.56	1.04	-0.04	1.37	
NCU05889	eukaryotic translation initiation factor 3 subunit 6	19.43	19.05	19.15	19.38	-0.38	0.23	-0.28	0.33	-0.40	0.10	-0.56	-0.06	1.37	

NCU01776	60S ribosomal protein L15	21.94	22.35	22.24	21.85		0.41	-0.39	0.30	-0.50		0.39	-0.52	0.02	-0.89	1.37	
NCU03004	acetate-3	18.46	19.01	19.43	19.66		0.55	0.23	0.97	0.65		0.53	0.10	0.69	0.26	1.36	
NCU01827	60S ribosomal protein L27	22.18	21.67	21.77	21.59		-0.51	-0.18	-0.41	-0.08		-0.53	-0.31	-0.69	-0.47	1.36	
NCU09770	acetate utilization-8	15.71	15.56	16.10	16.95		-0.15	0.85	0.39	1.39		-0.17	0.72	0.11	1.00	1.36	
NCU01587	cofilin	21.05	20.47	20.01	20.90		-0.58	0.89	-1.04	0.43		-0.60	0.76	-1.32	0.04	1.36	
NCU03702	rRNA 2'-O-methyltransferase fibrillarin	18.72	18.20	19.00	20.16		-0.52	1.16	0.28	1.96		-0.54	1.03	0.00	1.57	1.35	
NCU01992	coatomer subunit gamma	18.67	18.50	19.00	19.72		-0.17	0.72	0.33	1.22		-0.19	0.59	0.05	0.83	1.34	
NCU02289	ubiquitin-conjugating enzyme E2	16.17	14.82	17.56	16.96		-1.35	-0.60	1.39	2.14		-1.37	-0.73	1.11	1.75	1.34	
NCU07320	phosphatidylinositol transporter	16.50	15.56	17.00	17.21		-0.94	0.21	0.50	1.65		-0.96	0.08	0.22	1.26	1.34	
NCU01768	hypothetical protein	19.97	20.28	20.80	20.63		0.31	-0.17	0.83	0.35		0.29	-0.30	0.55	-0.04	1.33	
NCU00824	histone deacetylase-3	14.28	14.69	16.05	15.12		0.41	-0.93	1.77	0.43		0.39	-1.06	1.49	0.04	1.32	
NCU05810	cross pathway control-2	22.26	22.20	22.30	21.84		-0.06	-0.46	0.04	-0.36		-0.08	-0.59	-0.24	-0.75	1.32	
NCU08605	proteosome catalytic beta-2	17.69	17.44	16.61	17.73		-0.25	1.12	-1.08	0.29		-0.27	0.99	-1.36	-0.10	1.32	
NCU02075	heat shock protein 70-2	23.08	22.91	23.33	23.67		-0.17	0.34	0.25	0.76		-0.19	0.21	-0.03	0.37	1.32	
NCU09320	histidine-2	21.15	20.45	20.51	20.60		-0.70	0.09	-0.64	0.15		-0.72	-0.04	-0.92	-0.24	1.32	
NCU04192	vacuolar aspartyl aminopeptidase Lap4	18.27	19.16	18.65	18.06		0.89	-0.59	0.38	-1.10		0.87	-0.72	0.10	-1.49	1.32	
NCU00964	hypothetical protein	16.59	15.41	15.14	15.80		-1.18	0.66	-1.45	0.39		-1.20	0.53	-1.73	0.00	1.32	
NCU07725	chorismate mutase	17.15	16.67	17.47	17.73		-0.48	0.26	0.32	1.06		-0.50	0.13	0.04	0.67	1.31	
NCU03347	nicotinic acid-4	19.31	19.59	18.68	19.53		0.28	0.85	-0.63	-0.06		0.26	0.72	-0.91	-0.45	1.31	
NCU03127	urease Ure	15.82	16.43	15.91	16.06		0.61	0.15	0.09	-0.37		0.59	0.02	-0.19	-0.76	1.31	
NCU01422	multiprotein-bridging factor 1	14.21	13.44	14.37	15.18		-0.77	0.81	0.16	1.74		-0.79	0.68	-0.12	1.35	1.30	
NCU03150	60S ribosomal protein L24	21.09	19.81	19.67	20.01		-1.28	0.34	-1.42	0.20		-1.30	0.21	-1.70	-0.19	1.30	
NCU00261	CTP synthase	17.86	17.48	16.64	17.85		-0.38	1.21	-1.22	0.37		-0.40	1.08	-1.50	-0.02	1.30	
NCU06764	proteasome catalytic alpha-2	19.21	19.20	18.34	19.37		-0.01	1.03	-0.87	0.17		-0.03	0.90	-1.15	-0.22	1.29	
NCU02111	myosin-5	16.38	17.04	18.14	17.31		0.66	-0.83	1.76	0.27		0.64	-0.96	1.48	-0.12	1.29	
NCU06261	uracil phosphoribosyltransferase	16.66	16.74	16.36	16.95		0.08	0.59	-0.30	0.21		0.06	0.46	-0.58	-0.18	1.29	
NCU03350	xanthine dehydrogenase-1	14.85	16.23	15.39	14.13		1.38	-1.26	0.54	-2.10		1.36	-1.39	0.26	-2.49	1.29	
NCU03124	casein kinase II subunit alpha	18.89	18.81	18.06	19.05		-0.08	0.99	-0.83	0.24		-0.10	0.86	-1.11	-0.15	1.28	
NCU05420	aromatic-3	20.17	19.85	20.02	20.17		-0.32	0.15	-0.15	0.32		-0.34	0.02	-0.43	-0.07	1.28	
NCU03717	translation initiation factor 4E	14.37	14.65	15.32	17.22		0.28	1.90	0.95	2.57		0.26	1.77	0.67	2.18	1.27	
NCU07027	glycogen phosphorylase	23.29	22.89	23.03	22.70		-0.40	-0.33	-0.26	-0.19		-0.42	-0.46	-0.54	-0.58	1.27	
NCU05425	tricarboxylic acid-7	17.73	17.97	18.53	18.31		0.24	-0.22	0.80	0.34		0.22	-0.35	0.52	-0.05	1.27	
NCU03393	ribosome-associated protein-1	22.61	22.75	22.91	22.82		0.14	-0.09	0.30	0.07		0.12	-0.22	0.02	-0.32	1.27	
NCU08485	WD repeat protein	15.90	17.25	16.48	15.04		1.35	-1.44	0.58	-2.21		1.33	-1.57	0.30	-2.60	1.27	
NCU00304	hypothetical protein	17.53	17.66	17.55	16.24		0.13	-1.31	0.02	-1.42		0.11	-1.44	-0.26	-1.81	1.27	
NCU09014	cleavage and polyadenylation specificity factor subunit 5	17.84	17.07	16.87	17.60		-0.77	0.73	-0.97	0.53		-0.79	0.60	-1.25	0.14	1.27	
NCU01219	glutaredoxin	20.08	19.65	19.56	18.02		-0.43	-1.54	-0.52	-1.63		-0.45	-1.67	-0.80	-2.02	1.26	
NCU04306	methionine aminopeptidase 2B	17.59	14.80	17.49	19.19		-2.79	1.70	-0.10	4.39		-2.81	1.57	-0.38	4.00	1.25	
NCU05642	cysteinyl-tRNA synthetase	19.01	18.49	19.15	19.82		-0.52	0.67	0.14	1.33		-0.54	0.54	-0.14	0.94	1.25	
NCU07542	rad23-like	20.13	18.93	17.52	19.65		-1.20	2.13	-2.61	0.72		-1.22	2.00	-2.89	0.33	1.24	
NCU06617	myosin regulatory light chain cdc4	17.71	18.28	17.87	17.99		0.57	0.12	0.16	-0.29		0.55	-0.01	-0.12	-0.68	1.24	
NCU03125	NIMA-interacting protein TinC	20.17	19.51	19.44	20.03		-0.66	0.59	-0.73	0.52		-0.68	0.46	-1.01	0.13	1.24	
NCU00936	succinate semialdehyde dehydrogenase	17.14	15.94	17.74	17.80		-1.20	0.06	0.60	1.86		-1.22	-0.07	0.32	1.47	1.24	
NCU04115	clathrin light chain	13.51	16.02	17.10	16.23		2.51	-0.87	3.59	0.21		2.49	-1.00	3.31	-0.18	1.24	
NCU02273	PEP4 homolog	19.16	18.66	19.28	20.06		-0.50	0.78	0.12	1.40		-0.52	0.65	-0.16	1.01	1.23	
NCU08501	E3 ubiquitin-protein ligase TOM1-like protein	17.21	16.21	17.52	17.88		-1.00	0.36	0.31	1.67		-1.02	0.23	0.03	1.28	1.23	
NCU04337	survival factor 1	18.80	18.60	19.01	19.43		-0.20	0.42	0.21	0.83		-0.22	0.29	-0.07	0.44	1.23	
NCU06722	tryptophan-5	19.45	19.40	18.44	19.62		-0.05	1.18	-1.01	0.22		-0.07	1.05	-1.29	-0.17	1.23	
NCU07550	triosephosphate isomerase	23.58	23.53	23.90	24.49		-0.05	0.59	0.32	0.96		-0.07	0.46	0.04	0.57	1.23	
NCU09175	GPI-anchored cell wall beta-1,3-endoglucanase EglC	18.27	17.73	17.77	16.72		-0.54	-1.05	-0.50	-1.01		-0.56	-1.18	-0.78	-1.40	1.23	
NCU00414	adenosine kinase	22.39	21.93	22.10	21.93		-0.46	-0.17	-0.29	0.00		-0.48	-0.30	-0.57	-0.39	1.22	
NCU03605	amidohydrolase	14.98	15.33	16.40	15.65		0.35	-0.75	1.42	0.32		0.33	-0.88	1.14	-0.07	1.22	
NCU02407	dihydrolipoyl dehydrogenase	17.77	17.33	19.73	18.43		-0.44	-1.30	1.96	1.10		-0.46	-1.43	1.68	0.71	1.22	
NCU09123	calcium/calmodulin-dependent kinase-1	18.61	17.80	18.70	19.38		-0.81	0.68	0.09	1.58		-0.83	0.55	-0.19	1.19	1.21	
NCU07874	nuclear and cytoplasmic polyadenylated RNA-binding protein pub1	17.74	17.44	16.81	17.88		-0.30	1.07	-0.93	0.44		-0.32	0.94	-1.21	0.05	1.21	
NCU06290	hypothetical protein	15.75	15.85	16.53	16.25		0.10	-0.28	0.78	0.40		0.08	-0.41	0.50	0.01	1.21	
NCU00579	oxysterol binding protein 1	13.64	14.50	14.96	14.90		0.86	-0.06	1.32	0.40		0.84	-0.19	1.04	0.01	1.21	
NCU06091	vacuolar protein sorting-associated protein 74	14.09	14.86	16.00	14.98		0.77	-1.02	1.91	0.12		0.75	-1.15	1.63	-0.27	1.21	
NCU02260	regulatory particle, ATPase-like-3	18.60	18.84	19.63	19.17		0.24	-0.46	1.03	0.33		0.22	-0.59	0.75	-0.06	1.20	
NCU07962	hypothetical protein	16.05	16.72	17.60	16.87		0.67	-0.73	1.55	0.15		0.65	-0.86	1.27	-0.24	1.20	
NCU00518	MGS207 protein	15.85	16.09	16.49	17.46		0.24	0.97	0.64	1.37		0.22	0.84	0.36	0.98	1.20	
NCU03632	farnesyltransferase/geranylgeranyltransferase type I alpha subunit	16.06	15.14	16.10	16.87		-0.92	0.77	0.04	1.73		-0.94	0.64	-0.24	1.34	1.20	
NCU07688	rho GTPase activator Rga	14.45	14.57	14.28	14.79		0.12	0.51	-0.17	0.22		0.10	0.38	-0.45	-0.17	1.20	
NCU09699	histone acetyltransferase Spt10	17.29	18.03	17.59	17.52		0.74	-0.07	0.30	-0.51		0.72	-0.20	0.02	-0.90	1.20	
NCU04780	VEG136 protein	13.98	14.34	14.73	15.69		0.36	0.96	0.75	1.35		0.34	0.83	0.47	0.96	1.19	
NCU02395	uroporphyrinogen-III synthase	15.01	15.95	15.19	15.24		0.94	0.05	0.18	-0.71		0.92	-0.08	-0.10	-1.10	1.19	
NCU05777	ubiquitin carboxyl-terminal hydrolase 14	18.16	18.27	18.92	18.65		0.11	-0.27	0.76	0.38		0.09	-0.40	0.48	-0.01	1.19	
NCU08823	band	13.61	14.25	14.06	13.76		0.64	-0.30	0.45	-0.49		0.62	-0.43	0.17	-0.88	1.18	
NCU01452	40S ribosomal protein S1	22.77	22.12	22.09	22.71		-0.65	0.62	-0.68	0.59		-0.67	0.49	-0.96	0.20	1.18	
NCU04344	translation initiation factor eIF-2B alpha subunit	17.39	17.96	16.86	17.68		0.57	0.82	-0.53	-0.28		0.55	0.69	-0.81	-0.67	1.18	
NCU10852	exochitinase	14.35	13.30	14.37	15.12		-1.05	0.75	0.02	1.82		-1.07	0.62	-0.26	1.43	1.18	
NCU08894	glutamyl-tRNA synthetase	21.11	20.17	21.44	21.69		-0.94	0.25	0.33	1.52		-0.96	0.12	0.05	1.13	1.18	
NCU11371	COBW domain-containing protein 1	15.30	15.78	16.33	15.99		0.48	-0.34	1.03	0.21		0.46	-0.47	0.75	-0.18	1.18	
NCU06844	hypothetical protein	16.35	16.73	16.67	15.26		0.38	-1.41	0.32	-1.47		0.36	-1.54	0.04	-1.86	1.18	
NCU05252	deoxyhypusine hydroxylase	16.19	17.21	16.89	15.40		1.02	-1.49	0.70	-1.81		1.00	-1.62	0.42	-2.20	1.18	
NCU01368	proteosome catalytic beta-4	17.83	16.23	17.95	18.61		-1.60	0.66	0.12	2.38		-1.62	0.53	-0.16	1.99	1.17	
NCU03876	eukaryotic translation initiation factor 3 subunit 2	19.22	18.81	18.96	19.26		-0.41	0.30	-0.26	0.45		-0.43	0.17	-0.54	0.06	1.17	
NCU02806	Neurospora fourteen-three-three homolog-2	24.03	23.77	23.95	23.51		-0.26	-0.44	-0.08	-0.26		-0.28	-0.57	-0.36	-0.65	1.17	
NCU02109	UDP-N-acetylglucosamine pyrophosphorylase	20.70	21.12	21.60	21.35		0.42	-0.25	0.90	0.23		0.40	-0.38	0.62	-0.16	1.16	
NCU00202	coronin 1	20.01	20.50	20.43	20.24		0.49	-0.19	0.42	-0.26		0.47	-0.32	0.14	-0.65	1.16	
NCU02887	voltage-gated potassium channel beta-2 subunit	18.82	19.17	19.20	19.10		0.35	-0.10	0.38	-0.07		0.33	-0.23	0.10	-0.46	1.16	
NCU06472	histone acetyl transferase-1	15.55	16.48	16.25	14.93		0.93	-1.32	0.70	-1.55		0.91	-1.45	0.42	-1.94	1.16	
NCU02493	proteasome component C1	18.63	19.38	18.10	18.92		0.75	0.82	-0.53	-0.46		0.73	0.69	-0.81	-0.85	1.16	
NCU06711	hypothetical protein	17.99	17.95	18.29	18.91		-0.04	0.62	0.30	0.96		-0.06	0.49	0.02	0.57	1.16	
NCU02547	hypothetical protein	14.70	16.27	16.73	17.65		1.57	0.92	2.03	1.38		1.55	0.79	1.75	0.99	1.16	
NCU00028	S	19.90	20.41	19.96	20.24		0.51	0.28	0.06	-0.17		0.49	0.15	-0.22	-0.56	1.16	
NCU01945	IMP-specific 5'-nucleotidase 1	17.30	17.79	18.58	17.95		0.49	-0.63	1.28	0.16		0.47	-0.76	1.00	-0.23	1.15	
NCU07755	tyrosyl-tRNA synthetase	20.04	18.74	18.23	19.70		-1.30	1.47	-1.81	0.96		-1.32	1.34	-2.09	0.57	1.15	
NCU06661	60S ribosomal protein L22	21.27	20.86	20.95	19.69		-0.41	-1.26	-0.32	-1.17		-0.43	-1.39	-0.60	-1.56	1.15	
NCU04676	glutathione S-transferase	17.96	18.45	18.47	17.82		0.49	-0.65	0.51	-0.63		0.47	-0.78	0.23	-1.02	1.15	
NCU03606	replication protein A-1	16.69	16.42	15.73	16.90		-0.27	1.17	-0.96	0.48		-0.29	1.04	-1.24	0.09	1.15	
NCU09377	B-regulatory subunit-1	18.21	17.99	18.37	18.97		-0.22	0.60	0.16	0.98		-0.24	0.47	-0.12	0.59	1.14	
NCU03166	adenine-3A	22.06	21.92	22.13	21.82		-0.14	-0.31	0.07	-0.10		-0.16	-0.44	-0.21	-0.49	1.14	
NCU04579	dihydroxy-acid dehydratase	18.69	19.38	19.72	19.95		0.69	0.23	1.03	0.57		0.67	0.10	0.75	0.18	1.14	
NCU08390	hypothetical protein	19.14	20.71	18.90	19.37		1.57	0.47	-0.24	-1.34		1.55	0.34	-0.52	-1.73	1.13	
NCU09842	mitogen activated protein kinase-1	18.27	17.39	19.04	18.77		-0.88	-0.27	0.77	1.38		-0.90	-0.40	0.49	0.99	1.13	
NCU00775	tricarboxylic acid-6	19.26	19.83	20.29	20.00		0.57	-0.29	1.03	0.17		0.55	-0.42	0.75	-0.22	1.13	
NCU05269	heat shock protein 88	23.50	23.25	23.48	23.57		-0.25	0.09	-0.02	0.32		-0.27	-0.04	-0.30	-0.07	1.13	
NCU00150	histidine-7	18.27	17.11	18.20	18.97		-1.16	0.77	-0.07	1.86		-1.18	0.64	-0.35	1.47	1.13	

NCU07012	hypothetical protein	15.43	14.50	14.14	15.37		-0.93	1.23	-1.29	0.87		-0.95	1.10	-1.57	0.48	1.13	
NCU01002	coatomer subunit epsilon	18.09	18.72	18.61	18.28		0.63	-0.33	0.52	-0.44		0.61	-0.46	0.24	-0.83	1.13	
NCU08411	aspartate aminotransferase	20.25	20.04	20.24	19.77		-0.21	-0.47	-0.01	-0.27		-0.23	-0.60	-0.29	-0.66	1.12	
NCU02224	regulatory particle, non-ATPase-like-3	18.86	17.65	19.10	19.43		-1.21	0.33	0.24	1.78		-1.23	0.20	-0.04	1.39	1.12	
NCU02566	alanyl-tRNA synthetase	21.64	21.55	21.88	22.50		-0.09	0.62	0.24	0.95		-0.11	0.49	-0.04	0.56	1.12	
NCU02475	glycine dehydrogenase	15.65	17.85	15.21	15.85		2.20	0.64	-0.44	-2.00		2.18	0.51	-0.72	-2.39	1.11	
NCU01902	glucosamine 6-phosphate N-acetyltransferase	18.24	18.29	18.64	19.65		0.05	1.01	0.40	1.36		0.03	0.88	0.12	0.97	1.11	
NCU05832	hypothetical protein	17.18	17.97	17.74	17.39		0.79	-0.35	0.56	-0.58		0.77	-0.48	0.28	-0.97	1.11	
NCU03306	hypothetical protein	18.04	17.79	18.19	18.54		-0.25	0.35	0.15	0.75		-0.27	0.22	-0.13	0.36	1.11	
NCU04754	isoleucine-valine-6	16.68	17.56	17.90	18.62		0.88	0.72	1.22	1.06		0.86	0.59	0.94	0.67	1.11	
NCU02784	hypothetical protein	15.94	15.21	15.29	15.87		-0.73	0.58	-0.65	0.66		-0.75	0.45	-0.93	0.27	1.11	
NCU00567	arginine-6	14.65	14.72	15.62	15.12		0.07	-0.50	0.97	0.40		0.05	-0.63	0.69	0.01	1.10	
NCU08507	zinc finger protein zpr1	18.06	17.91	19.06	18.52		-0.15	-0.54	1.00	0.61		-0.17	-0.67	0.72	0.22	1.10	
NCU04202	nucleoside diphosphate kinase-1	22.97	22.83	23.04	22.56		-0.14	-0.48	0.07	-0.27		-0.16	-0.61	-0.21	-0.66	1.10	
NCU02479	glutamine synthetase	18.78	19.36	19.36	18.94		0.58	-0.42	0.58	-0.42		0.56	-0.55	0.30	-0.81	1.10	
NCU04130	acylase ACY 1	16.37	17.13	17.45	17.76		0.76	0.31	1.08	0.63		0.74	0.18	0.80	0.24	1.10	
NCU01939	nonsense-mediated mRNA decay protein	18.96	18.77	19.74	19.40		-0.19	-0.34	0.78	0.63		-0.21	-0.47	0.50	0.24	1.10	
NCU00422	curved DNA-binding protein	21.40	21.62	21.90	22.27		0.22	0.37	0.50	0.65		0.20	0.24	0.22	0.26	1.09	
NCU07415	proline iminopeptidase	17.32	17.23	17.41	16.51		-0.09	-0.90	0.09	-0.72		-0.11	-1.03	-0.19	-1.11	1.09	
NCU01381	hypothetical protein	16.77	18.09	18.45	19.53		1.32	1.08	1.68	1.44		1.30	0.95	1.40	1.05	1.09	
NCU09746	gephyrin	14.42	15.89	15.44	14.39		1.47	-1.05	1.02	-1.50		1.45	-1.18	0.74	-1.89	1.08	
NCU08997	repressible alkaline phosphatase	16.25	15.28	15.45	14.43		-0.97	-1.02	-0.80	-0.85		-0.99	-1.15	-1.08	-1.24	1.08	
NCU04342	hypothetical protein	18.48	18.58	18.99	18.91		0.10	-0.08	0.51	0.33		0.08	-0.21	0.23	-0.06	1.08	
NCU02319	aminopeptidase	20.53	20.67	21.09	20.97		0.14	-0.12	0.56	0.30		0.12	-0.25	0.28	-0.09	1.07	
NCU03438	Arp2/3 complex subunit	16.90	16.67	17.01	17.87		-0.23	0.86	0.11	1.20		-0.25	0.73	-0.17	0.81	1.07	
NCU04569	5-oxoprolinase	22.98	22.82	23.14	23.87		-0.16	0.73	0.16	1.05		-0.18	0.60	-0.12	0.66	1.07	
NCU06698	glycogenin	15.98	16.66	16.39	16.32		0.68	-0.07	0.41	-0.34		0.66	-0.20	0.13	-0.73	1.07	
NCU07414	hypothetical protein similar to protein mitochondrial targeting protein M	14.40	17.12	17.53	18.77		2.72	1.24	3.13	1.65		2.70	1.11	2.85	1.26	1.07	
NCU08340	ADP-ribosylation factor 1	18.13	17.90	19.11	18.57		-0.23	-0.54	0.98	0.67		-0.25	-0.67	0.70	0.28	1.07	
NCU08578	oxysterol binding protein	15.85	17.14	18.27	16.63		1.29	-1.64	2.42	-0.51		1.27	-1.77	2.14	-0.90	1.07	
NCU03131	FAD dependent oxidoreductase superfamily	14.87	16.75	17.12	17.46		1.88	0.34	2.25	0.71		1.86	0.21	1.97	0.32	1.07	
NCU01163	GAF domain nucleotide-binding protein	16.83	17.28	17.97	17.35		0.45	-0.62	1.14	0.07		0.43	-0.75	0.86	-0.32	1.07	
NCU08008	adenylyl cyclase-associated protein	17.47	16.48	18.94	17.94		-0.99	-1.00	1.47	1.46		-1.01	-1.13	1.19	1.07	1.06	
NCU08882	NAD binding Rossmann fold oxidoreductase	20.66	20.86	21.21	21.12		0.20	-0.09	0.55	0.26		0.18	-0.22	0.27	-0.13	1.06	
NCU03500	aminotransferase	17.74	16.66	18.74	18.20		-1.08	-0.54	1.00	1.54		-1.10	-0.67	0.72	1.15	1.06	
NCU06336	N2,N2-dimethylguanosine tRNA methyltransferase	14.72	16.18	15.98	14.51		1.46	-1.47	1.26	-1.67		1.44	-1.60	0.98	-2.06	1.06	
NCU01107	short-chain dehydrogenase	16.90	17.40	18.31	17.42		0.50	-0.89	1.41	0.02		0.48	-1.02	1.13	-0.37	1.06	
NCU04910	hypothetical protein	18.37	17.99	16.70	18.64		-0.38	1.94	-1.67	0.65		-0.40	1.81	-1.95	0.26	1.06	
NCU10066	coatomer alpha subunit	19.35	19.30	19.58	19.78		-0.05	0.20	0.23	0.48		-0.07	0.07	-0.05	0.09	1.05	
NCU01177	NAD dependent epimerase/dehydratase	17.70	18.87	19.18	20.06		1.17	0.88	1.48	1.19		1.15	0.75	1.20	0.80	1.05	
NCU08791	catalase-1	17.89	18.67	18.60	18.16		0.78	-0.44	0.71	-0.51		0.76	-0.57	0.43	-0.90	1.05	
NCU09309	proteasome component PRE2	18.85	17.61	18.48	19.43		-1.24	0.95	-0.37	1.82		-1.26	0.82	-0.65	1.43	1.05	
NCU10058	phosphoglucomutase 2	23.62	23.90	24.11	23.70		0.28	-0.41	0.49	-0.20		0.26	-0.54	0.21	-0.59	1.04	
NCU06410	GTP-binding protein YPT52	14.96	15.55	15.97	15.54		0.59	-0.43	1.01	-0.01		0.57	-0.56	0.73	-0.40	1.04	
NCU10273	Hsc70 cochaperone	16.77	17.02	17.31	18.28		0.25	0.97	0.54	1.26		0.23	0.84	0.26	0.87	1.04	
NCU05342	hypothetical protein	18.80	18.50	18.82	19.47		-0.30	0.65	0.02	0.97		-0.32	0.52	-0.26	0.58	1.04	
NCU03813	formate dehydrogenase	20.79	21.08	21.50	21.25		0.29	-0.25	0.71	0.17		0.27	-0.38	0.43	-0.22	1.04	
NCU00633	hypothetical protein	18.25	18.40	18.71	20.18		0.15	1.47	0.46	1.78		0.13	1.34	0.18	1.39	1.04	
NCU09004	eukaryotic translation initiation factor 6	18.61	18.54	18.82	19.35		-0.07	0.53	0.21	0.81		-0.09	0.40	-0.07	0.42	1.04	
NCU09366	proteasome catalytic beta-6	19.09	19.75	19.71	19.42		0.66	-0.29	0.62	-0.33		0.64	-0.42	0.34	-0.72	1.04	
NCU03703	60S ribosomal protein L17	21.45	21.31	21.55	21.00		-0.14	-0.55	0.10	-0.31		-0.16	-0.68	-0.18	-0.70	1.03	
NCU07409	hypothetical protein	16.84	17.49	17.49	17.17		0.65	-0.32	0.65	-0.32		0.63	-0.45	0.37	-0.71	1.03	
NCU11395	S	13.20	16.34	16.80	15.26		3.14	-1.54	3.60	-1.08		3.12	-1.67	3.32	-1.47	1.03	
NCU04791	menadione-induced gene-10	17.11	17.61	16.81	17.50		0.50	0.69	-0.30	-0.11		0.48	0.56	-0.58	-0.50	1.02	
NCU05942	proteosome catalytic alpha-3	19.53	19.19	19.44	18.95		-0.34	-0.49	-0.09	-0.24		-0.36	-0.62	-0.37	-0.63	1.02	
NCU03061	translation initiation factor RLI1	19.38	18.98	18.73	19.75		-0.40	1.02	-0.65	0.77		-0.42	0.89	-0.93	0.38	1.02	
NCU04277	threonine-4	19.71	19.00	19.25	19.27		-0.71	0.02	-0.46	0.27		-0.73	-0.11	-0.74	-0.12	1.02	
NCU00768	mRNA binding post-transcriptional regulator	17.56	17.80	16.69	17.95		0.24	1.26	-0.87	0.15		0.22	1.13	-1.15	-0.24	1.01	
NCU01249	importin alpha subunit	19.26	18.52	19.70	19.67		-0.74	-0.03	0.44	1.15		-0.76	-0.16	0.16	0.76	1.01	
NCU04552	cytoplasmic ribosomal protein-5	19.85	20.39	20.71	20.34		0.54	-0.37	0.86	-0.05		0.52	-0.50	0.58	-0.44	1.01	
NCU00481	a-pheromone processing metallopeptidase Ste23	18.34	18.91	19.25	18.82		0.57	-0.43	0.91	-0.09		0.55	-0.56	0.63	-0.48	1.01	
NCU04470	HD domain-containing protein	15.92	15.44	15.61	16.28		-0.48	0.67	-0.31	0.84		-0.50	0.54	-0.59	0.45	1.01	
NCU02998	nicotinate-nucleotide pyrophosphorylase	18.80	18.29	18.59	19.29		-0.51	0.70	-0.21	1.00		-0.53	0.57	-0.49	0.61	1.01	
NCU10477	ubiquitin-conjugating enzyme E2	16.87	17.28	16.46	17.27		0.41	0.81	-0.41	-0.01		0.39	0.68	-0.69	-0.40	1.01	
NCU00649	nicotinic acid-6	17.99	18.91	19.18	18.86		0.92	-0.32	1.19	-0.05		0.90	-0.45	0.91	-0.44	1.00	
NCU00847	hypothetical protein	17.53	17.97	18.74	17.94		0.44	-0.80	1.21	-0.03		0.42	-0.93	0.93	-0.42	1.00	
NCU11427	CUE domain-containing protein	14.99	12.73	15.53	15.40		-2.26	-0.13	0.54	2.67		-2.28	-0.26	0.26	2.28	1.00	
NCU00665	Ser/Thr protein phosphatase	14.31	16.58	16.15	14.68		2.27	-1.47	1.84	-1.90		2.25	-1.60	1.56	-2.29	1.00	
NCU08991	sulfur control-3	17.34	17.59	17.85	18.42		0.25	0.57	0.51	0.83		0.23	0.44	0.23	0.44	1.00	
NCU06432	40S ribosomal protein S12	19.99	19.46	19.72	19.77		-0.53	0.05	-0.27	0.31		-0.55	-0.08	-0.55	-0.08	1.00	
NCU10008	tricarboxylic acid-14	19.87	20.68	20.93	20.31		0.81	-0.62	1.06	-0.37		0.79	-0.75	0.78	-0.76	1.00	
NCU02960	peroxin 5	16.50	15.43	15.85	16.86		-1.07	1.01	-0.65	1.43		-1.09	0.88	-0.93	1.04	1.00	
NCU04448	T-complex protein 1 subunit alpha	17.86	17.96	18.31	18.26		0.10	-0.05	0.45	0.30		0.08	-0.18	0.17	-0.09	0.99	
NCU07574	hypothetical protein	17.01	16.15	16.10	17.47		-0.86	1.37	-0.91	1.32		-0.88	1.24	-1.19	0.93	0.99	
NCU08595	ribosome biogenesis protein	13.58	14.97	15.21	14.82		1.39	-0.39	1.63	-0.15		1.37	-0.52	1.35	-0.54	0.99	
NCU09709	T-complex protein 1 subunit zeta	17.60	17.97	18.28	17.93		0.37	-0.35	0.68	-0.04		0.35	-0.48	0.40	-0.43	0.99	
NCU01523	GTP-binding protein ypt3	16.27	15.13	17.71	16.66		-1.14	-1.05	1.44	1.53		-1.16	-1.18	1.16	1.14	0.99	
NCU06042	FAD dependent oxidoreductase	18.34	18.65	18.94	17.48		0.31	-1.46	0.60	-1.17		0.29	-1.59	0.32	-1.56	0.99	
NCU06047	40S ribosomal protein S2	22.30	22.32	22.14	22.71		0.02	0.57	-0.16	0.39		0.00	0.44	-0.44	0.00	0.98	
NCU11357	cell cycle control protein	16.86	16.43	16.22	17.31		-0.43	1.09	-0.64	0.88		-0.45	0.96	-0.92	0.49	0.98	
NCU07868	hypothetical protein	17.99	18.20	18.45	19.17		0.21	0.72	0.46	0.97		0.19	0.59	0.18	0.58	0.98	
NCU04164	hypothetical protein	18.55	17.68	17.96	17.31		-0.87	-0.65	-0.59	-0.37		-0.89	-0.78	-0.87	-0.76	0.98	
NCU09547	U4/U6.U5 tri-snRNP-associated protein 2	12.82	13.36	14.06	13.15		0.54	-0.91	1.24	-0.21		0.52	-1.04	0.96	-0.60	0.98	
NCU00590	hypothetical protein	15.81	14.75	15.71	16.16		-1.06	0.45	-0.10	1.41		-1.08	0.32	-0.38	1.02	0.97	
NCU00892	methionine-6	14.81	16.50	14.00	15.26		1.69	1.26	-0.81	-1.24		1.67	1.13	-1.09	-1.63	0.97	
NCU03204	iron-sulfur cluster assembly associated protein Nar1	12.97	13.30	13.81	13.31		0.33	-0.50	0.84	0.01		0.31	-0.63	0.56	-0.38	0.97	
NCU06892	40S ribosomal protein S20	21.22	20.59	20.87	20.94		-0.63	0.07	-0.35	0.35		-0.65	-0.06	-0.63	-0.04	0.97	
NCU06105	ADP-ribosylation factor-binding protein GGA1	15.34	16.02	17.29	15.62		0.68	-1.67	1.95	-0.40		0.66	-1.80	1.67	-0.79	0.97	
NCU03139	histidine-3	22.08	22.16	22.87	22.46		0.08	-0.41	0.79	0.30		0.06	-0.54	0.51	-0.09	0.96	
NCU00602	siroheme synthase	16.27	16.21	16.57	14.19		-0.06	-2.38	0.30	-2.02		-0.08	-2.51	0.02	-2.41	0.96	
NCU06732	leukotriene A-4 hydrolase	18.84	17.96	18.39	19.00		-0.88	0.61	-0.45	1.04		-0.90	0.48	-0.73	0.65	0.96	
NCU08909	beta-1,3-glucanosyltransferase	19.12	17.77	18.13	18.70		-1.35	0.57	-0.99	0.93		-1.37	0.44	-1.27	0.54	0.96	
NCU02428	nucleolar essential protein 1	16.40	15.20	15.89	16.62		-1.20	0.73	-0.51	1.42		-1.22	0.60	-0.79	1.03	0.96	
NCU09331	HMF1	20.78	21.17	21.27	21.23		0.39	-0.04	0.49	0.06		0.37	-0.17	0.21	-0.33	0.95	
NCU00493	coatomer subunit delta	19.20	17.55	18.58	19.41		-1.65	0.83	-0.62	1.86		-1.67	0.70	-0.90	1.47	0.95	
NCU03148	nascent polypeptide-associated complex beta subunit	21.41	21.67	21.02	21.84		0.26	0.82	-0.39	0.17		0.24	0.69	-0.67	-0.22	0.95	
NCU04020	lysyl-tRNA synthetase	21.43	21.46	19.96	21.92		0.03	1.96	-1.47	0.46		0.01	1.83	-1.75	0.07	0.95	
NCU03633	mevalonate kinase	14.35	14.93	15.69	14.56		0.58	-1.13	1.34	-0.37		0.56	-1.26	1.06	-0.76	0.95	
NCU09230	cysteine-16	17.22	17.63	18.03	17.28		0.41	-0.75	0.81	-0.35		0.39	-0.88	0.53	-0.74	0.95	

NCU01412	proline-3	19.74	20.55	20.21	20.20		0.81	-0.01	0.47	-0.35		0.79	-0.14	0.19	-0.74	0.95	
NCU05410	arginine-5	15.57	15.66	16.05	13.99		0.09	-2.06	0.48	-1.67		0.07	-2.19	0.20	-2.06	0.94	
NCU01793	RNA binding domain-containing protein	19.15	17.87	18.20	18.16		-1.28	-0.04	-0.95	0.29		-1.30	-0.17	-1.23	-0.10	0.94	
NCU00200	tryptophan-1	19.77	19.91	20.27	20.12		0.14	-0.15	0.50	0.21		0.12	-0.28	0.22	-0.18	0.94	
NCU10067	regulatory particle, non-ATPase-like-12	18.00	17.71	17.86	19.05		-0.29	1.19	-0.14	1.34		-0.31	1.06	-0.42	0.95	0.94	
NCU04904	hypothetical protein	15.79	15.80	16.10	15.58		0.01	-0.52	0.31	-0.22		-0.01	-0.65	0.03	-0.61	0.94	
NCU04153	pseudouridine synthase	15.99	15.73	15.89	16.79		-0.26	0.90	-0.10	1.06		-0.28	0.77	-0.38	0.67	0.94	
NCU02657	ethionine resistant-1	22.77	22.52	22.80	22.58		-0.25	-0.22	0.03	0.06		-0.27	-0.35	-0.25	-0.33	0.94	
NCU06875	hypothetical protein	17.02	18.44	17.54	17.53		1.42	-0.01	0.52	-0.91		1.40	-0.14	0.24	-1.30	0.94	
NCU09744	nuclear pore complex subunit	17.10	16.71	16.22	17.67		-0.39	1.45	-0.88	0.96		-0.41	1.32	-1.16	0.57	0.93	
NCU06836	acetate utilization-5	17.03	16.98	17.27	16.93		-0.05	-0.34	0.24	-0.05		-0.07	-0.47	-0.04	-0.44	0.93	
NCU02433	ATG4 protein	14.55	14.13	14.20	15.60		-0.42	1.40	-0.35	1.47		-0.44	1.27	-0.63	1.08	0.93	
NCU02765	RNA binding protein	16.25	15.75	15.29	16.90		-0.50	1.61	-0.96	1.15		-0.52	1.48	-1.24	0.76	0.93	
NCU02335	short chain dehydrogenase	12.80	14.63	14.29	13.68		1.83	-0.61	1.49	-0.95		1.81	-0.74	1.21	-1.34	0.92	
NCU01290	centromere/microtubule-binding protein CBF5	17.06	16.31	15.87	17.92		-0.75	2.05	-1.19	1.61		-0.77	1.92	-1.47	1.22	0.92	
NCU07365	proteasome catalytic beta-7	18.36	18.75	18.87	18.85		0.39	-0.02	0.51	0.10		0.37	-0.15	0.23	-0.29	0.92	
NCU08133	carbonic anhydrase 2	20.00	20.14	20.68	20.34		0.14	-0.34	0.68	0.20		0.12	-0.47	0.40	-0.19	0.92	
NCU00326	calcium homeostasis protein Regucalcin	18.50	17.72	18.54	18.81		-0.78	0.27	0.04	1.09		-0.80	0.14	-0.24	0.70	0.92	
NCU01855	hypothetical protein	13.90	14.51	15.17	13.74		0.61	-1.43	1.27	-0.77		0.59	-1.56	0.99	-1.16	0.92	
NCU06247	hypothetical protein	18.26	18.70	19.32	18.35		0.44	-0.97	1.06	-0.35		0.42	-1.10	0.78	-0.74	0.91	
NCU02509	60S ribosomal protein L11	19.84	19.67	19.82	20.52		-0.17	0.70	-0.02	0.85		-0.19	0.57	-0.30	0.46	0.91	
NCU00413	60S ribosomal protein L2	21.34	21.56	21.15	21.78		0.22	0.63	-0.19	0.22		0.20	0.50	-0.47	-0.17	0.91	
NCU07831	eukaryotic translation initiation factor 3	20.44	20.22	20.25	21.01		-0.22	0.76	-0.19	0.79		-0.24	0.63	-0.47	0.40	0.91	
NCU09700	T-complex protein 1 subunit beta	17.64	18.28	18.42	18.35		0.64	-0.07	0.78	0.07		0.62	-0.20	0.50	-0.32	0.91	
NCU03283	2-keto-4-pentenoate hydratase	18.45	18.81	19.30	18.45		0.36	-0.85	0.85	-0.36		0.34	-0.98	0.57	-0.75	0.91	
NCU08671	phosphomevalonate kinase	18.53	19.64	19.68	19.46		1.11	-0.22	1.15	-0.18		1.09	-0.35	0.87	-0.57	0.91	
NCU09347	fructose-2,6-bisphosphatase	15.59	13.63	18.03	15.79		-1.96	-2.24	2.44	2.16		-1.98	-2.37	2.16	1.77	0.91	
NCU07366	glucosamine-fructose-6-phosphate aminotransferase	19.90	19.74	20.05	20.24		-0.16	0.19	0.15	0.50		-0.18	0.06	-0.13	0.11	0.91	
NCU04237	phosphopantothenoylcysteine decarboxylase	15.90	15.40	15.80	16.11		-0.50	0.31	-0.10	0.71		-0.52	0.18	-0.38	0.32	0.91	
NCU08840	chromatin remodeling complex subunit	16.37	14.76	17.02	16.64		-1.61	-0.38	0.65	1.88		-1.63	-0.51	0.37	1.49	0.90	
NCU04909	hypothetical protein	17.20	16.40	17.00	17.40		-0.80	0.40	-0.20	1.00		-0.82	0.27	-0.48	0.61	0.90	
NCU08040	NEDD8-activating enzyme E1 regulatory subunit	17.18	16.02	16.84	17.33		-1.16	0.49	-0.34	1.31		-1.18	0.36	-0.62	0.92	0.90	
NCU02798	hypothetical protein	19.19	19.36	20.20	19.47		0.17	-0.73	1.01	0.11		0.15	-0.86	0.73	-0.28	0.90	
NCU09442	DUF757 domain-containing protein	16.05	15.40	16.86	16.39		-0.65	-0.47	0.81	0.99		-0.67	-0.60	0.53	0.60	0.90	
NCU02385	hypothetical protein	15.77	15.77	14.46	16.37		0.00	1.91	-1.31	0.60		-0.02	1.78	-1.59	0.21	0.90	
NCU07167	isoflavone reductase	19.62	20.06	20.28	20.47		0.44	0.19	0.66	0.41		0.42	0.06	0.38	0.02	0.90	
NCU02010	leucine-4	20.52	19.59	20.83	20.83		-0.93	0.00	0.31	1.24		-0.95	-0.13	0.03	0.85	0.89	
NCU01240	hypothetical protein	18.12	17.94	18.05	18.90		-0.18	0.85	-0.07	0.96		-0.20	0.72	-0.35	0.57	0.89	
NCU08627	cytoplasmic ribosomal protein-7	19.53	19.65	20.10	19.85		0.12	-0.25	0.57	0.20		0.10	-0.38	0.29	-0.19	0.89	
NCU02788	ric8-like	15.31	15.70	16.26	14.98		0.39	-1.28	0.95	-0.72		0.37	-1.41	0.67	-1.11	0.89	
NCU02605	DNA-directed RNA polymerase I/II/III subunit 10	14.53	17.40	14.89	15.24		2.87	0.35	0.36	-2.16		2.85	0.22	0.08	-2.55	0.89	
NCU06684	RNA binding protein	17.57	16.14	17.09	17.63		-1.43	0.54	-0.48	1.49		-1.45	0.41	-0.76	1.10	0.89	
NCU03804	serine/threonine-protein phosphatase 2B catalytic subunit	18.32	17.37	17.90	18.32		-0.95	0.42	-0.42	0.95		-0.97	0.29	-0.70	0.56	0.89	
NCU03836	O-sialoglycoprotein endopeptidase	17.05	16.09	16.67	17.10		-0.96	0.43	-0.38	1.01		-0.98	0.30	-0.66	0.62	0.89	
NCU02124	dienelactone hydrolase	20.82	21.25	21.30	21.32		0.43	0.02	0.48	0.07		0.41	-0.11	0.20	-0.32	0.88	
NCU02393	mitogen-activated protein kinase-2	19.14	19.39	20.10	19.32		0.25	-0.78	0.96	-0.07		0.23	-0.91	0.68	-0.46	0.88	
NCU02397	isoleucine-valine-5	19.82	19.80	20.28	20.20		-0.02	-0.08	0.46	0.40		-0.04	-0.21	0.18	0.01	0.88	
NCU02076	eukaryotic translation initiation factor 4E	14.03	15.50	15.60	16.11		1.47	0.51	1.57	0.61		1.45	0.38	1.29	0.22	0.87	
NCU07126	hypothetical protein	14.05	14.56	14.48	14.54		0.51	0.06	0.43	-0.02		0.49	-0.07	0.15	-0.41	0.87	
NCU06556	thioredoxin II	21.30	21.21	21.36	21.90		-0.09	0.54	0.06	0.69		-0.11	0.41	-0.22	0.30	0.87	
NCU02560	hypothetical protein	17.68	16.67	17.42	17.81		-1.01	0.39	-0.26	1.14		-1.03	0.26	-0.54	0.75	0.87	
NCU02634	hypothetical protein	18.67	17.96	18.36	18.61		-0.71	0.25	-0.31	0.65		-0.73	0.12	-0.59	0.26	0.87	
NCU07182	40S ribosomal protein S24	21.31	21.82	21.55	21.77		0.51	0.22	0.24	-0.05		0.49	0.09	-0.04	-0.44	0.87	
NCU00707	hypothetical protein	14.81	15.91	14.63	15.33		1.10	0.70	-0.18	-0.58		1.08	0.57	-0.46	-0.97	0.87	
NCU02505	succinate	23.20	22.95	23.24	23.18		-0.25	-0.06	0.04	0.23		-0.27	-0.19	-0.24	-0.16	0.87	
NCU09109	60S ribosomal protein L33	17.46	20.22	19.88	19.44		2.76	-0.44	2.42	-0.78		2.74	-0.57	2.14	-1.17	0.87	
NCU09803	thioredoxin	17.65	16.75	17.11	16.85		-0.90	-0.26	-0.54	0.10		-0.92	-0.39	-0.82	-0.29	0.87	
NCU02133	superoxide dismutase-1	22.33	22.25	23.00	22.68		-0.08	-0.32	0.67	0.43		-0.10	-0.45	0.39	0.04	0.87	
NCU08669	betaine aldehyde dehydrogenase 2	20.00	19.74	20.19	20.35		-0.26	0.16	0.19	0.61		-0.28	0.03	-0.09	0.22	0.86	
NCU06923	methionine aminopeptidase 1	17.96	18.58	18.57	18.55		0.62	-0.02	0.61	-0.03		0.60	-0.15	0.33	-0.42	0.86	
NCU00629	embden-meyerhof pathway-3	21.66	22.34	22.27	22.25		0.68	-0.02	0.61	-0.09		0.66	-0.15	0.33	-0.48	0.86	
NCU02720	hypothetical protein	17.50	16.67	17.56	17.75		-0.83	0.19	0.06	1.08		-0.85	0.06	-0.22	0.69	0.86	
NCU04510	glycerol dehydrogenase-3	20.90	20.82	21.76	21.21		-0.08	-0.55	0.86	0.39		-0.10	-0.68	0.58	0.00	0.85	
NCU04221	trehalase-2	14.89	15.33	13.54	15.54		0.44	2.00	-1.35	0.21		0.42	1.87	-1.63	-0.18	0.85	
NCU04280	aconitate hydratase	15.47	15.54	15.76	16.18		0.07	0.42	0.29	0.64		0.05	0.29	0.01	0.25	0.85	
NCU08955	UBX domain-containing protein	13.25	14.15	16.41	12.43		0.90	-3.98	3.16	-1.72		0.88	-4.11	2.88	-2.11	0.85	
NCU03050	ARP2/3 complex 34 kDa subunit	19.72	19.72	19.95	20.22		0.00	0.27	0.23	0.50		-0.02	0.14	-0.05	0.11	0.85	
NCU06702	yop-1	17.33	16.10	16.56	16.64		-1.23	0.08	-0.77	0.54		-1.25	-0.05	-1.05	0.15	0.85	
NCU03980	T-complex protein 1 subunit epsilon	17.78	17.96	18.14	18.87		0.18	0.73	0.36	0.91		0.16	0.60	0.08	0.52	0.85	
NCU05554	unknown-25	21.86	21.84	21.95	22.32		-0.02	0.37	0.09	0.48		-0.04	0.24	-0.19	0.09	0.85	
NCU02438	dihydrolipoamide succinyltransferase	17.00	16.75	18.23	17.25		-0.25	-0.98	1.23	0.50		-0.27	-1.11	0.95	0.11	0.84	
NCU06431	40S ribosomal protein S22	20.63	20.71	21.50	20.87		0.08	-0.63	0.87	0.16		0.06	-0.76	0.59	-0.23	0.84	
NCU03304	proteosome catalytic beta-3	18.32	19.51	19.30	19.19		1.19	-0.11	0.98	-0.32		1.17	-0.24	0.70	-0.71	0.83	
NCU06622	U2 small nuclear ribonucleoprotein A'	13.85	16.03	14.21	14.61		2.18	0.40	0.36	-1.42		2.16	0.27	0.08	-1.81	0.83	
NCU08935	peroxisomal-coenzyme A synthetase	17.47	16.43	17.15	17.48		-1.04	0.33	-0.32	1.05		-1.06	0.20	-0.60	0.66	0.83	
NCU08973	SNARE protein Ykt6	15.35	15.41	17.22	15.35		0.06	-1.87	1.87	-0.06		0.04	-2.00	1.59	-0.45	0.83	
NCU10061	proteasome catalytic alpha-1	19.42	19.85	19.76	19.90		0.43	0.14	0.34	0.05		0.41	0.01	0.06	-0.34	0.83	
NCU06464	hypothetical protein	21.34	21.80	20.81	21.88		0.46	1.07	-0.53	0.08		0.44	0.94	-0.81	-0.31	0.83	
NCU00477	carboxypeptidase Y	19.15	17.92	18.91	19.21		-1.23	0.30	-0.24	1.29		-1.25	0.17	-0.52	0.90	0.83	
NCU07929	eukaryotic translation initiation factor 3 subunit 3	18.06	18.30	17.01	18.73		0.24	1.72	-1.05	0.43		0.22	1.59	-1.33	0.04	0.82	
NCU00519	ribulose-phosphate 3-epimerase	18.51	18.48	17.81	19.19		-0.03	1.38	-0.70	0.71		-0.05	1.25	-0.98	0.32	0.82	
NCU06664	translin	15.44	15.42	15.49	16.44		-0.02	0.95	0.05	1.02		-0.04	0.82	-0.23	0.63	0.82	
NCU00726	cyclosporin-resistant-1	23.80	23.11	23.49	23.47		-0.69	-0.02	-0.31	0.36		-0.71	-0.15	-0.59	-0.03	0.81	
NCU06998	scavenger mRNA decapping enzyme	15.04	14.38	14.75	14.48		-0.66	-0.27	-0.29	0.10		-0.68	-0.40	-0.57	-0.29	0.81	
OLEGCP	custom peptide set20.67	21.66	21.39	20.92	23.40		-0.27	2.48	-0.74	2.01		-0.29	2.35	-1.02	1.62	0.81	
NCU08094	dihydrofolate reductase	16.84	17.79	16.00	17.47		0.95	1.47	-0.84	-0.32		0.93	1.34	-1.12	-0.71	0.81	
NCU03651	NADP-dependent malic enzyme	18.31	18.48	19.18	18.26		0.17	-0.92	0.87	-0.22		0.15	-1.05	0.59	-0.61	0.80	
NCU04815	lactoylglutathione lyase	17.88	17.97	18.46	17.85		0.09	-0.61	0.58	-0.12		0.07	-0.74	0.30	-0.51	0.80	
NCU04479	LAP2	19.39	18.71	19.12	19.20		-0.68	0.08	-0.27	0.49		-0.70	-0.05	-0.55	0.10	0.80	
NCU00951	inorganic pyrophosphatase	23.12	23.32	23.53	23.94		0.20	0.41	0.41	0.62		0.18	0.28	0.13	0.23	0.80	
NCU01369	ATP-dependent RNA helicase ded-1	19.49	19.58	19.70	20.51		0.09	0.81	0.21	0.93		0.07	0.68	-0.07	0.54	0.80	
NCU06554	phosphoserine phosphatase	19.15	17.49	18.91	19.10		-1.66	0.19	-0.24	1.61		-1.68	0.06	-0.52	1.22	0.79	
NCU04594	4-carboxymuconolactone decarboxylase	15.12	16.27	16.32	17.27		1.15	0.95	1.20	1.00		1.13	0.82	0.92	0.61	0.79	
NCU01157	glutamate-cysteine ligase catalytic subunit	15.64	17.07	17.08	17.82		1.43	0.74	1.44	0.75		1.41	0.61	1.16	0.36	0.79	
NCU08920	ATP-binding cassette sub-family F member 2	18.28	17.66	18.79	18.57		-0.62	-0.22	0.51	0.91		-0.64	-0.35	0.23	0.52	0.79	
NCU07567	T-complex protein 1 subunit theta	19.67	19.06	19.49	19.64		-0.61	0.15	-0.18	0.58		-0.63	0.02	-0.46	0.19	0.79	
NCU04097	ABC transporter	19.31	19.36	19.95	19.56		0.05	-0.39	0.64	0.20		0.03	-0.52	0.36	-0.19	0.79	
NCU01160	ATP-dependent RNA helicase dbp-5	16.87	16.01	16.89	17.04		-0.86	0.15	0.02	1.03		-0.88	0.02	-0.26	0.64	0.79	
NCU02572	U5 small nuclear ribonucleoprotein component	12.67	13.35	13.06	16.04		0.68	2.98	0.39	2.69		0.66	2.85	0.11	2.30	0.79	

NCU00040	eukaryotic translation initiation factor 3 110 kDa subunit	20.02	20.03	19.80	20.60	0.01	0.80	-0.22	0.57	-0.01	0.67	-0.50	0.18	0.79
NCU06101	hypothetical protein	14.75	14.42	13.47	16.61	-0.33	3.14	-1.28	2.19	-0.35	3.01	-1.56	1.80	0.79
NCU08677	hypothetical protein	17.38	17.49	18.11	17.44	0.11	-0.67	0.73	-0.05	0.09	-0.80	0.45	-0.44	0.79
NCU08003	RSC complex subunit	16.50	15.86	16.25	15.87	-0.64	-0.38	-0.25	0.01	-0.66	-0.51	-0.53	-0.38	0.78
NCU07723	norsolorinic acid reductase	16.72	16.35	17.06	17.05	-0.37	-0.01	0.34	0.70	-0.39	-0.14	0.06	0.31	0.78
NCU06652	S	21.45	22.09	22.23	22.58	0.64	0.35	0.78	0.49	0.62	0.22	0.50	0.10	0.78
NCU07987	cystathionine beta-lyase	16.50	17.19	15.90	17.12	0.69	1.22	-0.60	-0.07	0.67	1.09	-0.88	-0.46	0.77
NCU05542	xanthine phosphoribosyltransferase 1	17.21	17.93	17.46	17.76	0.72	0.30	0.25	-0.17	0.70	0.17	-0.03	-0.56	0.77
NCU02840	regulatory particle, ATPase-like-1	18.27	18.37	19.41	18.21	0.10	-1.20	1.14	-0.16	0.08	-1.33	0.86	-0.55	0.77
NCU06348	myo-inositol-1-monophosphatase	18.09	17.13	18.70	18.30	-0.96	-0.40	0.61	1.17	-0.98	-0.53	0.33	0.78	0.77
NCU01021	eukaryotic translation initiation factor 3 subunit EifCf	18.61	18.92	18.50	19.11	0.31	0.61	-0.11	0.19	0.29	0.48	-0.39	-0.20	0.77
NCU02619	tRNA-dihydrouridine synthase 3	16.36	16.27	15.97	17.59	-0.09	1.62	-0.39	1.32	-0.11	1.49	-0.67	0.93	0.77
NCU07832	pre-mRNA processing splicing factor 8	13.07	14.70	14.58	14.92	1.63	0.34	1.51	0.22	1.61	0.21	1.23	-0.17	0.76
NCU09089	hypothetical protein	16.17	20.05	19.43	20.20	3.88	0.77	3.26	0.15	3.86	0.64	2.98	-0.24	0.76
NCU01666	isoleucine-valine-4	15.97	16.67	15.50	16.58	0.70	1.08	-0.47	-0.09	0.68	0.95	-0.75	-0.48	0.75
NCU09545	methylenetetrahydrofolate reductase 2	15.06	16.85	16.55	16.70	1.79	0.15	1.49	-0.15	1.77	0.02	1.21	-0.54	0.75
NCU09715	alpha,alpha-trehalose-phosphate synthase	19.12	18.33	19.96	19.32	-0.79	-0.64	0.84	0.99	-0.81	-0.77	0.56	0.60	0.74
NCU02207	T-complex protein 1 subunit beta	17.85	18.27	18.44	18.72	0.42	0.28	0.59	0.45	0.40	0.15	0.31	0.06	0.74
NCU15835	hypothetical protein	15.57	15.69	14.11	16.76	0.12	2.65	-1.46	1.07	0.10	2.52	-1.74	0.68	0.74
NCU03436	tangerine	15.07	15.92	15.20	15.66	0.85	0.46	0.13	-0.26	0.83	0.33	-0.15	-0.65	0.74
NCU09371	6,7-dimethyl-8-ribityllumazine synthase	17.84	18.40	18.15	18.38	0.56	0.23	0.31	-0.02	0.54	0.10	0.03	-0.41	0.74
NCU01083	spermidine-2	16.76	15.41	15.99	15.71	-1.35	-0.28	-0.77	0.30	-1.37	-0.41	-1.05	-0.09	0.74
NCU04016	phosphoglycerate mutase	16.14	16.14	17.32	16.07	0.00	-1.25	1.18	-0.07	-0.02	-1.38	0.90	-0.46	0.74
NCU11259	hypothetical protein	15.60	16.13	14.81	16.32	0.53	1.51	-0.79	0.19	0.51	1.38	-1.07	-0.20	0.73
NCU01420	hypothetical protein	16.84	16.68	17.60	15.50	-0.16	-2.10	0.76	-1.18	-0.18	-2.23	0.48	-1.57	0.73
NCU06943	SIK1	17.49	17.43	16.61	18.84	-0.06	2.23	-0.88	1.41	-0.08	2.10	-1.16	1.02	0.73
NCU00364	hypothetical protein	16.84	17.17	16.55	17.41	0.33	0.86	-0.29	0.24	0.31	0.73	-0.57	-0.15	0.73
NCU06003	mannose-1-phosphate guanyltransferase	19.05	19.08	19.55	19.26	0.03	-0.29	0.50	0.18	0.01	-0.42	0.22	-0.21	0.73
NCU00480	ubiquitin C-terminal hydrolase	15.41	14.27	15.51	15.49	-1.14	-0.02	0.10	1.22	-1.16	-0.15	-0.18	0.83	0.73
NCU16822	hypothetical protein	19.56	19.65	19.37	20.15	0.09	0.78	-0.19	0.50	0.07	0.65	-0.47	0.11	0.73
NCU04086	hydrolase	16.84	17.49	17.59	18.24	0.65	0.65	0.75	0.75	0.63	0.52	0.47	0.36	0.73
NCU01221	60S ribosomal protein L16	21.34	20.14	21.00	21.16	-1.20	0.16	-0.34	1.02	-1.22	0.03	-0.62	0.63	0.73
NCU05983	3,4-dihydroxy-2-butanone 4-phosphate synthase	18.26	17.29	18.10	18.24	-0.97	0.14	-0.16	0.95	-0.99	0.01	-0.44	0.56	0.72
NCU00443	ran-specific GTPase-activating protein 1	21.08	21.59	21.49	21.65	0.51	0.16	0.41	0.06	0.49	0.03	0.13	-0.33	0.71
NCU04592	3-oxoacyl	17.61	18.56	18.46	18.61	0.95	0.15	0.85	0.05	0.93	0.02	0.57	-0.34	0.71
NCU06112	glutamate decarboxylase	18.03	18.94	18.26	18.67	0.91	0.41	0.23	-0.27	0.89	0.28	-0.05	-0.66	0.70
NCU02480	short-chain dehydrogenase/reductase	20.15	20.58	20.66	20.80	0.43	0.14	0.51	0.22	0.41	0.01	0.23	-0.17	0.69
NCU03358	ketoreductase	18.33	18.74	18.85	19.51	0.41	0.66	0.52	0.77	0.39	0.53	0.24	0.38	0.69
NCU08330	hypothetical protein	20.50	20.21	20.61	20.23	-0.29	-0.38	0.11	0.02	-0.31	-0.51	-0.17	-0.37	0.68
NCU01612	pre-mRNA splicing factor ATP-dependent RNA helicase PRP43	12.90	13.48	13.24	15.11	0.58	1.87	0.34	1.63	0.56	1.74	0.06	1.24	0.68
NCU01956	encodes anonymous transcript-2	16.83	14.62	16.15	16.18	-2.21	0.03	-0.68	1.56	-2.23	-0.10	-0.96	1.17	0.68
NCU08275	aromatic-L-amino-acid decarboxylase	16.71	17.35	17.41	17.66	0.64	0.25	0.70	0.31	0.62	0.12	0.42	-0.08	0.68
NCU06340	TPR repeat protein	16.43	16.13	16.55	16.10	-0.30	-0.45	0.12	-0.03	-0.32	-0.58	-0.16	-0.42	0.68
NCU07808	hypothetical protein	17.43	17.55	17.25	18.72	0.12	1.47	-0.18	1.17	0.10	1.34	-0.46	0.78	0.67
NCU07367	regulatory particle, ATPase-like-4	19.20	18.36	19.71	19.35	-0.84	-0.36	0.51	0.99	-0.86	-0.49	0.23	0.60	0.66
NCU06210	hypothetical protein	21.04	20.38	20.90	20.93	-0.66	0.03	-0.14	0.55	-0.68	-0.10	-0.42	0.16	0.65
NCU00194	ornithine transaminase	21.43	21.66	21.24	22.05	0.23	0.81	-0.19	0.39	0.21	0.68	-0.47	0.00	0.65
NCU03936	tRNA methyltransferase	13.56	15.20	14.84	16.86	1.64	2.02	1.28	1.66	1.62	1.89	1.00	1.27	0.65
NCU04323	dihydroorotase	19.10	19.39	18.57	19.93	0.29	1.36	-0.53	0.54	0.27	1.23	-0.81	0.15	0.65
NCU08936	clock-controlled gene-15	17.97	18.65	17.12	18.88	0.68	1.76	-0.85	0.23	0.66	1.63	-1.13	-0.16	0.64
NCU00463	kynureninase	18.36	18.03	18.92	17.34	-0.33	-1.58	0.56	-0.69	-0.35	-1.71	0.28	-1.08	0.64
NCU09602	heat shock protein 70-1	24.82	24.48	24.96	25.05	-0.34	0.09	0.14	0.57	-0.36	-0.04	-0.14	0.18	0.64
NCU01843	T-complex protein 1 subunit gamma	19.03	18.98	19.55	19.27	-0.05	-0.28	0.52	0.29	-0.07	-0.41	0.24	-0.10	0.64
NCU08811	arf GTPase-activating protein	15.15	14.36	16.60	14.99	-0.79	-1.61	1.45	0.63	-0.81	-1.74	1.17	0.24	0.63
NCU02003	translation elongation factor-1	24.36	24.85	24.83	25.01	0.49	0.18	0.47	0.16	0.47	0.05	0.19	-0.23	0.63
NCU06457	asparaginyl-tRNA synthetase	20.40	20.89	20.11	21.08	0.49	0.97	-0.29	0.19	0.47	0.84	-0.57	-0.20	0.62
NCU07127	dienelactone hydrolase	16.54	16.17	17.57	16.54	-0.37	-1.03	1.03	0.37	-0.39	-1.16	0.75	-0.02	0.62
NCU03087	diploid state maintenance protein chpA	15.83	17.29	16.73	17.03	1.46	0.30	0.90	-0.26	1.44	0.17	0.62	-0.65	0.62
NCU01433	phosphoprotein phosphatase-1	16.77	17.83	17.74	18.52	1.06	0.78	0.97	0.69	1.04	0.65	0.69	0.30	0.61
NCU02459	DUF52 domain-containing protein	17.21	16.34	18.23	17.21	-0.87	-1.02	1.02	0.87	-0.89	-1.15	0.74	0.48	0.61
NCU03151	peroxisomal membrane protein	21.05	21.64	21.54	21.75	0.59	0.21	0.49	0.11	0.57	0.08	0.21	-0.28	0.60
NCU03768	lysophospholipase	17.67	17.35	18.57	17.71	-0.32	-0.86	0.90	0.36	-0.34	-0.99	0.62	-0.03	0.60
NCU03370	hypothetical protein	14.67	16.43	14.91	15.70	1.76	0.79	0.24	-0.73	1.74	0.66	-0.04	-1.12	0.60
NCU02430	cystathionine gamma-synthase	18.65	18.83	19.00	19.38	0.18	0.38	0.35	0.55	0.16	0.25	0.07	0.16	0.59
NCU07734	zinc finger protein gcs1	15.21	13.86	15.17	15.01	-1.35	-0.16	-0.04	1.15	-1.37	-0.29	-0.32	0.76	0.59
NCU02707	60S ribosomal protein L6	20.94	21.64	21.28	21.61	0.70	0.33	0.34	-0.03	0.68	0.20	0.06	-0.42	0.59
NCU01486	IKI3 family protein	16.22	16.74	16.81	17.35	0.52	0.54	0.59	0.61	0.50	0.41	0.31	0.22	0.59
NCU11173	thiamine-phosphate pyrophosphorylase	17.32	17.81	17.90	18.31	0.49	0.41	0.58	0.50	0.47	0.28	0.30	0.11	0.59
NCU04642	adenosine deaminase	16.24	15.48	16.03	15.86	-0.76	-0.17	-0.21	0.38	-0.78	-0.30	-0.49	-0.01	0.58
NCU01328	transketolase	24.70	24.39	24.78	24.61	-0.31	-0.17	0.08	0.22	-0.33	-0.30	-0.20	-0.17	0.58
NCU09269	ran-like	21.78	21.65	22.10	21.75	-0.13	-0.35	0.32	0.10	-0.15	-0.48	0.04	-0.29	0.58
NCU00582	cryptochrome	15.31	14.12	15.71	15.27	-1.19	-0.44	0.40	1.15	-1.21	-0.57	0.12	0.76	0.58
NCU03972	regulatory particle, non-ATPase-like-7	19.17	18.81	19.30	19.36	-0.36	0.06	0.13	0.55	-0.38	-0.07	-0.15	0.16	0.57
NCU03282	3-hydroxyanthranilate 3,4-dioxygenase	19.35	19.15	19.68	19.14	-0.20	-0.54	0.33	-0.01	-0.22	-0.67	0.05	-0.40	0.57
NCU01667	arginine-12	17.01	18.32	17.98	18.41	1.31	0.43	0.97	0.09	1.29	0.30	0.69	-0.30	0.57
NCU00147	ARD/ARD family protein	19.75	19.69	20.10	19.91	-0.06	-0.19	0.35	0.22	-0.08	-0.32	0.07	-0.17	0.56
NCU06743	hypothetical protein	20.50	20.83	20.46	21.10	0.33	0.64	-0.04	0.27	0.31	0.51	-0.32	-0.12	0.56
NCU06876	adenosine 5'-monophosphoramidase	16.64	17.17	17.04	18.18	0.53	1.14	0.40	1.01	0.51	1.01	0.12	0.62	0.56
NCU06821	CRO1 protein	16.07	14.76	15.61	15.35	-1.31	-0.26	-0.46	0.59	-1.33	-0.39	-0.74	0.20	0.55
NCU01444	rheb small monomeric GTPase RhbA	16.24	16.15	16.54	16.36	-0.09	-0.18	0.30	0.21	-0.11	-0.31	0.02	-0.18	0.55
NCU07539	hypothetical protein	18.25	16.89	18.07	17.89	-1.36	-0.18	-0.18	1.00	-1.38	-0.31	-0.46	0.61	0.54
NCU06360	histidinol-phosphate aminotransferase	19.69	19.64	20.06	19.90	-0.05	-0.16	0.37	0.26	-0.07	-0.29	0.09	-0.13	0.54
NCU05301	methyltransferase	19.46	20.40	20.11	20.45	0.94	0.34	0.65	0.05	0.92	0.21	0.37	-0.34	0.53
NCU08666	HAD superfamily hydrolase	15.73	16.59	16.50	17.19	0.86	0.69	0.77	0.60	0.84	0.56	0.49	0.21	0.53
NCU06226	60S ribosomal protein L25	20.49	21.14	19.90	21.57	0.65	1.67	-0.59	0.43	0.63	1.54	-0.87	0.04	0.52
NCU01552	ribosomal protein S28	18.82	19.50	19.16	19.53	0.68	0.37	0.34	0.03	0.66	0.24	0.06	-0.36	0.51
NCU07001	methionine-5	17.15	16.28	16.93	16.40	-0.87	-0.53	-0.22	0.12	-0.89	-0.66	-0.50	-0.27	0.51
NCU01701	ribitol kinase	17.30	17.49	17.29	18.02	0.19	0.73	-0.01	0.53	0.17	0.60	-0.29	0.14	0.51
NCU05137	non-anchored cell wall protein-1	21.65	21.36	22.12	21.26	-0.29	-0.86	0.47	-0.10	-0.31	-0.99	0.19	-0.49	0.51
NCU08952	translation regulator GCD7	16.09	16.58	15.69	17.62	0.49	1.93	-0.40	1.04	0.47	1.80	-0.68	0.65	0.50
NCU09730	kinesin-1	16.94	15.95	18.05	16.67	-0.99	-1.38	1.11	0.72	-1.01	-1.51	0.83	0.33	0.50
NCU03241	FK506-resistant-4	17.51	18.40	16.80	18.73	0.89	1.93	-0.71	0.33	0.87	1.80	-0.99	-0.06	0.49
NCU06863	histone H1	15.06	15.63	14.81	15.89	0.57	1.08	-0.25	0.26	0.55	0.95	-0.53	-0.13	0.49
NCU05850	rubredoxin-NA	20.85	20.17	21.26	20.94	-0.68	-0.32	0.41	0.77	-0.70	-0.45	0.13	0.38	0.49
NCU05526	lysine-5	21.32	21.06	21.95	21.39	-0.26	-0.56	0.63	0.33	-0.28	-0.69	0.35	-0.06	0.48
NCU05804	60S ribosomal protein L19	21.49	21.91	21.94	22.19	0.42	0.25	0.45	0.28	0.40	0.12	0.17	-0.11	0.48
NCU05295	proteasome catalytic alpha-5	19.74	18.89	19.76	19.62	-0.85	-0.14	0.02	0.73	-0.87	-0.27	-0.26	0.34	0.47
NCU08002	carnitine acetyl transferase	17.78	17.18	17.75	17.34	-0.60	-0.41	-0.03	0.16	-0.62	-0.54	-0.31	-0.23	0.47
NCU08046	eukaryotic translation initiation factor 3	17.42	16.86	17.40	17.09	-0.56	-0.31	-0.02	0.23	-0.58	-0.44	-0.30	-0.16	0.46

NCU07380	eukaryotic translation initiation factor 3	18.57	18.79	18.82	19.41		0.22	0.59	0.25	0.62		0.20	0.46	-0.03	0.23	0.46	
NCU00867	hypothetical protein	16.54	15.09	16.81	16.23		-1.45	-0.58	0.27	1.14		-1.47	-0.71	-0.01	0.75	0.46	
NCU04648	glutamine-dependent NA	17.37	17.82	17.64	18.67		0.45	1.03	0.27	0.85		0.43	0.90	-0.01	0.46	0.46	
NCU08960	hypothetical protein	19.95	20.64	20.22	20.69		0.69	0.47	0.27	0.05		0.67	0.34	-0.01	-0.34	0.45	
NCU00915	aspartyl-tRNA synthetase	21.66	21.85	21.80	22.20		0.19	0.40	0.14	0.35		0.17	0.27	-0.14	-0.04	0.44	
NCU11365	aminotransferase	19.21	19.76	19.27	19.91		0.55	0.64	0.06	0.15		0.53	0.51	-0.22	-0.24	0.43	
NCU00464	60S ribosomal protein L32	20.58	21.74	20.85	21.57		1.16	0.72	0.27	-0.17		1.14	0.59	-0.01	-0.56	0.43	
NCU03870	hypothetical protein	17.83	17.23	18.53	17.79		-0.60	-0.74	0.70	0.56		-0.62	-0.87	0.42	0.17	0.43	
NCU09536	hypothetical protein	17.00	17.73	16.55	18.32		0.73	1.77	-0.45	0.59		0.71	1.64	-0.73	0.20	0.42	
NCU03988	60S ribosomal protein L18	18.89	19.38	18.84	19.65		0.49	0.81	-0.05	0.27		0.47	0.68	-0.33	-0.12	0.42	
NCU07352	hypothetical protein	19.11	18.33	19.38	19.09		-0.78	-0.29	0.27	0.76		-0.80	-0.42	-0.01	0.37	0.42	
NCU08423	bromodomain protein-3	15.55	14.09	15.18	14.65		-1.46	-0.53	-0.37	0.56		-1.48	-0.66	-0.65	0.17	0.41	
NCU07459	protein arginine N-methyltransferase-1	18.72	19.04	18.88	19.30		0.32	0.42	0.16	0.26		0.30	0.29	-0.12	-0.13	0.41	
NCU06687	glycogen synthase-1	20.32	20.03	20.47	20.30		-0.29	-0.17	0.15	0.27		-0.31	-0.30	-0.13	-0.12	0.40	
NCU05667	anchored cell wall protein-3	18.04	16.56	19.36	15.69		-1.48	-3.67	1.32	-0.87		-1.50	-3.80	1.04	-1.26	0.40	
NCU10073	actin binding protein	17.30	16.75	17.46	16.75		-0.55	-0.71	0.16	0.00		-0.57	-0.84	-0.12	-0.39	0.40	
NCU03200	serine/threonine protein kinase-10	16.24	15.21	16.28	15.98		-1.03	-0.30	0.04	0.77		-1.05	-0.43	-0.24	0.38	0.40	
NCU03100	pentose phosphate metabolism-2	25.42	25.10	25.57	25.34		-0.32	-0.23	0.15	0.24		-0.34	-0.36	-0.13	-0.15	0.39	
NCU06232	leucine-1	20.95	20.90	21.21	21.25		-0.05	0.04	0.26	0.35		-0.07	-0.09	-0.02	-0.04	0.37	
NCU00505	small nuclear ribonucleoprotein Sm D1	16.56	17.08	16.45	17.57		0.52	1.12	-0.11	0.49		0.50	0.99	-0.39	0.10	0.36	
NCU08944	N-acetyltransferase complex ARD1 subunit	16.99	18.14	16.65	18.41		1.15	1.76	-0.34	0.27		1.13	1.63	-0.62	-0.12	0.32	
NCU04118	aspartokinase	16.33	16.89	16.46	17.61		0.56	1.15	0.13	0.72		0.54	1.02	-0.15	0.33	0.31	
NCU03234	ubr1-like	13.64	16.05	14.06	15.59		2.41	1.53	0.42	-0.46		2.39	1.40	0.14	-0.85	0.31	
NCU07776	anchored cell wall protein-5	16.84	18.83	17.84	19.25		1.99	1.41	1.00	0.42		1.97	1.28	0.72	0.03	0.31	
NCU09783	D-isomer specific 2-hydroxyacid dehydrogenase	16.28	17.65	16.16	17.61		1.37	1.45	-0.12	-0.04		1.35	1.32	-0.40	-0.43	0.31	
NCU07814	DUF89 domain-containing protein	17.59	18.21	18.02	18.83		0.62	0.81	0.43	0.62		0.60	0.68	0.15	0.23	0.31	
NCU03084	inosine-uridine preferring nucleoside hydrolase	19.96	19.79	20.20	20.23		-0.17	0.03	0.24	0.44		-0.19	-0.10	-0.04	0.05	0.31	
NCU06031	rehydrin	22.32	22.58	22.56	23.11		0.26	0.55	0.24	0.53		0.24	0.42	-0.04	0.14	0.30	
NCU02542	embden-meyerhof pathway-1	22.03	21.58	22.14	21.96		-0.45	-0.18	0.11	0.38		-0.47	-0.31	-0.17	-0.01	0.30	
NCU09572	ARP2/3 complex 21 kDa subunit	17.61	18.14	17.88	18.80		0.53	0.92	0.27	0.66		0.51	0.79	-0.01	0.27	0.29	
NCU00838	3-dehydroshikimate dehydratase	15.88	14.77	15.91	14.74		-1.11	-1.17	0.03	-0.03		-1.13	-1.30	-0.25	-0.42	0.28	
NCU09469	EF hand domain-containing protein	16.77	17.37	16.98	17.56		0.60	0.58	0.21	0.19		0.58	0.45	-0.07	-0.20	0.28	
NCU05488	RNA-binding protein Vip1	21.20	20.37	21.21	20.60		-0.83	-0.61	0.01	0.23		-0.85	-0.74	-0.27	-0.16	0.28	
NCU00112	aminopeptidase P	18.31	16.83	18.16	16.89		-1.48	-1.27	-0.15	0.06		-1.50	-1.40	-0.43	-0.33	0.26	
NCU02839	T-complex protein 1	18.31	18.82	18.66	19.39		0.51	0.73	0.35	0.57		0.49	0.60	0.07	0.18	0.25	
NCU06099	hypothetical protein	16.29	17.42	16.13	18.09		1.13	1.96	-0.16	0.67		1.11	1.83	-0.44	0.28	0.24	
NCU00295	hypothetical protein	16.39	15.48	17.05	15.87		-0.91	-1.18	0.66	0.39		-0.93	-1.31	0.38	0.00	0.24	
NCU02786	hypothetical protein	19.06	18.84	19.38	19.13		-0.22	-0.25	0.32	0.29		-0.24	-0.38	0.04	-0.10	0.24	
NCU01704	NADP:D-xylose dehydrogenase	18.09	16.54	18.80	17.14		-1.55	-1.66	0.71	0.60		-1.57	-1.79	0.43	0.21	0.20	
NCU00087	pentose phosphate metabolism-1	20.31	20.16	20.62	20.50		-0.15	-0.12	0.31	0.34		-0.17	-0.25	0.03	-0.05	0.19	
NCU09911	formyltetrahydrofolate deformylase	19.89	20.28	20.07	20.63		0.39	0.56	0.18	0.35		0.37	0.43	-0.10	-0.04	0.18	
NCU03539	unknown-24	18.37	16.88	18.86	16.85		-1.49	-2.01	0.49	-0.03		-1.51	-2.14	0.21	-0.42	0.18	
NCU03859	MYG1 protein	17.54	18.26	17.88	18.83		0.72	0.95	0.34	0.57		0.70	0.82	0.06	0.18	0.18	
NCU04759	nuclear transport factor 2	20.32	20.08	20.57	20.51		-0.24	-0.06	0.25	0.43		-0.26	-0.19	-0.03	0.04	0.16	
NCU06578	KapG	19.03	18.57	19.25	19.03		-0.46	-0.22	0.22	0.46		-0.48	-0.35	-0.06	0.07	0.16	
NCU03009	zuotin	19.00	18.16	19.11	18.54		-0.84	-0.57	0.11	0.38		-0.86	-0.70	-0.17	-0.01	0.15	
NCU07608	pentose phosphate metabolism-3	19.37	19.87	19.56	20.21		0.50	0.65	0.19	0.34		0.48	0.52	-0.09	-0.05	0.14	
NCU02813	eukaryotic translation initiation factor 3 subunit M	19.29	18.93	19.63	19.31		-0.36	-0.32	0.34	0.38		-0.38	-0.45	0.06	-0.01	0.11	
NCU04600	protein phosphatase 2C isoform gamma	21.36	20.76	21.61	21.23		-0.60	-0.38	0.25	0.47		-0.62	-0.51	-0.03	0.08	0.11	
NCU08053	hypothetical protein	17.99	16.96	18.13	17.31		-1.03	-0.82	0.14	0.35		-1.05	-0.95	-0.14	-0.04	0.10	
NCU02450	threonine dehydratase	18.85	18.21	19.22	18.62		-0.64	-0.60	0.37	0.41		-0.66	-0.73	0.09	0.02	0.10	
NCU04343	ergothioneine-1	21.33	20.66	21.67	20.97		-0.67	-0.70	0.34	0.31		-0.69	-0.83	0.06	-0.08	0.09	
	Not detected in both replicates of each sample	only in delta-Por - blue; only in WT - yellow															
NCU00865	oxalate decarboxylase oxdC			21.00	21.09												
NCU03559	ubiquinol-cytochrome c reductase complex core protein 2 precursor			19.85	20.10												
NCU00399	cell wall protein PhiA			20.08	19.84												
NCU10521	glutathione S-transferase-4			19.22	20.05												
NCU07053	aldehyde dehydrogenase			19.17	19.49												
NCU02955	elongation factor G 1			18.63	17.88												
NCU11291	hypothetical protein			17.85	18.59												
NCU09808	dynamin-1			17.49	18.87												
NCU09290	proteasome catalytic beta-1			18.35	18.00												
NCU04697	cyanide hydratase			17.39	18.42												
NCU07456	riboflavin-5			17.63	18.03												
NCU09119	ATP synthase subunit gamma			18.08	17.38												
NCU09024	hypothetical protein			18.05	17.39												
NCU05986	sucrase/ferredoxin domain-containing protein			17.12	18.14												
NCU04314	hypothetical protein			17.30	17.85												
NCU09897	vacuolar membrane ATPase-5			17.26	17.34												
NCU01765	NADH:ubiquinone oxidoreductase 78			17.96	16.63												
NCU01906	GCY protein			16.75	17.76												
NCU09537	rho-GTPase-activating protein 8			17.44	16.93												
NCU07257	F-box domain-containing protein			16.84	17.41												
NCU01213	superoxide dismutase-2			17.45	16.56												
NCU07468	NAD dependent epimerase/dehydratase			17.10	16.69												
NCU03526	ubiquitin-activating enzyme E1 3			16.50	17.25												
NCU01347	hypothetical protein			16.47	17.08												
NCU03979	biotin-1			16.43	17.08												
NCU02482	tricarboxylic acid-2			17.03	16.45												
NCU05841	UMTA			16.63	16.84												
NCU08117	hypothetical protein			16.09	17.22												
NCU06653	hypothetical protein			16.39	16.75												
NCU06142	hypothetical protein			17.10	15.97												
NCU09409	arylesterase/monooxygenase			16.64	16.42												
NCU09705	GAL10			16.29	16.73												
NCU05419	hydroxymethylglutaryl-CoA lyase			16.15	16.85												
NCU07296	colonial temperature sensitive-1			17.25	15.68												
NCU01200	peptidyl-prolyl cis-trans isomerase B			15.96	16.93												
NCU01797	nonrepressor of conidiation-2			16.16	16.53												
NCU00137	ssDNA binding protein			16.92	15.73												
NCU03793	hypothetical protein			16.32	16.30												
NCU03617	haloacid dehalogenase			15.64	16.93												
NCU00395	mRNA processing protein			16.22	16.34												
NCU11424	carotenoid oxygenase-2			15.44	17.03												
NCU03732	SIS1			15.25	17.20												
NCU06303	alcohol dehydrogenase			16.64	15.78												
NCU02962	hypothetical protein			16.75	15.66												
NCU03662	hypothetical protein			15.80	16.51												

NCU04044	NADH:ubiquinone oxidoreductase 51kD subunit			16.24	16.00												
NCU08005	NADPH-adrenodoxin reductase Arh1			16.07	16.11												
NCU02555	actin-related protein-4			16.57	15.56												
NCU08035	vacuolar ATPase subunit D			15.58	16.51												
NCU07925	LRP16			15.90	16.08												
NCU01376	phosphorus-2			16.43	15.52												
NCU07280	serine/threonine protein kinase-50			16.75	15.14												
NCU04098	monothiol glutaredoxin-5			16.16	15.72												
NCU01753	gamma-glutamyltranspeptidase			15.96	15.85												
NCU16560	hypothetical protein			16.07	15.63												
NCU06205	regulator of conidiation-1			16.16	15.52												
NCU06576	hypothetical protein			15.62	16.05												
NCU01118	hypothetical protein			16.34	15.10												
NCU05185	bifunctional P-450:NADPH-P450 reductase			15.88	15.49												
NCU00437	nitrilase			14.58	16.75												
NCU02283	calcium/calmodulin-dependent kinase-2			15.78	15.54												
NCU02954	lysine-7			17.00	14.23												
NCU01512	phenylalanyl-tRNA synthetase			15.58	15.60												
NCU06240	protein kinase A catalytic subunit-1			15.07	16.10												
NCU06199	RNA binding protein Jsn1			14.82	16.31												
NCU03814	leucine-5			15.81	15.30												
NCU09721	AP-1 complex subunit beta-1			15.85	15.24												
NCU09532	L-threo-3-deoxy-hexulosonate aldolase			15.72	15.23												
NCU09283	acetyltransferase			15.73	15.17												
NCU02597	hypothetical protein			15.48	15.41												
NCU06966	hypothetical protein			16.20	14.68												
NCU06270	mitochondrial processing peptidase			14.59	16.27												
NCU00465	chaperone dnaJ			15.42	15.35												
NCU04281	WD-repeat protein pop3			15.05	15.65												
NCU03112	NADH-cytochrome b5 reductase 2			16.71	13.94												
NCU02685	pre-mRNA splicing helicase			14.64	15.99												
NCU04039	GNAT family N-acetyltransferase			14.56	16.01												
NCU09535	hypothetical protein			15.13	15.40												
NCU05539	2',3'-cyclic-nucleotide 3'-phosphodiesterase			15.37	15.14												
NCU07849	thiamine-4			15.98	14.52												
NCU05248	hypothetical protein			15.16	15.34												
NCU02481	mitochondrial 2-methylisocitrate lyase			15.71	14.79												
NCU07733	electron transfer flavoprotein beta-subunit			14.92	15.57												
NCU06695	cytochrome oxidase-6			15.33	15.13												
NCU00050	pyruvate dehydrogenase X component			14.78	15.64												
NCU01327	hypothetical protein			14.99	15.39												
NCU05188	FAD monooxygenase			15.71	14.60												
NCU08898	lysine-6			14.65	15.62												
NCU06401	hypothetical protein			15.01	15.23												
NCU05553	hypothetical protein			15.12	15.09												
NCU07859	cyclopropane-fatty-acyl-phospholipid synthase			15.21	14.90												
NCU00879	WD domain-containing protein			15.10	14.97												
NCU00378	aldehyde dehydrogenase			13.76	16.26												
NCU03303	hypothetical protein			16.21	13.77												
NCU00566	synaptobrevin			14.88	15.09												
NCU00581	phosphopantetheinyl transferase			14.45	15.48												
NCU03714	thioredoxin			16.38	13.51												
NCU00831	serine carboxypeptidase			15.09	14.76												
NCU02630	heat shock protein 78			16.89	12.94												
NCU00918	hypothetical protein			13.86	15.91												
NCU05574	hypothetical protein			14.43	15.33												
NCU03229	translocase of inner mitochondrial membrane 44			15.41	14.30												
NCU04247	ropy-4			15.02	14.67												
NCU02004	phosphoserine phosphatase			14.91	14.74												
NCU03566	short chain dehydrogenase/reductase			15.06	14.59												
NCU09263	anchored cell wall protein-4			14.89	14.74												
NCU09485	chaperone dnaK			14.60	14.91												
NCU06419	MAPK/ERK kinase			15.80	13.68												
NCU02495	CwfJ domain-containing protein			15.28	14.18												
NCU04121	AP-1 complex subunit gamma-1			14.60	14.81												
NCU07547	60S acidic ribosomal protein P0			15.06	14.35												
NCU04245	translocase of outer mitochondrial membrane 70			15.28	14.11												
NCU04945	mitochondrial intermembrane space protein Mia40			14.87	14.51												
NCU02578	paired amphipathic helix protein Sin3a			15.67	13.70												
NCU08299	60S ribosomal protein L3			14.29	15.01												
NCU09139	tubulin-specific chaperone			15.02	14.28												
NCU06976	ropy-1			14.63	14.65												
NCU07453	hypothetical protein			14.34	14.84												
NCU02573	FAM96B			15.60	13.53												
NCU06349	hypothetical protein			13.81	15.25												
NCU06017	thiosulfate sulfurtransferase			14.77	14.28												
NCU06593	MAP kinase activator			15.58	13.43												
NCU02349	UDP-glucose:glycoprotein glucosyltransferase			14.29	14.61												
NCU06404	GTP-binding protein SAS1			14.59	14.26												
NCU12046	gamma-butyrobetaine dioxygenase			14.46	14.26												
NCU07019	COP9 signalosome-6			14.15	14.46												
NCU06534	hypothetical protein			14.19	14.38												
NCU09980	DUF255 domain-containing protein			13.72	14.85												
NCU07773	thymidylate kinase			14.58	13.95												
NCU06284	vacuolar protein sorting-associated protein 35			14.17	14.32												
NCU04252	SNARE docking complex subunit			13.86	14.45												
NCU01190	DUF410 domain-containing protein			13.38	14.83												
NCU11368	5',5'''-P-1,P-4-tetraphosphate phosphorylase 2			14.22	13.93												
NCU01427	albino-3			13.90	14.18												
NCU08132	alpha-1,3-glucan synthase Ags2			13.78	14.29												
NCU00551	myo2-like			14.00	14.00												
NCU08296	30S ribosomal subunit S4			12.19	15.51												
NCU09703	acetolactate decarboxylase			14.39	13.27												
NCU08045	choline-1			14.48	13.00												
NCU01179	translocase of outer mitochondrial membrane 40			13.83	13.61												
NCU07895	hypothetical protein			14.02	13.32												
NCU01878	vesicle-mediated transporter			14.46	12.81												
NCU02695	hypothetical protein			12.09	15.10												
NCU05802	ATP-dependent RNA helicase A			13.61	13.17												

NCU09438	translation repressor/antiviral protein Ski3			13.82	12.58												
NCU02353	hypothetical protein			12.95	13.38												
NCU03042	U2 snRNP component prp10			12.93	13.17												
NCU11323	hypothetical protein			12.74	13.20												
NCU01844	hypothetical protein			13.25	12.56												
NCU03314	mob2-like-a			12.41	13.13												
NCU07474	ergot alkaloid biosynthetic protein A			12.83	12.23												
NCU04017	heterochromatin protein	15.95	15.16														
NCU01720	hypothetical protein	16.79	16.79														
NCU02520	thioredoxin	14.73	14.10														
NCU03981	elongation factor Tu GTP binding domain-containing protein	14.94	15.40														
NCU03126	hypothetical protein	16.41	17.11														
NCU02961	exonuclease	15.43	14.89														
NCU03315	hypothetical protein	14.31	13.01														
NCU02388	hypothetical protein	14.64	15.54														
NCU00309	WSC domain-containing protein	14.22	14.55														
NCU00419	DNA-directed RNA polymerase I and III polypeptide	14.83	14.86														
NCU02236	hypothetical protein	16.37	15.19														
NCU16790	hypothetical protein	16.10	14.46														
NCU09436	alternative chromatin assembly factor-1	17.73	17.60														
NCU05518	peroxisomal copper amine oxidase	16.49	16.58														
NCU08385	protein phosphatase PP1 regulatory subunit sds22	14.19	13.69														
NCU07253	1,3-beta-glucanosyltransferase gel1	15.26	14.86														
NCU03588	FPD1	16.62	15.90														
NCU01164	transcription elongation complex subunit	16.49	16.99														
NCU02244	C2 domain-containing protein	14.67	14.53														
NCU06104	RLI and DUF367 domain-containing protein	14.13	14.19														
NCU04328	metaphase-anaphase transition protein	14.23	13.63														
NCU04409	ankyrin repeat protein	15.86	15.49														
NCU08131	glycosylhydrolase family 13-1	14.46	13.26														
NCU05285	C2H2 transcription factor RfeC	15.82	14.94														
NCU09203	transcription factor iws-1	13.83	13.99														
NCU06446	hypothetical protein	15.56	15.71														
NCU03886	PH domain-containing protein	13.83	13.47														
NCU09708	PDCD2 C terminal domain-containing protein	16.55	15.53														
NCU02508	hypothetical protein	16.03	16.05														
NCU01904	2-deoxy-D-gluconate 3-dehydrogenase	14.71	14.80														
NCU08505	DUF866 domain-containing protein	14.97	15.40														
NCU00366	eukaryotic translation initiation factor 5	17.24	17.15														
NCU07466	cystathionine beta-synthase	15.59	15.56														
NCU07714	hypothetical protein	14.74	15.70														
NCU03696	nitroreductase	15.30	15.00														
NCU04048	hypothetical protein	12.83	12.49														
NCU05169	catalase-4	15.09	15.34														
NCU05306	hypothetical protein	14.90	16.09														
NCU05706	glutathione S-transferase	14.77	15.56														
NCU00089	MYND domain-containing protein	15.82	14.04														
NCU07110	hypothetical protein	13.97	13.30														
NCU07568	hypothetical protein	13.02	13.23														
NCU08355	hypothetical protein	14.96	14.45														
NCU00571	hypothetical protein	15.37	15.32														
NCU02729	transducin family protein	15.11	15.75														
NCU07952	C2H2 type zinc finger domain-containing protein	15.15	14.43														
NCU03415	aldehyde dehydrogenase	14.55	14.82														
NCU09378	DNA-directed RNA polymerase II polypeptide	14.82	16.20														
NCU10498	60S ribosomal protein L35	18.65	19.49														
NCU04227	hypothetical protein	13.75	14.85														
NCU03738	40S ribosomal protein S29	18.35	18.78														
NCU00617	phosducin family protein	16.17	14.35														
NCU00212	histone h4-2	17.44	19.01														
NCU02005	phosphoadenosine phosphosulfate reductase	17.07	16.66														
NCU08324	DNA-binding protein HGH1	15.89	15.45														
NCU04363	hypothetical protein	12.28	14.00														
NCU02540	meiotic expression up-regulated protein 14	13.73	14.79														
NCU08769	conidiation-6	16.70	17.17														
NCU08268	TOR signaling pathway regulator	13.98	14.58														
NCU06123	phosphoketolase	16.29	16.93														
NCU01705	tRNA methyltransferase	15.91	14.78														
NCU01215	thioesterase	14.74	14.23														
NCU11279	glutamyl-tRNA synthetase	15.50	15.69														
NCU02477	ribosome biogenesis protein Sqt1	15.82	16.15														
NCU09227	kelch repeat-containing protein	15.93	14.32														
NCU03302	60S ribosomal protein L36	21.72															
NCU07495	sphingolipid long chain base-responsive protein LSP1	15.71	15.70														
NCU04554	glycosylhydrolase family 18-5	14.43	12.39														
NCU09521	ribosome biogenesis protein	16.74	16.67														
NCU09504	hypothetical protein	16.09	17.26														
NCU08228	UDP-glucose dehydrogenase	13.51	14.91														
NCU08678	translation machinery-associated protein 20	15.77	16.08														
Proteins not detected in both replicates of WT and dPOR																	
NCU08990	60S ribosomal protein L39	20.57		20.24	20.60												
NCU04334	chaperonin	15.39		19.20	18.99												
NCU07787	clock-controlled gene-14	16.19		18.69	19.34												
NCU03297	cytochrome c peroxidase		16.31	18.64	18.83												
NCU00451	hypothetical protein	14.47		18.67	18.34												
NCU03777	mitochondrial 3-hydroxyisobutyryl-CoA hydrolase		14.46	18.43	18.44												
NCU00293	NADH pyrophosphatase		15.31	18.43	18.17												
NCU09267	copper radical oxidase		13.39	18.32	18.20												
NCU03749	hydroxyacylglutathione hydrolase		17.48	17.68	18.60												
NCU02595	hypothetical protein		16.41	18.25	17.66												
NCU07585	siroheme synthase	17.32		17.59	17.89												
NCU05345	zearalenone lactonase		17.58	18.14	17.22												
NCU04063	protein transporter sec-13		16.16	18.38	16.92												
NCU01728	6-phosphofructo-2-kinase	14.80		17.28	17.91												
NCU08333	bleomycin hydrolase		14.52	17.58	17.54												
NCU00104	heat shock protein 98		14.97	16.75	17.95												
NCU01674	alpha-soluble NSF attachment protein		16.21	17.46	17.05												
NCU06009	oxidoreductase	14.46		16.72	17.77												

NCU01339	hypothetical protein	13.92		17.75	16.68
NCU04380	acyl-CoA synthetase		14.82	17.54	16.85
NCU02610	dynein light chain type 1	14.19		17.69	16.68
NCU06877	CRAL/TRIO domain-containing protein	14.06		16.93	17.14
NCU06989	hypothetical protein	14.78		17.38	16.62
NCU11181	Ras superfamily GTPase		16.19	16.53	17.35
NCU05118	vacuolar membrane ATPase-10	14.79		17.35	16.53
NCU05234	GTP-binding protein ryh1	15.30		17.25	16.42
NCU01441	inosine triphosphate pyrophosphatase	16.04		16.59	17.07
NCU04059	hypothetical protein		15.53	16.38	17.21
NCU02738	peflin-1	15.42		16.67	16.86
NCU04905	hypothetical protein	14.83		17.33	16.18
NCU06854	RuvB-like helicase 2	16.17		16.74	16.72
NCU00088	4-nitrophenylphosphatase		17.17	16.32	17.11
NCU07064	L-galactonate dehydratase		14.59	16.52	16.89
NCU05805	serine hydroxymethyltransferase	14.96		17.14	16.19
NCU08336	tricarboxylic acid-12	14.07		16.32	16.99
NCU09477	ADP, ATP carrier protein	16.45		16.71	16.57
NCU00372	hypothetical protein		13.72	16.13	16.93
NCU07221	high conidial production-1	14.92		16.77	16.22
NCU07361	COP9 signalosome-4	15.18		15.94	16.96
NCU05803	translational activator		14.81	15.38	17.35
NCU00770	hypothetical protein	14.74		15.91	16.62
NCU00891	xylitol dehydrogenase	14.74		16.89	15.64
NCU03515	cell division control protein 10		14.19	16.64	15.85
NCU03625	small nuclear ribonucleoprotein Sm D3		16.37	16.21	16.21
NCU08297	cell division control protein 3		14.41	16.55	15.76
NCU07851	superoxide dismutase 1 copper chaperone	15.80		15.97	16.32
NCU03357	ubiquitin conjugation factor E4		13.76	16.41	15.79
NCU07506	fumarylacetoacetate hydrolase	12.42		17.04	15.15
NCU00468	prephenate dehydrogenase		13.66	16.51	15.68
NCU00170	DNAJ domain-containing protein		15.27	16.83	15.32
NCU02098	G/U mismatch-specific DNA glycosylase	15.44		16.06	16.08
NCU01013	delta-aminolevulinic acid dehydratase	13.51		15.28	16.68
NCU04655	hypothetical protein	15.11		15.65	16.19
NCU00655	mitochondrial enoyl reductase		14.27	15.52	16.24
NCU04176	RNA polymerase II Elongator subunit	15.25		15.30	16.26
NCU09265	calreticulin		12.80	16.39	14.77
NCU02550	alpha-galactosidase		14.24	15.27	15.87
NCU03065	mitotic control protein dis3	16.36		15.02	16.03
NCU07435	chitin biosynthesis protein CHS5	14.36		14.90	16.06
NCU02281	hypothetical protein		14.28	15.41	15.55
NCU02394	oxidation resistance protein 1		13.16	14.19	16.74
NCU00893	folC		14.24	15.21	15.72
NCU01189	hypothetical protein	14.86		15.46	15.31
NCU16888	hypothetical protein	15.07		16.01	14.75
NCU06029	hypothetical protein	14.27		15.56	15.20
NCU04110	branchpoint-bridging protein		13.54	15.21	15.27
NCU01364	oxidoreductase	13.58		15.93	14.54
NCU04775	nicotinamide N-methyltransferase	13.57		15.13	15.31
NCU01549	hypothetical protein		15.42	16.36	14.08
NCU01484	rho-type GTPase-1		14.52	14.03	16.39
NCU09048	hypothetical protein	13.48		15.15	15.21
NCU01703	aldo-keto reductase	14.59		14.96	15.34
NCU00042	selenoprotein domain-containing protein		12.11	14.87	15.41
NCU03893	short-chain dehydrogenase/reductase SDR		14.10	15.49	14.76
NCU09474	acetyltransferase	14.42		15.85	14.30
NCU00334	3' exoribonuclease	13.72		15.35	14.77
NCU00130	glycosylhydrolase family 1-1		14.47	14.82	15.29
NCU02116	metallo-beta-lactamase		12.71	14.79	15.32
NCU00944	threonine-5		14.91	15.52	14.57
NCU00560	WD domain-containing protein		13.92	15.43	14.58
NCU04613	HAD superfamily hydrolase	14.89		16.35	13.48
NCU01766	hypothetical protein	14.20		15.43	14.36
NCU07069	pre-mRNA splicing factor	14.74		14.57	15.19
NCU02532	NEDD8-activating enzyme E1 catalytic subunit	13.95		14.14	15.60
NCU01337	dihydrofolate synthetase Fol3	12.96		14.83	14.79
NCU16624	hypothetical protein		13.29	14.79	14.66
NCU00311	VTS1		13.79	13.88	15.46
NCU02515	dipeptidyl aminopeptidase	14.36		14.81	14.50
NCU01257	hypothetical protein		16.03	14.69	14.51
NCU07878	hypothetical protein		14.72	14.29	14.90
NCU09688	AP-1 complex subunit mu		15.34	13.53	15.64
NCU11129	carboxypeptidase s	15.01		14.91	14.04
NCU09571	tRNA		14.02	13.63	15.18
NCU10927	signal recognition particle protein		13.42	13.41	15.38
NCU05289	nucleolar GTP-binding protein 1	14.03		14.19	14.53
NCU08663	nonsense-mediated mRNA decay protein 3		14.38	14.07	14.64
NCU07726	bcp-1	14.94		13.80	14.89
NCU01758	YdiU domain-containing protein	13.58		14.35	14.34
NCU06856	ubiquitin fusion degradation protein	14.94		13.43	15.07
NCU03366	hypothetical protein		15.04	13.88	14.27
NCU03311	polyadenylation factor subunit CstF64	13.54		14.66	13.42
NCU09648	aldehyde dehydrogenase	14.07		13.77	14.23
NCU08023	cytohesin-2		12.85	13.93	13.95
NCU02218	hypothetical protein	12.63		14.59	13.23
NCU00668	rho guanyl nucleotide exchange factor 1		13.27	13.30	14.50
NCU06530	palmitoyl-protein thioesterase	15.03		13.36	14.39
NCU05680	alpha-1,2-mannosyltransferase Kre5		13.66	13.47	14.22
NCU08380	plasma membrane phosphatase required for sodium stress response	13.23		14.39	13.25
NCU10059	streptomycin biosynthesis protein StrI		13.21	13.20	14.33
NCU07731	hypothetical protein		12.25	13.26	14.23
NCU16400	hypothetical protein		12.62	13.45	13.95
NCU03538	deoxyribonuclease tatD		14.86	13.07	13.84
NCU00272	cullin-4	13.21		13.17	13.13
NCU16656	hypothetical protein	12.80		13.20	12.58
NCU06059	TRAX homolog	13.51		12.28	13.33
NCU08344	60s ribosomal protein	19.55	18.46		20.55
NCU01948	ribosomal protein Srp1	20.00	19.07		20.06

NCU07857	60S ribosomal protein L34		20.14		19.25											
NCU01966	60S ribosomal protein L37	20.36	19.53	19.17												
NCU08907	clock-controlled gene-13				18.64											
NCU00868	hypothetical protein				18.55											
NCU02437	histone H2A	17.52	19.27		18.24											
NCU09693	hypothetical protein	17.88	18.36	17.51												
NCU07120	DUF1264 domain-containing protein	14.64	14.11	17.33												
NCU07954	hypothetical protein	17.79	18.28		16.95											
NCU02134	transcription factor				16.75											
NCU11237	prenylated Rab acceptor 1			16.74												
NCU01889	hypothetical protein			16.73												
NCU07683	small nuclear ribonucleoprotein	16.24	15.53		16.66											
NCU07670	cytochrome-19				16.60											
NCU03753	clock-controlled gene-1	16.65	16.69	16.59												
NCU02227	acetylornithine deacetylase				16.57											
NCU03191	rab protein geranylgeranyltransferase component A	17.07	15.79		16.56											
NCU09732	acetyl-CoA acetyltransferase				16.53											
NCU03579	alanine racemase		15.84		16.48											
NCU17060	DRAP deaminase			16.46												
NCU07452	menadione-induced gene-2	16.07	16.69	16.44												
NCU09013	UPF0047 domain-containing protein			16.42												
NCU08616	unknown-18	16.92	16.57		16.41											
NCU07732	arginine-2			16.34												
NCU04596	gamma-cysteine synthetase regulatory subunit	16.84	16.21		16.32											
NCU06679	chromatin assembly-3	18.03	17.04		16.30											
NCU03607	threonine dehydratase catabolic	14.37		16.27												
NCU05116	phosphoglycerate mutase		16.09		16.25											
NCU09534	peroxiredoxin HYR1	16.32	16.85		16.21											
NCU09727	NADPH-dependent FMN/FAD containing oxidoreductase	14.75	15.91		16.21											
NCU05261	ATP-dependent protease La			16.21												
NCU07421	hypothetical protein		14.39		16.16											
NCU09541	hypothetical protein	15.72			16.15											
NCU01320	glutathione S-transferase-3			16.10												
NCU09311	methionine-10		16.68		16.08											
NCU01515	DNA-directed RNA polymerase II polypeptide	16.05	15.32	16.05												
NCU03207	ran GTPase activating protein 1	19.64	19.72		16.04											
NCU06801	hypothetical protein				16.00											
NCU00385	delta-subunit ATP synthase			15.99												
NCU02358	SDS23			15.97												
NCU10053	thymidylate synthase	16.62	16.19		15.96											
NCU16698	twinfilin-1			15.96												
NCU00444	transcription initiation factor TFIID subunit 14		14.38	15.96												
NCU02372		14.64		15.94												
NCU08661	pantothenic acid-4	13.66	13.86	15.93												
NCU08173	early conidial development-2				15.90											
NCU09611	fructosamine-3-kinase				15.90											
NCU09138	peroxisomal dehydratase	14.74	15.25	15.90												
NCU04264	extracellular developmental signal biosynthesis protein FluG		15.66	15.86												
NCU04051	GCN20	12.53			15.84											
NCU07492	hypothetical protein	14.36	14.12		15.83											
NCU04790	eukaryotic peptide chain release factor GTP-binding subunit	13.46		15.83												
NCU02536	ubiquinone biosynthesis monooxygenase COQ6			15.81												
NCU04015	vacuolar protein sorting-associated protein 27	13.46			15.79											
NCU02228	geranylgeranyl transferase type I beta subunit	14.98			15.75											
NCU02534	NADH:ubiquinone oxidoreductase 49			15.73												
NCU05375	hypothetical protein				15.73											
NCU03321	eukaryotic ribosome biogenesis protein 1				15.73											
NCU00133	FACT complex subunit pob-3	17.33	17.36		15.72											
NCU04047	hypothetical protein	15.98	16.17		15.72											
NCU01178	6-phosphofructo-2-kinase/fructose-2,6-bisphosphatase	15.33	15.32		15.72											
NCU11426	nuclease-2				15.71											
NCU03401	hypothetical protein	15.04			15.71											
NCU05050	2-hydroxyacid dehydrogenase	14.37	14.96	15.70												
NCU02952	hypothetical protein				15.69											
NCU08111	arginine N-methyltransferase 2				15.68											
NCU02780	U2 small nuclear ribonucleoprotein B	15.70	15.16		15.67											
NCU01747	glycerophosphocholine phosphodiesterase Gde1	14.68	15.28		15.64											
NCU06361	cwl1			15.64												
NCU04302	ubiquitin-conjugating enzyme E				15.63											
NCU10777	GMF family protein	16.53	17.51		15.62											
NCU02661	DNA-directed RNA polymerase II polypeptide			15.60												
NCU10388	peptidyl-prolyl cis-trans isomerase-like 3			15.57												
NCU02757	60S ribosomal protein L2				15.54											
NCU02800	arsenite resistance protein Ars2			15.53												
NCU00410	eukaryotic release factor 1	14.50	16.64		15.53											
NCU02712	acetate kinase	15.59	15.16	15.53												
NCU04771	fructosyl-amino acid oxidase	15.20			15.52											
NCU06781	1,3-beta-glucanosyltransferase Gel2	16.15	15.71		15.52											
NCU05989	hypothetical protein				15.51											
NCU05204	cullin-1			15.50												
NCU04196	tRNA		16.22	15.49												
NCU09349	ATP-dependent RNA helicase has-1	14.26			15.48											
NCU08951	H/ACA ribonucleoprotein complex subunit 2				15.45											
NCU04617	hypothetical protein				15.44											
NCU03692	methyltransferase			15.44												
NCU07010	cortical actin cytoskeleton protein asp1	15.18	15.83		15.44											
NCU09058	enoyl-CoA hydratase				15.43											
NCU03081	hypothetical protein				15.42											
NCU02904	alpha/beta hydrolase fold protein			15.41												
NCU03764	microtubule associated protein		15.79		15.41											
NCU02361	formamidase	18.65	18.67	15.41												
NCU02570	WD domain-containing protein	14.41			15.40											
NCU09741	NADPH-cytochrome P450 reductase				15.40											
NCU07402	pyridoxal reductase				15.36											
NCU07382	U6 snRNA-associated Sm-like protein LSm2				15.36											
NCU03092	nuclear localization sequence binding protein	18.20	15.93		15.35											
NCU02614	peptidyl-prolyl cis-trans isomerase H		15.37		15.35											
NCU02264	prefoldin subunit 3	15.83			15.35											
NCU04137	vacuolar protein sorting-associated protein Vps5				15.35											

NCU04516	hypothetical protein	17.03	16.50		15.34												
NCU06605	defective in methylation-8	15.75	16.18	15.32													
NCU09880	small nuclear ribonucleoprotein SmG		14.75		15.32												
NCU07509	geranylgeranyltransferase beta subunit		13.67		15.28												
NCU09702	endo-beta-1,6-galactanase			15.26													
NCU05254	ribose-phosphate pyrophosphokinase			15.26													
NCU01922	hypothetical protein	15.41		15.25													
NCU04743	tubulin-tyrosine ligase				15.24												
NCU00409	chorismate mutase/prephenate dehydratase	17.08	16.64		15.24												
NCU04845	phytanoyl-CoA dioxygenase				15.23												
NCU00920	isoleucyl-tRNA synthetase				15.21												
NCU05452	splicing factor 3b	13.73	14.34	15.21													
NCU02967	ankyrin repeat domain-containing protein		15.20		15.20												
NCU07967	hypothetical protein			15.20													
NCU05224	uridine-cytidine kinase 2	15.59			15.20												
NCU01069	amphiphysin-like-1				15.19												
NCU03086	HpcH/HpaI aldolase/citrate lyase family protein			15.18													
NCU02803	pre-mRNA-splicing ATP-dependent RNA helicase prp-28				15.17												
NCU01981	translation factor sui1	17.18	16.75	15.17													
NCU01181	acyl-CoA dehydrogenase-3				15.17												
NCU03803	sorbitol utilization protein SOU2		15.81		15.15												
NCU03159	3' exoribonuclease	14.15	14.17		15.14												
NCU00676	F1-ATP synthase assembly protein			15.13													
NCU00432	hypothetical protein			15.11													
NCU03760	hypothetical protein			15.11													
NCU16374	hypothetical protein				15.11												
NCU06258	hypothetical protein				15.11												
NCU00157	COP9 signalosome-1			15.10													
NCU06131	hypothetical protein			15.10													
NCU16337	nucleoporin-9				15.10												
NCU05304	nuclear segregation protein				15.10												
NCU03482	RuvB-like helicase 1				15.09												
NCU05971	xaa-Pro dipeptidase			15.08													
NCU02231	tRNA	14.11		15.08													
NCU01431	bni1-like	13.28	13.50	15.07													
NCU00166	isochorismatase domain-containing protein 2A			15.07													
NCU09621	guanine deaminase			15.06													
NCU01613	protoperithecia-2		15.18		15.05												
NCU03910	microsomal cytochrome b5				15.05												
NCU08018	fumarylacetoacetate hydrolase		13.56		15.02												
NCU09892	prolyl-tRNA synthetase			15.01													
NCU07404	glycosylhydrolase 38-1	15.89	15.83	15.01													
NCU08877	glycine cleavage system H protein		16.50	15.01													
NCU08359	cytidine deaminase				14.98												
NCU02978	cytoskeleton assembly control protein SLA1p	13.26	14.98	14.95													
NCU06616	S-adenosylmethionine-dependent methyltransferase	14.34			14.94												
NCU01643	5'-3' exoribonuclease 2			14.93													
NCU08030	UDP-glucose 4-epimerase Gal10	14.96	15.54		14.92												
NCU04301	peroxin 19	14.76	15.27		14.89												
NCU08178	purine nucleoside permease				14.88												
NCU11234	translation initiation protein Sua5	12.75	14.08		14.87												
NCU06558	D-amino-acid oxidase			14.87													
NCU01147	tyrosine decarboxylase	14.71			14.86												
NCU04612	mitogen activated protein kinase kinase 2			14.86													
NCU05299	NADH:ubiquinone oxidoreductase 29.9				14.86												
NCU01229	elp3 ortholog				14.85												
NCU00956	hypothetical protein			14.84													
NCU06880	AhpC/TSA family protein	14.44	14.11	14.82													
NCU03769	nucleosome assembly factor-2				14.81												
NCU04265	invertase				14.81												
NCU16757	H/ACA ribonucleoprotein complex subunit 1				14.80												
NCU01795	zinc finger containing protein			14.79													
NCU02754	casein kinase II subunit beta-2				14.79												
NCU06278	elongation factor 2				14.79												
NCU01913	pre-mRNA splicing factor				14.78												
NCU07982	acetolactate synthase			14.78													
NCU06644	hypothetical protein	15.03	13.68		14.75												
NCU09862	ubiquinone biosynthesis methyltransferase coq5			14.74													
NCU08604	hypothetical protein				14.72												
NCU02405	hypothetical protein		15.73		14.71												
NCU01374	CutC family protein				14.71												
NCU04636	cysteine desulfurase				14.69												
NCU00095	tetratricopeptide repeat protein 1	15.64	15.08	14.68													
NCU06869	cleavage and polyadenylation specificity factor			14.68													
NCU06347	actin cytoskeleton-regulatory complex protein end-3				14.68												
NCU00622	cell polarity protein				14.67												
NCU05390	mitochondrial phosphate carrier protein			14.66													
NCU10893	deoxyuridine 5'-triphosphate nucleotidohydrolase	15.12	16.51		14.66												
NCU06500	cell division control protein 25				14.65												
NCU04783	EH domain binding protein epsin 2			14.65													
NCU09473	3-ketoacyl-acyl carrier protein reductase	13.81	15.35	14.63													
NCU06672	autophagy ubiquitin-activating enzyme ApgG	14.31		14.63													
NCU03269	serine/threonine-protein phosphatase 2A activator 2				14.60												
NCU04566	protein kinase-10	13.98		14.59													
NCU02254	tubulin folding cofactor C				14.58												
NCU09326	hypothetical protein	14.41		14.55													
NCU00722	amidohydrolase			14.55													
NCU00814	hypothetical protein				14.54												
NCU06394	actin-related protein-5				14.53												
NCU04166	nucleolar GTP-binding protein 2	14.48	16.62	14.52													
NCU04796	3-ketoacyl-CoA thiolase			14.52													
NCU08674	pentatricopeptide repeat protein				14.51												
NCU04996	hypothetical protein	17.78	17.70	14.51													
NCU03028	deubiquitination-protection protein dph1				14.50												
NCU04466	cyanamide hydratase	16.09	15.34		14.49												
NCU00799	homocysteine S-methyltransferase	12.34	12.98		14.47												
NCU06871	doily			14.46													
NCU09930	folic acid synthesis protein				14.46												
NCU01845	phosphoglycerate mutase family domain-containing protein	17.15	16.86		14.45												

NCU02209	delta-12 fatty acid desaturase			14.45													
NCU05196	mitochondrial DnaJ chaperone				14.44												
NCU07318	mannitol-1-phosphate 5-dehydrogenase		14.29		14.43												
NCU02084	arginase				14.43												
NCU04469	hypothetical protein				14.41												
NCU03329	hypothetical protein			14.40													
NCU17030	heterogeneous nuclear ribonucleoprotein HRP1				14.39												
NCU05808	serine/threonine protein kinase			14.39													
NCU06543	acyl-CoA dehydrogenase			14.37													
NCU08956	MSP domain-containing protein				14.37												
NCU04908	hypothetical protein				14.36												
NCU07906	methyltransferase			14.36													
NCU08946	hypothetical protein			14.35													
NCU04074	NADH:ubiquinone oxidoreductase 30.4			14.33													
NCU08026	3' exoribonuclease	14.27	14.43		14.33												
NCU04595	protein kinase-11	14.15	14.61	14.33													
NCU03030	cytochrome-18			14.32													
NCU06344	disulfide isomerase	14.96	13.84		14.32												
NCU04172	hsp70-like protein		14.49		14.31												
NCU01500	nicotinamide riboside kinase 1			14.31													
NCU08031	paxneb protein superfamily				14.30												
NCU07379	bZIP-type transcription factor			14.29													
NCU04246	hypothetical protein				14.28												
NCU00895	RAB GTPase Ypt5				14.28												
NCU01772	DNA-directed RNA polymerase III polypeptide	14.09			14.27												
NCU06429	alpha-actinin			14.27													
NCU04261	hypothetical protein				14.25												
NCU07658	protein transporter sec72				14.25												
NCU09656	carboxymethylenebutenolidase				14.24												
NCU09553	3-hydroxybutyryl CoA dehydrogenase				14.24												
NCU08518	hypothetical protein				14.24												
NCU07060	hypothetical protein		13.69		14.23												
NCU03819	COPII coat assembly protein sec-16	13.02		14.23													
NCU05151	phosphoketolase			14.23													
NCU08421	hypothetical protein				14.22												
NCU09779	oxidoreductase				14.20												
NCU03280	NUDIX domain-containing protein				14.20												
NCU06706	killer toxin sensitivity protein				14.19												
NCU09894	CCAAT-box-binding transcription factor				14.19												
NCU03176	hypothetical protein				14.18												
NCU01800	37S ribosomal protein S5			14.18													
NCU03170	molybdopterin-converting factor subunit 2		13.55		14.16												
NCU09202	protein kinase dsk1			14.15													
NCU00434	protein phosphatase 2C isoform beta	14.71	14.19		14.14												
NCU16966	hypothetical protein			14.14													
NCU05757	nitrilase			14.13													
NCU08816	hypothetical protein	15.94	16.59		14.13												
NCU02736	hypothetical protein	16.21	15.11		14.11												
NCU06506	hypothetical protein	17.34	17.39	14.10													
NCU00695	hypothetical protein			14.10													
NCU08164	retinol dehydrogenase 13			14.09													
NCU04022	C2H2 finger domain-containing protein				14.08												
NCU04427	hypothetical protein				14.07												
NCU10518	general stress response protein Whi2			14.06													
NCU03440	AP-2 complex subunit alpha				14.05												
NCU09006	NEDD8-activating enzyme E1 regulatory subunit				14.04												
NCU01430	hypothetical protein			14.01													
NCU04333	ubiquitin C-terminal hydrolase	13.14			14.00												
NCU04407	RNA methylase			13.99													
NCU04309	phosphoribulokinase/uridine kinase				13.98												
NCU00595	ubiquitin thiolesterase			13.98													
NCU05392	leucine carboxyl methyltransferase 2	13.08	14.00		13.96												
NCU03908	fmp-52				13.96												
NCU06658	SET domain-containing protein				13.95												
NCU09594	seryl-tRNA synthetase				13.94												
NCU04800	E3 ubiquitin-protein ligase				13.94												
NCU09142	ropy-6			13.93													
NCU01218	hypothetical protein			13.92													
NCU00810	glycosylhydrolase family 2-3	14.00		13.91													
NCU03947	ubiquitin ligase			13.90													
NCU01636	2-hydroxychromene-2-carboxylate isomerase			13.88													
NCU00771	UBX domain-containing protein 7	14.77	14.44		13.87												
NCU03557	amino-acid N-acetyltransferase subunit Mak10			13.86													
NCU02056	salicylaldehyde dehydrogenase	15.68	15.30		13.84												
NCU07548	hypothetical protein				13.80												
NCU02296	allantoinase-1			13.77													
NCU08874	BET3 family protein				13.76												
NCU06615	hypothetical protein				13.74												
NCU05194	DNA replication licensing factor mcm6	11.73		13.74													
NCU06067	cdc24-like			13.73													
NCU04766	Ccr4-Not transcription complex subunit				13.73												
NCU03530	anchored cell wall protein-6	15.15		13.72													
NCU01822	retrograde transporter	14.91	15.34	13.70													
NCU00469	alpha-tubulin suppressor protein Aats1	15.63		13.70													
NCU04266	hypothetical protein	15.31	15.54	13.66													
NCU03435	hypothetical protein				13.66												
NCU06428	pseudouridine synthase TruD/Pus7	15.05			13.66												
NCU08167	aerobactin siderophore biosynthesis protein iucB	13.25			13.65												
NCU01857	DEAD/DEAH box RNA helicase				13.65												
NCU08036	pre-mRNA-splicing factor syf-1			13.65													
NCU03363	FRQ-interacting RNA helicase			13.65													
NCU08121	ribosome biogenesis protein	14.70			13.64												
NCU01449	riboflavin-3		14.09	13.62													
NCU09827	hypothetical protein	16.36	17.60		13.60												
NCU06540	topisomerase II associated protein	15.18	13.90	13.60													
NCU01397	hypothetical protein			13.60													
NCU01393	class III chitinase	14.79	14.89		13.57												
NCU00813	disulfide isomerase				13.55												
NCU02968	hypothetical protein				13.55												

NCU06315	inositol hexaphosphate kinase 3	15.04		13.54													
NCU11251	pre-mRNA splicing factor			13.52													
NCU07528	tRNA pseudouridine synthase	15.76	14.89	13.52													
NCU01817	DIL and Ankyrin domain-containing protein				13.46												
NCU08899	hypothetical protein				13.45												
NCU00243	microtubule-associated protein RP/EB family member 1	16.56	17.15		13.42												
NCU08120	mitochondrial ribosomal protein DAP3			13.41													
NCU01503	pre-rRNA processing protein Tsr1		13.36		13.40												
NCU00811	hypothetical protein	15.39			13.39												
NCU00417	cell cycle control protein cwf14	14.42		13.39													
NCU06539	hypothetical protein			13.39													
NCU04288	Pol			13.38													
NCU00068	hypothetical protein			13.37													
NCU03713	guanosine-diphosphatase		12.93		13.36												
NCU01019	hypothetical protein				13.32												
NCU09466	hypothetical protein			13.31													
NCU01276	N-acetyltransferase 5			13.30													
NCU08291	ferrochelatase			13.30													
NCU00287	hypothetical protein		12.89	13.29													
NCU01455	signal recognition particle protein		12.80	13.29													
NCU00900	hypothetical protein	14.55	14.46		13.27												
NCU08301	protein phosphatase 4-like		14.77	13.21													
NCU04797	fructose-bisphosphatase-1				13.20												
NCU02006	hypothetical protein	13.29			13.16												
NCU00065	tRNA-dihydrouridine synthase 1		14.62		13.11												
NCU11321	ribosomal L1 domain-containing protein 1				13.10												
NCU02203	C2H2 finger domain-containing protein				13.10												
NCU06659	GTP-binding protein	16.03	13.65		13.08												
NCU02620	polarity defective-4				13.03												
NCU02291	glutaryl-CoA dehydrogenase				13.02												
NCU00336	U3 small nucleolar RNA-associated protein 10				13.00												
NCU03776	ABC multidrug transporter				12.99												
NCU00798	hypothetical protein	18.39	19.54	12.91													
NCU07872	protein kinase-2			12.89													
NCU00263	serin endopeptidase			12.88													
NCU08664	tetratricopeptide repeat protein 1				12.87												
NCU09057	hypothetical protein		12.80	12.83													
NCU02651	hypothetical protein				12.82												
NCU00553	rho GTPase activator				12.80												
NCU00939	development and carotenogenesis control-1			12.79													
NCU07242	short-chain dehydrogenase/oxidoreductase			12.78													
NCU00995	hypothetical protein		15.73		12.76												
NCU03971	mRNA cap guanine-N7 methyltransferase	15.06			12.74												
NCU09658	hypothetical protein				12.67												
NCU01245	hypothetical protein				12.65												
NCU03305	calcium P-type ATPase-1			12.59													
NCU08468	bud6-like	14.02		12.50													
NCU03483	ropy-3				12.35												
NCU07405	hypothetical protein				12.32												
NCU03186	ankyrin repeat and BTB/POZ domain-containing protein				12.31												
NCU02734	citrate lyase beta subunit			12.21													
NCU02460	syntaxin-1			12.21													
NCU09499	hypothetical protein				12.17												
NCU03641	glycosylhydrolase family 3-1				11.92												
NCU04124	golgi membrane protein			11.76													
NCU03657	glycosylhydrolase family 63-1				11.67												
NCU07514	hypothetical protein																
NCU02564	cysteine-12																
NCU05404	endoglucanase																
NCU06242	lectin family integral membrane protein																
NCU00211	mitochondrial ribosomal protein subunit L31																
NCU09300	RNA Polymerase II CTD phosphatase Fcp1	14.69															
NCU05075	mitochondrial tricarboxylate transporter																
NCU00565	lipoic acid synthetase																
NCU01928	UBX domain-containing protein																
NCU06382	ABC transporter																
NCU01479	matrix mitochondrial AAA protease-1																
NCU08300	hypothetical protein																
NCU06748	hypothetical protein																
NCU16017	mito hypothetical protein																
NCU06832	kinesin																
NCU07173	ADP-ribosylation factor 6																
NCU06776	hypothetical protein																
NCU00614	DUF1620 domain-containing protein																
NCU09813	SMT3		15.13														
NCU09748	transcription initiation factor IIA gamma chain	14.60															
NCU05816	hypothetical protein																
NCU09647	hypothetical protein																
NCU01170	hypothetical protein																
NCU09538	microsomal dipeptidase																
NCU04814	DUF21 and CBS domain-containing protein																
NCU02618	peroxin 13																
NCU00600	rho-type GTPase-3																
NCU03946	polyadenylate-binding protein 2		15.08														
NCU01474	60S ribosomal protein L4																
NCU02232	DNA primase small subunit	14.31															
NCU00476	UPF0023 family protein		14.45														
NCU06333	translocation protein SEC62																
NCU04605	hypothetical protein																
NCU03155	hypothetical protein																
NCU05136	endo alpha-1,4 polygalactosaminidase precursor																
NCU03637	CaaX prenyl protease Ste24																
NCU00232	hypothetical protein		16.31														
NCU04832	2-dehydropantoate 2-reductase																
NCU00502	ATP synthase subunit 4																
NCU00242	V-SNARE																
NCU09579	retinol dehydrogenase 12																
NCU08676	hypothetical protein																
NCU03310	prohibitin-2																

NCU06008	hypothetical protein																
NCU02160	Rac1 homolog-1	15.34															
NCU00484	NADH:ubiquinone oxidoreductase 18.4																
NCU03367	hypothetical protein	13.40															
NCU01850	peroxin 16																
NCU00904	D-lactate dehydrogenase																
NCU09540	hypothetical protein		14.23														
NCU16004	mito NADH dehydrogenase subunit 6																
NCU01689	mitochondrial DNA replication protein YHM2																
NCU02423	mitoferrin-1																
NCU06103	mitochondrial translation initiation factor																
NCU04405	mago nashi protein	14.82															
NCU06275	hypothetical protein																
NCU02280	NADH:ubiquinone oxidoreductase 21.3b																
NCU08379	hypothetical protein																
NCU02269	mitochondrial carnitine/acylcarnitine carrier protein																
NCU01183	GTP-binding protein 1	15.00															
NCU03966	mitochondrial Rho GTPase 1																
NCU03735	hypothetical protein																
NCU00536	homoserine O-acetyltransferase																
NCU06454	cell division cycle 42-like																
NCU00737	presequence translocated-associated motor subunit pam-17																
NCU00564	mitochondrial 40S ribosomal protein MRP2																
NCU06958	mito ribosomal protein S21																
NCU07712	ATP-dependent RNA helicase dbp-10		14.12														
NCU09143	hypothetical protein																
NCU05231	ER membrane DUF1077 domain-containing protein																
NCU04068	60S ribosomal protein L6																
NCU00411	hypothetical protein																
NCU05286	hypothetical protein																
NCU06643	oleate-induced peroxisomal protein																
NCU01792	heat shock protein 90a		15.79														
NCU03230	mitochondrial ribosomal protein subunit S18																
NCU09259	hypothetical protein	13.69															
NCU05959	vesicle transport V-SNARE protein VTI1																
NCU06150	hypothetical protein																
NCU07434	short-chain dehydrogenase/reductase SDR																
NCU06451	hypothetical protein																
NCU07546	multidrug resistance protein MDR																
NCU17277	hypothetical protein																
NCU05899	flotillin domain-containing protein																
NCU07955	polarity defective-1																
NCU06741	cytochrome c oxidase subunit VIb																
NCU01501	mitochondrial peptidyl-tRNA hydrolase Pth2																
NCU00466	glutamyl-tRNA synthetase																
NCU09517	hypothetical protein																
NCU01983	hypothetical protein																
NCU03901	peroxin 14																
NCU09216	oligosaccharyl transferase epsilon subunit																
NCU01680	plasma membrane ATPase-1																
NCU02079	cyclin-dependent kinase regulatory subunit		15.59														
NCU08743	inorganic phosphate transporter																
NCU02310	hypothetical protein																
NCU02544	ABC transporter																
NCU01601	hypothetical protein		15.78														
NCU01298	hypothetical protein																
NCU06469	54S ribosomal protein L12																
NCU03957	COQ7																
NCU06760	serine/threonine protein kinase-48																
NCU00923	topogenesis of outer membrane beta barrel protein 37																
NCU06076	hypothetical protein																
NCU00169	translocation complex component																
NCU04336	60S ribosomal protein L19																
NCU04781	NADH:ubiquinone oxidoreductase 9.8																
NCU03231	mitochondrial chaperone BCS1																
NCU01006	complex I intermediate associated protein 84																
NCU03624	hypothetical protein																
NCU02373	NADH:ubiquinone oxidoreductase 40																
NCU07503	hypothetical protein																
NCU02623	mitochondrial hypoxia responsive domain-containing protein																
NCU05551	hypothetical protein																
NCU01668	hypothetical protein																
NCU07129	amino-acid permease inda1																
NCU03276	bud emergence 46-like																
NCU02644	hypothetical protein		13.79														
NCU02507	hypothetical protein																
NCU06327	benzoate 4-monooxygenase cytochrome P450																
NCU03080	hypothetical protein																
NCU04013	yellow-1																
NCU06515	hypothetical protein																
NCU02937	hypothetical protein																
NCU04171	50S ribosomal protein L5																
NCU17008	hypothetical protein	12.61															
NCU02541	oligosaccharyltransferase alpha subunit																
NCU00670	NADH:ubiquinone oxidoreductase 9.5																
NCU04837	mitochondrial 2-oxodicarboxylate carrier 1																
NCU11345	hypothetical protein																
NCU01514	mitochondrial dicarboxylate carrier																
NCU03783	transmembrane domain transporter																
NCU04180	mitochondrial folate carrier protein Flx1																
NCU01813	high affinity glucose transporter																
NCU03509	permease general amino acid																
NCU03838	transcription elongation factor spt-4		14.72														
NCU01763	hypothetical protein																
NCU01142	NADH:ubiquinone oxidoreductase 13.4																
NCU01920	hypothetical protein																
NCU01169	NADH:ubiquinone oxidoreductase 24																
NCU03616	semicolonial-7																
NCU08924	acyl-CoA dehydrogenase-2																

NCU08166	hypothetical protein																
NCU01575	SNARE complex subunit																
NCU08484	mutagen sensitive-27	16.14															
NCU05313	mitochondria fission 1 protein																
NCU01241	mitochondrial carrier protein																
NCU01467	NADH:ubiquinone oxidoreductase 10.4																
NCU07842	Woronin sorting complex																
NCU01101	mitochondrial import 1																
NCU06881	succinyl-CoA:3-ketoacid-coenzyme A transferase subunit A																
NCU01916	mitochondrial carrier protein RIM2																
NCU16833	hypothetical protein																
NCU00584	hypothetical protein																
NCU04784	hypothetical protein																
NCU03340	cytochrome-c oxidase chain VIIc																
NCU06587	hypothetical protein																
NCU03394	ribosomal protein S15																
NCU08947	ubiquinol-cytochrome-C reductase 8																
NCU03216	adenylate kinase																
NCU02706	protein transporter GOS1																
NCU06541	alpha-1,2 mannosyltransferase KTR1																
NCU00121	CLC channel protein																
NCU09866	thyroid hormone receptor interactor 12	14.91															
NCU00456	hypothetical protein																
NCU04653	LCCL domain-containing protein																
NCU08370	sporulation protein RMD8																
NCU02804	mitochondrial 60S ribosomal protein L25																
NCU05235	ribosome biogenesis protein RLP24		16.54														
NCU01282	vacuolar-sorting protein snf-7																
NCU05717	37S ribosomal protein Mrp17																
NCU00523	NAD-dependent deacetylase sirtuin-2	15.36															
NCU09598	mitochondrial escape protein 2																
NCU01644	intracellular protein transporter UsoA	13.49															
NCU09539	40S ribosomal protein S13																
NCU02419	mitochondrial 37S ribosomal protein S17																
NCU06200	DUF1682 domain-containing protein																
NCU08410	50S ribosomal protein L24																
NCU07330	endosomal integral membrane protein																
NCU00952	mitochondrial ribosomal protein L44																
NCU03800	endosomal cargo receptor																
NCU02013	hypothetical protein																
NCU04304	porin	14.36															
NCU05046	E1-E2 ATPase-1																
NCU00418	NADH:ubiquinone oxidoreductase 14.8																
NCU03711	GTP-binding protein ypt7																
NCU03627	hypothetical protein																
NCU05985	glycerol-3-phosphate O-acyltransferase																
NCU01751	ATP-binding cassette protein																
NCU07736	PEP5																
NCU01395	WSC domain-containing protein	15.89															
NCU04471	hypothetical protein																
NCU09091	mitochondrial inner membrane magnesium transporter mrs2																
NCU06740	hypothetical protein																
NCU04276	hypothetical protein																
NCU03801	alkaline phosphatase																
NCU01360	NADH:ubiquinone oxidoreductase 11.5																
NCU07549	mitochondrial ribosomal protein L43																
NCU09403	NmrA family protein																
NCU06244	translocase of inner mitochondrial membrane, subunit 13																
NCU09462	hypothetical protein																
NCU03554	hypothetical protein																
NCU16018	mito NADH dehydrogenase subunit 1																
NCU01516	mitochondrial co-chaperone GrpE																
NCU05360	calcium permease																
NCU00528	hyphal anastomosis-4																
NCU00712	3-hydroxy-3-methylglutaryl-coenzyme A reductase																
NCU01535	PEX33																
NCU07746	F-box domain-containing protein																
NCU10351	pyridoxine kinase	12.56															
NCU08930	NADH:ubiquinone oxidoreductase 21.3a																
NCU02016	hypothetical protein																
NCU08009	DNA replication licensing factor mcm3	12.32															
NCU00552	albino-1																
NCU05635	hypothetical protein																
NCU03156	NADH:ubiquinone oxidoreductase 10.5																
NCU00030	mitochondrial nuclease																
NCU05225	NADH dehydrogenase 64																
NCU01761	hypothetical protein																
NCU03257	ammonium transporter MEP1																
NCU02565	hypothetical protein																
NCU07884	mitochondrial oxaloacetate transporter																
NCU09012	hypothetical protein																
NCU01485	hypothetical protein																
NCU09498	hypothetical protein		15.85														
NCU01041	mitochondrial metalloendopeptidase OMA1																
NCU07003	hypothetical protein		12.69														
NCU03997	hypothetical protein																
NCU05220	ATP synthase subunit F																
NCU07806	hypothetical protein																
NCU04090	niemann-Pick C1 protein																
NCU06536	hypothetical protein																
NCU00051	hypothetical protein																
NCU06850	1,3-beta-glucanosyltransferase gel3	14.69															
NCU08552	50S ribosomal protein L14																
NCU03007	pre-mRNA-splicing factor slt-11	14.59															
NCU06986	DUF221 domain-containing protein																
NCU09610	sey-1																
NCU07793	hypothetical protein																
NCU08356	acetamidase																
NCU04156	ergosterol-1																

NCU04480	hypothetical protein																
NCU16968	cytochrome c mitochondrial import factor																
NCU03020	IdgA domain-containing protein																
NCU03158	alpha/beta hydrolase																
NCU07493	hypothetical protein	14.29															
NCU02403	ubiquinone biosynthesis protein COQ9																
NCU09249	prefoldin subunit 2		13.68														
NCU03906	BSD domain-containing protein	14.78															
NCU00041	guanine nucleotide-binding protein subunit gamma																
NCU07756	tricarboxylic acid-11																
NCU08934	hypothetical protein																
NCU00894	hypothetical protein																
NCU09299	NADH:ubiquinone oxidoreductase 14																
NCU05298	nitrate nonutilizer-3	15.20															
NCU00436	ultraviolet-sensitive-5																
NCU05219	hypothetical protein	13.44															
NCU00605	ThiF domain-containing protein																
NCU00227	mitochondrial cation transporter																
NCU03083	hypothetical protein																
NCU01740	histone H2A.Z-specific chaperone chz-1		17.62														
NCU03188	sugar 1,4-lactone oxidase																
NCU01115	tRNA-splicing endonuclease subunit SEN2																
NCU11258	LYR family protein																
NCU06606	iron-sulfur subunit-1																
NCU06488	chromatin remodeling factor 3-1		13.38														
NCU09161	translation factor pelota	13.16															
NCU03639	lipase																
NCU02951	40S ribosomal protein S8																
NCU01648	dolichyl-phosphate-mannose-protein mannosyltransferase 2																
NCU04127	hypothetical protein																
NCU05515	unknown-4																
NCU08980	NA																
NCU03057	hypothetical protein		14.31														
NCU02545	hypothetical protein																
NCU04117	ATP-dependent permease MDL2																
NCU08549	UDP-galactose 4-epimerase	16.43															
NCU08342	COP9 signalosome-7		12.29														
NCU05521	pathogenicity protein																
NCU04823	NADP-dependent alcohol dehydrogenase C	16.68															
NCU04474	sulfite oxidase		17.03														
NCU01804	nudix/MutT family protein		13.81														
NCU06113	ammecr1 family protein	12.98															
NCU07948	hypothetical protein																
NCU05828	hypothetical protein																
NCU03263	transmembrane protein																
NCU07913	hypothetical protein	13.42															
NCU08226	hypothetical protein																
NCU06265	hypothetical protein																
NCU05577	glycosylhydrolase family 3-5																
NCU05454	glycerol-3-phosphate dehydrogenase																
NCU10732	mitochondrial dicarboxylate transporter																
NCU01319	hypothetical protein																
NCU00596	lipoyltransferase																
NCU02474	translocase of outer mitochondrial membrane 5																
NCU00440	guanine nucleotide binding protein beta-1																
NCU03546	hypothetical protein		13.55														
NCU06777	syntaxin																
NCU07112	hypothetical protein																
NCU04526	hypothetical protein																
NCU08794	UPF0041 domain-containing protein																
NCU04232	hypothetical protein																
NCU05027	hypothetical protein																
NCU03217	MRS7 family protein																
NCU11282	acyltransferase																
NCU03335	ER associated DnaJ chaperone																
NCU07386	Fe superoxide dismutase																
NCU07852	ribosomal protein L13																
NCU09878	hypothetical protein																
NCU04921	DUF1295 domain-containing protein																
NCU06518	NADH-cytochrome b5 reductase 2																
NCU00589	auxin Efflux Carrier superfamily																
NCU16012	mito NADH dehydrogenase subunit 5																
NCU02669	signal sequence binding protein																
NCU04502	hypothetical protein																
NCU01201	trm-112		14.86														
NCU06493	guanine nucleotide-binding protein alpha-1																
NCU04669	hypothetical protein																
NCU03784	cation chloride cotransporter																
NCU08579	ubiquitin C-terminal hydrolase																
NCU01657	cytochrome-c oxidase assembly protein																
NCU03093	NADH:ubiquinone oxidoreductase 12.3																
NCU01650	hypothetical protein																
NCU04138	hypothetical protein																
NCU03199	ATP synthase subunit H																
NCU05591	ABC transporter CDR4																
NCU02250	oligomycin-resistant																
NCU09874	hypothetical protein																
NCU16007	mito NADH dehydrogenase subunit 3																
NCU01141	CDP-diacylglycerol-glycerol-3-phosphate 3-phosphatidyltransferase																
NCU05202	hypothetical protein																
NCU04212	hypothetical protein																
NCU06610	PQ loop repeat protein																
NCU01964	RING-9 protein																
NCU00959	tricarboxylic acid-10																
NCU03798	translocase of inner mitochondrial membrane 22																
NCU09460	NADH:ubiquinone oxidoreductase 20.1																
NCU04005	casein kinase-1b																
NCU09133	anchored cell wall protein-7		16.07														
NCU07864	60S ribosome biogenesis protein Mak11		14.47														

NCU01401	topogenesis of outer membrane beta barrel proteins 38																
NCU08350	hypothetical protein																
NCU02807	hypothetical protein																
NCU00153	NA																
NCU05221	NADH:ubiquinone oxidoreductase 21																
NCU01304	mitochondrial mRNA processing protein PET127																
NCU09877	hypothetical protein																
NCU06095	grainy-head homolog	13.46															
NCU01146	signal sequence receptor alpha chain																
NCU02064	hypothetical protein																
NCU05813	mitochondrial large ribosomal subunit																
NCU07042	alcohol dehydrogenase	14.11															
NCU03008	hypothetical protein																
NCU09343	hypothetical protein																
NCU05287	50S ribosomal protein L4																
NCU06790	hypothetical protein																
NCU03233	hypothetical protein																
NCU07002	THO complex component	12.52															
NCU01997	ABC transporter																
NCU04603	hypothetical protein	16.28															
NCU02271	YagE family protein																
NCU08071	cytochrome-21																
NCU06402	C-4 methylsterol oxidase																
NCU16006	mito NADH dehydrogenase subunit 2																
NCU05838	hypothetical protein																
NCU03823	serine/threonine protein kinase-25																
NCU05752	indoleamine 2,3-dioxygenase		15.08														
NCU08278	mitochondrial carrier protein																
NCU06846	MFS myo-inositol transporter																
NCU07657	hypothetical protein	13.16															
NCU00458	hypothetical protein																
NCU06778	oxidase assembly protein 2																
NCU02177	hypothetical protein																
NCU00833	hypothetical protein																
NCU09017	hypothetical protein		12.82														
NCU06632	translocase of outer mitochondrial membrane 20																
NCU06803	glutamate decarboxylase		13.88														
NCU01353	glycosylhydrolase family 16-1		14.83														
NCU06542	mitochondrial 30S ribosomal protein S12																
NCU00527	mitochondrial genome maintenance protein Mgr2																
NCU04019	nicotinic acid-5	13.31															
NCU00114	30S ribosomal protein S10																
NCU07362	L-lactate ferricytochrome c oxidoreductase																
NCU03818	phospholipid-translocating P-type ATPase																
NCU06120	hypothetical protein																
NCU04817	electron transfer protein 1																
NCU03177	sco1																
NCU07701	DUF300 domain-containing protein																
NCU04753	NADH:ubiquinone oxidoreductase 11.6																
NCU02753	WD repeat-containing protein		14.31														
NCU07465	mitochondrial phosphate carrier protein 2																
NCU01633	hexose transporter HXT13																
NCU08607	endoplasmic reticulum-Golgi intermediate compartment protein 3																
NCU04656	MFS transporter																
NCU08093	hypothetical protein																
NCU04244	syntaxin-2																
NCU01654	long-chain-fatty-acid-CoA ligase 1																
NCU00204	hypothetical protein																
NCU08267	activating signal cointegrator 1 complex subunit 3		11.93														
NCU07960	MFS transporter																
NCU04287	hypothetical protein																
NCU02633	nuclear mRNA splicing factor-associated protein		13.39														
NCU01745	SPX domain-containing protein																
NCU01767	phophatase 2C family protein																
NCU07075	calcium exchanger																
NCU07904	short chain dehydrogenase/reductase																
NCU00636	ATP synthase subunit D																
NCU07959	hypothetical protein																
NCU08146	hypothetical protein																
NCU06111	GTPase Ras2p																
NCU05009	NADH:ubiquinone oxidoreductase 21.3																
NCU06495	mitochondrial inner membrane organization protein mio10																
NCU00447	serine palmitoyltransferase 2																
NCU12023	37S ribosomal protein MRP4																
NCU01553	para-hydroxybenzoate-polyprenyltransferase Coq2																
NCU01606	ATP synthase subunit 5																
NCU02690	mitochondrial integral membrane protein																
NCU02115	hypothetical protein																
NCU04305	hypothetical protein																
NCU16844	hypothetical protein																
NCU01950	pre-mRNA-splicing factor slu-7		14.31														
NCU08995	dual specificity phosphatase																
NCU04270	hypothetical protein		14.61														
NCU03549	hypothetical protein																
NCU06231	vacuolar amino acid transporter 1																
NCU07375	MFS phosphate transporter																
NCU05338	hypothetical protein																
NCU09642	high affinity sulfate transporter 1																
NCU01828	hypothetical protein																
NCU04764	hypothetical protein																
NCU00216	NADH-cytochrome b5 reductase 1																
NCU09663	Axe2																
NCU01404	RNA methyltransferase																
NCU01324	hypothetical protein																
NCU02305	decaprenyl-diphosphate synthase subunit 1																
NCU05076	hypothetical protein																
NCU05226	ABC transporter																
NCU04061	hypothetical protein		13.66														
NCU08897	protein transporter SEC61 subunit alpha																

NCU00109	TipA protein		14.88														
NCU02802	arginine-13																
NCU02267	mitochondrial protein Fmp25																
NCU05272	TBC domain-containing protein																
NCU02336	capsule associated protein																
NCU04768	electron transfer flavoprotein-ubiquinone oxidoreductase																
NCU00585	albino-2																
NCU02095	hypothetical protein																
NCU04181	translocase of outer mitochondrial membrane 7																
NCU03989	ADP,ATP carrier protein																
NCU08940	ubiquinol-cytochrome-C reductase 7																
NCU08919	chromatin remodelling factor 2-1	15.39															
NCU04898	cation-transporting ATPase 4																
NCU00269	set-domain histone methyltransferase-2		11.84														
NCU03408	hypothetical protein																
NCU07988	ATPase family AAA domain-containing protein 1																
NCU02894	flavin-binding monooxygenase																
NCU02157	COQ4																
NCU06694	fatty acid elongase																
NCU00958	5-azacytidine resistance protein azr1																
NCU08272	cytochrome b2	14.56															
NCU10055	new eukaryotic opsin-1																
NCU03338	hypothetical protein																
NCU05578	hypothetical protein																
NCU09332	mannosyltransferase PMTI																
NCU01140	NA																
NCU00168	1-acylglycerol-3-phosphate O-acyltransferase																
NCU16454	bacilysin biosynthesis oxidoreductase bacC																
NCU07558	sporulation protein RMD1																
NCU04114	hypothetical protein																
NCU04549	hypothetical protein																
NCU03695	phosphatidylserine decarboxylase proenzyme 1																
NCU05715	sorting nexin mvp-1																
NCU00541	hypothetical protein	13.66															
NCU08683	rho-type GTPase-2																
NCU09999	hypothetical protein																
NCU05835	hypothetical protein	13.94															
NCU06921	mRNA splicing factor		13.62														
NCU00754	multidrug resistant protein																
NCU02184	chitinase-1																
NCU01975	complex I intermediate associated protein 30																
NCU08618	ADP-ribosylation factor family protein																
NCU07295	translocase of inner mitochondrial membrane 54																
NCU07447	DUF803 domain-containing protein																
NCU00669	oligosaccharyl transferase subunit																
NCU02070	peroxin 2																
NCU02811	hyphal anastamosis-8																
NCU07858	DUF498 domain-containing protein																
NCU06546	hypothetical protein																
NCU04493	hypothetical protein		14.98														
NCU03145	oxidative stress resistance																
NCU04355	phosphatidylinositol 4-kinase type II subunit alpha																
NCU02287	acyl-CoA dehydrogenase-1																
NCU01342	endoplasmic reticulum vesicle protein 25																
NCU11248	oligosaccharyltransferase subunit ribophorin II																
NCU08561	succinate/fumarate mitochondrial transporter																
NCU05079	MFS peptide transporter																
NCU06929	transcription elongation factor spt-5		13.17														
NCU09859	metalloprotease 1																
NCU07879	mitochondrial metal transporter 2																
NCU07478	mitochondrial carrier protein																
NCU07672	hypothetical protein																
NCU08686	hypothetical protein																
NCU03948	amidohydrolase 3																
NCU00025	integral membrane protein																
NCU09886	hypothetical protein																
NCU08941	calcium-binding mitochondrial carrier protein Aralar2																
NCU00316	peroxisomal adenine nucleotide transporter 1																
NCU07166	hypothetical protein	13.46															
NCU03873	hypothetical protein																
NCU06145	RING-6	14.15															
NCU03929	acyl-CoA synthetase																
NCU07720	MATE efflux family protein																
NCU06992	DNA repair protein Nse1	13.72															
NCU02074	endoplasmic oxidoreductin-1																
NCU01907	syntaxin 5																
NCU07324	conidiation-13																
NCU08510	hypothetical protein																
NCU00766	hypothetical protein	13.73															
NCU09505	hypothetical protein	14.10															
NCU05279	hypothetical protein																
NCU03561	mitochondrial carrier protein																
NCU09257	hypothetical protein																
NCU02107	AMP-binding enzyme		13.03														
NCU00226	MFS transporter																
NCU02162	SURF-family protein																
NCU07560	50S ribosomal subunit L30																
NCU09895	thioesterase																
NCU02063	mitochondrial intermediate peptidase																
NCU01647	GTP-binding protein ypt4																
NCU06107	hypothetical protein		13.13														
NCU08541	hypothetical protein																
NCU04133	peptidase family M28																
NCU00360	NAD dependent epimerase/dehydratase																
NCU04801	chalcone synthase	15.76															
NCU00369	dephospho-CoA kinase																
NCU06786	50S ribosomal protein L30																
NCU08861	hypothetical protein																
NCU05601	cytochrome-2																

NCU07717	replication protein A-2	14.56															
NCU00644	ATP synthase subunit G																
NCU08110	vacuolar transporter chaperone 4																
NCU02577	hypothetical protein																
NCU06924	kynurenine 3-monooxygenase																
NCU08326	mitochondrial carrier protein LEU5																
NCU01962	encodes anonymous transcript-5																
NCU08358	hypothetical protein																
NCU05982	hexaprenyldihydroxybenzoate methyltransferase																
NCU04946	hypothetical protein																
NCU00135	phosphatidyl synthase																
NCU02681	translocation protein																
NCU07015	queuine tRNA-ribosyltransferase		13.20														
NCU01568	hypothetical protein																
NCU10143	ATPase type 13A2																
NCU09428	hypothetical protein																
NCU00643	L-arabinitol 4-dehydrogenase	13.05															
NCU06532	dihydroorotate reductase PyrE																
NCU10225	hypothetical protein																
NCU07286	membrane-associated progesterone receptor component 1																
NCU07200	metalloprotease 1																
NCU08893	60S ribosomal protein L1																
NCU05633	stomatin family protein																
NCU02632	sulfate transporter																
NCU06054	squalene synthetase																
NCU00278	hypothetical protein																
NCU04736	calcium P-type ATPase-2																
NCU07442	betaine aldehyde dehydrogenase																
NCU03953	NADH:ubiquinone oxidoreductase 19.3																
NCU05775	amino acid transporter																
NCU02936	proline oxidase																
NCU01564	calcium dependent mitochondrial carrier protein																
NCU02624	cytochrome P450 51	15.03															
NCU06977	hypothetical protein																
NCU02704	branched-chain alpha-keto acid dehydrogenase E2 component																
NCU05589	sip-5		14.29														
NCU01540	SPX domain-containing protein																
NCU03827	40S ribosomal protein S9																
NCU04092	N-acylethanolamine amidohydrolase																
NCU16005	mito mitochondrial ribosomal protein S5																
NCU04806	hypothetical protein																
NCU04259	serine/threonine protein kinase-27																
NCU07974	endoglucanase B																
NCU04206	hypothetical protein																
NCU02943	translocase of inner mitochondrial membrane 50																
NCU02451	mitochondrial hypoxia responsive domain-containing protein																
NCU00057	ubiquinol cytochrome-c reductase assembly protein Cbp3																
NCU00078	hypothetical protein																
NCU08994	hypothetical protein																
NCU10987	MFS transporter Fmp42																
NCU02490	DnaJ domain-containing protein																
NCU03343	37S ribosomal protein S25																
NCU01145	hypothetical protein		12.07														
NCU06485	hypothetical protein	15.13															
NCU07403	secretory component protein shr3																
NCU03147	assembly factor cbp-4																
NCU07937	sphingoid long chain base kinase 4																
NCU02472	NADH:ubiquinone oxidoreductase 20.8																
NCU08949	hypothetical protein																
NCU11348	NADH-ubiquinone oxidoreductase B18 subunit																
NCU10747	hypothetical protein																
NCU02504	DUF1640 domain-containing protein																
NCU07263	carnitine/acyl carnitine carrier																
NCU11287	hypothetical protein		15.09														
NCU01859	NADH:ubiquinone oxidoreductase 20.9																
NCU01989	hypothetical protein																
NCU03168	aspartic-type endopeptidase																
NCU08138	cytochrome C1 heme lyase																
NCU07363	hypothetical protein																
NCU06246	ATP-dependent RNA helicase mrh-4																
NCU07855	hypothetical protein	14.18															
NCU06596	hypothetical protein																
NCU07232	hsp30-like protein	14.93															
NCU08992	hypothetical protein																
NCU00006	hypothetical protein		14.02														
NCU06468	midasin		13.85														
NCU09806	hypothetical protein		14.42														
NCU00684	endonuclease/Exonuclease/phosphatase																
NCU01675	ribosomal RNA-processing protein 12		13.17														
NCU00371	translocase of inner mitochondrial membrane 23																
NCU01912	mannosyltransferase 1																
NCU02041	anchored cell wall protein-11																
NCU03135	hypothetical protein																
NCU06749	60S ribosomal protein L3																
NCU01454	mitochondrial hydrolase																
NCU03556	peroxisomal membrane protein Pmp47																
NCU16016	mito cytochrome c oxidase subunit 1																
NCU09002	NADH:ubiquinone oxidoreductase 10.6																
NCU06403	hypothetical protein																
NCU08354	hypothetical protein																
NCU00424	Spo76 protein		14.47														
NCU00789	DUF221 domain-containing protein																
NCU02705	F1F0 ATP synthase assembly protein Atp10																
NCU04350	chitin synthase 6																
NCU02100	LMBR1 domain-containing protein 1																
NCU04189	colonial temperature sensitive-2	14.53															
NCU03752	EBP domain-containing protein																
NCU01023	50S ribosomal protein L17																
NCU06601	hypothetical protein																

NCU00562	hypothetical protein																
NCU03591	ATP-dependent bile acid permease																
NCU08976	fatty acid elongase																
NCU01007	mito ribosomal protein S18																
NCU00925	pescadillo		14.68														
NCU06761	sphingosine-1-phosphate lyase																
NCU01894	hypothetical protein																
NCU03359	intermembrane space AAA protease-1																
NCU00905	N-acylethanolamine amidohydrolase																
NCU01307	hypothetical protein																
NCU06317	stress response RCI peptide																
NCU00431	translocase of mitochondrial outer membrane 22																
NCU02149	cytochrome-1																
NCU06588	hypothetical protein																
NCU06619	methyltryptophan resistant																
NCU10311	mitochondrial ribosomal protein subunit S4																
NCU05689	cytochrome oxidase-4																
NCU05844	hypothetical protein																
NCU09207	hypothetical protein																
NCU05600	serine/threonine protein kinase-33																
NCU09476	40S ribosomal protein S25		19.89														
NCU06189	5-aminolevulinate synthase																
NCU03088	hypothetical protein																
NCU08565	hypothetical protein																
NCU03926	37S ribosomal protein Rsm24																
NCU01255	mitochondrial dynamin GTPase																
NCU06768	60S ribosomal protein L16																
NCU00957	anchored cell wall protein-2																
NCU06450	mitochondrial ribosomal protein																
NCU04461	transmembrane domain-containing protein																
NCU06370	vesicle-associated membrane protein 712																
NCU01436	questionable protein																
NCU00969	NADH:ubiquinone oxidoreductase 17.8																
NCU02152	RRM domain-containing protein	14.73															
NCU05029	iron-sulfur clusters transporter atm-1																
NCU08339	endosomal P24B protein																
NCU04169	hypothetical protein																
NCU00167	hypothetical protein																
NCU05160	ATP-dependent Zn protease	13.65															
NCU09755	hypothetical protein																
NCU03463	vacuolar pH-sensitive ATPase-1																
NCU11118	hypothetical protein																
NCU01807	hypothetical protein																
NCU02603	oxidoreductase		13.49														
NCU05593	topogenesis of mitochondrial outer membrane beta barrel proteins 55																
NCU07072	galactose-1-phosphate uridylyltransferase																
NCU01102	oxidase assembly protein 1																
NCU03802	carnitine biosynthesis-1																
NCU04622	hypothetical protein																
NCU01473	mitochondrial large ribosomal subunit YmL35																
NCU09972	hypothetical protein																
NCU08217	SRP receptor beta subunit																
NCU01470	vacuolar protein 8																
NCU01655	hypothetical protein																
NCU03211	hypothetical protein																
NCU09192	CDP-diacylglycerol-inositol 3-phosphatidyltransferase PIS																
NCU08382	peroxisomal half ABC transporter																
NCU03312	CorA family metal ion transporter																
NCU03875	chromatin remodelling factor 4-1		14.27														
NCU01024	hypothetical protein																
NCU09599	hypothetical protein																
NCU03395	vacuolar membrane ATPase-6																
NCU05714	50S ribosomal subunit protein L15																
NCU05457	cytochrome a-4																
NCU09816	cytochrome-26																
NCU04503	ribosome biogenesis protein Ssf2		14.96														
NCU05259	acyl-CoA desaturase 1																
NCU00101	pbn-1																
NCU09931	oxidoreductase																
NCU01888	tRNA-specific adenosine deaminase																
NCU04644	hypothetical protein																

Table S5. Categories of proteins enriched the pool of down-regulated proteins in ∆Por-1.

A. Proteins in Functional Category 1.01 Amino acid metabolism

NCU09896	adenylyl-sulfate kinase
NCU08998	4-aminobutyrate aminotransferase
NCU07930	cysteine-18
NCU09864	Mt: 2-oxoisovalerate dehydrogenase alpha subunit

B. Proteins in Functional Category 01.01.09.03.01 Biosynthesis of cysteine

NCU01652	O-acetylhomoserine
NCU08216	cystathionine beta-synthase
NCU01985	cysteine-11

C. Proteins in Functional Category 1.02 Nitrogen, sulfur and selenium metabolism

NCU01652	O-acetylhomoserine
NCU09896	adenylyl-sulfate kinase
NCU03973	alanine
NCU01195	amination-deficient
NCU08998	4-aminobutyrate aminotransferase
NCU05238	cysteine-4
NCU01985	cysteine-11
NCU01428	hydroxyisourate hydrolase
NCU04720	nitrate nonutilizer-6
NCU09291	Mt: FAD dependent sulfhydryl oxidase Erv2

D. Proteins in Functional Category 01.02.03.01 Sulfate assimilation

NCU09896	adenylyl-sulfate kinase
NCU05238	cysteine-4
NCU01985	cysteine-11

E. Proteins in Functional Category 01.02.03.04 Conjunction of sulfate

NCU09896	adenylyl-sulfate kinase
NCU01985	cysteine-11

F. Proteins in Functional Category 01.06.06.11 Tetracyclic and pentacyclic triterpenes (cholesterin, steroids and hopanoids) metabolism

NCU02571	acetyl-CoA acetyltransferase
NCU11381	diphosphomevalonate decarboxylase
NCU03922	hydroxymethylglutaryl-CoA synthase
NCU07719	isopentenyl-diphosphate delta-isomerase
NCU03897	RNA binding effector protein Scp160
NCU00712	Mt: 3-hydroxy-3-methylglutaryl-coenzyme A reductase
NCU02669	Mt: signal sequence binding protein

G. Proteins in Functional Category 16.21.17 pyridoxal phosphate binding

NCU01652	O-acetylhomoserine
NCU03973	alanine
NCU08998	4-aminobutyrate aminotransferase
NCU09116	aromatic aminotransferase Aro8
NCU04292	branched-chain-amino-acid aminotransferase
NCU08216	cystathionine beta-synthase

Table S6. Categories of proteins enriched the pool of upregulated proteins in ΔPor-1. Categories were determined as described in the legend to Figure / .

A. Proteins in functional category 02 (Energy)

02.01 tricarboxylic-acid pathway (citrate cycle, Krebs cycle, TCA cycle)

NCU00461	glutamate dehydrogenase-1
NCU00720	L-lactate dehydrogenase
NCU01227	succinate--CoA ligase
NCU01692	citrate synthase
NCU02366	Aconitate hydratase
NCU06482	pyruvate dehydrogenase E1 component alpha subunit
NCU07659	pyruvate dehydrogenase E2 component

02.13.03 aerobic respiration

NCU01808	cytochrome c-1
NCU02162	SURF-family protein
NCU02514	ATPase-1
NCU02549	mitochondrial-processing peptidase subunit beta
NCU05430	ATPase-2
NCU06086	Q subcomponent-binding protein, mitochondrial
NCU06141	protoheme IX farnesyltransferase
NCU08004	electron transfer flavoprotein alpha subunit

B. Proteins in functional category 01 (Metabolism)

01.05 C-compound and carbohydrate metabolism

NCU00350	epoxide hydrolase
NCU01092	3-oxoacyl-(acyl-carrier-protein) reductase
NCU03068	pyridoxine-3
NCU03949	Nitronate monooxygenase
NCU05041	Trehalose 6-phosphate synthase
NCU06482	Pyruvate dehydrogenase E1 component alpha subunit
NCU07008	Carotenoid oxygenase-1
NCU07659	Pyruvate dehydrogenase E2 component
NCU07737	Salicylate hydroxylase
NCU08044	Malonic semialdehyde reductase
NCU08402	Zinc-binding alcohol dehydrogenase

NCU09798	aryl-alcohol dehydrogenase
NCU10572	short chain oxidoreductase
NCU00720	L-lactate dehydrogenase
NCU00865	oxalate decarboxylase
NCU01227	succinyl-CoA synthetase alpha subunit
NCU01272	mitochondrial presequence protease
NCU01692	citrate synthesis
NCU02097	short chain dehydrogenase
NCU02366	aconitate hydratase
NCU04483	epoxide hydrolase
NCU07008	Carotenoid oxygenase 1
NCU06441	2-hydroxyglutarate---pyruvate transhydrogenase
NCU09519	2,5-diketo-D-gluconic acid reductase A
NCU09559	clock-controlled gene-9

01.05.02.07 sugar, glucoside, polyol, and carboxylate catabolism

NCU00720	L-lactate dehydrogenase
NCU00865	oxalate decarboxylase
NCU01227	Succinyl-CoA synthetase alpha subunit
NCU01692	Citrate synthesis
NCU02097	short chain dehydrogenase
NCU02366	Aconitate hydratase
NCU04483	epoxide hydrolase
NCU05594	L-galactose dehydrogenase
NCU06441	2-hydroxyglutarate---pyruvate transhydrogenase
NCU06687	glycogen synthase-1
NCU07240	Aflatoxin B1 aldehyde reductase member 2
NCU08384	xylose reductase
NCU09519	2,5-diketo-D-gluconic acid reductase A

C. Proteins in functional category 20.03.22 transport ATPases

NCU01207	vacuolar membrane ATPase-1
NCU02514	ATPase-1
NCU05430	ATPase-2
NCU07546	multidrug resistance protein MDR
NCU08515	vacuolar membrane ATPase-2
NCU0**7753**	multidrug resistance protein 3

D. Proteins in functional category 32 (cell rescue, defense and virulence)

32.01 stress response

NCU00355	catalase-3
NCU00465	chaperone dnaJ
NCU01589	heat shock protein 60
NCU01808	cytochrome c-1
NCU02113	ubiquitin-conjugating enzyme E2 13
NCU03068	pyridoxine-3
NCU03739	ERP38 protein
NCU03786	serine/threonine-protein phosphatase 2A regulatory subunit B
NCU04069	3'-phosphoadenosine 5'-phosphatase isoform B
NCU05041	trehalose-phosphatase
NCU05143	rds1
NCU06372	ubiquitin hydrolase L3
NCU08693	heat shock protein 70-5
NCU09559	clock-controlled gene-9

32.01.05 heat shock response

NCU00465	chaperone dnaJ
NCU01166	cAMP-dependent protein kinase regulator
NCU01589	heat shock protein 60
NCU08384	xylose reductase
NCU08693	heat shock protein 70-5

32.07.07.01 catalase reaction

NCU00355	catalase-3
NCU05770	catalase-2

32.07.07.03 glutathione conjugation reaction

NCU00173	esterase D
NCU00549	glutathione transferase omega-1
NCU05780	glutathione S-transferase-1

32.07.07.07 superoxide metabolism

NCU00350	epoxide hydrolase
NCU07851	superoxide dismutase 1 copper chaperone
NCU09560	superoxide dismutase
